# Effects of diet on the outcomes of rheumatic and musculoskeletal diseases (RMDs): systematic review and meta-analyses informing the 2021 EULAR recommendations for lifestyle improvements in people with RMDs

**DOI:** 10.1136/rmdopen-2021-002167

**Published:** 2022-05-11

**Authors:** James M Gwinnutt, Maud Wieczorek, Javier Rodríguez-Carrio, Andra Balanescu, Heike A Bischoff-Ferrari, Annelies Boonen, Giulio Cavalli, Savia de Souza, Annette de Thurah, Thomas E Dorner, Rikke Helene Moe, Polina Putrik, Lucía Silva-Fernández, Tanja Stamm, Karen Walker-Bone, Joep Welling, Mirjana Zlatković-Švenda, Francis Guillemin, Suzanne M M Verstappen

**Affiliations:** 1 Centre for Epidemiology Versus Arthritis, Faculty of Biology, Medicine and Health, The University of Manchester, Manchester, UK; 2 EA 4360 Apemac, Université de Lorraine, Nancy, France; 3 Center on Aging and Mobility, University of Zurich, Zurich, Switzerland; 4 Area of Immunology, Department of Functional Biology, Universidad de Oviedo, Oviedo, Spain; 5 Department of Metabolism, Instituto de Investigación Sanitaria del Principado de Asturias (ISPA), Oviedo, Spain; 6 Department of Internal Medicine and Rheumatology, ‘Sf Maria’ Hospital, ‘Carol Davila’ University of Medicine and Pharmacy, Bucharest, Romania; 7 Department of Aging Medicine and Aging Research, University Hospital Zurich and University of Zurich, Zurich, Switzerland; 8 University Clinic for Aging Medicine, City Hospital Zurich - Waid, Zurich, Switzerland; 9 Department of Internal Medicine, Division of Rheumatology, Maastricht University Medical Center, Maastricht, The Netherlands; 10 Care and Public Health Research Institute (CAPHRI), Maastricht University, Maastricht, The Netherlands; 11 Unit of Immunology, Rheumatology, Allergy and Rare Diseases, IRCCS San Raffaele Hospital and Vita-Salute San Raffaele University, Milan, Italy; 12 Centre for Rheumatic Diseases, King's College London, London, UK; 13 Department of Clinical Medicine, Aarhus University, Aarhus, Denmark; 14 Department of Rheumatology, Aarhus University Hospital, Aarhus, Denmark; 15 Centre for Public Health, Department of Social and Preventive Medicine, Medical University of Vienna, Vienna, Austria; 16 Social Insurance Fund for Public Service, Railway and Mining Industries, Sitzenberg-Reidling, Austria; 17 Karl-Landsteiner Institute for Health Promotion Research, Sitzenberg-Reidling, Austria; 18 National Advisory Unit for Rehabilitation in Rheumatology, Division of Rheumatology and Research, Diakonhjemmet Hospital, Oslo, Norway; 19 Rheumatology Department, Hospital Universitari Son Espases, Palma de Mallorca, Spain; 20 Section for Outcomes Research, Center for Medical Statistics, Informatics, and Intelligent Systems, Medical University of Vienna, Vienna, Austria; 21 Ludwig Boltzmann Institute for Arthritis and Rehabilitation, Vienna, Austria; 22 MRC Versus Arthritis Centre for Musculoskeletal Health and Work, University of Southampton, Southampton, UK; 23 NVLE Dutch Patient Organization for Systemic Autoimmune Diseases, Utrecht, The Netherlands; 24 Institute of Rheumatology, University of Belgrade School of Medicine, Belgrade, Serbia; 25 Department of Internal Medicine, University of East Sarajevo Faculty of Medicine Foča, Republika Srpska, Bosnia and Herzegovina; 26 Inserm, CHRU Nancy, CIC-1433 Epidémiologie Clinique, Université de Lorraine, Nancy, France; 27 NIHR Manchester Biomedical Research Centre, Manchester University NHS Foundation Trust, Manchester Academic Health Science Centre, Manchester, UK

**Keywords:** epidemiology, arthritis, patient reported outcome measures

## Abstract

**Background:**

A EULAR taskforce was convened to develop recommendations for lifestyle behaviours in rheumatic and musculoskeletal diseases (RMDs). In this paper, the literature on the effect of diet on the progression of RMDs is reviewed.

**Methods:**

Systematic reviews and meta-analyses were performed of studies related to diet and disease outcomes in seven RMDs: osteoarthritis (OA), rheumatoid arthritis (RA), systemic lupus erythematosus, axial spondyloarthritis, psoriatic arthritis, systemic sclerosis and gout. In the first phase, existing relevant systematic reviews and meta-analyses, published from 2013 to 2018, were identified. In the second phase, the review was expanded to include published original studies on diet in RMDs, with no restriction on publication date. Systematic reviews or original studies were included if they assessed a dietary exposure in one of the above RMDs, and reported results regarding progression of disease (eg, pain, function, joint damage).

**Results:**

In total, 24 systematic reviews and 150 original articles were included. Many dietary exposures have been studied (n=83), although the majority of studies addressed people with OA and RA. Most dietary exposures were assessed by relatively few studies. Exposures that have been assessed by multiple, well conducted studies (eg, OA: vitamin D, chondroitin, glucosamine; RA: omega-3) were classified as moderate evidence of small effects on disease progression.

**Conclusion:**

The current literature suggests that there is moderate evidence for a small benefit for certain dietary components. High-level evidence of clinically meaningful effect sizes from individual dietary exposures on outcomes in RMDs is missing.

**Key messages**

**What is already known about this subject?**

- People’s diet can influence health related outcomes, such as cardiovascular outcomes and mental health.
- It is unclear whether dietary factors influence rheumatic and musculoskeletal disease (RMD) specific outcomes.

**What does this study add?**

- This study brings together the literature on diet and progression of seven RMDs, concluding that research on diet has largely focused on osteoarthritis and rheumatoid arthritis, and there is little evidence suggesting dietary factors can make large differences to the outcomes of people with RMDs.

**How might this impact on clinical practice or further developments?**

- Based on the current literature, health professionals can advise people with RMDs that consuming specific dietary components is unlikely to influence the progression of their RMD, but that it is important to maintain a healthy diet and healthy weight for general health reasons.

Rheumatic and musculoskeletal diseases (RMDs) are a diverse range of conditions that primarily affect people’s joints, causing pain, disability and reductions in health-related quality of life (HR-QoL).^1–3^ According to the Global Burden of Disease study, RMDs are one of the leading causes of global disability.^4 5^ Some RMDs have effective pharmacological treatments that limit disease progression (eg, rheumatoid arthritis (RA)^6^), whereas others have no effective disease modifying treatment options (eg, osteoarthritis (OA)^7^). However, in all RMDs there is room for additional improvement in outcomes. In the general population, lifestyle modifications have been shown to improve non-RMD related outcomes. For instance, diet (ie, specific food stuffs ingested as part of daily living, and supplements or nutrients ingested to improve health) has a significant impact on the risk of chronic disease^8^ and benefits to mental health.^9^ However, it is unclear whether lifestyle modifications, such as changes to diet, have a beneficial impact on RMD related outcomes (including disease activity, pain, function, HR-QoL, radiographic damage, fatigue and depression).

In 2018, a EULAR Taskforce was convened to develop recommendations for lifestyle improvements in people with RMDs with regards to RMD progression (including both modifiable (eg, pain, fatigue) and irreversible (eg, joint damage) outcomes).^10^ The taskforce decided to focus on six lifestyle factors: diet, exercise, weight, alcohol, smoking and paid work, and seven diseases: RA, OA, axial spondyloarthritis (axSpA), psoriatic arthritis (PsA), systemic lupus erythematosus (SLE), systemic sclerosis (SSc) and gout (henceforth referred to collectively as RMDs). For each of these lifestyle factors, systematic reviews were performed, aiming to collate all relevant literature on each factor in order to formulate evidence based recommendations. This article reports the results of systematic reviews on the effect of diet on progression of RMDs.

## Methods

### Design

This study was performed in accordance with the EULAR standard operating procedure for EULAR endorsed recommendations^11^ and is reported following the PRISMA (Preferred Reporting Items for Systematic Reviews and Meta-Analyses) guidelines.^12^

### Search strategy

The articles included in this review were identified in two steps. First, a systematic search was conducted aiming to identify published systematic reviews and meta-analyses on any of the included exposures and RMDs (listed below; online supplemental tables 1 and 2) published between 1 January 2013 and 18 Septemner 2018, using the Medline, Embase and Cochrane Library databases. Two reviewers screened the titles and abstracts (JMG, MW) and then a team of four reviewers screened the eligible full texts (JMG, MW, JRC, GC; two reviewers per full text). Only systematic reviews and meta-analyses related to diet are presented in this report.

10.1136/rmdopen-2021-002167.supp1 Supplementary data

Then a systematic review of original articles of dietary interventions for people with RMDs was conducted. Where the research team agreed that sufficient systematic reviews and meta-analyses had been published on a given exposure in a given disease, these exposures were excluded from the systematic review of original articles (OA: vitamin E, bromelain, glucosamine, willow bark extract, chondroitin, *Artemisia annua* extract, green lipped muscle extract, methylsulfonylmethane, avocado/soy bean unsaponifiables, L-carnitine, curcumin, pycnogenol, *Boswellia serrata* extract, C*urcuma longa* extract, passion fruit peel extract, collagen hydrolysate; RA: marine oils, omega-3, probiotics, vitamin D). The search strategy was developed based on a predefined PICO (PICO=participants, intervention/exposure, comparison, outcome (online supplemental table 3 for search strategy)) and implemented in the Medline, Embase and CENTRAL databases on 8 March 2019. Titles and abstracts followed by full texts were screened by two reviewers (JMG, JRC).

### Inclusion and exclusion criteria

For the review to identify relevant published systematic reviews and meta-analyses, the following inclusion criteria were used:

- Systematic reviews or meta-analyses of randomised controlled trials (RCTs) or observational studies.
- Including people with an RMD (OA, RA, SLE, axSpA, PsA, SSc, gout).
- Studying the relationship between diet and outcomes (see online supplemental table 4 for a list of included outcomes).

For the review identifying original studies of dietary exposures in RMDs, the following inclusion criteria were used:

- Longitudinal study design (randomised trials, non-randomised trials, single-arm intervention studies, longitudinal observational studies).
- Including adults with an RMD (OA, RA, SLE, axSpA, PsA, SSc, gout).
- Studying the relationship between dietary exposures and outcomes (see online supplemental table 4 for a list of included outcomes).

Conference abstracts were excluded.

### Risk of bias assessment

The AMSTAR-2 tool was used to assess the risk of bias in published systematic reviews and meta-analyses.^13^ Each review was graded as critically low, low, moderate or high quality. The Cochrane Risk of Bias tool was used to assess methodological quality of included RCTs,^14^ rating the reporting of four criteria: randomisation procedure, allocation concealment procedure, blinding of participants and blinding of assessors. Each aspect was graded as either low risk of bias, or high/unclear risk of bias. The process was aided by a machine-learning algorithm that identifies passages and estimates a grade for each category. This has been demonstrated to speed up the quality assessment process.^15^ A reviewer (JMG) checked each of the algorithm’s estimates and the passages that the algorithm was using to make these estimates, and made any changes to grades where the algorithm did not identify suitable passages. The QUIPS tool was used to assess the quality of observational studies of diet.^16^

### Synthesis of data

Due to the heterogeneity of the studies, the findings from the included studies are presented in the form of a narrative summary, sorted by RMD and then by category of diet exposure (animal products; experimental diets; food components; fruits, vegetables and other plant-based interventions; minerals and supplements; vitamins). For each exposure, results from systematic reviews are presented first where available, followed by results from individual studies published after the reviews. Where no reviews were identified, results from individual studies are presented.

If possible, the results of RCTs were pooled using random effects meta-analysis. Standardised mean differences (SMDs) were calculated if possible for individual studies and combined in meta-analyses as this allows results measured on different instruments to be combined (SMDs in online supplemental tables). An SMD is estimated as the difference between the scores of the intervention and control group at follow-up divided by the pooled SD.^17^ The means and SDs were extracted from each RCT, or effect estimates (eg, ORs, relative risk ratios, adjusted where available) from observational studies. SDs were estimated from 95% confidence intervals or standard errors when not reported. Means and SDs were estimated from medians and ranges or IQRs when only these summary statistics were presented using a published formula.^18^ Overall, a SMD≥0.2 was considered a small effect, ≥0.5 as a medium sized effect, and ≥0.8 as a large effect.^19^ Heterogeneity was quantified using the I^2^ statistic. All statistical analyses were performed using Stata version 14 (StataCorp, College Station, TX).

The Grading of Recommendations, Assessment, Development and Evaluations (GRADE) system defines high quality evidence as evidence where further research is very unlikely to change our confidence in the estimate of effect.^20^ Therefore, evidence was rated as high quality if supported by meta-analyses of at least five RCTs at low-moderate risk of bias, reporting consistent results without important limitations.^21^ GRADE defines moderate quality evidence as evidence where further research is likely to have an important impact on the confidence of the estimate of effect, or may change the estimate.^20^ Evidence was rated as moderate if supported by meta-analyses of at least three RCTs or supported by a single RCT with a sample size ≥100 and at low-moderate risk of bias or multiple large observational studies. GRADE defines low quality evidence as evidence where further research is very likely to have an important influence on our confidence in the estimates, or is likely to change the estimate.^20^ Evidence was rated as low if supported by multiple RCTs of small sample size or high risk of bias, or by single observational studies only. GRADE defines very low quality of evidence as evidence where the estimate of the effect is very uncertain.^20^ Evidence was rated as very low if supported by single small RCTs, or non-randomised trials or single arm intervention studies. Evidence could be downgraded in the event of other potential biases (such as study limitations, inconsistency of results, imprecision, publication bias^21^ or conflicts of interest).

## Results

### Study selection and study characteristics

**Figure 1:**
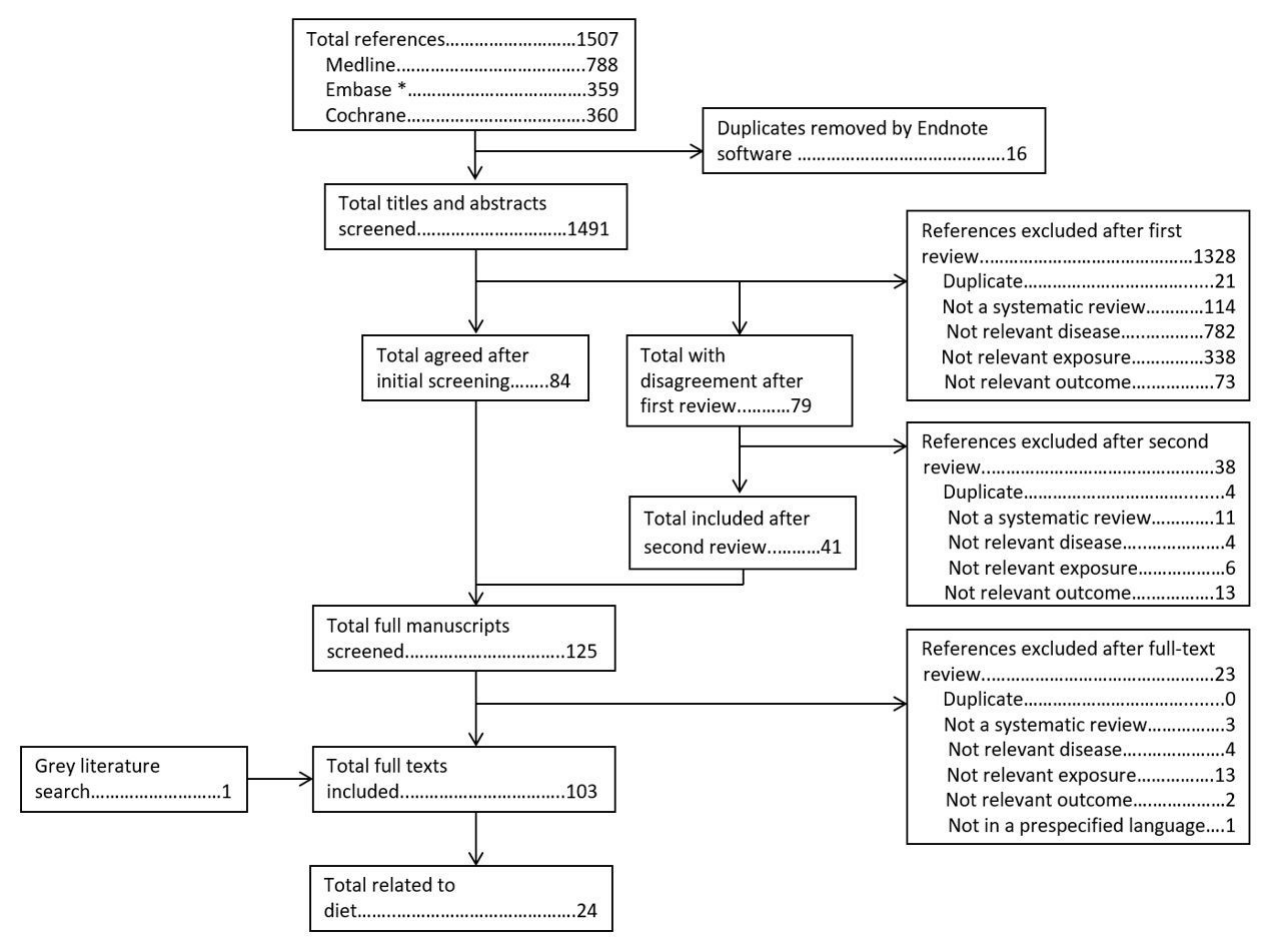
Flowchart of search strategy to identify published systematic reviews and meta-analyses. *Embase search excluded journals included in Medline.

The search of systematic reviews and meta-analyses yielded 1507 abstracts, of which 16 were duplicates. Of these, 125 full manuscripts were screened, of which 103 were included (figure 1). Only 24 assessed diet and progression of RMDs, and are included in this review (other references assessed other exposures within the taskforce; eg, exercise, smoking).

**Figure 2:**
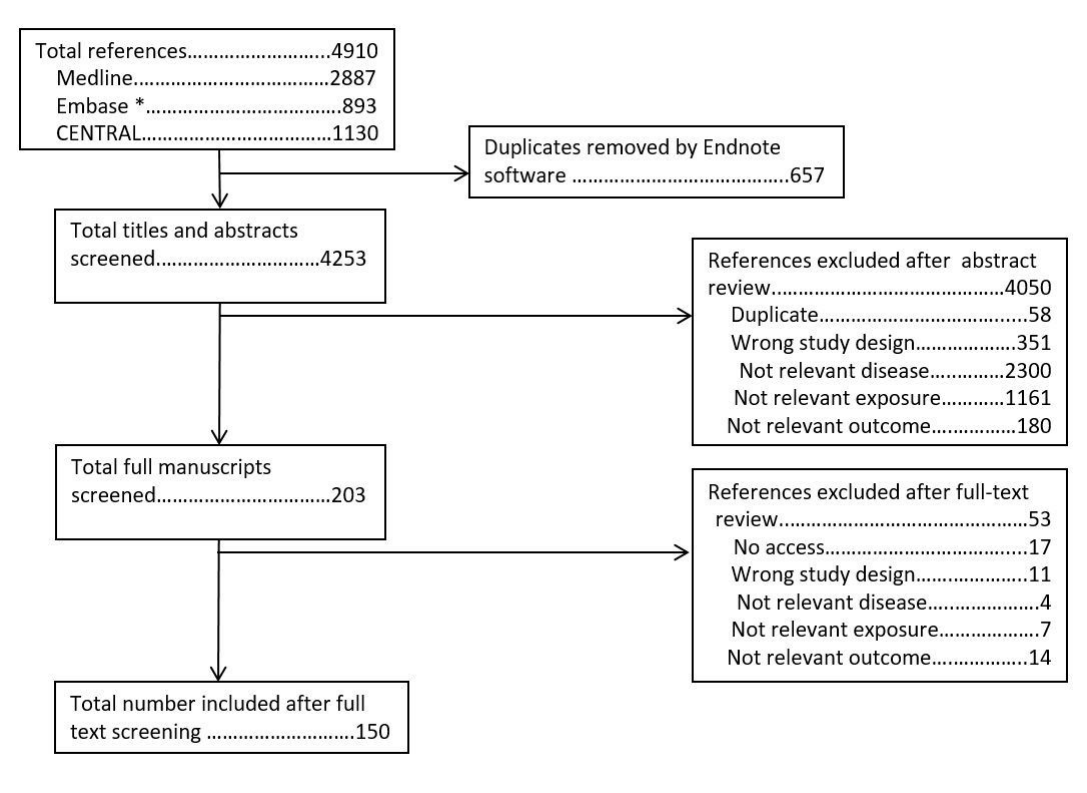
PRISMA (Preferred Reporting Items for Systematic Reviews and Meta-Analyses) flowchart for review of individual studies of diet. *Embase search excluded journals included in Medline.

The search for original studies identified 4910 abstracts. After removal of 657 duplicates, 4253 titles and abstracts were screened. Of these, 203 full manuscripts were screened, of which 150 are included in this article (figure 2).

### Osteoarthritis

#### Animal products

In total, two systematic reviews,^22 23^ 10 RCTs,^24–33^ one single-arm intervention^34^ and one prospective cohort study^35^ assessed animal products in OA. One meta-analysis^22^ reported moderate effects of undenatured type II collagen on pain and function (pain: SMD −0.67, 95% CI −1.01 to –0.33; function: SMD −0.55, 95% CI −0.94 to –0.17). Milk consumption was studied by one prospective cohort study, which reported a reduction in joint space narrowing as milk consumption increased.^35^ One small RCT reported moderate effects on pain, function and stiffness following egg-shell membrane consumption.^30^ An RCT studying *Channa striatus* extract (common name: striped snakehead fish) reported moderate sized effects for pain, function and stiffness.^24^ One meta-analysis^23^ and four RCTs studying fish oil supplements were included. The meta-analysis reported small, non-significant effects of fish oil on pain (SMD −0.16, 95% CI −0.57 to 0.24) and function (SMD 0.11, 95% CI −0.13 to 0.35) in OA.^23^ One meta-analysis of one RCT^22^ reported a small effect of green-lipped mussel extract on pain that was not significant (SMD −0.37, 95% CI −0.81 to 0.08). Promerim was assessed by one single-arm intervention study.^34^ Pain improved after receiving promerim and exercise (online supplemental tables 5–13).

#### Experimental diets

One meta-analysis,^36^ three RCTs^37–39^ and one single arm intervention study^40^ assessed experimental diets for OA. One meta-analysis compared dietary restriction plus exercise versus exercise controls, reporting small benefits in favour of pain (SMD −0.24, 95% CI −0.50 to 0.02) and function (SMD −0.34, 95% CI −0.59 to –0.08).^36^ One RCT reported no difference in pain or function between low and very low calorie diets.^39^ A single arm intervention study reported reductions in pain, functional limitations and stiffness when following a low calorie diet,^40^ and a small scale RCT reported improvements on several sub-scales of the SF36 from a whole-food, plant based diet in OA.^38^ Another small scale RCT reported no benefit of the Mediterranean diet for physical function in people with OA^37^ (online supplemental tables 14–18).

#### Food components

Two prospective cohort studies using data from the Osteoarthritis Initiative assessed the association between specific food components and OA progression.^41 42^ One large prospective study reported that higher fibre intake was associated with lower odds of being in high pain trajectories.^41^ The other reported that higher fat intake was associated with faster joint space narrowing progression^42^ (online supplemental tables 19 and 20).

#### Fruits, vegetables and other plant based interventions

In total, three meta-analyses,^22 43 44^ two systematic reviews,^7 45^ 20 RCTs^46–65^ and one single arm intervention study^66^ assessing fruit and vegetables were identified. *Artemisia annua* extract was included in one meta-analysis,^22^ reporting no significant benefit on pain (SMD −0.37, 95% CI −1.03 to 0.29) and function (SMD −0.15, 95% CI −0.81 to 0.50). Avocado and soybean unsaponifiables (ASU) were assessed by two meta-analyses^22 44^ and two systematic reviews.^7 45^ One meta-analysis reported moderate sized effects of ASUs on pain (SMD −0.57, 95% CI −0.95 to –0.19) and function (SMD −0.48, 95% CI −0.69 to –0.28),^22^ whereas the other reported small effects (pain: 8% reduction, 95% CI 1% to 16%; function: 7% reduction, 95% CI 2% to 12%).^44^ One systematic review^45^ reported that only one out of four RCTs reported a significant effect on pain; the other^7^ identified a meta-analysis^67^ reporting a small effect on pain. Two meta-analyses^22 44^ assessed *Boswellia serrata* extract, both reporting significant effects on pain and function, one reporting large effects (pain: SMD −1.61, 95% CI −2.10 to –1.13; function: SMD −1.15, 95% CI −1.63 to –0.68), the other moderate effects (pain, 100 point scale: −17, 95% CI −26 to –8; function, 100 point scale: −8, 95% CI −14 to –2). Bromelain was included in one meta-analysis, which reported no significant effect on pain (SMD −0.05, 95% CI −0.75 to 0.64) and a small, non-significant effect on function (SMD −0.34, 95% CI −1.04 to 0.36).^22^ One meta-analysis^22^ including one RCT^68^ assessing *Curcuma longa* reported large effects on pain (SMD −1.63, 95% CI −2.22 to –1.03) and function (SMD −1.27. 95% CI −1.83 to –0.70). The same meta-analysis^22^ included two RCTs assessing curcumin, again reporting large effects on pain (SMD −1.19, 95% CI −1.93 to –0.45) and function (SMD −1.13, 95% CI −1.80 to –0.46). Two RCTs assessed fruit powders of *Elaeagnus angustifolia* (Russian olive), reporting small to moderate sized effects on pain and function.^50 63^ Passion fruit was included in one meta-analysis,^22^ which identified one RCT^69^ reporting large effects on pain (SMD −1.65, 95% CI −2.44 to –0.86) and function (SMD −1.55, 95% CI −2.33 to –0.77). *Rosa canina* mix was studied by three RCTs. Two^58 60^ reported moderate to large effects of *Rosa canina* mix on pain and function. The third was a crossover study^59^ and reported the effects only when the placebo was taken first, indicating crossover effects. All three studies were funded by companies producing the intervention. Two papers reported on the same RCT assessing sesame powder, reporting a medium sized significant effect on pain.^49 51^ One meta-analysis^22^ and three RCTs^63–65^ assessed various tree bark extracts. The meta-analysis reported large effects of pine tree extract on pain (SMD −1.21, 95% CI −1.53 to –0.89) and function (SMD −1.84, 95% CI −2.32 to –1.35). One RCT reported a large effect of *Phellodendron* on pain in normal weight people with OA, but not overweight people.^64^ One meta-analysis^43^ reported a moderate sized effect of turmeric on pain (pooled mean difference −15.36, 95% CI –26.94 to –3.77). One RCT reported no effect on C-reactive protein (CRP) and 6 min walk test of *Scutellaria baicalensis* and *Acacia catechu*.^61^ RCTs tested aquamin,^55^ argan oil,^56^ cherry juice,^53 54^ garlic capsules,^46^ ginger,^52^ green tea extract,^57^ pomegranate,^47 48^ seaweed extract,^62^ and *Elaeagnus angustifoli* and *Boswellia Thurifera*,^63^ reporting no consistent effects on pain and function (online supplemental tables 21–43).

#### Minerals and supplements

Two meta-analyses,^22 70^ three systematic reviews,^7 45 71^ four RCTs^72–75^ and one single arm study^76^ assessed various minerals and supplements for OA. Two meta-analyses studied chondroitin for OA, one including nine studies^22^ and the other 12,^70^ and reported a small effect of chondroitin on pain (SMD −0.34, 95% CI −0.49 to –0.19^22^; SMD −0.51, 95% CI −0.74 to –0.28)^70^ and an inconsistent effect on function (SMD −0.36, 95% CI −0.58 to –0.13^22^; SMD 0.11, 95% CI −0.47 to 0.68).^70^ However, two systematic reviews concluded that chondroitin was not associated with reductions in pain.^45 71^ A third cited a range of meta-analyses reporting a wide range of effect sizes.^7^ One meta-analysis and two systematic reviews included glucosamine for OA. The meta-analysis^22^ reported small effect sizes for pain (SMD −0.28, 95% CI −0.52 to –0.04) and function (SMD −0.45, 95% CI −0.73 to –0.17). The systematic reviews^7 45^ identified a Cochrane review^77^ that reported a moderate sized benefit for pain as well as a large scale RCT^78^ that reported a null effect. One meta-analysis included three RCTs assessing methylsulfonylmethane supplementation, reporting a small effect on pain (SMD −0.47, 95% CI −0.80 to –0.14) and a large effect on function (SMD −1.10, 95% CI −1.81 to –0.38).^22^ RCTs reported large effects on CRP and erythrocyte sedimentation rate from calcium fructobate,^74^ and medium-large effects on pain and function from creatine,^73 75^ L-carnitine^22^ and *Lactobacillus casei shirota*.^72^ One single arm intervention tested a multi-mineral containing 72 natural minerals, reporting improvements in pain and function following the intervention^76^ (online supplemental tables 44–53).

#### Vitamins

In total, three meta-analyses,^22 79 80^ three systematic reviews,^71 81 82^ eight RCTs^83–90^ and three prospective cohort studies^91–93^ were identified studying vitamin supplementation for OA. RCTs testing multi-vitamins,^88^ vitamin B3^89^ and vitamin B12^90^ reported small non-significant effects on pain. One prospective cohort study reported that self-reported vitamin C supplementation was not associated with lower risk of radiographic progression.^91^ One RCT compared vitamin E+C versus placebo and reported a small effect on pain after 8 weeks.^87^ Three meta-analyses,^22 79 80^ three systematic reviews,^71 81 82^ four RCTs^83–86^ and one prospective cohort study^93^ were identified that studied the effect of vitamin D. The meta-analyses reported small effects on pain and function as a result of vitamin D (pain: SMD −0.19, 95% CI −0.31 to –0.06^22^; SMD −0.32, 95% CI −0.63 to –0.02,^79^ mean difference in The Western Ontario and McMaster Universities Osteoarthritis Index (WOMAC) −1.65, 95% CI −2.16 to –1.14^80^; function: SMD −0.36, 95% CI −0.61 to –0.11,^22^ mean difference WOMAC −1.87, 95% CI –2.58 to –1.17).^80^ The systematic reviews reported no effect on pain, but a small effect on function. Three out of six observational studies included in a systematic review reported an inverse relationship between vitamin D and radiographic progression.^82^ The latest RCT not included in the meta-analyses reported no difference in pain between high and low doses of vitamin D after participants underwent total knee replacement.^83^ One meta-analysis^22^ and one systematic review^71^ studied vitamin E. Both reported no effect of vitamin E on pain (SMD 0.01, 95% CI −0.44 to 0.45) and the meta-analysis reported no effect of vitamin E on function (SMD −0.10, 95% CI −0.55 to 0.35) (online supplemental tables 54–62).

#### Summary

There were only relatively few studies for most dietary exposures in OA, meaning that the evidence for these exposures was graded as low or very low (table 1). For diets that had moderate evidence (fish oil, chondroitin, glucosamine, vitamin D, ASU), the effect sizes for outcomes were generally small and therefore not clinically relevant.

**Table 1:** Osteoarthritis results summary

Quality/effect size	None	Small	Medium	Large
Very low	Artemisia annua (function), bromelain (pain), ginger (pain), green tea extract (pain, function), Mediterranean diet (function), *Scutellaria baicalensis* and *Acacia catechu* (6MWT, CRP), *Uncaria guianensis* extract (pain), WFPB diet (pain), vitamin B12 (pain)	Aquamin (pain), *Artemisia annua* (pain), bromelain (function), vitamin B3 (pain), vitamin C+E (pain)	Aquamin (function), creatine (pain, function), egg-shell membrane (pain, function, stiffness), multi-mineral (pain, function), promerim (pain), seaweed extract (pain, function), sesame powder (pain), turmeric (pain)	Calcium fructobate (CRP, ESR), L-carnitine (pain, function), passion fruit extract (pain, function), *Phellodendron amurense* extract (pain)
Low	Cherry juice (pain, function), *Elaeagnus angustifoli* extract +*Boswellia thurifera* (pain, function), garlic (pain, function), pomegranate (pain, function), vitamin C (radiographic progression), vitamin E (pain, function)	Argan oil (pain), *Channa striatus* (pain), *Elaeagnus angustifoli* extract (pain, function), fruit powder (pain, function), green lipped mussel extract (pain), low calorie diet (pain, function), methylsulfonylmethane (pain), multi-vitamins (pain), vitamin D (radiographic progression)	Argan oil (function), *Boswellia Serrata* (pain, function), *Channa striatus* (function), collagen (pain, function), fibre (pain), fat (JSW), *Lactobacillus casei shirota* (stiffness), milk (JSW)	*Curcuma longa* (pain, function), curcumin (pain, function), *Lactobacillus casei shirota* (pain, function), methylsulfonylmethane (function), pine tree extract (pain, function), *Rosa canina* mix (pain, function)
Moderate	Fish oil (pain, function)	ASU (pain, function), chondroitin (pain, function), glucosamine (pain, function), vitamin D (pain, function)	–	–
High	–	–	–	–

ASU, avocado and soybean unsaponifiable; CRP, C-reactive protein; ESR, erythrocyte sedimentation rate; JSW, joint space width; 6MWT, 6 min walk test; WFPB, whole food plant based.

### Rheumatoid arthritis

#### Animal products

In total, three meta-analyses,^23 94 95^ one systematic review,^96^ 12 RCTs^97–107^ and one non-randomised trial^108^ assessed products derived from animals for RA. One RCT compared collagen extracted from pigskins with placebo, reporting no significant effect on pain, function and disease activity.^99^ Three meta-analyses,^23 94 95^ one systematic review,^96^ eight RCTs^97 98 100–105^ and one non-randomised trial^108^ studied the effect of fish oils and omega-3 on RA progression. The meta-analyses reported small effects of fish oils on pain (SMD −0.32, 95% CI −0.59 to –0.05^94^; SMD −0.21, 95% CI −0.42 to –0.00),^23^ with the same results from the meta-analysis of identified RCTs (SMD −0.27, 95% CI −0.54 to 0.00) (online supplemental figure 3). One meta-analysis reported a small, significant effect on function (SMD −0.26, 95% CI −0.46 to –0.06),^94^ whereas another reported no effect (SMD 0.05, 95% CI −0.11 to 0.21).^23^ One RCT reported an improvement in disease activity,^97^ whereas another reported no effect.^98^ Two RCTs studied the effect of mussel extracts,^106 107^ reporting small non-significant effects on pain and function (online supplemental tables 63–67).

#### Experimental diets

One meta-analysis,^95^ 12 RCTs,^109–120^ five non-randomised trials,^121–125^ one single arm study^126^ and one extension to an RCT comparing responders with non-responders^127^ studied experimental diets in RA. Multiple RCTs and non-randomised trials studied liquid elemental diets,^109 113 115 116^ hypoallergenic diets,^112 117 125^ ketogenic diets^124^ and vegetarian or vegan diets,^111 114 118–120 123 126 127^ reporting no effect on the majority of outcomes assessed, including pain, function, joint counts, acute phase reactants, and morning stiffness. One meta-analysis,^95^ one RCT^110^ and two non-randomised trials^121 122^ studied the Mediterranean diet for RA. The meta-analysis reported no significant effect of the Mediterranean diet on fatigue (SMD 0.37, 95% CI −0.18 to 0.93).^95^ The RCT^110^ reported a large effect of the diet on pain and a small effect on disease activity (online supplemental tables 68–74).

#### Fruits, vegetables and other plant based interventions

One meta-analysis,^95^ eight RCTs,^128–135^ one non-randomised trial^136^ and three single arm studies^137–139^ assessed plant based interventions for RA. RCTs tested microalgae oil,^128^ herbal medicine,^135^ pomegranate extract,^129^ quercetin^130 132 134^ and rose hip powder,^133^ reporting no consistent effects on outcomes, including pain, function, disease activity, joint counts and QoL. *Andrographis paniculata* was included in one meta-analysis, which reported no effect on fatigue.^95^ One RCT^131^ investigated a combination of ginger, curcumin and black pepper for RA and reported large effects on the Disease Activity Score 28 (DAS28) and its components. One single arm intervention study assessed gum arabic powder, concluding that DAS28 and its components fell after administration.^137^ However, there was no control group. One non-randomised trial of *Nigella sativa* oil reported moderate benefits in terms of pain, disease activity, tender and swollen joints and morning stiffness^136^ (online supplemental tables 75–85).

#### Minerals and supplements

Two meta-analyses,^140 141^ 14 RCTs^134 142–153^ and one single arm study^154^ assessed minerals and supplements for RA. Two meta-analyses^140 141^ assessed studies on probiotics in RA, both reporting either small non-significant effects or no effects on function (SMD −0.30, 95% CI −0.89 to 0.29^140^; MD −0.11, 95% CI −0.23 to 0.01),^141^ swollen joint count (SMD −0.30, 95% CI −0.62 to 0.02^140^; MD 0.17, 95% CI −0.39 to 0.73)^141^ and CRP (SMD −0.32, 95% CI −0.65 to 0.00; MD −1.40, 95% CI −4.06, 1.26)^141^ and an inconsistent effect on disease activity (SMD −0.58, 95% CI −0.97 to –0.19^140^; MD 0.02, 95% CI −0.58 to 0.63).^141^ RCTs tested alpha lipoic acid,^134 145^ co-enzyme Q10,^144^ creatine,^143^ glucosamine,^148^ linoleic acid,^147^ manganese^152^ and zinc,^150 151^ reporting no effects on outcomes including pain, function and acute phase reactants. One RCT studied ambrotose complex for RA, reporting a small effect on pain but no effect on any other outcomes.^146^ An RCT compared grape juice enriched with potassium with standard grape juice, reporting large effects in terms of pain, disease activity, tender and swollen joint counts and acute phase reactants.^153^ An RCT assessing a combination of supplements (beta-hydroxy-beta-methylbutyrate, glutamine and arginine) reported moderate effects on disease activity, function and fatigue^149^ (online supplemental tables 86–98).

#### Vitamins

One meta-analysis,^155^ six RCTs,^147 156–160^ one non-randomised trial^161^ and two single arm studies^162 163^ assessed vitamin supplementation in RA. One meta-analysis^155^ reported no significant effect of vitamin D supplementation on pain (MD 2.79, 95% CI −1.87 to 7.44) and disease activity (MD −0.31, 95% CI −0.86 to 0.25). Two RCTs reported inconsistent effects on pain, function and disease activity.^156 158^ Two RCTs studied vitamin B6, with one^157^ reporting no significant effect on disease activity, swollen/tender joint count and acute phase reactants, and the other^159^ reporting no effect on CRP. One RCT^160^ assessing vitamin E reported a large effect on pain, but no effect on swollen/tender joint counts and morning stiffness. Another RCT^147^ reported no effect of vitamin E on acute phase reactants (online supplemental tables 99–104).

#### Summary

The evidence for most dietary exposures in RA was graded as low or very low (table 2), primarily due to small numbers of studies with small sample sizes. The dietary exposures with moderate quality evidence (probiotics, vitamin D, fish oil/omega-3) showed either no effect or effect sizes that are probably not clinically significant.

**Table 2:** Rheumatoid arthritis results summary

Quality/effect size	None	Small	Medium	Large
Very low	Collagen (pain, function, disease activity), creatine (function, disease activity), elemental diets (pain, function, TJC, SJC, MS), glucosamine (CRP, ESR), herbal medicine (function, TJC, SJC), hypoallergenic diets (pain, TJC, SJC, MS), ketogenic diets (TJC, CRP, ESR), Linoleic acid (CRP, ESR), manganese (disease activity), Mediterranean diet (disease activity), microalgae oil (SJC), pomegranate (pain, function, TJC, SJC), vitamin B6 (SJC), vitamin E (TJC, SJC, MS, CRP, ESR)	Gum arabic (disease activity), herbal medicine (pain), Mediterranean diet (fatigue, function), microalgae oil (function, disease activity, TJC), mussel extracts (pain, function), quercetin (disease activity, TJC, SJC), vitamin B6 (disease activity, TJC, CRP, ESR)	*Nigella sativa* oil (pain, disease activity, TJC, SJC, morning stiffness)	Ginger+curcumin + black pepper (disease activity), Mediterranean diet (pain), potassium (pain, disease activity, TJC, SJC, CRP, ESR), vitamin E (pain)
Low	Alpha-lipoic acid (pain, CRP), ambrotose complex (function), *Andrographis Paniculata* (fatigue), rose hip powder (function, QoL), vegetarian/vegan diet (pain, function),	Alpha-lipoic acid (function), ambrotose complex (pain), antioxidant combinations (disease activity), co-enzyme Q10 (CRP), rose hip powder (pain, disease activity), zinc (pain, function)	Beta-hydroxy-beta-methylbutyrate+glutamine + arginine (disease activity, function, fatigue), quercetin (pain, function)	Antioxidant combinations (pain, function)
Moderate	Fish oil/omega-3 (function, disease activity), probiotics (function, SJC, CRP, disease activity), vitamin D (pain, disease activity)	Fish oil/omega-3 (pain)	–	–
High	–	–	–	–

CRP, C-reactive protein; ESR, erythrocyte sedimentation rate; MS, morning stiffness; QoL, quality of life; SJC, swollen joint count; TJC, tender joint count.

### Systemic lupus erythematosus

#### Animal products

One systematic review,^164^ six RCTs^165–170^ and one non-randomised trial^171^ assessed fish oil/omega-3 for SLE. Two out of three studies included in the systematic review^164^ reported reductions in disease activity following omega-3 intervention. The largest RCT reported no difference in disease activity between omega-3 and placebo.^167^ Another RCT reported reductions in disease activity in the fish group from baseline and no reduction in the placebo group, but did not compare the two groups.^168^ One other RCT^166^ reported large effects on pain and function following omega-3 intervention, but no effect on fatigue. Two RCTs reported no effect of omega-3 on CRP^165 168^ (online supplemental tables 105–107).

#### Experimental diets

Three systematic reviews^164 172 173^ identified one RCT^174^ comparing a low glycaemic diet with a low calorie diet, concluding no effect on disease activity or fatigue. An RCT^175^ reported a large effect of a cholesterol lowering educational programme on QoL compared with no advice (online supplemental tables 108–110).

#### Food components

Three observational cohort studies^176–178^ assessed the association between food components and outcomes in SLE. Two cohort studies^176 177^ assessed the association between consumption of various food elements and risk of active disease and atherosclerotic vascular events. High consumption of vitamin B6, fibre and vitamin C was associated with lower risk of developing active disease. None of the food components investigated were associated with reduced risk of atherosclerotic vascular events. Another cohort study^178^ investigated poor nutrition in SLE, reporting that lower calorie intake was associated with more organ damage and lower percentage of protein was associated with worse mental health (online supplemental tables 111–113).

#### Fruits, vegetables and other plant based interventions

Two RCTs^179 180^ studied plant based interventions for SLE. One RCT^180^ reported no effect of curcumin on disease activity, and the other RCT^179^ reported no effect of green tea extract on disease activity, but significant benefit in terms of fatigue (median (IQR) at 3 months, green tea: 81 (63.1–95.5); placebo: 56.2 (28.1–84.3), p=0.006) (online supplemental tables 114–116).

#### Minerals and supplements

One RCT^169^ and one non-randomised trial^181^ studied mineral supplementation for SLE. The non-randomised trial^181^ assessed calcium+vitamin D supplementation compared with no treatment or steroid treatment. The supplements had a large effect compared with no treatment on disease activity and a moderate effect on erythrocyte sedimentation rate, but no effect compared with steroids. Another RCT^169^ assessed copper supplementation, reporting no effect on disease activity (online supplemental tables 117–119).

#### Vitamins

One meta-analysis,^155^ one systematic review^173^ and two RCTs^182 183^ studied vitamins in SLE. All studies assessed vitamin D, with all studies reporting no significant effect of vitamin D on disease activity,^155 182^ fatigue^173^ and anti-dsDNA level^155 183^ (online supplemental tables 120–122).

#### Summary

The evidence for fish oil/omega-3 for SLE was rated as moderate but showed no effect on outcomes (table 3). The evidence for all other studies was rated as low or very low.

**Table 3:** Systemic lupus erythematosus results summary

Quality/effect size	None	Small	Medium	Large
Very low	Copper (disease activity), curcumin (disease activity), fish oil/omega-3 (CRP), green tea extract (disease activity), low glycaemic (disease activity, fatigue)	Fish oil/omega-3 (ESR)	Calcium+vitamin D (ESR), green tea extract (fatigue)	Calcium+vitamin D (disease activity), cholesterol lowering education (QoL), fish oil/omega-3 (pain, function)
Low	Vitamin D (disease activity, fatigue, anti-dsDNA)	Fish oil/omega-3 (disease activity), fibre (disease activity), poor nutrition (organ damage), vitamin B6 (disease activity), vitamin C (disease activity)	–	–
Moderate	–	–	–	–
High	–	–	–	–

CRP, C-reactive protein; ESR, erythrocyte sedimentation rate; QoL, quality of life.

### Axial spondyloarthritis

#### Food components

One systematic review^184^ of 16 studies assessed various food components in axSpA. There was no association between the consumption of alpha-linoleic acid, carbohydrates, linoleic acid, long-chain omega-3 fatty acids, fibre, polyunsaturated fatty acids, protein or saturated fatty acids and disease activity or acute phase reactant levels. There was no association between fat consumption and acute phase reactant level. (online supplemental tables 123 and 124).

#### Minerals and supplements

One RCT^185^ assessed probiotic supplementation versus placebo, reporting no significant effect on pain, function, disease activity, tender/swollen joints and spinal mobility (online supplemental tables 125 and 126).

#### Summary

The evidence for dietary exposures in axSpA was rated as very low (table 4).

**Table 4:** Axial spondyloarthritis and psoriatic arthritis results summary

Quality/effect size	None	Small	Medium	Large
Very low	**axSpA** Alpha-linoleic acid (disease activity, ESR, CRP), carbohydrates (disease activity, ESR, CRP), fat (disease activity, ESR, CRP), fibre (disease activity), linoleic acid (disease activity, ESR, CRP), long-chain omega-3 fatty acids (disease activity, CRP), polyunsaturated fatty acids (disease activity, CRP), probiotics (pain, function, SJC, spinal mobility), protein (disease activity, ESR, CRP), saturated fatty acids (disease activity, ESR, CRP) **PsA** Selenium+coenzyme Q10+vitamin E (psoriasis severity)	**axSpA** Long-chain omega-3 fatty acids (ESR), polyunsaturated fatty acids (ESR), probiotics (disease activity, TJC)		**PsA** Selenium+coenzyme Q10+vitamin E (disease activity)
Low	**PsA** Marine animal oil/omega-3 (CRP)	**PsA** Marine animal oil/omega-3 (ESR)	**PsA** Marine animal oil/omega-3 (function, SJC)	–
Moderate	**PsA** Marine animal oil/omega-3 (pain, disease activity, TJC, enthesitis, patient global, psoriasis severity,	–	–	–
High	–	–	–	–

axSpA, axial spondyloarthritis; CRP, C-reactive protein; ESR, erythrocyte sedimentation rate; PsA, psoriatic arthritis; SJC, swollen joint count; TJC, tender joint count.

### Psoriatic arthritis

#### Animal products

Three RCTs^186–188^ assessed marine animal oil/omega-3 for PsA. The studies reported no significant effect on pain,^186 187^ function,^186 187^ disease activity,^186^ tender joints,^186–188^ swollen joints^186 187^ enthesitis,^186^ psoriasis severity,^186^ patient global^187^ and acute phase reactants^187 188^ (online supplemental tables 127 and 128).

#### Minerals and supplements

One RCT^189^ studied supplementation of selenium, co-enzyme Q10 and vitamin E for psoriasis with joint involvement and radiographic erosion, reporting a large effect on disease severity but no effect on psoriasis severity (online supplemental tables 129 and 130).

#### Summary

The evidence for marine animal oil/omega-3 for PsA was rated as moderate and showed no effect on outcomes (table 4). Other dietary exposures were rated as low evidence.

### Systemic sclerosis

#### Experimental diets

Two single arm studies^190 191^ assessed medical nutrition therapy for SSc. One single arm study^190^ assessed a diet and lifestyle plan, reporting improvements in patient global assessment but not in QoL. Another single arm study^191^ provided supplements for vitamin and mineral deficiencies and encouraged healthy eating. There were no significant changes on any of the SF36 dimensions (online supplemental tables 131 and 132).

#### Vitamins

Three RCTs^192–194^ studied vitamin supplementation in SSc. Two RCTs tested vitamins C and E (one also included selenium and beta-carotene) for SSc, one reporting better Rodnan Skin score at 1 month,^192^ the other reporting no difference in frequency of Raynaud’s attacks.^193^ The final RCT^194^ assessed vitamin D supplementation, reporting a large effect on Rodnan skin score at 9 months (online supplemental tables 133–135).

#### Summary

The evidence for dietary exposures in SSc was rated as low or very low (table 5).

**Table 5:** Systemic sclerosis and gout results summary

Quality/effect size	None	Small	Medium	Large
Very low	**SSc** Nutrition therapy (QoL, pain, mental health, physical health, fatigue), vitamin C+E (Raynaud’s attacks) **Gout** Vitamin C (uric acid)	**SSc** Nutrition therapy (patient global) **Gout** Herbal medicine (uric acid)	–	**SSc** Vitamin C+E (Rodnan skin score), vitamin D (Rodnan skin score)
Low	**Gout** Enriched milk powder (function, uric acid, gout flare)	**Gout** Enriched milk powder (pain)	–	–
Moderate	–	–	–	–
High	–	–	–	–

QoL, quality of life; SSc, systemic sclerosis.

### Gout

#### Animal products

Two systematic reviews^195 196^ identified one RCT^197^ which assessed enriched milk powder for gout. Pain scores were significantly lower in the intervention group, but this was judged not to be clinically significant. There was no difference between the groups in terms of function, uric acid level and gout flares (online supplemental tables 136 and 137).

#### Fruits, vegetables and other plant based interventions

One RCT^198^ studied Chinese herbal medicine and concluded it had no significant hypouricemic effect (online supplemental tables 138 and 139).

#### Vitamins

One RCT^199^ and one single arm study^200^ assessed vitamin C supplementation for gout. The control arm was treated with allopurinol in the RCT, and demonstrated greater reductions in uric acid. The single arm study reported no changes in uric acid (online supplemental tables 140 and 141).

#### Summary

The evidence for dietary exposures in gout was rated as low or very low (table 5).

### Studies of more than one RMD

One single arm study^201^ of a powdered meal replacement included people with OA and people with RA, reporting a slight improvement in the 50 foot walk test. A non-randomised trial^202^ assessing linoleic acid included people with RA and people with axSpA and reported no effect on tender or swollen joint count, morning stiffness, grip strength and ESR (online supplemental tables 142 and 143).

## Discussion

Many studies have been published assessing diet in OA and RA, with relatively fewer studies in the other RMDs. However, the majority of exposures in all RMDs have only been assessed by a handful of studies, which were often underpowered and at moderate to high risk of bias. Typically, these studies reported low effect sizes for outcomes, although some reported large effects. This could be due to publication bias^203^ or influence of commercial sponsors. When many studies have been performed (eg, chondroitin for OA^70^) or RCTs with large sample sizes have been conducted (eg, vitamin D for OA)^84 85^ the effect sizes on outcomes are small and not clinically meaningful. Therefore, based on the current evidence, there is no single dietary intervention which has substantial benefits on the outcomes of people with OA and RA.^204^ While there have been far fewer research studies published for the other included RMDs, again there is no consistent evidence that any dietary exposure significantly improves outcomes in these conditions. Despite this, people with RMDs should still aim for a healthy, balanced diet given the literature demonstrating the benefits in terms of non-RMD outcomes and lack of harms.^8 9^ Furthermore, the impact of a healthy diet on weight and body composition (ie, calorie balance) is likely important for determining outcomes. This was the focus of a separate review as part of this project.^205^

Alongside the influence of publication bias on these results, many studies were rated as having moderate or high risk of bias (see online supplementary material). Studies often failed to report on the randomisation or allocation concealment process as well as steps taken to ensure participants and assessors were blinded to group allocation. These factors could also influence the results, potentially inflating reported effect sizes. Furthermore, there was limited reporting of adverse events.

This review has a number of strengths. Its broad scope allows us to gain a global understanding of the effect of diet in RMDs. The review was conducted with rigour, with multiple assessors screening the titles, abstracts and full texts. Furthermore, appropriate quality assessment tools were utilised to assess the quality of all included studies. However, given the scope of the research question it is possible that some studies were missed in the review. This was limited as much as possible by designing and testing an extensive search strategy as well as including other published systematic reviews and meta-analyses, increasing the likelihood of including as many relevant studies as possible. Furthermore, some exposures were deemed to be sufficiently covered in previous reviews and were not included in the search of original articles. However, this may mean some articles were not included (eg, due to when they were published). Lastly, while the research team included a range of experts in rheumatology research and evidence synthesis, no specific nutritionists or dietitians were included in the authorship team.

Future research on diet in RMDs should aim for higher methodological and reporting standards. Some studies did not report data in sufficient detail for extraction and thus inclusion in this review. For example, an RCT by Kjeldsen-Kragh and colleagues from 1991 tested a vegetarian diet and fasting and reported a significant improvement in many patient reported outcomes (eg, pain, disability), but only presented data in the form of line-graphs meaning no precise data could be extracted.^206^ Furthermore, studies should be sufficiently powered with long-term follow-up. Standardised definitions for different diet exposures should be formulated to allow comparison across studies, and standard outcomes assessed. Finally, research into the additive or synergistic effect of different dietary components should be researched, given the complex and interrelated nature of people’s diets.

In conclusion, this broad systematic review of 174 published articles shows there is large heterogeneity in the literature on the effects of diet on RMD outcomes, both within and across RMDs. There are many published research studies on RA and OA, investigating a range of dietary exposures. For the other included RMDs, the current evidence base is limited. From the current evidence, there appears to be no single dietary factor which leads to meaningful improvements in RMD outcomes.

## Data Availability

No data are available.
