# Supplementary material for: Effects of diet on the outcomes of rheumatic and musculoskeletal diseases (RMDs): systematic review and meta-analyses informing the 2021 EULAR recommendations for lifestyle improvements in people with RMDs
Source: RMD Open. 2022 May 11;8(2):e002167. doi: 10.1136/rmdopen-2021-002167 (PMC9096533; doi:10.1136/rmdopen-2021-002167)
Supplement: Supplementary data [file rmdopen-2021-002167supp001.pdf]

Supplementary tables – diet review

Supplementary table 1 – Search strategy for systematic review of published reviews and meta-analyses

Category	Term
Diseases	1. Arthritis, Rheumatoid (mesh) (exp) (include all subheadings)
	2. Inflammatory $arthritis
	3. Undifferentiated arthritis
	4. RA
	5. Atrophic arthritis
	6. Proliferative arthritis
	7. Osteoarth$
	8. Arthrosis
	9. Degenerative joint disease
	10. Hypertrophic arthritis
	11. Arthropathy
	12. Polyarthritis
	13. OA
	14. Arthritis psoriatica
	15. Arthropathic psoriasis
	16. Psoriatic arthropathy
	17. Arthritis, Psoriatic (mesh) (exp) (include all subheadings)
	18. Psoria$ arthriti$ [have to uncheck “map team to subject heading”]
	19. Psoria$ arthropath$ [have to uncheck “map team to subject heading”]
	20. Undifferentiated oligoarthritis
	21. Arthritic psoriasis
	22. PsA
	23. Ankylosing spondylitis (mesh) (exp) (include all subheadings)
	24. Ankylosi$
	25. Spondyloarthr$ [have to uncheck “map team to subject heading”]
	26. Spondylarthr$ [have to uncheck “map team to subject heading”]
	27. Spondylitis (mesh) (exp) (include all subheadings)
	28. Bechtere$ [have to uncheck “map team to subject heading”]
	29. Marie-Strumpell
	30. Spinal arthritis
	31. Lupus erythematosus, systemic (mesh) (exp) (include all subheadings)
	32. systemic lupus erythematosus
	33. SLE
	34. Libman-Sacks disease
	35. Libman Sacks disease
	36. Lupus erythematosus disseminatus
	37. Disseminated lupus erythematosus
	38. Lupus syndrome
	39. Sclerosis, Systemic (mesh) (exp) (include all subheadings)
	40. SSc
	~~41. Scleros$~~ (removed because of ALS, multiple sclerosis etc.)
	42. Thibierge-Weissenbach syndrome
	43. Morphea
	44. Gout (mesh) (exp) (include all subheadings)
	45. Gout$

	46. Podagra<br>47. Tophus<br>48. Tophi<br>49. Tophaceous<br>50. Urate<br>51. Uric acid<br>52. Hyperurecemi$ [have to uncheck “map team to subject heading”]<br>53. Hyperurecaemi$ [have to uncheck “map team to subject heading”]<br>54. Hyperuricemia$<br>55. Hyperuricaemi$ [have to uncheck “map team to subject heading”]<br>56. arthritis urica<br>57. Gout acute
Life-style exposures	58. Diet (mesh) (exp) (include all subheadings)<br>59. Nutrition<br>60. Food (mesh) (exp) (include all subheadings)<br>61. Food habit$<br>62. Nutritional status (mesh) (exp) (include all subheadings)<br>63. Vitamin$ (mesh) (exp) (include all subheadings)<br>64. Antioxidant$ (mesh) (exp) (include all subheadings)<br>65. Fatty acid$ (mesh) (exp) (include all subheadings)<br>66. Carbohydrate$ (mesh) (exp) (include all subheadings)<br>67. Diet$ protein<br>68. Calcium<br>69. Fish oil$ (mesh) (exp) (include all subheadings)<br>70. Fruit (mesh) (exp) (include all subheadings)<br>71. Vegetable$ (mesh) (exp) (include all subheadings)<br>72. Micronutrient$ (mesh) (exp) (include all subheadings)<br>73. Nutriment$<br>74. Neutraceutical$<br>75. Exercis$<br>76. Strength$<br>77. Endurance<br>78. Cardiorespiratory<br>79. Aerobic<br>80. Aerobic training<br>81. Exercise program$<br>82. Exercise therap$ [have to uncheck “map team to subject heading”]<br>83. Physical education<br>84. Physical training<br>85. Physical therapy<br>86. Physiotherapy<br>87. Muscle stretching<br>88. Sport (mesh) (exp) (include all subheadings)<br>89. Bod$y Weight (mesh) (exp) (include all subheadings)<br>90. Weight change<br>91. Weight loss (mesh) (exp) (include all subheadings)<br>92. Weight reduction<br>93. Weight gain<br>94. Anti obesity<br>95. Anti-obesity<br>96. Antiobesity

	97. Slimming<br>98. Smok$<br>99. Smoking (mesh) (exp) (include all subheadings)<br>100. Tobacco (mesh) (exp) (include all subheadings)<br>101. Cigarette$<br>102. Pipe$<br>103. Cigar$<br>104. Nicotine (mesh) (exp) (include all subheadings)<br>105. Water pipe<br>106. Hookah<br>107. Shisha<br>108. Paid work<br>109. Employment (mesh) (exp) (include all subheadings)<br>110. Work$ disability<br>111. Productivity<br>112. Employability<br>113. Work$ ability<br>114. Absenteeism (mesh) (exp) (include all subheadings)<br>115. Sick leave (mesh) (exp) (include all subheadings)<br>116. Presenteeism (mesh) (exp) (include all subheadings)<br>117. Sick$ absence<br>118. Work instability<br>119. Return to work (mesh) (exp) (include all subheadings)<br>120. Economic consequences<br>121. Occupational health<br>122. Labo$r
Systematic review terms	123. Systematic adj5 review<br>124. Narrative review<br>125. Meta-analysis (mesh) (exp)<br>126. Meta analysis<br>127. Meta adj5 analysis<br>128. Meta-synthesis<br>129. Meta synthesis<br>130. Meta adj5 synthesis<br>131. Literature review<br>132. Literature search<br>133. Meta-narrative review<br>134. Meta narrative review
Combining terms	135. RA – 1 OR 2 OR 3 OR 4 OR 5 OR 6<br>136. OA – 7 OR 8 OR 9 OR 10 OR 11 OR 12 OR 13<br>137. PSA – 14 OR 15 OR 16 OR 17 OR 18 OR 19 OR 20 OR 21 OR 22<br>138. AS – 23 OR 24 OR 25 OR 26 OR 27 OR 28 OR 29 OR 30<br>139. SLE – 31 OR 32 OR 33 OR 34 OR 35 OR 36 OR 37 OR 38<br>140. SSc – 39 OR 40 OR 41 OR 42 OR 43<br>141. Gout – 44 OR 45 OR 46 OR 47 OR 48 OR 49 OR 50 OR 51 OR 52 OR 53 OR 54 OR 55 OR 56 OR 57<br>142. Diseases – 136 OR 137 OR 138 OR 139 OR 140 OR 141 OR 142<br>143. Diet – 58 OR 59 OR 60 OR 61 OR 62 OR 63 OR 64 OR 65 OR 66 OR 67 OR 68 OR 69 OR 70 OR 71 OR 72 OR 73 OR 74<br>144. Exercise – 75 OR 76 OR 77 OR 78 OR 79 OR 80 OR 81 OR 82 OR 83 OR 84 OR 85 OR 86 OR 87 OR 88

	145. Weight – 89 OR 90 OR 91 OR 92 OR 93 OR 94 OR 95 OR 96 OR 97<br>146. Smoking - 98 OR 99 OR 100 OR 101 OR 102 OR 103 OR 104 OR 105 OR 106 OR 107<br>147. Work – 108 OR 109 OR 110 OR 111 OR 112 OR 113 OR 114 OR 115 OR 116 OR 117 OR 118 OR 119 OR 120 OR 121 OR 122<br>148. Exposures – 144 OR 145 OR 146 OR 147 OR 148<br>149. Systematic review terms - 123 OR 124 OR 125 OR 126 OR 127 OR 128 OR 129 OR 130 OR 131 OR 132 OR 133 OR 134 OR 135<br>150. 143 AND 149 AND 150

Supplementary table 2 – search strategy to identify published systematic reviews and meta-analyses on alcohol

The results from the first review of published systematic reviews and meta-analyses (supplementary table 1) were presented at a teleconference in January 2019. At this teleconference, it was decided to add alcohol as an exposure of interest for this taskforce. This led to a second systematic review of published reviews and meta-analyses. For completeness, the search strategy for this review is below. The results from this review are not reported in this systematic review on diet; they are published in a separate review on smoking and alcohol. However, these studies are included in the flow chart of figure 1, hence the inclusion of the search strategy here.

Category	Term
	1. Arthritis, Rheumatoid (mesh) (exp) (include all subheadings)<br>2. Inflammatory $arthritis<br>3. Undifferentiated arthritis<br>4. RA<br>5. Atrophic arthritis<br>6. Proliferative arthritis<br>7. Osteoarth$<br>8. Arthrosis<br>9. Degenerative joint disease<br>10. Hypertrophic arthritis<br>11. Arthropathy<br>12. Polyarthritis<br>13. OA<br>14. Arthritis psoriatica<br>15. Arthropathic psoriasis<br>16. Psoriatic arthropathy<br>17. Arthritis, Psoriatic (mesh) (exp) (include all subheadings)<br>18. Psoria$ arthriti$ [have to uncheck “map team to subject heading”]<br>19. Psoria$ arthropath$ [have to uncheck “map team to subject heading”]<br>20. Undifferentiated oligoarthritis<br>21. Arthritic psoriasis<br>22. PsA<br>23. Ankylosing spondylitis (mesh) (exp) (include all subheadings)<br>24. Ankylosi$<br>25. Spondyloarthr$ [have to uncheck “map team to subject heading”]<br>26. Spondylarthr$ [have to uncheck “map team to subject heading”]<br>27. Spondylitis (mesh) (exp) (include all subheadings)<br>28. Bechtere$ [have to uncheck “map team to subject heading”]<br>29. Marie-Strumpell<br>30. Spinal arthritis<br>31. Lupus erythematosus, systemic (mesh) (exp) (include all subheadings)<br>32. systemic lupus erythematosus<br>33. SLE<br>34. Libman-Sacks disease<br>35. Libman Sacks disease<br>36. Lupus erythematosus disseminatus<br>37. Disseminated lupus erythematosus

	38. Lupus syndrome<br>39. Sclerosis, Systemic (mesh) (exp) (include all subheadings)<br>40. SSc<br>41. Thibierge-Weissenbach syndrome<br>42. Morphea<br>43. Gout (mesh) (exp) (include all subheadings)<br>44. Gout$<br>45. Podagra<br>46. Tophus<br>47. Tophi<br>48. Tophaceous<br>49. Urate<br>50. Uric acid<br>51. Hyperurecemi$ [have to uncheck “map team to subject heading”]<br>52. Hyperurecaemi$ [have to uncheck “map team to subject heading”]<br>53. Hyperuricemia$<br>54. Hyperuricaemi$ [have to uncheck “map team to subject heading”]<br>55. arthritis urica<br>56. Gout acute
Exposure	57. Alcohol<br>58. Ethanol<br>59. Beer<br>60. Wine<br>61. Spirit$<br>62. liquor
Systematic review terms	63. Systematic adj5 review<br>64. Narrative review<br>65. Meta-analysis (mesh) (exp)<br>66. Meta analysis<br>67. Meta adj5 analysis<br>68. Meta-synthesis<br>69. Meta synthesis<br>70. Meta adj5 synthesis<br>71. Literature review<br>72. Literature search<br>73. Meta-narrative review<br>74. Meta narrative review
Combining terms	75. RA – 1 OR 2 OR 3 OR 4 OR 5 OR 6<br>76. OA – 7 OR 8 OR 9 OR 10 OR 11 OR 12 OR 13<br>77. PSA – 14 OR 15 OR 16 OR 17 OR 18 OR 19 OR 20 OR 21 OR 22<br>78. AS – 23 OR 24 OR 25 OR 26 OR 27 OR 28 OR 29 OR 30<br>79. SLE – 31 OR 32 OR 33 OR 34 OR 35 OR 36 OR 37 OR 38<br>80. SSc – 39 OR 40 OR 41 OR 42<br>81. Gout – 43 OR 44 OR 45 OR 46 OR 47 OR 48 OR 49 OR 50 OR 51 OR 52 OR 53 OR 54 OR 55 OR 56<br>82. Alcohol – 57 OR 58 OR 59 OR 60 OR 61 OR 62<br>83. Systematic review terms - 63 OR 64 OR 65 OR 66 OR 67 OR 68 OR 69 OR 70 OR 71 OR 72 OR 73 OR 74<br>84. Disease – 75 OR 76 OR 77 OR 78 OR 79 OR 80 OR 81<br>85. 82 AND 83 AND 84

Supplementary table 3 – Search terms for diet review

Category	Term
Disease terms	1. Arthritis, Rheumatoid (mesh) (exp) (include all subheadings)<br>2. Inflammatory $arthritis<br>3. Undifferentiated arthritis<br>4. RA<br>5. Atrophic arthritis<br>6. Proliferative arthritis<br>7. Osteoarth$<br>8. Arthrosis<br>9. Degenerative joint disease<br>10. Hypertrophic arthritis<br>11. Arthropathy<br>12. Polyarthritis<br>13. OA<br>14. Arthritis psoriatica<br>15. Arthropathic psoriasis<br>16. Psoriatic arthropathy<br>17. Arthritis, Psoriatic (mesh) (exp) (include all subheadings)<br>18. Psoria$ arthriti$ [have to uncheck “map team to subject heading”]<br>19. Psoria$ arthropath$ [have to uncheck “map team to subject heading”]<br>20. Undifferentiated oligoarthritis<br>21. Arthritic psoriasis<br>22. PsA<br>23. Ankylosing spondylitis (mesh) (exp) (include all subheadings)<br>24. Ankylosi$<br>25. Spondyloarthr$ [have to uncheck “map team to subject heading”]<br>26. Spondylarthr$ [have to uncheck “map team to subject heading”]<br>27. Spondylitis (mesh) (exp) (include all subheadings)<br>28. Bechtere$ [have to uncheck “map team to subject heading”]<br>29. Marie-Strumpell<br>30. Spinal arthritis<br>31. Lupus erythematosus, systemic (mesh) (exp) (include all subheadings)<br>32. systemic lupus erythematosus<br>33. SLE<br>34. Libman-Sacks disease<br>35. Libman Sacks disease<br>36. Lupus erythematosus disseminatus<br>37. Disseminated lupus erythematosus<br>38. Lupus syndrome<br>39. Sclerosis, Systemic (mesh) (exp) (include all subheadings)<br>40. SSc<br>41. Thibierge-Weissenbach syndrome<br>42. Morphea<br>43. Gout (mesh) (exp) (include all subheadings)<br>44. Gout$<br>45. Podagra<br>46. Tophus<br>47. Tophi<br>48. Tophaceous<br>49. Urate

	50. Uric acid<br>51. Hyperurecemi$ [have to uncheck “map team to subject heading”]<br>52. Hyperurecaemi$ [have to uncheck “map team to subject heading”]<br>53. Hyperuricemia$<br>54. Hyperuricaemi$ [have to uncheck “map team to subject heading”]<br>55. arthritis urica<br>56. Gout acute<br>57. Inflammatory joint disease
Diet exposures	58. Diet (mesh) (exp) (include all subheadings)<br>59. Nutrition<br>60. Food (mesh) (exp) (include all subheadings)<br>61. Food habit$<br>62. Nutritional status (mesh) (exp) (include all subheadings)<br>63. Vitamin$ (mesh) (exp) (include all subheadings)<br>64. Antioxidant$ (mesh) (exp) (include all subheadings)<br>65. Fatty acid$ (mesh) (exp) (include all subheadings)<br>66. Carbohydrate$ (mesh) (exp) (include all subheadings)<br>67. Diet$ protein<br>68. Calcium<br>69. Fish oil$ (mesh) (exp) (include all subheadings)<br>70. Fruit (mesh) (exp) (include all subheadings)<br>71. Vegetable$ (mesh) (exp) (include all subheadings)<br>72. Micronutrient$ (mesh) (exp) (include all subheadings)<br>73. Nutriment$<br>74. Neutraceutical$<br>75. Dietary supplement<br>76. Probiotic<br>77. Prebiotic<br>78. Functional food
Exclusions	79. Cross-sectional<br>80. Cross sectional<br>81. Children<br>82. Child<br>83. Juvenile<br>84. Adolescent<br>85. Teenager<br>86. Animal<br>87. Rat<br>88. Mouse<br>89. Case study<br>90. Case series<br>91. Systematic adj5 review<br>92. Narrative review<br>93. Meta-analysis (mesh) (exp)<br>94. Meta analysis<br>95. Meta adj5 analysis<br>96. Meta-synthesis<br>97. Meta synthesis<br>98. Meta adj5 synthesis<br>99. Literature review<br>100. Literature search

	101. Meta-narrative review<br>102. Meta narrative review<br>103. Septic arthritis
OA exclusions	104. Vitamin E<br>105. Bromelain<br>106. Glucosamine<br>107. Willow bark extract<br>108. Chondroitin<br>109. Artemisia annua extract<br>110. Green lipped muscle extract<br>111. Diacerin<br>112. Methylsulfonylmethane<br>113. Avocado adj3 unsaponifiables<br>114. Soybean adj3 unsaponifiables<br>115. Undenatured type II collagen<br>116. Undenatured type 2 collagen<br>117. L-carnitine<br>118. Curcumin<br>119. Pycnogenol<br>120. Boswellia serrata<br>121. Cucuma longa<br>122. Passion fruit<br>123. Collagen hydrolysate
RA exclusions	124. Marine oil<br>125. Omega-3<br>126. Omega 3<br>127. Probiotics<br>128. Vitamin D
Combining terms	129. RA – 1 OR 2 OR 3 OR 4 OR 5 OR 6<br>130. RA exclusions – 124 OR 125 OR 126 OR 127 OR 128<br>131. RA minus exclusions – 129 NOT 130<br>132. OA – 7 OR 8 OR 9 OR 10 OR 11 OR 12 OR 13<br>133. OA exclusions –104 OR 105 OR 106 OR 107 OR 108 OR 109 OR 110 OR 111 OR 112 OR 113 OR 114 OR 115 OR 116 OR 117 OR 118 OR 119 OR 120 OR 121 OR 122 OR 123<br>134. OA minus exclusions – 132 NOT 133<br>135. PSA – 14 OR 15 OR 16 OR 17 OR 18 OR 19 OR 20 OR 21 OR 22<br>136. AS – 23 OR 24 OR 25 OR 26 OR 27 OR 28 OR 29 OR 30<br>137. SLE – 31 OR 32 OR 33 OR 34 OR 35 OR 36 OR 37 OR 38<br>138. SSc – 39 OR 40 OR 41 OR 42<br>139. Gout – 43 OR 44 OR 45 OR 46 OR 47 OR 48 OR 49 OR 50 OR 51 OR 52 OR 53 OR 54 OR 55 OR 56<br>140. Diseases – 128 OR 131 OR 132 OR 133 OR 134 OR 135 OR 136 OR 57<br>141. Diet – 58 OR 59 OR 60 OR 61 OR 62 OR 63 OR 64 OR 65 OR 66 OR 67 OR 68 OR 69 OR 70 OR 71 OR 72 OR 73 OR 74 OR 75 OR 76 OR 77 OR 78<br>142. Exclusions – 79 OR 80 OR 81 OR 82 OR 83 OR 84 OR 85 OR 86 OR 87 OR 88 OR 89 OR 90 OR 91 OR 92 OR 93 OR 94 OR 95 OR 96 OR 97 OR 98 OR 99 OR 100 OR 101 OR 102 OR 103<br>143. 140 AND 141<br>144. 143 NOT 142

Supplementary table 4 – Included outcomes and examples of measures used to assess these outcomes

- Disease activity
  - OA
    - Western Ontario and McMaster Universities Arthritis Index [WOMAC]
  - RA
    - Acute phase reactants (i.e. C-reactive protein and erythrocyte sedimentation rate)
    - Swollen joint count
    - Tender joint count
    - Physician global assessment of disease activity (VAS)
    - Patient global health (VAS)
    - Disease activity composite measures (eg. Disease Activity Score [DAS28, DAS44], Rheumatoid arthritis Impact of Disease Score [RAID])
  - PsA [1]
    - Acute phase reactants (i.e. C-reactive protein and erythrocyte sedimentation rate)
    - Swollen joint count
    - Tender joint count
    - Physician global assessment of disease activity (VAS)
    - Patient global assessment of disease activity (VAS)
    - Dactylitis (e.g. Leeds dactylitis index)
    - Enthesitis (e.g. Mander/Newcastle Enthesitis Index, Leeds Enthesitis index)
    - Extent of psoriasis (e.g. Psoriasis Area and Severity Index [PASI])
    - Nail involvement (e.g. Nail Psoriasis Severity Index)
    - Disease activity composite measures (e.g. Composite Psoriatic Disease Activity Index [CPDAI], Disease Activity in Psoriatic Arthritis [DAPSA], clinical Disease Activity in Psoriatic Arthritis [cDAPSA], PsA Impact of Disease Score [PsAID] Psoriatic Arthritis Disease Activity Score [PASDAS])
  - AS [2]
    - Acute phase reactants (i.e. C-reactive protein and erythrocyte sedimentation rate)
    - Swollen joint count
    - Tender joint count
    - Disease activity composite measures (e.g. Ankylosing Spondylitis Disease Activity Score [ASDAS], Bath Ankylosing Spondylitis Disease Activity Index [BASDAI], Disease Activity Score [DAS44])
    - Enthesitis
    - Spinal mobility (e.g. Bath Ankylosing Spondylitis Metrology Index [BASMI])
    - Stiffness
  - SLE [3]
    - Disease activity composite measures (e.g. British Isles Lupus Assessment Group measure [BILAG], Systemic Lupus Erythematosus Disease Activity Index [SLEDAI])
    - Organ damage measures (e.g. Systemic Lupus International Collaborating Clinics (SLICC)/American College of Rheumatology Damage Index [SDI])
  - SSc [4]
    - Skin (e.g. Modified Rodnan skin score, visual analogue scale [VAS]/likert scale, Durometer reading)
    - Musculoskeletal (e.g. tender joint count, tender friction rubs assessed by doctor, serum creatinine)

- Cardiac / pulmonary / renal / gastrointestinal involvement
- Raynaud's phenomenon (e.g. Raynaud condition score, VAS raynauds)
- Digital ulcers (e.g. activity digital tip ulcer count on volar surface, VAS digital ulcer)
- Acute phase reactants (i.e. C-reactive protein and erythrocyte sedimentation rate)

  - Gout [5]
    - Serum urate
    - Gout flare recurrence
    - Tophus regression [6] / tophi number
    - Joint inflammation / tenderness score
- Physical functioning
  - OA
    - Physical function (e.g. the Knee Injury and Osteoarthritis Outcome Score [KOOS], Veterans Short Form 12 Health Survey [VR-12], Hip disability and Osteoarthritis Outcome Score [HOOS], WOMAC).
    - Objective measures (e.g. gait speed, grip strength)
    - Range of motion of effected joint
  - RA
    - Physical function (e.g. the Health Assessment Questionnaire [HAQ], Arthritis Impact Measurement Scale [AIMS], SF36-physical function)
    - Objective measures (e.g. gait speed, grip strength)
  - PsA
    - Physical function (e.g. the HAQ, Arthritis Impact Measurement Scale [AIMS], SF36-physical function)
    - Objective measures (e.g. gait speed, grip strength)
  - AS
    - Physical function (e.g. Health Assessment Questionnaire for the Spondylarthropathies [HAQ-S], Dougados Functional Index [DFI], Bath Ankylosing Spondyltitis Functional Index [BASFI])
    - Objective measures (e.g. gait speed, grip strength)
  - SLE [7]
    - Physical function (e.g. the HAQ, SF-36 physical function, Valued Life Activities Disability Scale)
    - Objective measures (e.g. gait speed, grip strength)
  - SSc
    - Physical function (e.g. the HAQ, SF-36).
    - Objective measures (e.g. gait speed, grip strength)
  - Gout
    - Physical function (e.g. HAQ [5;8], SF-36)
    - Objective measures (e.g. gait speed, grip strength)
- Pain
  - OA [9]
    - OARSI-OMERACT Initiative: New OA Pain Measure
    - Dallas Pain Questionnaire
    - Neck Pain and Disability Scale [NPAD]
    - WOMAC
    - Australian/Canadian Hand OA Index (AUSCAN)
  - RA
    - Patient pain rating (e.g. visual analogue scale)
  - PSA

        - Patient pain rating (e.g. visual analogue scale)
    - AS
        - Patient pain rating (e.g. visual analogue scale)
    - SLE
        - Patient pain rating (e.g. visual analogue scale)
    - SSc
        - Patient pain rating (e.g. visual analogue scale)
    - Gout
        - Patient pain rating (e.g. visual analogue scale / likert scale) [10]
- Fatigue
    - OA
        - Patient fatigue rating (e.g. visual analogue scale, other disease specific measure)
        - Generic fatigue questionnaire (e.g. Chalder Fatigue Scale)
    - RA
        - Patient fatigue rating (e.g. visual analogue scale, other disease specific measure)
        - Generic fatigue questionnaire (e.g. Chalder Fatigue Scale)
        - Bristol Rheumatoid Arthritis Fatigue – multidimensional questionnaire (BRAF-MDQ)
    - PSA
        - Patient fatigue rating (e.g. visual analogue scale, other disease specific measure)
        - Generic fatigue questionnaire (e.g. Chalder Fatigue Scale)
    - AS
        - Patient fatigue rating (e.g. visual analogue scale, other disease specific measure)
        - Generic fatigue questionnaire (e.g. Chalder Fatigue Scale)
    - SLE
        - Patient fatigue rating (e.g. visual analogue scale, other disease specific measure)
        - Generic fatigue questionnaire (e.g. Chalder Fatigue Scale)
    - SSc
        - Patient fatigue rating (e.g. visual analogue scale, other disease specific measure)
        - Generic fatigue questionnaire (e.g. Chalder Fatigue Scale)
    - Gout
        - Patient fatigue rating (e.g. visual analogue scale, other disease specific measure)
        - Generic fatigue questionnaire (e.g. Chalder Fatigue Scale)
- Erosions
    - Joint damage by X-ray (e.g. Sharp method, Larsen method, Lane Index, Wilke Index , Kellgren-Lawrence hand OA radiological index [9])
- Physical comorbidity
    - Major comorbidity
        - MACE (major adverse cardiac event)
        - Lung disease
        - Peptic ulcer disease
        - Liver disease
        - Renal disease
        - Tuberculosis / other serious infections

- Diabetes
- Hyperthyroidism
- Depression
- Cancer
- Fractures
- High cholesterol / dyslipidaemia

- Mental health
  - Mental health assessment questionnaires (e.g. Hospital Anxiety and Depression Scale (HADS), the AIMS, Mini-mental state examination)
- Quality of life (e.g. EQ-5D, SF-36)
  - Disease specific quality of life measures (e.g. RaQOL [11], ASQOL [12], PsAQoL [13])
- Work status
  - Categorical rating of work status (e.g. at work, retired, sick leave)
  - Number of days absent from work in a given time window

Supplementary table 5 – Description of reviews of animal products in OA

Table – Animal products, description of reviews

Authors (date)	Review type	Study type included	Type of OA	Exposure detail	Number of studies included	Funders
Liu (2018) [14]	MA	RCTs	Hip, knee or hand	Collagen hydrolysate<br>Undenatured type II collagen<br>Green lipped mussel extract	2<br>1<br>1	**Government** (NHMRC program grant, Department of education grant), **Industry** (PuraPharm postgrad scholarship), author disclosures (Flexion, Nestle, Merck)
Senftleber (2017) [15]	MA	RCTs	Knee or hip	Marine oil supplements	6	**Charity** (Oak Foundation [indirectly funded]), **Government** (National Institute of Arthritis and Musculoskeletal and Skin Diseases, NIH [individual fellowship of an author])

MA = meta-analysis, NHMRC = National Health and Medical Research Council, NIH = National Institutes of Health, OA = osteoarthritis, RCT = randomised controlled trial

Supplementary table 6 – Description of studies of animal products in OA

Table – Animal products (OA), description of included studies

Author (date) [country]	Study design	Type of OA	Inclusion criteria	Exposure detail	N	Age years, mean (SD)	N (%) female	Funders
Azidah (2017) [Malaysia] [16]	RCT	Knee	ACR OA criteria, radiological grade I-II, symptoms ≥3 months<br>Exclusions: secondary knee OA, disability comorbidity (e.g. renal / liver disease, neoplasm, other rheumatic disease), severe knee pain, willingness to have surgery, history of joint lavage, arthroscopy, hyaluronic acid treatment in previous 6 months, intraarticular steroids in last 3 months, allergy to channa striatus	1) 1000mg/day channa striatus<br>2) 500mg/day channa striatus<br>p) corn starch placebo	1) 39<br>2) 38<br>p) 39	1) 52.0 (5.8)<br>2) 52.9 (6.7)<br>p) 52.8 (7.0)	1) 28 (70%)<br>2) 23 (57.5%)<br>p) 34 (85.0%)	**University** (Universiti Sains Malaysia Research University)
Hill (2016) [Australia] [17]	RCT	Knee	Aged >40 years, ACR criteria for OA, VAS pain >20mm<br>Exclusions: severe radiographic OA (Grade 3 – OARSI Atlas20), dementia or inability to give informed consent, pregnancy or lactation, planned knee replacement, high dose fish oil use for ≥6 months, contraindications to MRI	1) High dose fish oil containing 4.5g EPA+DHA per day<br>2) Low dose fish oil containing 0.45g EPA+DHA per day	1) 101<br>2) 101	1) 60.8 (10.4)<br>2) 61.1 (9.6)	1) 59 (58.4)<br>2) 40 (39.6)	**Government** (National Health and Medical Research Council of Australia), **Charity** (Arthritis Australia)
Chen (2016) [Australia] [18]	RCT	Knee	Aged >40 years, ACR criteria for OA, VAS pain >20mm<br>Exclusions: severe radiographic OA (Grade 3 – OARSI Atlas20), dementia or inability to give informed consent, pregnancy or lactation, planned knee replacement, high dose fish oil use for ≥6 months, contraindications to MRI	1) High dose fish oil containing 4.5g EPA+DHA per day<br>2) Low dose fish oil containing 0.45g EPA+DHA per day	1) 101<br>2) 101	1) 60.8 (10.4)<br>2) 61.1 (9.6)	1) 59 (58.4)<br>2) 40 (39.6)	**Government** (National Health and Medical Research Council of Australia), **Charity** (Arthritis Australia)
Kumar (2015) [India] [19]	RCT	Knee	Age 30-65 years, KL grade 2-4, VAS ≥40 [type of VAS undefined]	1) Pork, 2) Beef, p1) placebo, p2) placebo 5g skin dissolved in 250ml water or milk in morning and night after food	1) 19<br>2) 18<br>p1) 11<br>p2) 10	not reported	1) 17 (89.4)<br>2) 11 (57.9)<br>p1) 10 (90.9)<br>p2) 7 (63.6)	Not reported
Schauss (2012) [USA] [20]	RCT	Knee or hip	Age: 40-70, Pain VAS (0-10) ≥4 for ≥3 months.<br>Exclusions: serious/chronic medical conditions, pregnancy, RA / inflammatory arthritis, NSAID therapy / alternative therapy for OA for past 15 days	1) Capsules of hydrolysed chicken sternal cartilage extract composed of hydrolysed collagen type II<br>p) Capsules of cellulose	1) 35<br>p) 33	1) 54.3 (8.69)<br>p) 54.5 (9.79)	1) 23 (65.7)<br>p) 18 (54.5)	**Industry** (BioCell technology)

ACR = American College of Rheumatology, DHA = docosahexaenoic acid, EPA = eicosapentaenoic acid, KL = Kellgren Lawrence, N = number, NSAID = non-steroidal anti-inflammatory drug, OA = osteoarthritis, P = placebo, RA = rheumatoid arthritis, RCT = randomised controlled trial, SD = standard deviation, USA = United States of America, VAS = visual analogue scale

Table – Animal products (OA) cont., description of included studies

Author (date) [country]	Study design	Type of OA	Inclusion criteria	Exposure detail	N	Age years, mean (SD)	N (%) female	Funders
Nagaoka (2010) [Japan] [21]	RCT	Knee	Aged 40-85 years, KL grade 0-3<br>Exclusion: any inflammatory bone/cartilage condition, previous knee surgery, known allergy to chicken, participant in clinical trial, women who are pregnant, nursing or of child bearing potential, treatment with intra-articular hyaluronic acid, steroids within 3 weeks, use of health foods, presence of significant clinical condition	1) Capsules of chicken comb extract<br>p) Placebo pills containing cellulose	1) 9<br>p) 12	1) 62.4 (12.5)<br>p) 63.3 (9.5)	1) 17 (81.0)<br>P) 18 (81.8)	Not reported
Ruff (2009) [USA] [22]	RCT	Knee	Age >18 years, symptoms of OA, ACR functional grade I-III, persistent knee pain.<br>Exclusions: receiving remission inducing drugs in past 4 months, other serious conditions, body weight >113.5kg, allergy to eggs, pregnant women	1) Capsules containing egg shell membrane<br>p) Capsules contacting vegetarian placebo	1) 29<br>p) 31	not reported	not reported	**Industry** (ESM Technologies)
Kalman (2008) [USA] [23]	RCT	Knee	Aged >40 years, KL grade >2, pain for at least 15 of previous 20 days<br>Exclusion: chicken/corn/potato/rice/cellulose allergy, inflammatory arthritis, MS or autoimmune disorder, oral steroids in past 4 weeks, intraarticular steroids in past 3 months, joint injury, other serious condition, pregnancy, renal dysfunction	1) Capsule of chicken comb extract<br>p) matched placebo capsules	1) 11<br>p) 9	1) 57.7 (10.1)<br>p) 54.6 (7.7)	1) 7 (63.6)<br>p) 4 (44.4)	**Industry** (Biobérica)
Hesslink (2002) [India] [24]	RCT	Knee	ACR OA criteria	1) Capsules containing standard fish oil blend rich in Omega-3<br>p) Identical capsules of soy lecithin	1) 33<br>p) 31	1) 58.1 (6.3)<br>p) 55.5 (6.8)	1) 11 (33.3)<br>p) 14 (45.2)	**Industry** (Imagenetix, Inc.)
Stammers (1992) [UK] [25]	RCT	Not reported	Aged 49-87 years, NSAIDS for at least 2 weeks	1) 10ml cod liver oil – 786 mg of EPA<br>p) 10ml olive oil	1) 44<br>p) 42	1) 67<br>p) 69	1) 29 (65.9)<br>p) 33 (78.6)	**Industry** (Seven Seas Ltd)
Kilinc (2018) [Turkey][26]	Single arm int.	Knee	Bilateral knee pain ≥4cm on VAS, analgesic and anti-inflammatory medication discontinued 3 weeks before start of treatment, KL grade II-III	720mg promerim for 15 days, then 360mg promerim for next 15 days. Patients also received exercise program.	92	51.5 (7.1)	69 (75)	No funding
Lu (2014) [USA] [27]	Pros. Cohort	Knee	Age 45-79, OA initiative<br>Exclusion: baseline KL grade = 4, primary lateral joint space narrowing, difference of rim distance from tibial plateau to tibial rim closest to femoral condyle between baseline and any follow ≥2 mm	Mean glasses of milk per week, coded as: none, ≤3, 4-6, ≥7	2148	62.4 (9.0	1260 (58.7%)	**Government** (National Heart, Lung and Blood Institute, NIH), **Industry** (OAI: Pfizer, Novartis, Merck, GSK)

ACR = American College of Rheumatology, EPA = eicosapentaenoic acid, GSK = GlaxoSmithKline, KL = Kellgren Lawrence, N = number, NSAID = non-steroidal anti-inflammatory drug, OA = osteoarthritis, P = placebo, RA = rheumatoid arthritis, RCT = randomised controlled trial, SD = standard deviation, USA = United States of America, VAS = visual analogue scale

Supplementary table 7 – Collagen and OA progression, results

Table – Collagen, results and quality assessment

Outcome (outcome measure)	Study (date) [study type]	Standardised result, SMD (95% CI) unless otherwise stated	Natural result	AMSTAR2 quality	Rand. Seq.	Alloc. Conc.	Blind. Part.	Blind. Asses.
Pain	Liu (2018) [MA] [14]	Undenatured type II collagen<br>Short term: pooled SMD -0.67 (-1.01, -0.33);<br>Collagen hydrolysate<br>Medium term: pooled SMD -0.28 (-0.54, -0.02);		Moderate				
	Kumar (2015) [RCT] [19]	Pork collagen vs pork placebo at 91 days<br>SMD -1.79 (-2.67, -0.91)<br>Beef collagen vs beef placebo at 91 days<br>SMD -1.83 (-2.75, -0.91)	Pain VAS, Baseline / 91 days, mean (SD)<br>Pork collagen: 63.2 (10.6) / 31.1 (15.2)<br>Beef collagen: 66.0 (12.3) / 28.0 (10.9)<br>Pork placebo: 60.0 (6.3) / 57.3 (13.5)<br>Beef placebo: 62.0 (14.0) / 55.0 (20.1)		L	H/UC	H/UC	H/UC
	Schauss (2012) [RCT] [20]	Collagen vs placebo at 70 days<br>SMD -0.44 (-0.93, 0.04)	WOMAC pain, Baseline / 70 days, mean (SD)<br>Collagen: 9.88 (2.93) / 6.13 (2.66)<br>Placebo: 10.53 (2.71) / 7.48 (3.40)		L	L	L	L
	Nagaoka (2010) [RCT] [21]	Collagen vs placebo at 16 weeks<br>SMD -0.57 (-1.45, 0.31)	Pain VAS, BL / 16 weeks, mean (SD)<br>Collagen: 55.4 (8.6) / 12.6 (6.3)<br>Placebo: 54.7 (8.5) / 22.2 (21.5)		H/UC	H/UC	H/UC	H/UC
	Kalman (2008) [RCT] [23]	Collagen vs placebo at 8 weeks<br>SMD -0.03 (-0.91, 0.85)	WOMAC pain, Baseline / 8 weeks, mean (SD)<br>Collagen: 10.4 (3.6) / 6.3 (4.0)<br>Placebo: 10.4 (2.7) / 6.4 (2.7)		L	H/UC	L	H/UC
	Bespoke MA of:<br>Kumar 2015 [beef]<br>Kumar 2015 [Pork]<br>Schauss 2012<br>Nagaoka 2010<br>Kalman 2008	Collagen vs placebo<br>Meta-SMD -0.78 (-1.10, -0.45)<br>I^2 = 74.0%						

Alloc. Conc. = allocation concealment, AMSTAR2 = Assessing the Methodological Quality of Systematic Reviews, BL = baseline, Blind. Asses. = Blinded assessors, Blind. Part. = blinded participants, CI = confidence interval, H/UC = high / unclear risk of bias, L = low risk of bias, MA = meta-analysis, OA = osteoarthritis, Rand. Seq. = random sequence generation, RCT = randomised controlled trial, SD = standard deviation, SMD = Standardised mean difference, VAS = visual analogue scale, WOMAC = Western Ontario and McMaster Universities Osteoarthritis Index

Table (cont.) – Collagen, results and quality assessment

Outcome (outcome measure)	Study (date) [study type]	Standardised result, SMD (95% CI) unless otherwise stated	Natural result	AMSTAR2 quality	Rand. Seq.	Alloc. Conc.	Blind. Part.	Blind. Asses.
Function	Liu (2018) [MA] [14]	Undenatured type II collagen<br>Short term: pooled SMD -0.55 (-0.94, -0.17);<br>Collagen hydrolysate<br>Short term: pooled SMD 0.11 (-0.57, 0.78)		Moderate				
	Kumar (2015) [RCT] [19]	Pork collagen vs pork placebo at 91 days<br>SMD -1.49 (-2.33, -0.65)<br>Beef collagen vs beef placebo at 91 days<br>SMD -1.47 (-2.34, -0.60)	WOMAC function, Baseline / 91 days, mean (SD)<br>Pork collagen: 47.2 (9.8) / 31.1 (9.8)<br>Beef collagen: 50.3 (9.6) / 25.8 (11.3)<br>Pork placebo: 47.3 (8.6) / 45.5 (9.4)<br>Beef placebo: 50.1 (14.7) / 47.3 (19.4)		L	H/UC	H/UC	H/UC
	Schauss (2012) [RCT] [20]	Collagen vs placebo at 70 days<br>SMD -0.67 (-1.16, -0.18)	WOMAC function, Baseline / 70 days, mean (SD)<br>Collagen: 40.35 (8.51) / 26.65 (8.62)<br>Placebo: 39.20 (8.75) / 32.90 (10.03)		L	L	L	L
	Kalman (2008) [RCT] [23]	Collagen vs placebo at 8 weeks<br>SMD -0.32 (-1.20, 0.57)	WOMAC function, Baseline / 8 weeks, mean (SD)<br>Collagen: 36.3 (7.7) / 23.1 (15.1)<br>Placebo 37.4 (10.6) / 27.3 (10.7)		L	H/UC	L	H/UC
	Bespoke MA of:<br>Kumar 2015 [beef]<br>Kumar 2015 [Pork]<br>Schauss 2012<br>Kalman 2008	Collagen vs placebo<br>Pooled SMD -0.89 (-1.24, -0.54)<br>I^2 = 47.7%						
Stiffness	Kalman (2008) [RCT] [23]	Collagen vs placebo at 8 weeks<br>SMD 0.00 (-0.88, 0.88)	WOMAC stiffness, Baseline / 8 weeks, mean (SD)<br>Collagen: 4.2 (1.0) / 2.9 (1.9)<br>Placebo 4.5 (1.9) / 2.9 (1.0)		L	H/UC	L	H/UC
QoL	Kumar (2015) [RCT] [19]	Pork collagen vs pork placebo at 91 days<br>SMD -1.57 (-2.42, -0.72)<br>Beef collagen vs beef placebo at 91 days<br>SMD -1.57 (-2.45, -0.69)	"QoL Score", Baseline / 91 days, mean (SD)<br>Pork collagen: 53.4 (10.4) / 34.3 (10.8)<br>Beef collagen: 56.9 (9.9) / 28.7 (11.4)<br>Pork placebo: 53.3 (8.8) / 51.2 (10.7)<br>Beef placebo: 56.3 (15.4) / 52.8 (20.9)		L	H/UC	H/UC	H/UC

Alloc. Conc. = allocation concealment, AMSTAR2 = Assessing the Methodological Quality of Systematic Reviews, Blind. Asses. = Blinded assessors, Blind. Part. = blinded participants, CI = confidence interval, H/UC = high / unclear risk of bias, L = low risk of bias, MA = meta-analysis, OA = osteoarthritis, QoL = quality of life, Rand. Seq. = random sequence generation, RCT = randomised controlled trial, SD = standard deviation, SMD = Standardised mean difference, VAS = visual analogue scale, WOMAC = Western Ontario and McMaster Universities Osteoarthritis Index

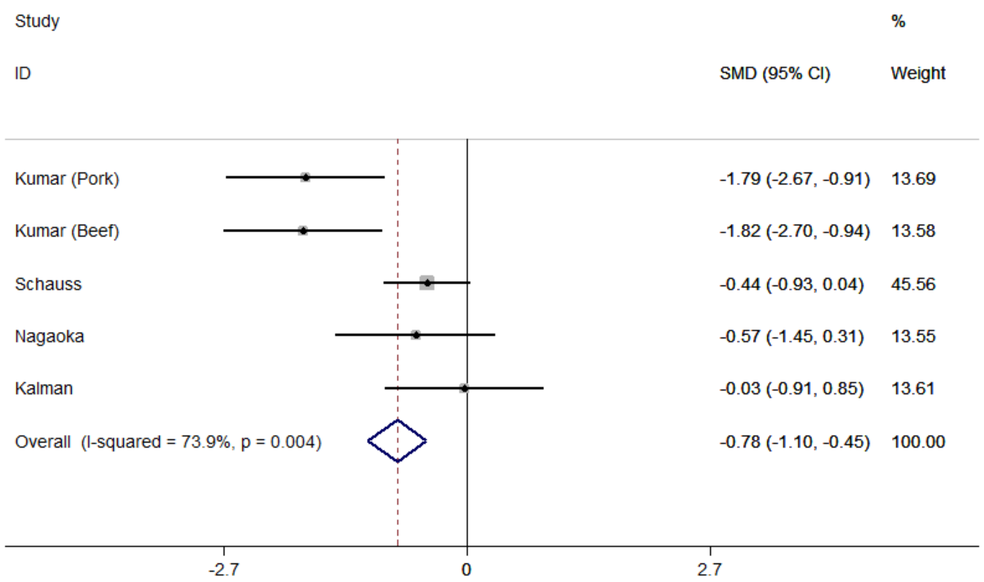

Supplementary figure 1 – Collagen, bespoke meta-analysis for pain (OA)

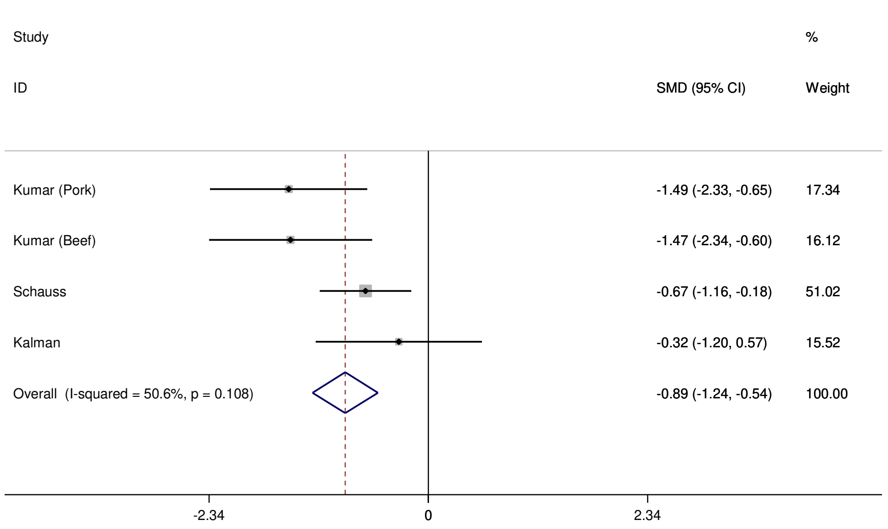

Supplementary Figure 2 – Collagen, bespoke meta-analysis for function (OA)

Supplementary table 8 – Milk and OA progression, results

Table – Collagen, results and quality assessment

Outcome (outcome measure)	Study (date) [study type]	Standardised result, SMD (95% CI) unless otherwise stated	Natural result	Study Pop.	Attr.	Prog. Meas.	Outc. Meas.	Conf.	Stats.
Joint space width	Lu (2014) [Pros. Obs.] [27]		Decrease in joint space width, HR (95% CI)<br>*Men*<br>none: ref<br><=3: 0.77 (0.53, 1.13)<br>4-6: 0.92 (0.60, 1.40)<br>>=7: 0.61 (0.39, 0.94); p=0.075<br>*Women*:<br>none: ref<br><=3: 0.67 (0.50, 0.91)<br>4-6: 0.71 (0.50, 1.00)<br>>=7: 0.56 (0.38, 0.81); p=0.008	L	L	M	L	L	L

Attr. = attrition, CI = confidence interval, Conf. = confounding, HR = hazard ratio, L = low risk of bias, M = moderate risk of bias, OA = osteoarthritis, Outc. Meas = outcome measurement, Prog. Meas. = prognostic factor measurement, Pros. Obs. = prospective observational, SMD = standardised mean difference, Stats. = statistical analysis, Study Pop. = study population

Supplementary table 9 – Egg shell membrane and OA progression, results

Table (cont.) – Egg-shell membrane, results and quality assessment

Outcome (outcome measure)	Study (date) [study type]	Standardised result, SMD (95% CI) unless otherwise stated	Natural result	AMSTAR2 quality	Rand. Seq.	Alloc. Conc.	Blind. Part.	Blind. Asses.
Pain	Ruff (2009) [RCT] [22]	Egg-shall vs placebo at 60 days<br>SMD -0.56 (-1.15, 0.04)	WOMAC pain, baseline / 60 days, mean (SD)<br>Egg-shell: 44.0 (16.8) / 37.5 (25.2)<br>Placebo: 50.6 (19.4) / 50.7 (22.2); p=0.038		L	L	L	L
Function	Ruff (2009) [RCT] [22]	Egg-shall vs placebo at 60 days<br>SMD -0.48 (-1.08, 0.11)	WOMAC function, baseline / 60 days, mean (SD)<br>Egg-shell: 48.1 (19.5) / 40.5 (27.1)<br>Placebo: 55.2 (21.3) / 53.1 (24.9); p=0.076		L	L	L	L
Stiffness	Ruff (2009) [RCT] [22]	Egg-shall vs placebo at 60 days<br>SMD -0.86 (-1.47, -0.24)	WOMAC stiffness, baseline / 60 days, mean (SD)<br>Egg-shell: 50.5 (20.3) / 35.0 (25.8)<br>Placebo: 59.3 (24.0) / 56.5 (24.3); p=0.005		L	L	L	L

Alloc. Conc. = allocation concealment, AMSTAR2 = Assessing the Methodological Quality of Systematic Reviews, Blind. Asses. = Blinded assessors, Blind. Part. = blinded participants, CI = confidence interval, H/UC = high / unclear risk of bias, L = low risk of bias, OA = osteoarthritis Rand. Seq. = random sequence generation, RCT = randomised controlled trial, SD = standard deviation, SMD = Standardised mean difference, WOMAC = Western Ontario and McMaster Universities Osteoarthritis Index

Supplementary table 10 – Channa Striatus extract and OA progression, results

Table – Channa Striatus (OA), results and quality assessment

Outcome	Study (date) [study type]	Standardised result, SMD (95% CI) unless otherwise stated	Natural result	AMSTAR2 quality	Rand. Seq.	Alloc. Conc.	Blind. Part.	Blind. Asses.
Pain	Azidah (2017) [RCT] [16]	1000mg Channa striatus vs placebo at 6 months<br>SMD -0.42 (-0.87, 0.03)<br>500mg Channa striatus vs placebo at 6 months<br>SMD -0.31 (-0.76, 0.14)	WOMAC pain, 6 months, mean (SD*)<br>Channa striatus 1000mg: 85.91 (96.94)<br>Channa striatus 500mg: 96.65 (98.11)<br>Placebo: 126.99 (96.86); p=0.139		L	L	L	L
Function	Azidah (2017) [RCT] [16]	1000mg Channa striatus vs placebo at 6 months<br>SMD -0.56 (-1.01, -0.11)<br>500mg Channa striatus vs placebo at 6 months<br>SMD -0.42 (-0.87, 0.03)	WOMAC function, 6 months, mean (SD*)<br>Channa striatus 1000mg: 312.91 (329.36)<br>Channa striatus 500mg: 358.15 (329.37)<br>Placebo: 496.48 (329.36)		L	L	L	L
Stiffness	Azidah (2017) [RCT] [16]	1000mg Channa striatus vs placebo at 6 months<br>SMD -0.51 (-0.96, -0.06)<br>500mg Channa striatus vs placebo at 6 months<br>SMD -0.53 (-0.98, -0.07)	WOMAC stiffness, 6 months, mean (SD*)<br>Channa striatus 1000mg: 35.12 (44.53)<br>Channa striatus 500mg: 34.25 (44.52)<br>Placebo: 57.76 (44.53); p=0.016		L	L	L	L

*calculated from 95% confidence interval reported in paper

Alloc. Conc. = allocation concealment, AMSTAR2 = Assessing the Methodological Quality of Systematic Reviews, BL = baseline, Blind. Asses. = Blinded assessors, Blind. Part. = blinded participants, CI = confidence interval, H/UC = high / unclear risk of bias, L = low risk of bias, Rand. Seq. = random sequence generation, RCT = randomised controlled trial, SD = standard deviation, SMD = Standardised mean difference, WOMAC = Western Ontario and McMaster Universities Osteoarthritis Index

Supplementary table 11 – Fish oil and OA progression, results

Table – Fish oil (OA), results and quality assessment

Outcome	Study (date) [study type]	Standardised result, SMD (95% CI) unless otherwise stated	Natural result	AMSTAR2 quality	Rand. Seq.	Alloc. Conc.	Blind. Part.	Blind. Asses.
Pain	Senftleber (2017) [MA] [15]	Fish Oil vs control<br>SMD -0.16 (-0.57, 0.24)		High				
	Hill (2016) [RCT] [17]		WOMAC pain, Mean difference (SE), high vs low dose<br>1 year: 2.3 (1.2) p = 0.06<br>2 years: 3.3 (1.3) p=0.009 [in favour of low dose]		L	L	L	L
	Hesslink (2002) [RCT] [24]	Fish oil vs placebo at 68 days<br>SMD -0.61 (-1.12, -0.11)	LI pain, BL / day 68, mean (SD*)<br>Fish oil: 6.0 (0.6) / 3.9 (1.7)<br>Placebo: 6.1 (1.1) / 5.1 (2.2)		H/UC	H/UC	L	L
	Stammers (1992) [RCT] [25]	Fish oil vs placebo, change from baseline to 6 months<br>SMD 0.21 (-0.21, 0.63)	VAS pain, change from bl to 6 months, mean (SD)<br>Fish oil: 1 (20)<br>Placebo: -3 (18)		H/UC	H/UC	H/UC	H/UC
Function	Senftleber (2017) [MA] [15]	Fish Oil vs control<br>SMD 0.11 (-0.13, 0.35)		High				
	Hill (2016) [RCT] [17]		WOMAC function, Mean difference (SE), high vs low dose<br>1 year: 6.5 (3.7) p = 0.08<br>2 years: 8.5 (4.0) p=0.032 [in favour of low dose]		L	L	L	L
	Hesslink (2002) [RCT] [24]	Fish oil vs placebo at 68 days<br>SMD -0.65 (-1.15, -0.14)	LI activities, BL / day 68, mean (SD*)<br>Fish oil: 4.6 (1.1) / 3.1 (1.7)<br>Placebo: 4.8 (1.1) / 4.2 (1.7)		H/UC	H/UC	L	L
	Stammers (1992) [RCT] [25]	Fish oil vs placebo, change from baseline to 6 months<br>SMD 0.13 (-0.30, 0.55)	VAS disability, change from bl to 6 months, mean (SD)<br>Fish oil: -2 (17)<br>Placebo: -4 (15)		H/UC	H/UC	H/UC	H/UC
Bone mineral density	Chen (2016) [RCT] [18]		Bone mineral density, high vs low dose, regression coefficient (95% CI) [fully adjusted]<br>Lumbar spine: 4.7 (-8.5, 17.9)<br>Femoral neck: -3.8 (-12.5, 4.9)		L	L	L	L

*SD calculated from standard error in paper

Alloc. Conc. = allocation concealment, AMSTAR2 = Assessing the Methodological Quality of Systematic Reviews, BL = baseline, Blind. Asses. = Blinded assessors, Blind. Part. = blinded participants, CI = confidence interval, H/UC = high / unclear risk of bias, L = low risk of bias, LI = Lequesne Index, MA = meta-analysis, OA = osteoarthritis, Rand. Seq. = random sequence generation, RCT = randomised controlled trial, SD = standard deviation, SE = standard error, SMD = Standardised mean difference, VAS = visual analogue scale, WOMAC = Western Ontario and McMaster Universities Osteoarthritis Index

Supplementary table 12 – Green lipped mussel extract and OA progression, results

Table – Green-lipped mussel extract (OA), results and quality assessment

Outcome	Study (date) [study type]	Standardised result, SMD (95% CI) unless otherwise stated	Natural result	AMSTAR2 quality	Rand. Seq.	Alloc. Conc.	Blind. Part.	Blind. Asses.
Pain	Liu (2018) [MA] [14]	Green-lipped mussel extract vs placebo<br>SMD -0.37 (-0.81, 0.08)		Moderate				

Alloc. Conc. = allocation concealment, AMSTAR2 = Assessing the Methodological Quality of Systematic Reviews, Blind. Asses. = Blinded assessors, Blind. Part. = blinded participants, CI = confidence interval, H/UC = high / unclear risk of bias, L = low risk of bias, MA = meta-analysis, OA = osteoarthritis, Rand. Seq. = random sequence generation, SMD = Standardised mean difference,

Supplementary table 13 – Promerim and OA progression, results

Table – Promerim (OA), results and quality assessment

Outcome	Study (date) [study type]	Standardised result, SMD (95% CI) unless otherwise stated	Natural result	AMSTAR2 quality	Rand. Seq.	Alloc. Conc.	Blind. Part.	Blind. Asses.
Pain	Kilinc (2018) [Single arm][26]		VAS pain, pre / post intervention, mean (SD)<br>5.6 (1.1) / 2.6 (1.7) p<0.001					
WOMAC total	Kilinc (2018) [Single arm][26]		WOMAC total, pre / post intervention, mean (SD)<br>46.4 (8.2) / 72.1 (14.4) p<0.001 §					

§ The paper appears to have reversed the scale of the WOMAC, so that higher scores indicate improved health, although this is not certain.

Alloc. Conc. = allocation concealment, AMSTAR2 = Assessing the Methodological Quality of Systematic Reviews, Blind. Asses. = Blinded assessors, Blind. Part. = blinded participants, CI = confidence interval, H/UC = high / unclear risk of bias, L = low risk of bias, OA = osteoarthritis, Rand. Seq. = random sequence generation, SD = standard deviation, SMD = Standardised mean difference, VAS = visual analogue scale, WOMAC = Western Ontario and McMaster Universities Osteoarthritis Index

Supplementary table 14 – Description of reviews of experimental diets in OA

Table – Experimental diets (OA), description of reviews

Authors (date)	Review type	Study type included	Type of OA	Exposure detail	Number of studies included	Funders
Alrushud (2017) [28]	MA	RCTs	Knee	Caloric restriction + physical activity	5 (2 included in MA)	**University** (King Saud University, Saudi Arabia), **Government** (Saudi Arabian Cultural Bureau)

MA = meta-analysis, OA = osteoarthritis, RCT = randomised controlled trial

Supplementary table 15 – Description of studies of experimental diets in OA

Table – Experimental diets (OA), description of included studies

Author (date) [country]	Study design	Type of OA	Inclusion criteria	Exposure detail	N	Age, mean (SD) years	N (%) female	Funders
Dyer (2017) [UK] [29]	RCT	not reported	OA, aged 31-90 years<br>Exclusions: comorbidity meaning they cannot follow diet, participating in other interventional research, prior involvement with Arthritis Action	1) Nutritional and dietary advice in line with Mediterranean diet given. Telephone support offered.<br>p) Control followed no intervention	1) 50<br>p) 49	1) 66 (11)<br>p) 60 (12)	1) 38 (76)<br>p) 44 (88)	**Charity** (Arthritis Action)
Clinton (2015) [USA] [30]	RCT	not reported	OA, aged 18-70 years<br>Exclusion: history of eating disorder, diabetes, inability to afford food, lack of control over food, pregnant or nursing, food allergies, following other medically prescribed diet	1) WFPB consists of fruits, vegetables, legumes and grains. No energy consumption restriction but encouraged to get at least 90% of calories from plants<br>P) Control: ordinary diet	1) 19<br>p) 18	1) 56.1 (8.4)<br>p) 60.0 (6.3)	1) 15 (78.9)<br>p) 16 (88.9)	**Charity** (Blue Cross Blue Shield)
Riecke (2010) [Denmark] [31]	RCT	Knee	Obese (BMI>30), aged >50 years, ACR OA criteria<br>Exclusions: previous/planned knee replacement, surgery or injections in knee in past 3 months, weight reducing drugs, lack of motivation to lose weight, inability to speak Danish	8 weeks of low calories:<br>1) 810 kcal per day<br>2) 415 kcal per day<br>Both groups then had 8 more weeks of 1200 kcal per day	1) 96<br>2) 96	1) 63.3 (6.3)<br>2) 61.8 (6.4)	1) 77 (80.2)<br>2) 78 (81.3)	**Charity** (The Oak Foundation, The Velux Foundation, The Augustinus Foundation, The A.P. Møller Foundation, Erik Hørslev og hustru BirgitHørslevs Fond, Aase og Ejnar Danielsens fond and Bjarne Jensens Fond) **Industry** (Cambridge Weight Plan) **Professional body** (Danish Rheumatism Association)
Lopez-Gomez (2018) [Spain] [32]	Single arm int.	Knee	Obese, pending surgery, knee OA	Nutrition education + hypocaloric diet (diet structured into 6 meals – lunch and dinner replaced by “oral nutritional supplement”	75	62.2 (8.5)	75 (100)	Not reported

ACR = American College of Rheumatology, BMI = Body Mass Index, Int. = intervention, kcal = kilocalories, N = number, OA = osteoarthritis, RCT = randomised controlled trial, SD = standard deviation, UK = United Kingdom, USA = United States of America, WFPB = Whole Food Plant Based

Supplementary table 16 – Calorie restriction and OA progression, results

Table – Calorie restriction (OA), results and quality assessment

Outcome	Study (date) [study type]	Standardised result, SMD (95% CI) unless otherwise stated	Natural result	AMSTAR2 quality	Rand. Seq.	Alloc. Conc.	Blind. Part.	Blind. Asses.
Pain	Alrushud (2017) [MA] [28]	Diet restriction vs exercise control at 18 months<br>SMD -0.24 (-0.50, 0.02)	WOMAC pain, BL / 18 months, mean (SD)<br>diet + exercise: 6.7 (3.4) / 3.7 (3.1)<br>exercise control: 6.1 (2.9) / 4.4 (2.7)<br>[1 study – Messier et al 2013 [33]]	Moderate				
	Riecke (2010) [RCT] [31]	415 kcal vs 810 kcal at 16 weeks<br>SMD -0.06 (-0.34, 0.22)	Pain VAS, change from BL – 16 weeks, mean (SD*)<br>810 kcal: -10.5 (17.93)<br>415 kcal: -11.6 (18.62); p=0.68		L	L	L	L
	Lopez-Gomez (2018) [single arm] [32]		WOMAC pain, BL / 3 months, mean (SD)<br>Calorie restriction: 52.94 (26.08) / 45.25 (23.57)<br>p<0.01					
Function	Alrushud (2017) [MA] [28]	Diet restriction vs exercise control at 18 months<br>SMD -0.34 (-0.59, -0.08)	WOMAC function, BL / 18 months, mean (SD)<br>diet + exercise: 24.6 (11.7) / 14.2 (10.4)<br>exercise control: 23.1 (10.3) / 17.6 (9.8)<br>[1 study – Messier et al 2013 [33]]	Moderate				
	Riecke (2010) [RCT] [31]	415 kcal vs 810 kcal at 16 weeks<br>SMD -0.08 (-0.37, 0.20)	Function VAS, change from BL – 16 weeks, mean (SD*)<br>810 kcal: -12.75 (18.91)<br>415 kcal: -14.44 (22.05); p0.57		L	L	L	L
	Lopez-Gomez (2018) [single arm] [32]		WOMAC function, BL / 3 months, mean (SD)<br>Calorie restriction: 49.19 (27.01) / 40.16 (22.06 – 54.41) [sic] p<0.01					
Stiffness	Lopez-Gomez (2018) [single arm] [32]		WOMAC stiffness, BL / 3 months, median (IQR)<br>Calorie restriction: 50 (25-75) / 25 (12.5-50); p=0.02					
6MWT	Alrushud (2017) [MA] [28]		Intervention vs exercise only control<br>meta-mean difference: 15.05 (-11.77, 41.87) in favour of intervention	Moderate				
QoL	Riecke (2010) [RCT] [31]	415 kcal vs 810 kcal at 16 weeks<br>SMD -0.03 (-0.32, 0.25)	KOOS QoL, change from BL – 16 weeks, mean (SD*)<br>810 kcal: 8.85 (15.68)<br>415 kcal: 8.31 (16.07); p=0.81		L	L	L	L

* calculated from standard error reported in paper

6MWT = six minute walk test, Alloc. Conc. = allocation concealment, AMSTAR2 = Assessing the Methodological Quality of Systematic Reviews, BL = baseline, Blind. Asses. = Blinded assessors, Blind. Part. = blinded participants, CI = confidence interval, H/UC = high / unclear risk of bias, IQR = interquartile range, kcal = kilocalories, KOOS = Knee Injury and Osteoarthritis Outcome Score, L = low risk of bias, QoL = Quality of life, Rand. Seq. = random sequence generation, RCT = randomised controlled trial, SD = standard deviation, SMD = Standardised mean difference, WOMAC = Western Ontario and McMaster Universities Osteoarthritis Index

Table – Calorie restriction, SF36 results, mean (SD)

Author (date) [BL]	PCS	MCS	GH	PF	RP	RE	SF	BP	V	MH
Lopez-Gomez (2018) [32] [BL]	-	-	41.49 (16.53)	25 (10-45) §	25 (0-100) §	69.13 (42.74)	75 (50-100) §	43.95 (23.68)	44.22 (23.68)	59.80 (27.40)
Lopez-Gomez (2018) [32] [FU]	-	-	48.79 (13.63)	75 (12.5-100) §	75 (12.5-100) §	81.85 (36.59)	87.5 (50-100) §	54.23 (27.76)	57.71 (54.34)	68.49 (22.98)
Riecke (2010) [810 kcal] †	6.07 (7.94 ‡)	1.32 (8.72 ‡)	-	-	-	-	-	-	-	-
Riecke (2010) [415 kcal] †	5.57 (8.13 ‡)	4.43 (8.03 ‡)	-	-	-	-	-	-	-	-

§ median (IQR)

† change from baseline to 16 weeks

‡ calculated from standard error in paper

BL = baseline, BP = bodily pain, FU = follow-up, GH = general health, IQR = interquartile range, MCS = mental component score, MH = mental health, PCS = physical component score, PF = physical function, RE = role emotional, RP = role physical, SD = standard deviation, SF = social functioning, V = vitality

Supplementary table 17 – Whole food, plant based diet and OA progression, results

Table – Whole food, plant based diet (OA), results and quality assessment

Outcome	Study (date) [study type]	Standardised result, SMD (95% CI) unless otherwise stated	Natural result	AMSTAR2 quality	Rand. Seq.	Alloc. Conc.	Blind. Part.	Blind. Asses.
Pain	Clinton (2015) [RCT] [30]		Pain VAS, week 6, mean (SD not reported)<br>WFPD: 2.21<br>Control: 2.38 p=NS		L	H/UC	H/UC	H/UC

Alloc. Conc. = allocation concealment, AMSTAR2 = Assessing the Methodological Quality of Systematic Reviews, Blind. Asses. = Blinded assessors, Blind. Part. = blinded participants, CI = confidence interval, H/UC = high / unclear risk of bias, L = low risk of bias, OA = osteoarthritis, Rand. Seq. = random sequence generation, RCT = randomised controlled trial, SD = standard deviation, SMD = Standardised mean difference, VAS = visual analogue scale, WFPB = whole food plant based

Table – Whole food plant based diet (OA), SF36 results at final follow-up

Author (date) [study arm]	PCS	MCS	GH	PF	RP	RE	SF	BP	V	MH
Clinton (2015) [WFPB] [30] †	7.44	9.97	7.15	7.11	9.29	7.97	10.47	8.61	11.97	9.48
Clinton (2015) [Control] [30] †	1.31‡	6.87	2.01 ‡	1.02 ‡	2.65 ‡	5.05	5.08	5.41	5.49 ‡	6.46

† change from baseline to 6 weeks, T score

‡ p<0.05, WFPB vs control

BP = bodily pain, GH = general health, MCS = mental component score, MH = mental health, PCS = physical component score, PF = physical function, RE = role emotional, RP = role physical, SF = social functioning, V = vitality, WFPB = whole food plant based

Supplementary table 18 – Mediterranean diet and OA progression, results

Table – Mediterranean diet (OA), results and quality assessment

Outcome	Study (date) [study type]	Standardised result, SMD (95% CI) unless otherwise stated	Natural result	AMSTAR2 quality	Rand. Seq.	Alloc. Conc.	Blind. Part.	Blind. Asses.
Function	Dyer (2017) [RCT] [29]	Med diet vs control at 4 months<br>SMD -0.18 (-0.58, 0.22)	AIMS2 function, BL / 4 months, mean (SD)<br>Med diet: 1.7 (1.5) / 1.6 (1.4)<br>Control: 2.0 (1.9) / 1.9 (1.9)		H/UC	H/UC	H/UC	H/UC
Affect	Dyer (2017) [RCT] [29]	Med diet vs control at 4 months<br>SMD -0.14 (-0.54, 0.25)	AIMS2 affect, BL / 4 months, mean (SD)<br>Med diet: 2.7 (1.8) / 2.6 (2.0)<br>Control: 3.4 (2.1) / 2.9 (2.2)		H/UC	H/UC	H/UC	H/UC

AIMS2 = Arthritis Impact and Measurement Scales 2, Alloc. Conc. = allocation concealment, AMSTAR2 = Assessing the Methodological Quality of Systematic Reviews, BL = baseline, Blind. Asses. = Blinded assessors, Blind. Part. = blinded participants, CI = confidence interval, H/UC = high / unclear risk of bias, L = low risk of bias, med = Mediterranean, OA = osteoarthritis, Rand. Seq. = random sequence generation, RCT = randomised controlled trial, SD = standard deviation, SMD = Standardised mean difference

Supplementary table 19 – Description of studies of food components in OA

Table – Food components (OA), description of included studies

Author (date) [country]	Study design	Type of OA	Inclusion criteria	Exposure detail	N	Age, mean (SD) years	N (%) female	Funders
Dai (2017) [USA] [34]	Pros. Cohort	Knee	Osteoarthritis Initiative, aged 45-79 years, absence of inflammatory arthritis	Fibre intake from food frequency questionnaire (quartiles)	3703	Q1) 59.7 (9.0)<br>Q2) 60.9 (9.1)<br>Q3) 61.8 (9.1)<br>Q4) 62.7 (9.1)	Q1: 1301 (58.1)<br>Q2: 1296 (58.1)<br>Q3: 1286 (57.5)<br>Q4: 1296 (58.0)	**Government** (NIH), **Industry** (OAI: Pfizer, Novartis, Merck, GSK)
Lu (2017) [USA] [35]	Pros. Cohort	Knee	Osteoarthritis Initiative, aged 45-79 years, all have radiographic OA in at least one knee<br>Exclusion: severe OA (KL grade = 4), difference of rim distance from tibial plateau to tibial rim closest to femoral condyle between baseline and any follow ≥2 mm	Fat intake from food frequency questionnaire (quartiles)	2092	Q1) 64.2 (8.7)<br>Q2) 62.8 (9.0)<br>Q3) 62.3 (9.1)<br>Q4) 60.8 (8.8)	Q1) (60)<br>Q2) (56.9)<br>Q3) (59)<br>Q4) 59.3)	**Government** (National Heart, Lung and Blood Institute, NIH), **Industry** (OAI: Pfizer, Novartis, Merck, GSK)

GSK = GlaxoSmithKline, KL = Kellgren Lawrence, N = number, NIH = National Institute for Health, OA = osteoarthritis, OAI = Osteoarthritis Initiative, pros. = prospective, Q1-4 = quartiles of fibre/fat intake, SD = standard deviation, USA = United States of America

Supplementary table 20 – Food components and OA progression, results

Table – Food components (OA), results and quality assessment

Outcome (outcome measure)	Study (date) [study type]	Standardised result, SMD (95% CI) unless otherwise stated	Natural result	Study Pop.	Attr.	Prog. Meas.	Outc. Meas.	Conf.	Stats.
Pain	Dai (2017) [Pros. Obs.] [34]		<u>Odds of being in mild / moderate / severe pain compared to no pain (95% CI)</u><br>Fibre intake Q2 vs Q1: 1.16 (0.97, 1.39) / 0.92 (0.75, 1.13) / 0.65 (0.48, 0.89)<br>Fibre intake Q3 vs Q1: 0.92 (0.77, 1.10) / 0.85 (0.70, 1.04) / 0.79 (0.59, 1.07)<br>Fibre intake Q4 vs Q1: 1.05 (0.88, 1.26) / 0.76 (0.61, 0.93) / 0.56 (0.41, 0.78)	L	L	M	L	L	L
JSW loss	Lu (2017) [Pros. Obs.] [35]		<u>JSW loss over follow-up, mean (SE)</u><br>*Total fat*<br>Q1: 0.26 (0.03); Q2: 0.27 (0.02)<br>Q3: 0.31 (0.02); Q4: 0.35 (0.03), p for trend = 0.02<br>*Saturated fat*<br>Q1: 0.25 (0.03); Q2: 0.26 (0.02)<br>Q3: 0.33 (0.02); 0.37 (0.03) p for trend <0.01<br>*Monounsaturated fat*<br>Q1: 0.36 (0.02); Q2: 0.29 (0.02);<br>Q3: 0.32 (0.02); Q4: 0.32 (0.02) p for trend = 0.19<br>*Polyunsaturated fat*<br>Q1: 0.34 (0.02); Q2: 0.31 (0.02);<br>Q3: 0.26 (0.02); Q4: 0.28 (0.02) p for trend = 0.02	L	L	M	L	L	L

Attr. = attrition, CI = confidence interval, Conf. = confounding, JSW = joint space width, L = low risk of bias, M = moderate risk of bias, OA = osteoarthritis, Outc. Meas = outcome measurement, Prog. Meas. = prognostic factor measurement, Q1-4 = quartiles of fibre/fat intake, Rand. Seq. = random sequence generation, SE = standard error, SMD = Standardised mean difference, Stats. = statistical analysis, Study Pop. = study population

Supplementary table 21 – Description of reviews of fruits, vegetables and other plant based interventions in OA

Table – Fruits, vegetables and other plant based interventions (OA), description of reviews

Authors (date)	Review type	Study type included	Type of OA	Exposure detail	Number of studies included	Funders
Liu (2018) [14]	MA	RCTs	Hip, knee or hand	Artemisia Annua extract<br>Avocado / soybean unsaponifiables<br>Boswellia Serrata<br>Bromelain<br>Curcuma Longa<br>Curcumin<br>Passion fruit<br>Pine tree extract	1<br>2<br>3<br>1<br>1<br>2<br>1<br>2	**Government** (NHMRC program grant, Department of education grant), **Industry** (PuraPharm postgrad scholarship), author disclosures (Flexion, Nestle, Merck)
Daily (2016) [36]	MA	RCTs	Knee	Turmeric extracts and its components	8	**Industry** (Korea Institute of Oriental Medicine), **Author disclosure** (lead author in president of a company that manufactures dietary supplements)
Cameron (2014) [37]	MA	RCTs	Hip, knee or hand	Avocado / soybean unsaponifiables<br>Boswellia Serrata	6<br>5	**Universities** (Victoria University, University of Freiberg, Australian Catholic University, University of the Sunshine Coast), **Government** (National Center for Complementary and Alternative Medicine)
Percope de Andrade (2015) [38]	SR	RCTs, other reviews	Hip and knee	Avocado / soybean unsaponifiables	4 RCTs, 1 review	**Not reported**, One author disclosed support from Zimmer (medical device company)
McAlindon (2014) [39]	SR	RCTs, other reviews	Knee	Avocado / soybean unsaponifiables	1 meta-analysis	**Professional body** (OARSI)

MA = meta-analysis, NHMRC = National Health and Medical Research Council, OA = osteoarthritis, OARSI = Osteoarthritis Research Society International, RCT = randomised controlled trial , SR = systematic review

Supplementary table 22 – Description of studies of fruits, vegetables and other plant based interventions in OA

Table – Fruits, vegetables and other plant based interventions (OA), description of included studies

Author (date) [country]	Study design	Type of OA	Inclusion criteria	Exposure detail	N	Age, mean (SD) years	N (%) female	Funders
Hashempur (2018) [Iran] [40]	RCT	Knee	Aged 40-75 years, mild-moderate OA (ACR criteria), symptoms for 6 months<br>Exclusions: Severe OA, ischemic heart disease, heart failure, hepatic and renal failure, pregnancy, lactation, history of gastrointestinal bleeding after NSAIDs, hypersensitivity or allergy to caffeine, alkaline drugs or warfarin use, recent initiation of joint protective activity, special diet for weight loss, recent change in physical activity, no ability to express pain.	1) Green tea extract tablets + diclofenac<br>p) Diclofenac only	1) 20<br>p) 20	1) 56.7 (8.1)<br>p) 53.1 (11.1)	1) 17 (85)<br>p) 15 (75)	**University** (Shiraz University of Medical Sciences)
Salimzadeh (2018) [Iran] [41]	RCT	Knee	Mild to moderate OA, ACR OA criteria, women, aged 50-75 years, post-menopause, BMI 25-40<br>Exclusions: severe pain, scheduled surgery, intra-articular therapy in last 3 months, NSAIDs or other analgesia, allergic to garlic, diabetes, other chronic disorders, on weight-loss protocol, smokers, HRT, omega-3 supplements, warfarin or other anti-coagulants	1) Odourless garlic tablets, 2x 500mg per day<br>p) Placebo tablets containing lactose	1) 39<br>p) 37	1) 58.9 (7.5)<br>p) 58.5 (7.4)	1) 39 (100)<br>p) 37 (100)	**University** (Tehran University of Medical Sciences and Health Services)
Essouiri (2017) [Morocco] [42]	RCT	Knee	ACR OA criteria<br>Exclusions: OA due to inflammatory arthritis, microcrystalline aetiology, patient had knee surgery, cancer, KL grade IV	1) Agran oil, 30 ml per day for 8 weeks<br>p) nothing (i.e. no placebo)	1) 51<br>p) 49	1) 58.2 (8.8)<br>p) 58.9 (5.6)	1) 51 (92.7)<br>p) 49 (94.2) [sic]	Not reported – authors declare to conflicts of interest
Karimifar (2017) [Iran] [43]	RCT	Knee	Aged 40-80 years, knee OA for ≥6 months based on ACR criteria, pain VAS >4cm, Lequesne pain and function index >7, CRP <10, ESR <20, KL grade II-III<br>Exclusions: Liver, renal or cardiac dysfunction, intra-articular steroids or hyaluronic acid within last 3 months, all other bone and joint disorders, peptic ulcer disease, knee arthroscopic procedure within last 3 months, pregnancy, lactation	1) Elaeagnus angustifolia capsule<br>2) Elaeagnus angustifolia capsule and Boswellia Thurifera capsules<br>p) Control	1) 23<br>2) 26<br>p) 26	1) 52.7 (11.1)<br>2) 52.0 (8.7)<br>p) 53.0 (8.6)	1) 21 (91.3)<br>2) 23 (88.5)<br>p) 22 (84.6)	**Industry** (Barij Essence Pharmaceutical Company)

ACR = American College of Rheumatology, BMI = body mass index, CRP = C-reactive protein, ESR = erythrocyte sedimentation rate, HRT = hormone replacement therapy, KL = Kellgren-Lawrence, ml = millilitre, N = number, NSAID = Non-steroidal anti-inflammatory drugs, OA = osteoarthritis, RCT = randomised controlled trial, SD = standard deviation, VAS = visual analogue scale

Table – Fruits, vegetables and other plant based interventions (OA), description of included studies

Author (date) [country]	Study design	Type of OA	Inclusion criteria	Exposure detail	N	Age, mean (SD) years	N (%) female	Funders
More (2017) [Germany] [44]	RCT	Knee	Aged 30-70 years, moderate pain (WOMAC 4-8)<br>Exclusions: pregnancy, knee pain for other reasons, allergies to study materials, serious disease, ingestion of other supplements, treatment with cartilage protecting medicine, steroids, NSAIDs (other than ASA, diclofenac or paracetamol), cortisone treatment in last 3 weeks, opioids, medication/alcohol/drug abuse, Acute meniscus injuries, Rheumatoid arthritis, Infection-associated arthritis, bone injury in lower extremities in last 12 months, disc prolapse, arthroscopic surgery in last 6 months, magnetics, shockwave or acupuncture therapy, simultaneous participation in another study, relationship with sponsor or investigator	1) Rose-Canina mix (fruit puree, U. dioica L. leaf dry extract, H. procumbens (Burch.) DC. Ex Meisn. or H. zeyheri Decne. (both species can be used for devil's claw preparations [28]) root dry extract) in liquid form<br>p) vegetable juice mix (olive oil, basil, vegetable juice concentrates)	1) 46<br>p) 44	1) 57.9 (8.3)<br>p) 55.7 (9.3)	1) 34 (73.9)<br>p) 33 (75.0)	**Industry** (Herbalist & Doc Gesundheitsgesellschaft mbH)
Rafraf (2017) [Iran] [45]	RCT	Knee	Women, aged 38-60 years, mild-moderate OA (ACR criteria), BMI 30-35<br>Exclusions: cardiovascular disease, diabetes, liver and kidney diseases, peptic or duodenal ulcer history, smoking, alcohol use, use of supplements (e.g. multivitamins, minerals) in past 4 weeks, allergy to pomegranate, use of NSAIDs	1) Dried pomegranate peel ground into powder and put into capsules<br>p) Placebo capsules filled with rice flower	1) 30<br>p) 30	1) 48.7 (7.8)<br>p) 52.2 (6.7)	1) 30 (100)<br>p) 30 (100)	**University** (Tabriz University of Medical Sciences)
Ghoochani (2016) [Iran] [46]	RCT	Knee	ACR criteria, aged 30-80 years<br>Exclusions: rheumatoid arthritis, diabetes, cardiovascular, liver or renal disease, cancer, consumption of antioxidants, pregnancy, treatment with oral/injectable steroids within 4 weeks or 6 months respectively	1) 200ml sugar and additive free pomegranate juice<br>p) followed usual lifestyle	1) 19<br>p) 19	1) 56.7 (10.2)<br>p) 53.8 (12.0)	1) 17 (89.5%)<br>p) 17 (89.5%)	**University** (Ahvaz Jundishapur University of Medical Sciences)
Haghighian (2015) [Iran] [47]	RCT	Knee	Aged 50-70 years, mild to moderate OA (ACR crit)<br>Exclusions: KL grade 1 or 4, BMI >35, cardiovascular disease, diabetes, liver or kidney disease, history of peptic or duodenal ulcers, smoking, alcohol use, use of supplements (e.g. multivitamins, minerals), allergy to sesame, using NSAIDs.	1) Sesame seed powder in 40g packs<br>p) Placebo powder	1) 22<br>p) 23	1) 56.9 (6.4)<br>p) 58.3 (7.8)	not reported	**University** (Tabriz University of Medical Sciences)

ACR = American College of Rheumatology, BMI = body mass index, KL = Kellgren-Lawrence, N = number, NSAID = non-steroidal anti-inflammatory drug, OA = osteoarthritis, RCT = randomised controlled trial, SD = standard deviation, WOMAC = Western Ontario and McMaster Universities Osteoarthritis Index

Table – Fruits, vegetables and other plant based interventions (OA), description of included studies

Author (date) [country]	Study design	Type of OA	Inclusion criteria	Exposure detail	N	Age, mean (SD) years	N (%) female	Funders
Arjmandi (2014) [USA] [48]	RCT	Knee	Overweight or obese, aged 40-90 years, KL grade I-III<br>Exclusions: history of liver / kidney disease or any other chronic or acute disease that might affect OA, allergy to shellfish or naproxen, knee surgery or significant injury in last 6 months, hyaluronan or cortisone injections in last 2 months	1) Capsules containing extracts of S. Baicalensis and A. Catechu<br>p) Naproxen	1) 45<br>p) 34	1) 63.8 (2.1)<br>p) 60.9 (1.8	1) 35 (77.7)<br>p) 26 (76.5)	**Industry** (Unigen, Inc.)
Ebrahimi (2014) [Iran] [49]	RCT	Knee	Mild to moderate OA, ACR criteria, female, aged 40-70 years, BMI 25-34.9<br>Exclusions: Secondary OA, active synovitis, neurological disorder affecting movement, uncontrolled hypertension, diabetes, CVD, kidney disorder, liver disorder, taking supplements, smokers	Elaeagnus angustifolia (Russian Olive)<br>1) whole fruit powder<br>2) powder made just from medulla<br>p) placebo made of corn starch	1) 26<br>2) 27<br>p) 25	1) 57.5 (7.2)<br>2) 54.5 (11.2)<br>p) 57 (7.8)	1) 26 (100)<br>2) 27 (100)<br>p) 25 (100)	**University** (Tabriz University)
Eftekhar Sadat (2013) [Iran] [50]	RCT	Knee	Aged 50-70 years, mild to moderate OA (ACR crit)<br>Exclusions: BMI >30 or <18.5, cardiovascular disease, history of peptic or duodenal ulcers, smoking, alcohol use, use of supplements (e.g. multivitamins, minerals), allergy to sesame.	1) Sesame seed powder in 40g packs<br>p) Standard drug therapy (no placebo)	1) 22<br>p) 23	not reported	81.82%	**University** (Tabriz University of Medical Sciences)
Paramdeep (2013) [India][51]	RCT (open label)	Knee	ACR Knee OA criteria, Knee pain 40-90mm on VAS<br>Exclusions: cardiovascular disease, hypertension, gastroduodenal disorders, diabetes, hepatic or renal impairment, bleeding disorders, pregnancy	1) diclofenac + placebo (lactose tablet)<br>2) 750mg tablet of ginger + placebo<br>3) diclofenac + ginder	1) 20<br>2) 20<br>3) 20	1) 54.8 (9.7)<br>2) 52.9 (8.1)<br>3) 50.1 (11.3)	1) 14 (70%)<br>2) 12 (60%)<br>3) 14 (70%)	Not reported
Schumacher (2013) [USA] [52]	RCT (cross-over)	Knee	Aged >18 years, mild-moderate OA that meets ACR criteria<br>Exclusions: systemic inflammatory conditions, chronic pain syndrome, steroid medication in last two months, hyaluronic acid injection in last 9 months, pregnancy, diabetes, inability to stop arthritis medication, food allergy, unstable medical conditions that would prevent completion	1) Cherry juice – prepared by mixing freshly prepared tart cherry juice with apple juice<br>p) Placebo juice – unsweetened black cherry Kool-aid soft drink with water	58	57 (11)	14 (24.1)	**Industry** (CherryPharm)

ACR = American College of Rheumatology, BMI = body mass index, CVD = cardiovascular disease, KL = Kellgren-Lawrence, N = number, OA = osteoarthritis, RCT = randomised controlled trial, SD = standard deviation, USA = United States of America, VAS = visual analogue scale

Table – Fruits, vegetables and other plant based interventions (OA), description of included studies

Author (date) [country]	Study design	Type of OA	Inclusion criteria	Exposure detail	N	Age, mean (SD) years	N (%) female	Funders
Kuehl et al 2012 [USA] [53]	RCT	not reported	Aged 40-70 years, at least moderate OA pain (>40mm VAS), 1990 ACR OA criteria, ability to maintain intervention, willingness to take drug<br>Exclusions: diabetes, not on stable pain medication, used non-pharmacological pain medication within last 30 days (e.g. acupuncture, ultrasound etc.)	1) Cherry juice – consumed two 10.5 oz bottles per day. 1 bottle = 50-60 cherries<br>p) Placebo juice – unsweetened cherry flavoured drink mixed with water. Cherry syrup and lemon juice added to match tartness	1) 10<br>p) 10	1) 55.9 (9.1)<br>p) 52.3 (14.2)	1) 10 (100)<br>p) 10 (100)	**Industry** (Cherry Research Committee), **University** (Oregon Clinical and Translational Research)
Myers (2010) [Australia] [54]	RCT	Knee	Aged 18-65, COAT score 3-7, willing to stop OA treatment<br>Exclusions: history of trauma with the affected joint, inflammatory joint conditions, steroid use in last 4 weeks, anti-inflammatory agents or anti-arthritic complementary therapy in last 3 weeks, liver function tests >3ULN, history of alcohol / substance abuse, lactating, pregnant, participated in another clinical trial in last 30 days, unwilling to have blood taken	1) 100mg of seaweed extracts<br>2) 1000mg of seaweed extracts<br>Interventions also included vitamin B6, zinc sulphate and manganese sulphate in formulation	1) 5<br>2) 7	Women: 61.2 (9.0)<br>Men: 57.1 (9.2)	6 (50)	**Industry** (Marinova Pty Ltd)
Frestedt (2009) [USA][55]	RCT	Knee	Ambulatory, aged 35-75, normal digestion, moderate-severe OA, met ACR criteria, WOMAC total ≤75, taking NSAIDs<br>Exclusion: rheumatoid arthritis, gout, Paget's disease, seizure disorder, diabetes, hypertension, cardiovascular disease, hepatic or renal disease, active cancer, HIV, prescription pain medication, involved in another clinical trial in past 3 months, lactating or at risk of pregnancy, intramuscular / systematic steroids within 1 month, intra-articular steroids within 2 months, hyaluronic acid within 4 months	1) Capsules of aquamin<br>p) Placebo capsules (maltodextran)	1) 8<br>p) 14	1) 62.5 (5.3)<br>p) 62.9 (11.4)	1) 7 (88)<br>p) 8 (57)	**Industry** (Marigot Ltd)
Oben (2009) [Cameroon] [56]	RCT	Knee	Aged 25-60 years, primary OA using ACR criteria<br>Exclusions: BMI >40, rheumatoid arthritis, joint replacement in either knee, unable to walk without assistance, enrolment in another clinical study in last 6 months, pregnancy, active infection, autoimmune disease, AIDS, HIV, active hepatitis, active malignancy, diabetes requiring insulin	1) Tablets containing blend of phellodendron amurense extract and citrus sinensis (L.) Osbeck [Rutaceae] peel extract<br>p) placebo capsules	1ov §) 20<br>1n §) 20<br>pov §) 20<br>pn §) 20	Not reported	not reported	**Industry** (Next Pharmaceuticals)

ACR = American College of Rheumatology, AIDS = acquired immune deficiency syndrome, BMI = body mass index, COAT = comprehensive arthritis test, HIV = human immunodeficiency virus, N = number, NSAID = non-steroidal anti-inflammatory drugs, OA = osteoarthritis, RCT = randomised controlled trial, SD = standard deviation, ULN = upper limit of normal, USA = United States of America, VAS = visual analogue scale, WOMAC = Western Ontario and McMaster Universities Osteoarthritis Index

Table – Fruits, vegetables and other plant based interventions (OA), description of included studies

Author (date) [country]	Study design	Type of OA	Inclusion criteria	Exposure detail	N	Age, mean (SD) years	N (%) female	Funders
Rein (2004) [Denmark] [57]	RCT	Various joints	X-ray verified OA<br>Exclusions: liver or kidney disease, allergies, drug/alcohol abuse, cancer, rheumatoid arthritis, fibromyalgia, gout, serious cardiovascular disease, asthma, any other disease that will reduce QoL, intra-articular hyaluroante, glucosamine sulphate, immunosuppressive drugs, steroids in past 6 weeks	1) Capsules of rose-canina fruit<br>p) identical placebo capsules	1) 56<br>p) 56	1) 67.1 (11.6)<br>p) 66.8 (11.8)	1) 37 (66.1)<br>p) 34 (60.7)	**Industry** (Hyben Vital International)
Warholm (2003) [Norway] [58]	RCT	Knee and hip	Radiographic OA, symptom duration <12 months, pain for >6 months or on list for surgery<br>Exclusions: Allergy to plant products, severe asthma, liver disease	1) capsules of powder produced from rose-canina fruit and seeds<br>p) placebo capsules	1) 50<br>p) 50	1) 63.3 (9.9)<br>p) 65.1 (12.2)	1) 31 (62.0)<br>p) 34 (68.0)	**Industry** (Hyben Vital International)
Piscoya (2001) [Peru] [59]	RCT	Knee	Aged 45-75 years, KL grade II-III, ACR criteria, pain most days of last month, requiring NSAID treatment<br>Exclusions: serious concomitant illness, secondary OA, hypersensitivity reactions to salicylates, intra-articular injection of steroids in last 3 months	1) Uncaria guianensis (Cat's Claw) extract in tablets<br>p) Placebo tablets	1) 30<br>p) 15	1) 59.9 (8.4)<br>p) 60.9 (6.5)	1) 0 (0)<br>p) 0 (0)	**Government** (Seguro Social del Peru, NIH)
Hunt (2016) [New Zealand] [60]	Single arm int.	Hip or knee	Hip or knee OA	Artemisia annua	28	62 (range 45-75)	16 (47.1)	**Industry** (Promisia Ltd)

ACR = American College of Rheumatology, KL = Kellgren-Lawrence, N = number, NSAID = Non-steroid anti-inflammatory drugs, OA = osteoarthritis, QoL = quality of life, RCT = randomised controlled trial, SD = standard deviation

Supplementary table 23 – Aquamin and OA progression, results

Table – Aqumin (red mineral algae) (OA), results and quality assessment

Outcome	Study (date) [study type]	Standardised result, SMD (95% CI) unless otherwise stated	Natural result	AMSTAR2 quality	Rand. Seq.	Alloc. Conc.	Blind. Part.	Blind. Asses.
Pain	Frestedt (2009) [RCT][55]	Aquamin vs placebo, change BL-12 weeks<br>SMD 0.34 (-0.54, 1.21)	WOMAC pain, change from BL – 12 weeks, mean (SD*)<br>Aquamin: 10.83 (23.48)<br>Placebo: 5.38 (10.48); p=0.63		L	L	L	L
Function	Frestedt (2009) [RCT][55]	Aquamin vs placebo, change BL-12 weeks<br>SMD 0.50 (-0.39, 1.38)	WOMAC function, change from BL – 12 weeks, mean (SD*)<br>Aquamin: 14.72 (25.57)<br>Placebo: 6.54 (8.08); p=0.43		L	L	L	L
Stiffness	Frestedt (2009) [RCT][55]	Aquamin vs placebo, change BL-12 weeks<br>SMD 0.23 (-0.65, 1.10)	WOMAC stiffness, change from BL – 12 weeks, mean (SD*)<br>Aquamin: 10.42 (35.84)<br>Placebo: 4.81 (16.35); p=0.83		L	L	L	L
6MWT	Frestedt (2009) [RCT][55]	Aquamin vs placebo, change BL-12 weeks<br>SMD 1.11 (0.17, 2.04)	6MWT, change from BL – 12 weeks, mean (SD*)<br>Aquamin: 150 (135.76)<br>Placebo: 12.5 (117.86); p=0.03		L	L	L	L

* Calculated from standard error reported in the paper; 6MWT = six minute walk test, Alloc. Conc. = allocation concealment, AMSTAR2 = Assessing the Methodological Quality of Systematic Reviews, BL = baseline, Blind. Asses. = Blinded assessors, Blind. Part. = blinded participants, CI = confidence interval, H/UC = high / unclear risk of bias, L = low risk of bias, OA = osteoarthritis, Rand. Seq. = random sequence generation, RCT = randomised controlled trial, SD = standard deviation, SMD = Standardised mean difference, WOMAC = Western Ontario and McMaster Universities Osteoarthritis Index

Supplementary table 24 – Argan oil and OA progression, results

Table – Argan oil (OA), results and quality assessment

Outcome	Study (date) [study type]	Standardised result, SMD (95% CI) unless otherwise stated	Natural result	AMSTAR2 quality	Rand. Seq.	Alloc. Conc.	Blind. Part.	Blind. Asses.
Pain	Essouiri (2017) [RCT] [42]	Pain at 8 weeks<br>SMD -0.28 (-0.67, 0.11)	WOMAC pain, BL / 8 weeks, mean (SD)<br>Argan oil: 6.55 (4.17) / 4.86 (3.93)<br>Control: 5.2 (3) / 5.84 (3); p<0.0001		L	H/UC	H/UC	H/UC
Function	Essouiri (2017) [RCT] [42]	Function at 8 weeks<br>SMD -0.72 (-1.21, -0.31)	WOMAC function, BL / 8 weeks, mean (SD)<br>Argan oil: 15.73 (7.62) / 11.71 (6.33)<br>Control: 14 (6.41) / 16.2 (6.2); p<0.0001		L	H/UC	H/UC	H/UC
Stiffness	Essouiri (2017) [RCT] [42]	Stiffness at 8 weeks<br>SMD -0.27 (-0.66, 0.13)	WOMAC stiffness, BL / 8 weeks, mean (SD)<br>Argan oil: 3.86 (2.5) / 3.69 (3.46)<br>Control: 3.82 (2.21) / 4.45 (2); p=0.1		L	H/UC	H/UC	H/UC

Alloc. Conc. = allocation concealment, AMSTAR2 = Assessing the Methodological Quality of Systematic Reviews, BL = baseline, Blind. Asses. = Blinded assessors, Blind. Part. = blinded participants, CI = confidence interval, H/UC = high / unclear risk of bias, L = low risk of bias, OA = osteoarthritis, Rand. Seq. = random sequence generation, RCT = randomised controlled trial, SD = standard deviation, SMD = Standardised mean difference, WOMAC = Western Ontario and McMaster Universities Osteoarthritis Index

Supplementary table 25 – Artemisia Annua and OA progression, results

Table – Artemisia Annua (OA), results and quality assessment

Outcome	Study (date) [study type]	Standardised result, SMD (95% CI) unless otherwise stated	Natural result	AMSTAR2 quality	Rand. Seq.	Alloc. Conc.	Blind. Part.	Blind. Asses.
Pain	Liu (2018) [MA] [14]	Artemisia annua vs placebo<br>SMD -0.37 (-1.03, 0.29)		Moderate				
	Hunt (2016) [Single arm] [60]		WOMAC pain, BL / 36 weeks, mean (SD)<br>8.6 (3.0) / 5.9 (4.0)					
Function	Liu (2018) [MA] [14]	Artemisia annua vs placebo<br>SMD -0.15 (-0.81, 0.50)		Moderate				
	Hunt (2016) [Single arm] [60]		WOMAC function, BL / 36 weeks, mean (SD)<br>28.6 (21.2) / 21.9 (15.1)					
Stiffness	Hunt (2016) [Single arm] [60]		WOMAC stiffness, BL / 36 weeks, mean (SD)<br>3.9 (1.6) / 3.3 (7.2)					

Alloc. Conc. = allocation concealment, AMSTAR2 = Assessing the Methodological Quality of Systematic Reviews, BL = baseline, Blind. Asses. = Blinded assessors, Blind. Part. = blinded participants, CI = confidence interval, H/UC = high / unclear risk of bias, L = low risk of bias, MA = meta-analysis, OA = osteoarthritis, Rand. Seq. = random sequence generation, SD = standard deviation, SMD = Standardised mean difference, WOMAC = Western Ontario and McMaster Universities Osteoarthritis Index

Supplementary table 26 – Avocado / soybean unsaponifiables and OA progression, results

Table – Avocado / soybean unsaponifiables (OA), results and quality assessment

Outcome	Study (date) [study type]	Standardised result, SMD (95% CI) unless otherwise stated	Natural result	AMSTAR2 quality	Rand. Seq.	Alloc. Conc.	Blind. Part.	Blind. Asses.
Pain	Liu (2018) [MA] [14]	ASU vs placebo<br>Short term: SMD -0.57 (-0.95, -0.19)		Moderate				
	Cameron (2014) [MA] [37]	ASU vs placebo<br>-8% (-16%, -1%) reduction		High				
	Percope de Andrade (2015) [SR] [38]		1/4 RCTs showed reductions in pain, 1 review did not support symptom modifying effect of ASU	Moderate				
	McAlindon (2014) [SR] [39]	ASU vs placebo<br>1 MA from 2008: SMD 0.39 (0.76, 0.01)		Moderate				
Function	Liu (2018) [MA] [14]	ASU vs placebo<br>Short term: SMD -0.48 (-0.69, -0.28)		Moderate				
	Cameron (2014) [MA] [37]	ASU vs placebo<br>-7% (-12%, -2%) reduction		High				

Alloc. Conc. = allocation concealment, AMSTAR2 = Assessing the Methodological Quality of Systematic Reviews, ASU = Avocado / soybean unsaponifiables, Blind. Asses. = Blinded assessors, Blind. Part. = blinded participants, CI = confidence interval, H/UC = high / unclear risk of bias, L = low risk of bias, MA = meta-analysis, OA = osteoarthritis, Rand. Seq. = random sequence generation, RCT = randomised controlled trial, SMD = Standardised mean difference, SR = systematic review

Supplementary table 27 – Boswellia serrata and OA progression, results

Table – Boswellia Serrata (OA), results and quality assessment

Outcome	Study (date) [study type]	Standardised result, SMD (95% CI) unless otherwise stated	Natural result	AMSTAR2 quality	Rand. Seq.	Alloc. Conc.	Blind. Part.	Blind. Asses.
Pain	Liu (2018) [MA] [14]	Boswellia serrata vs placebo<br>Short term: SMD -1.61 (-2.10, -1.13)		Moderate				
	Cameron (2014) [MA] [37]	Boswellia serrata vs placebo<br>Pain rated 17 points lower (8, 26) on 0-100 point scale		High				
Function	Liu (2018) [MA] [14]	Boswellia serrata vs placebo<br>Short term: SMD -1.15 (-1.63, -0.68)		Moderate				
	Cameron (2014) [MA] [37]	Boswellia serrata vs placebo<br>Function rated 8 points better (2, 14) on 100 point scale		High				

Alloc. Conc. = allocation concealment, AMSTAR2 = Assessing the Methodological Quality of Systematic Reviews, Blind. Asses. = Blinded assessors, Blind. Part. = blinded participants, CI = confidence interval, H/UC = high / unclear risk of bias, L = low risk of bias, MA = meta-analysis, OA = osteoarthritis, Rand. Seq. = random sequence generation, SMD = Standardised mean difference

Supplementary table 28 – Bromelain and OA progression, results

Table – Bromelain (OA), results and quality assessment

Outcome	Study (date) [study type]	Standardised result, SMD (95% CI) unless otherwise stated	Natural result	AMSTAR2 quality	Rand. Seq.	Alloc. Conc.	Blind. Part.	Blind. Asses.
Pain	Liu (2018) [MA] [14]	Bromelain vs placebo<br>Short term: SMD -0.05 (-0.75, 0.64)		Moderate				
Function	Liu (2018) [MA] [14]	Bromelain vs placebo<br>Short term: SMD -0.34 (-1.04, 0.36)		Moderate				

Alloc. Conc. = allocation concealment, AMSTAR2 = Assessing the Methodological Quality of Systematic Reviews, Blind. Asses. = Blinded assessors, Blind. Part. = blinded participants, CI = confidence interval, MA = meta-analysis, OA = osteoarthritis, Rand. Seq. = random sequence generation, SMD = Standardised mean difference

Supplementary table 29 – Cherry juice and OA progression, results

Table – Cherry juice (OA), results and quality assessment

Outcome	Study (date) [study type]	Standardised result, SMD (95% CI) unless otherwise stated	Natural result	AMSTAR2 quality	Rand. Seq.	Alloc. Conc.	Blind. Part.	Blind. Asses.
Pain	Schumacher (2013) [RCT] [52]	Cherry juice vs placebo at 6 weeks<br>SMD -0.14 (-0.55, 0.27)	WOMAC pain, BL / 6 weeks, mean (SD)<br>Cherry juice: 42.1 (22.9) / 36.3 (27)<br>Placebo: 41.5 (24.4) / 40.0 (26.6); p=0.24		L	H/UC	L	L
Function	Schumacher (2013) [RCT] [52]	Cherry juice vs placebo at 6 weeks<br>SMD -0.21 (-0.62, 0.20)	WOMAC function, BL / 6 weeks, mean (SD)<br>Cherry juice: 46.9 (23.7) / 39.1 (25.9)<br>Placebo: 46.7 (24.0) / 44.7 (27.2); p=0.13		L	H/UC	L	L
Stiffness	Schumacher (2013) [RCT] [52]	Cherry juice vs placebo at 6 weeks<br>Cherry juice 1st: SMD -0.11 (-0.68, 0.47)<br>Cherry juice 2nd: SMD -0.11 (-0.69, 0.47)<br>[Comparing change scores of cherry juice first was significant in paper]	WOMAC stiffness, BL / 6 weeks, mean (SD)<br>Cherry juice 1st: 51.1 (29.3) / 39.1 (30.1)<br>Placebo: 39.5 (34.3) / 42.4 (32.8) ; p=0.048<br>Cherry juice 2nd: 48.3 (25.8) / 44.0 (28.5)<br>Placebo: 55.1 (19.8) / 47.0 (26.8) § p=0.29		L	H/UC	L	L
CRP	Schumacher (2013) [RCT] [52]	Cherry juice vs placebo at 6 weeks<br>SMD -0.92 (-1.35, -0.49)	CRP, BL / 6 weeks, mean (SD)<br>Cherry juice: 2.38 (1.83) / 1.98 (1.73)<br>Placebo: 2.99 (2.39) / 4.21 (2.98)		L	H/UC	L	L
	Kuehl (2012) [RCT]	Cherry juice vs placebo at 21 days<br>SMD 0.05 (-0.82, 0.93)	CRP, BL / 21 days, mean (SD)<br>Cherry juice: 7.19 (6.67) / 3.77 (4.57)<br>Placebo: 2.61 (3.32) / 3.55 (3.56)<br>change score p=0.016		H/UC	H/UC	L	L
	Bespoke MA of:<br>Schumacher 2013<br>Kuehl 2012	Cherry juice vs placebo<br>Meta-SMD -0.51 (-1.45, 0.43)<br>I^2 73.6%						

§ stiffness in twice as the intervention x time interaction was significant (cross-over trial)

Alloc. Conc. = allocation concealment, AMSTAR2 = Assessing the Methodological Quality of Systematic Reviews, BL = baseline, Blind. Asses. = Blinded assessors, Blind. Part. = blinded participants, CI = confidence interval, CRP = C-Reactive Protein, H/UC = high / unclear risk of bias, L = low risk of bias, MA = meta-analysis, OA = osteoarthritis, Rand. Seq. = random sequence generation, RCT = randomised controlled trial, SD = standard deviation, SMD = Standardised mean difference, WOMAC = Western Ontario and McMaster Universities Osteoarthritis Index

Supplementary table 30 – Curcuma longa and OA progression, results

Table – Curcuma longa, results and quality assessment

Outcome	Study (date) [study type]	Standardised result, SMD (95% CI) unless otherwise stated	Natural result	AMSTAR2 quality	Rand. Seq.	Alloc. Conc.	Blind. Part.	Blind. Asses.
Pain	Liu (2018) [MA] [14]	<u>Curcuma longa vs placebo</u><br>Short term: SMD -1.63 (-2.22, -1.03)		Moderate				
Function	Liu (2018) [MA] [14]	<u>Curcuma longa vs placebo</u><br>Short term: SMD -1.27 (-1.83, -0.70)		Moderate				

Alloc. Conc. = allocation concealment, AMSTAR2 = Assessing the Methodological Quality of Systematic Reviews, Blind. Asses. = Blinded assessors, Blind. Part. = blinded participants, CI = confidence interval, H/UC = high / unclear risk of bias, L = low risk of bias, MA = meta-analysis, OA = osteoarthritis, Rand. Seq. = random sequence generation, SMD = Standardised mean difference

Supplementary table 31 – Curcumin and OA progression, results

Table – Curcumin (OA), results and quality assessment

Outcome	Study (date) [study type]	Standardised result, SMD (95% CI) unless otherwise stated	Natural result	AMSTAR2 quality	Rand. Seq.	Alloc. Conc.	Blind. Part.	Blind. Asses.
Pain	Liu (2018) [MA] [14]	Curcumin vs placebo<br>Short term: SMD -1.19 (-1.93, -0.45)		Moderate				
Function	Liu (2018) [MA] [14]	Curcumin vs placebo<br>Short term: SMD -1.13 (-1.80, -0.46)		Moderate				

Alloc. Conc. = allocation concealment, AMSTAR2 = Assessing the Methodological Quality of Systematic Reviews, Blind. Asses. = Blinded assessors, Blind. Part. = blinded participants, CI = confidence interval, MA = meta-analysis, OA = osteoarthritis, Rand. Seq. = random sequence generation, SMD = Standardised mean difference

Supplementary table 32 – Fruit powder and OA progression, results

Table – Fruit powder (OA), results and quality assessment

Outcome	Study (date) [study type]	Standardised result, SMD (95% CI) unless otherwise stated	Natural result	AMSTAR2 quality	Rand. Seq.	Alloc. Conc.	Blind. Part.	Blind. Asses.
Pain	Ebrahimi (2014) [RCT] [49]	Whole fruit powder vs placebo at 8 weeks<br>SMD -0.39 (-0.94, 0.17)<br>Medulla powder vs placebo at 8 weeks<br>SMD -0.51 (-1.06, 0.05)	WOMAC pain, BL / 8 weeks, mean (SD)<br>Whole fruit: 9.08† (4.58) / 7.62 (4.67)<br>Medulla: 9.75 (5.54) / 7.04 (4.92)<br>Placebo: 9.95 (3.71) / 9.30 (3.93)		L	L	L	L
	Karimifar (2017) [RCT] [43]	Elaeagnus angustifoli vs control at 4 weeks<br>SMD -0.37 (-0.94, 0.19)<br>Elaeagnus angustifoli + Boswellia Thurifera vs control at 4 weeks<br>SMD -0.25 (-0.80, 0.29)	Pain VAS, BL / 4 weeks, mean (SD)<br>Elaeagnus angustifoli: 7.04 (1.15) / 4.65 (1.84)<br>Elaeagnus angustifolia + Boswellia Thurifera: 7.03 (1.36) / 4.84 (1.96)<br>Control: 7.01 (1.25) / 5.30 (1.66); p=0.304		H/UC	H/UC	H/UC	H/UC
Function	Ebrahimi (2014) [RCT] [49]	Whole fruit powder vs placebo at 8 weeks<br>SMD -0.32 (-0.87, 0.24)<br>Medulla powder vs placebo at 8 weeks<br>SMD -0.67 (-1.23, -0.11)	WOMAC function, BL / 8 weeks, mean (SD)<br>Whole fruit: 23.66 (13.82) / 20.9 (13.96)<br>Medulla: 24.20 (12.12) / 17.78 (10.01)<br>Placebo: 25.91 (10.17) / 24.91 (11.16)		L	L	L	L
	Karimifar (2017) [RCT] [43]	Elaeagnus angustifoli vs control at 4 weeks<br>SMD -0.35 (-0.91, 0.22)<br>Elaeagnus angustifoli + Boswellia Thurifera vs control at 4 weeks<br>SMD -0.07 (-0.62, 0.47)	LPFI, BL / 4 weeks, mean (SD)<br>Elaeagnus angustifoli: 12.47 (2.88) / 8.32 (3.25)<br>Elaeagnus angustifolia + Boswellia Thurifera: 12.69 (3.35) / 9.09 (4.18)<br>Control: 12.84 (2.73) / 9.34 (2.66); p=0.578		H/UC	H/UC	H/UC	H/UC
Stiffness	Ebrahimi (2014) [RCT] [49]	Whole fruit powder vs placebo at 8 weeks<br>SMD -0.22 (-0.77, 0.33)<br>Medulla powder vs placebo at 8 weeks<br>SMD -0.24 (-0.78, 0.31)	WOMAC stiffness, BL / 8 weeks, mean (SD)<br>Whole fruit: 3.21 (2.08) / 2.56 (2.14)<br>Medulla: 4 (2.6) / 2.5 (2.34)<br>Placebo: 3.66 (2.63) / 3.08 (2.61)		L	L	L	L
Patient global	Karimifar (2017) [RCT] [43]	Elaeagnus angustifoli vs control at 4 weeks<br>SMD -0.24 (-0.80, 0.33)<br>Elaeagnus angustifoli + Boswellia Thurifera vs control at 4 weeks<br>SMD -0.64 (-1.20, -0.08)	Patient global VAS, BL / 4 weeks, mean (SD)<br>Elaeagnus angustifoli: 1.44 (0.62) / 2.38 (0.43)<br>Elaeagnus angustifolia + Boswellia Thurifera: 1.50 (0.68) / 2.17 (0.46)<br>Control: 1.79 (0.64) / 2.50 (0.57); p=0.202		H/UC	H/UC	H/UC	H/UC

†written in the paper as 90.08 – assumed this was a missprint

Alloc. Conc. = allocation concealment, AMSTAR2 = Assessing the Methodological Quality of Systematic Reviews, BL = baseline, Blind. Asses. = Blinded assessors, Blind. Part. = blinded participants, CI = confidence interval, H/UC = high / unclear risk of bias, L = low risk of bias, LPFI = Lequesne pain and function index, OA = osteoarthritis, Rand. Seq. = random sequence generation, RCT = randomised controlled trial, SD = standard deviation, SMD = Standardised mean difference, VAS = visual analogue scale, WOMAC = Western Ontario and McMaster Universities Osteoarthritis Index

Supplementary table 33 – Garlic and OA progression, results

Table – Garlic (OA), results and quality assessment

Outcome	Study (date) [study type]	Standardised result, SMD (95% CI) unless otherwise stated	Natural result	AMSTAR2 quality	Rand. Seq.	Alloc. Conc.	Blind. Part.	Blind. Asses.
Pain	Salimzadeh (2018) [RCT] [41]	Garlic vs placebo at 12 weeks<br>SMD 0.03 (-0.43, 0.47)	WOMAC pain baseline / 12 weeks, mean (SD)<br>Garlic: 8.3 (3.7) / (4.4)<br>Placebo: 9.6 (3.1) / 6.9 (3.7); p=0.475		L	H/UC	L	H/UC
Function	Salimzadeh (2018) [RCT] [41]	Garlic vs placebo at 12 weeks<br>SMD -0.17 (-0.62, 0.28)	WOMAC function at 12 weeks, mean (SD)<br>Garlic: 27.7 (11.9) / 22.2 (12.4)<br>Placebo: 27.8 (10.8) / 24.1 (10.2); p=0.221		L	H/UC	L	H/UC
Stiffness	Salimzadeh (2018) [RCT] [41]	Garlic vs placebo at 12 weeks<br>SMD -0.63 (-1.09, -0.17)	WOMAC stiffness at 12 weeks, mean (SD)<br>Garlic: 2.3 (1.6) / 1.4 (1.6)<br>Placebo: 2.7 (1.9) / 2.5 (1.9); p=0.023		L	H/UC	L	H/UC

Alloc. Conc. = allocation concealment, AMSTAR2 = Assessing the Methodological Quality of Systematic Reviews, Blind. Asses. = Blinded assessors, Blind. Part. = blinded participants, CI = confidence interval, H/UC = high / unclear risk of bias, L = low risk of bias, OA = osteoarthritis, Rand. Seq. = random sequence generation, RCT = randomised controlled trial, SD = standard deviation, SMD = Standardised mean difference, WOMAC = Western Ontario and McMaster Universities Osteoarthritis Index

Supplementary table 34 – Ginger and OA progression, results

Table – Ginger (OA), results and quality assessment

Outcome	Study (date) [study type]	Standardised result, SMD (95% CI) unless otherwise stated	Natural result	AMSTAR2 quality	Rand. Seq.	Alloc. Conc.	Blind. Part.	Blind. Asses.
Pain †	Paramdeep (2013) [RCT] [51]		<u>Pain VAS, % improvement from BL to 12 weeks</u><br>Diclofenac: 60.31%<br>Ginger: 59.11%<br>Ginger + diclofenac: 66.77%		H/UC	H/UC	H/UC	H/UC
WOMAC total	Paramdeep (2013) [RCT] [51]		<u>WOMAC total, % improvement from BL to 12 weeks</u><br>Diclofenac: 74.83%<br>Ginger: 63.68%<br>Ginger + diclofenac: 79.43%		H/UC	H/UC	H/UC	H/UC

† inclusion criteria states that the VAS used is a pain VAS, but for the rest of the paper the instrument is just referred to as the VAS – assuming that it is still measuring pain

Alloc. Conc. = allocation concealment, AMSTAR2 = Assessing the Methodological Quality of Systematic Reviews, BL = baseline, Blind. Asses. = Blinded assessors, Blind. Part. = blinded participants, CI = confidence interval, H/UC = high / unclear risk of bias, L = low risk of bias, OA = osteoarthritis, Rand. Seq. = random sequence generation, RCT = randomised controlled trial, SMD = Standardised mean difference, VAS = visual analogue scale, WOMAC = Western Ontario and McMaster Universities Osteoarthritis Index

Supplementary table 35 – Green tea extract and OA progression, results

Table – Green tea extract (OA), results and quality assessment

Outcome	Study (date) [study type]	Standardised result, SMD (95% CI) unless otherwise stated	Natural result	AMSTAR2 quality	Rand. Seq.	Alloc. Conc.	Blind. Part.	Blind. Asses.
Pain	Hashempur (2018) [RCT] [40]	Green tea vs control at 1 month<br>SMD 0.01 (-0.61, 0.63)	WOMAC pain, BL / 1 month, mean (SD)<br>Green tea: 10.45 (4.87) / 6.70 (4.31)<br>Control: 8.60 (3.42) / 6.65 (2.36); p=0.163		L	H/UC	H/UC	H/UC
Function	Hashempur (2018) [RCT] [40]	Green tea vs control at 1 month<br>SMD -0.13 (-0.75, 0.49)	WOMAC function, BL / 1 month, mean (SD)<br>Green tea: 31.15 (13.55) / 24.70 (13.94)<br>Control: 24.15 (9.73) / 26.15 (7.52); p=0.004		L	H/UC	H/UC	H/UC
Stiffness	Hashempur (2018) [RCT] [40]	Green tea vs control at 1 month<br>SMD -0.13 (-0.75, 0.49)	WOMAC function, BL / 1 month, mean (SD)<br>Green tea: 2.30 (1.86) / 1.65 (1.75)<br>Control: 1.85 (1.78) / 1.85 (1.38); p=0.150		L	H/UC	H/UC	H/UC

Alloc. Conc. = allocation concealment, AMSTAR2 = Assessing the Methodological Quality of Systematic Reviews, BL = baseline, Blind. Asses. = Blinded assessors, Blind. Part. = blinded participants, CI = confidence interval, H/UC = high / unclear risk of bias, L = low risk of bias, OA = osteoarthritis, Rand. Seq. = random sequence generation, RCT = randomised controlled trial, SD = standard deviation, SMD = Standardised mean difference, WOMAC = Western Ontario and McMaster Universities Osteoarthritis Index

Supplementary table 36 – Passion fruit and OA progression, results

Table – Passion fruit (OA), results and quality assessment

Outcome	Study (date) [study type]	Standardised result, SMD (95% CI) unless otherwise stated	Natural result	AMSTAR2 quality	Rand. Seq.	Alloc. Conc.	Blind. Part.	Blind. Asses.
Pain	Liu (2018) [MA] [14]	Passion fruit vs placebo<br>Short term : SMD -1.65 (-2.44, -0.86)		Moderate				
Function	Liu (2018) [MA] [14]	Passion fruit vs placebo<br>Short term : SMD -1.55 (-2.33, -0.77)		Moderate				

Alloc. Conc. = allocation concealment, AMSTAR2 = Assessing the Methodological Quality of Systematic Reviews, Blind. Asses. = Blinded assessors, Blind. Part. = blinded participants, CI = confidence interval, H/UC = high / unclear risk of bias, L = low risk of bias, MA = meta-analysis, Rand. Seq. = random sequence generation, SMD = Standardised mean difference

Supplementary table 37 – Pomegranate and OA progression, results

Table – Pomegranate (OA), results and quality assessment

Outcome	Study (date) [study type]	Standardised result, SMD (95% CI) unless otherwise stated	Natural result	AMSTAR2 quality	Rand. Seq.	Alloc. Conc.	Blind. Part.	Blind. Asses.
Pain	Rafraf (2017) [RCT] [45]	Pomegranate vs placebo at 8 weeks<br>SMD -0.55 (-1.07, -0.04) ‡	KOOS pain, BL / 8 weeks, mean (SD) †<br>Pomegranate: 47.68 (21.87) / 60.74 (21.55)<br>Placebo: 45.92 (23.47) / 48.14 (23.99); p=0.585		L	L	L	L
	Ghoochani (2016) [RCT] [46]	Pomegranate vs placebo at 6 weeks<br>SMD -0.53 (-1.18, 0.11)	WOMAC pain, BL / 6 weeks, mean (SD)<br>Pomegranate: 7.95 (4.99) / 7.32 (4.95)<br>Placebo: 9.63 (5.37) / 10.05 (5.18); p=0.10		H/UC	H/UC	H/UC	H/UC
	Bespoke MA of:<br>Rafraf 2017<br>Ghoochani 2016	Pomegranate vs placebo<br>Meta-SMD -0.54 (-0.95, -0.14)<br>I^2 = 0%						
Function	Rafraf (2017) [RCT] [45]	Pomegranate vs placebo at 8 weeks<br>SMD -0.30 (-0.81, 0.21) ‡	KOOS ADL, BL / 8 weeks, mean (SD) †<br>Pomegranate: 55.77 (19.31) / 69.17 (18.98)<br>Placebo: 56.79 (19.87) / 63.53 (18.58); p=0.263		L	L	L	L
	Ghoochani (2016) [RCT] [46]	Pomegranate vs placebo at 6 weeks<br>SMD -0.32 (-0.96, 0.32)	WOMAC function, BL / 6 weeks, mean (SD)<br>Pomegranate: 27.74 (10.56) / 22.53 (11.19)<br>Placebo: 25.47 (14.12) / 26.68 (14.35) p=0.32		H/UC	H/UC	H/UC	H/UC
	Bespoke MA of:<br>Rafraf 2017<br>Ghoochani 2016	Pomegranate vs placebo<br>Meta-SMD -0.31 (-0.71, 0.09)<br>I^2 = 0%						
QoL	Rafraf (2017) [RCT] [45]	Pomegranate vs placebo<br>SMD 0.18 (-0.32, 0.69) ‡ §	KOOS QoL, BL / 8 weeks, median (IQR) †<br>Pomegranate: 18.75 (4.67 - 37.5) / 31.25 (6.25 - 50.0)<br>Placebo: 37.5 (10.93 - 50.0) / 37.5 (12.5 - 56.25); p=0.548		L	L	L	L

† KOOS here = 0 (extreme problems) & 100 (no problems) – normally the other way round

‡ Effect size reversed here to fit into meta-analysis. Negative SMD = lower pain in treatment group compared to control

§ Mean and SD calculated from the median (IQR) using published formula[61]

ADL = activities of daily living, Alloc. Conc. = allocation concealment, AMSTAR2 = Assessing the Methodological Quality of Systematic Reviews, BL = baseline, Blind. Asses. = Blinded assessors, Blind. Part. = blinded participants, CI = confidence interval, H/UC = high / unclear risk of bias, IQR = interquartile range, KOOS = Knee injury and Osteoarthritis Outcome Score, L = low risk of bias, MA = meta-analysis, OA = osteoarthritis, QoL = quality of life, Rand. Seq. = random sequence generation, RCT = randomised controlled trial, SD = standard deviation, SMD = Standardised mean difference, WOMAC = Western Ontario and McMaster Universities Osteoarthritis Index

Supplementary table 38 – Rose canina mix and OA progression, results

Table – Rose-Canina mix (OA), results and quality assessment

Outcome	Study (date) [study type]	Standardised result, SMD (95% CI) unless otherwise stated	Natural result	AMSTAR2 quality	Rand. Seq.	Alloc. Conc.	Blind. Part.	Blind. Asses.
Pain	More (2017) [RCT] [44]	Rose-Canaina vs placebo, change from baseline to 12 weeks<br>SMD -2.49 (-3.04, -1.94)	WOMAC pain, change from BL to 12 weeks, mean (SD)<br>Rose-Canina: -29.87 (10.36)<br>Placebo: -10.23 (3.86); p<0.001		L	L	L	L
	Rein (2004) [RCT] [57]	Rose Canina vs placebo, change from baseline to 3 months<br>Group 1 §: -0.62 (-1.00, -0.24)<br>Group2 §: 0.20 (-0.17, 0.58)	Joint pain (0-4), change from baseline – 3 months, mean (SD)<br>*Group 1 §:*<br>Rose Canina: -1.91 (1.43)<br>Placebo: -1.02 (1.45); p=0.0078<br>*Group 2 §:*<br>Rose Canina: -1.45 (1.28)<br>Placebo: -1.72 (1.37); p=0.6084		L	L	L	L
	Warholm (2003) [RCT] [58]		Joint pain, N(%) reporting some effect over 4 months<br>Rose Canina: 31 (64.6%)<br>Placebo: 27 (56.3%) p=0.035		L	H/UC	L	H/UC
Function	More (2017) [RCT] [44]	Rose-Canaina vs placebo, change from baseline to 12 weeks<br>SMD -2.04 (-2.55, -1.53)	WOMAC function, change from BL to 12 weeks, mean (SD)<br>Rose-Canina: -23.82 (9.17)<br>Placebo: -9.17 (4.21)		L	L	L	L
Stiffness	More (2017) [RCT] [44]	Rose-Canaina vs placebo, change from baseline to 12 weeks<br>SMD -1.75 (-2.24, -1.26)	WOMAC stiffness, change from BL to 12 weeks, mean (SD)<br>Rose-Canina: -23.80 (11.84)<br>Placebo: -7.73 (5.11)		L	L	L	L
	Rein (2004) [RCT] [57]	Rose Canina vs placebo, change from baseline to 3 months<br>Group 1 §: -0.76 (-1.14, -0.38)<br>Group2 §: 0.31 (-0.07, 0.67)	Joint stiffness (0-4), change from baseline – 3 months, mean (SD)<br>*Group 1 §:*<br>Rose Canina: -1.91 (1.25)<br>Placebo: -0.91 (1.38); p=0.0025<br>*Group 2 §:*<br>Rose Canina: -1.28 (1.35)<br>Placebo: -1.71 (1.47); p=0.3850		L	L	L	L

§ Cross-over design: Group 1 received placebo and then active treatment, Group 2 received active treatment and then placebo

Alloc. Conc. = allocation concealment, AMSTAR2 = Assessing the Methodological Quality of Systematic Reviews, BL = baseline, Blind. Asses. = Blinded assessors, Blind. Part. = blinded participants, CI = confidence interval, H/UC = high / unclear risk of bias, L = low risk of bias, OA = osteoarthritis, Rand. Seq. = random sequence generation, RCT = randomised controlled trial, SD = standard deviation, SMD = Standardised mean difference, WOMAC = Western Ontario and McMaster Universities Osteoarthritis Index

Supplementary table 39 – S. Baicalensis and A. Catechu and OA progression, results

Table – S. Baicalensis and A. Catechu (OA), results and quality assessment

Outcome	Study (date) [study type]	Standardised result, SMD (95% CI) unless otherwise stated	Natural result	AMSTAR2 quality	Rand. Seq.	Alloc. Conc.	Blind. Part.	Blind. Asses.
6MWT	Arjmandi (2014) [RCT] [48]	S. Baicalensis and A. Catechu vs control<br>SMD 0.30 (-0.15, 0.75)	6MWT (m) at 1 week, mean (SD*)<br>S. Baicalensis and A. Catechu: 434.2 (75.67)<br>Control: 414.63 (47.29)		H/UC	H/UC	L	H/UC
CRP	Arjmandi (2014) [RCT] [48]	S. Baicalensis and A. Catechu vs control<br>SMD 0.06 (-0.39, 0.51)	CRP at 1 week, mean (SD*)<br>S. Baicalensis and A. Catechu: 3.11 (20.59)<br>Control: 2.02 (13.76)		H/UC	H/UC	L	H/UC

* SD calculated from standard error in paper

6MWT = six minute walk test, Alloc. Conc. = allocation concealment, AMSTAR2 = Assessing the Methodological Quality of Systematic Reviews, Blind. Asses. = Blinded assessors, Blind. Part. = blinded participants, CI = confidence interval, CRP = C-Reactive protein, H/UC = high / unclear risk of bias, L = low risk of bias, m= metres, OA = osteoarthritis, Rand. Seq. = random sequence generation, RCT = randomised controlled trial, SD = standard deviation, SMD = Standardised mean difference

Supplementary table 40 – Seaweed extract and OA progression, results

Table – Seaweed extract (OA), results and quality assessment

Outcome	Study (date) [study type]	Standardised result, SMD (95% CI) unless otherwise stated	Natural result	AMSTAR2 quality	Rand. Seq.	Alloc. Conc.	Blind. Part.	Blind. Asses.
Pain	Myers (2010) [RCT] [54]	1000mg vs 100mg at 12 weeks<br>SMD -0.83 (-2.04, 0.37)	COAT pain, BL / 12 weeks, mean (SD*)<br>100mg: 4.90 (1.79) / 3.83 (1.74)<br>1000mg: 4.79 (1.79) / 2.12 (2.24)		L	H/UC	H/UC	H/UC
Function	Myers (2010) [RCT] [54]	1000mg vs 100mg at 12 weeks<br>SMD -0.77 (-1.96, 0.43)	COAT function, BL / 12 weeks, mean (SD*)<br>100mg: 3.81 (1.70) / 3.67 (1.66)<br>1000mg: 4.80 (2.07) / 2.40 (1.66)		L	H/UC	H/UC	H/UC
Stiffness	Myers (2010) [RCT] [54]	1000mg vs 100mg at 12 weeks<br>SMD -0.75 (-1.95, 0.44)	COAT stiffness, BL / 12 weeks, mean (SD*)<br>100mg: 4.85 (1.73) / 3.61 (1.69)<br>1000mg: 4.72 (1.73) / 2.34 (1.69)		L	H/UC	H/UC	H/UC

* SD calculated from 95% CI in paper

Alloc. Conc. = allocation concealment, AMSTAR2 = Assessing the Methodological Quality of Systematic Reviews, BL = baseline, Blind. Asses. = Blinded assessors, Blind. Part. = blinded participants, CI = confidence interval, COAT = Comprehensive Osteoarthritis Test, H/UC = high / unclear risk of bias, L = low risk of bias, OA = osteoarthritis, Rand. Seq. = random sequence generation, RCT = randomised controlled trial, SD = standard deviation, SMD = Standardised mean difference

Supplementary table 41 – Sesame powder and OA progression, results

Table – Sesame powder (OA), results and quality assessment

Outcome	Study (date) [study type]	Standardised result, SMD (95% CI) unless otherwise stated	Natural result	AMSTAR2 quality	Rand. Seq.	Alloc. Conc.	Blind. Part.	Blind. Asses.
Pain	Eftekhar Sadat (2013) [RCT] [50]		Pain VAS at 2 months, median (IQR)<br>Sesame: 3.5 (4.25)<br>Control: 7 (3.00) § p=0.004		H/UC	H/UC	H/UC	H/UC
CRP	Haghighian (2015) [RCT] [47]	Sesame vs control at 2 months<br>SMD -0.23 (-0.82, 0.35) †	CRP, BL / 2 months, mean (SD)<br>Sesame: 1.45 (1.12) / 1.42 (1.32)<br>Control: 1.64 (1.19) / 1.68 (0.87); p=0.06		H/UC	H/UC	H/UC	H/UC

§ Cannot convert to mean (SD) to calculate SMD using formula[61] as need 25th and 75th centile, but only the difference between those centiles is reported

† p value in paper is 0.06

Alloc. Conc. = allocation concealment, AMSTAR2 = Assessing the Methodological Quality of Systematic Reviews, BL = baseline, Blind. Asses. = Blinded assessors, Blind. Part. = blinded participants, CI = confidence interval, CRP = C-reactive protein, H/UC = high / unclear risk of bias, IQR = interquartile range, L = low risk of bias, OA = osteoarthritis, Rand. Seq. = random sequence generation, RCT = randomised controlled trial, SD = standard deviation, SMD = Standardised mean difference, VAS = visual analogue scale

Supplementary table 42 – Tree bark extracts and OA progression, results

Table – Tree-bark extracts (OA), results and quality assessment

Outcome	Study (date) [study type]	Standardised result, SMD (95% CI) unless otherwise stated	Natural result	AMSTAR2 quality	Rand. Seq.	Alloc. Conc.	Blind. Part.	Blind. Asses.
Pain	Liu (2018) [MA] [14]	Pine tree extract (pycnogenol) vs placebo<br>Short term: SMD -1.21 (-1.53, -0.89)		Moderate				
	Karimifar (2017) [RCT] [43]	Elaeagnus angustifoli vs control at 4 weeks<br>SMD -0.37 (-0.94, 0.19)<br>Elaeagnus angustifoli + Boswellia Thurifera vs control at 4 weeks<br>SMD -0.25 (-0.80, 0.29)	Pain VAS, BL / 4 weeks, mean (SD)<br>Elaeagnus angustifoli: 7.04 (1.15) / 4.65 (1.84)<br>Elaeagnus angustifolia + Boswellia Thurifera: 7.03 (1.36) / 4.84 (1.96)<br>Control: 7.01 (1.25) / 5.30 (1.66); p=0.304		H/UC	H/UC	H/UC	H/UC
	Oben (2009) [RCT] [56]	Phellodendron vs placebo at 4 weeks [OV]<br>SMD -2.83 (-3.72, -1.94)<br>Phellodendron vs placebo at 4 weeks [n]<br>SMD -1.87 (-2.62, -1.12)	LAI, BL / 8 weeks, mean (SD)<br>Phellodendron [Ov]: 11.7 (1.5) / 6.3 (2.3)<br>Phellodendron [n]: 11.4 (1.2) / 7.7 (1.4)<br>Placebo [Ov]: 12.4 (1.3) / 11.8 (1.5)<br>Placebo [n]: 11.7 (2.4) / 9.9 (0.9)		L	H/UC	L	H/UC
	Piscoya (2001) [RCT] [59]	Uncaria guianensis vs placebo at 4 weeks<br>SMD -0.24 (-0.87, 0.38)	Pain, BL / 4 weeks, mean (SD*)<br>Uncaria guianensis: 4.41 (2.63) / 3.42 (1.81)<br>Placebo: 4.15 (2.98) / 3.94 (2.67)		H/UC	H/UC	L	L
Function	Liu (2018) [MA] [14]	Pine tree extract vs placebo<br>SMD -1.84 (-2.32, -1.35)		Moderate				
	Karimifar (2017) [RCT] [43]	Elaeagnus angustifoli vs control at 4 weeks<br>SMD -0.35 (-0.91, 0.22)<br>Elaeagnus angustifoli + Boswellia Thurifera vs control at 4 weeks<br>SMD -0.07 (-0.62, 0.47)	LPFI, BL / 4 weeks, mean (SD)<br>Elaeagnus angustifoli: 12.47 (2.88) / 8.32 (3.25)<br>Elaeagnus angustifolia + Boswellia Thurifera: 12.69 (3.35) / 9.09 (4.18)<br>Control: 12.84 (2.73) / 9.34 (2.66); p=0.578		H/UC	H/UC	H/UC	H/UC
Patient global	Karimifar (2017) [RCT] [43]	Elaeagnus angustifoli vs control at 4 weeks<br>SMD -0.24 (-0.80, 0.33)<br>Elaeagnus angustifoli + Boswellia Thurifera vs control at 4 weeks<br>SMD -0.64 (-1.20, -0.08)	Patient global VAS, BL / 4 weeks, mean (SD)<br>Elaeagnus angustifoli: 1.44 (0.62) / 2.38 (0.43)<br>Elaeagnus angustifolia + Boswellia Thurifera: 1.50 (0.68) / 2.17 (0.46)<br>Control: 1.79 (0.64) / 2.50 (0.57); p=0.202		H/UC	H/UC	H/UC	H/UC
ESR	Oben (2009) [RCT] [56]	Phellodendron vs placebo at 4 weeks [OV]<br>SMD -0.42 (-1.05, 0.20)<br>Phellodendron vs placebo at 4 weeks [n]<br>SMD 0.45 (-0.18, 1.08)	ESR, BL / 8 weeks, mean (SD)<br>Phellodendron [Ov]: 12.7 (0.9) / 12.9 (1.6)<br>Phellodendron [n]: 13.1 (1.2) / 13.3 (0.9)<br>Placebo [Ov]: 13.6 (2.5) / 13.6 (1.7)<br>Placebo [n]: 12.5 (1.4) / 12.8 (1.3)		L	H/UC	L	H/UC
CRP	Oben (2009) [RCT] [56]	Phellodendron vs placebo at 4 weeks [OV]<br>SMD -1.97 (-2.74, -1.21)<br>Phellodendron vs placebo at 4 weeks [n]<br>SMD -0.11 (-0.73, 0.51)	CRP, BL / 8 weeks, mean (SD)<br>Phellodendron [Ov]: 1.33 (0.2) / 0.68 (0.14)<br>Phellodendron [n]: 1.15 (0.22) / 0.64 (0.50)<br>Placebo [Ov]: 1.19 (0.26) / 1.08 (0.25)<br>Placebo [n]: 0.76 (0.19) / 0.68 (0.18)		L	H/UC	L	H/UC

* standard deviation calculation from standard error in paper

Alloc. Conc. = allocation concealment, AMSTAR2 = Assessing the Methodological Quality of Systematic Reviews, BL = baseline, Blind. Asses. = Blinded assessors, Blind. Part. = blinded participants, CI = confidence interval, CRP = C-reactive protein, ESR = erythrocyte sedimentation rate, H/UC = high / unclear risk of bias, L = low risk of bias, LAI = Lequesne Algofunctional Index, LPFI = Lequesne pain and function index, MA = meta-analysis, n = normal weight, OA = osteoarthritis, Ov = overweight, Rand. Seq. = random sequence generation, RCT = randomised controlled trial, SD = standard deviation, SMD = Standardised mean difference, VAS = visual analogue scale

Supplementary table 43 – Turmeric and OA progression, results

Table – Turmeric (OA), results and quality assessment

Outcome	Study (date) [study type]	Standardised result, SMD (95% CI) unless otherwise stated	Natural result	AMSTAR2 quality	Rand. Seq.	Alloc. Conc.	Blind. Part.	Blind. Asses.
Pain	Daily (2016) [MA] [36]		Turmeric vs control<br>meta-mean difference: -15.36 (-26.94, -3.77)	Low				

Alloc. Conc. = allocation concealment, AMSTAR2 = Assessing the Methodological Quality of Systematic Reviews, Blind. Asses. = Blinded assessors, Blind. Part. = blinded participants, CI = confidence interval, H/UC = high / unclear risk of bias, L = low risk of bias, MA = meta-analysis, OA = osteoarthritis, Rand. Seq. = random sequence generation, SMD = Standardised mean difference

Supplementary table 44 – Description of reviews of minerals and supplements in OA

Table – Minerals and supplements (OA), description of reviews

Authors (date)	Review type	Study type included	Type of OA	Exposure detail	Number of studies included	Funders
Liu (2018) [14]	MA	RCTs	Hip, knee or hand	Chondroitin<br>Glucosamine<br>L-carnitine<br>Methylsulfonylmethane	9<br>10<br>1<br>3	**Government** (NHMRC program grant, Department of education grant), **Industry** (PuraPharm postgrad scholarship), author disclosures (Flexion, Nestle, Merck)
Singh (2015) [62]	MA	RCTs	Hip, knee or hand	Chondroitin	43	**University** (University of Alabama at Birmingham, Minneapolis VA Medical Centre), **NGO** (Cochrane Complementary Medicine Field Bursary)
Gallagher (2015) [63]	SR	RCTs	Knee	Chondroitin	4	**No funding**
Percope de Andrade (2015) [38]	SR	RCTs, other reviews	Hip and knee	Chondroitin<br>Glucosamine	1 MA<br>2 MA, 1 RCT	**Not reported**, One author disclosed support from Zimmer (medical device company)
McAlindon (2014) [39]	SR	RCTs, other reviews	Knee	Chondroitin<br>Glucosamine	2 MA, 2 SR<br>2 MA, 3 SR	**Professional body** (OARSI)

MA = meta-analysis, NGO = non-governmental organisation, NHMRC = National Health and Medical Research Council, OA = osteoarthritis, OARSI = Osteoarthritis Research Society International, RCT = randomised controlled trial, SR = systematic review, VA = Veteran Affairs

Supplementary table 45 – Description of studies of minerals in OA

Table – Minerals and supplements (OA), description of included studies

Author (date) [country]	Study design	Type of OA	Inclusion criteria	Exposure detail	N	Age, mean (SD) years	N (%) female	Funders
Lei (2017) [China] [64]	RCT	Knee	Aged <80 years, ACR OA criteria, bilateral OA, degenerative primary knee OA with mild-moderate severity<br>Exclusions: Using medications or food supplements in previous 6 months, OA secondary to trauma, rheumatoid arthritis, inflammatory disorders or haemophilia, candidate for joint replacement, active and generalised inflammatory comorbidity, mal-absorption disorders, presence of cardiac, renal or hepatic failure, using steroids >10mg/day, intra-articular injections during preceding 6 months, physically or mentally compromised	1) Skimmed milk containing Lactobacillus Casei Shirota<br>p) Skimmed milk with no bacteria	1) 215<br>p) 218	1) 66.5 (5.2)<br>p) 67.2 (4.8)	1) 120 (55.8)<br>p) 121 (55.5)	**Government** (Food and Drug Administration of Hebei Province)
Neves (2011) [Brazil] [65]	RCT	Knee	Women, aged 50-65, ACR OA criteria<br>Exclusions: participation in physical activity training during past year, BMI >35, cardiovascular disease, musculoskeletal disturbances which preclude exercise, vegetarian diet, previous use of creatine, glomerular filtration rate <30, KL grade I or IV, pain scale <2cm or >8 cm, use of NSAIDs during past 3 weeks, hyaluronic acid use in last 6 months, intraarticular steroid use in last 3 months	All patients underwent exercise regime<br>1) 20g creatine for 7 days and then 5g per day for next 11 weeks. Dissolved in juice.<br>p) Dextrose dissolved in juice	1) 13<br>p) 11	1) 58 (3)<br>p) 56 (3)	1) 13 (100)<br>p) 11 (100)	**Charity** (Fundação de Amparo à Pesquisa do Estado de São Paulo), **Industry** (Ethika)
Scorei (2011) [Romania] [66]	RCT	Knee	Men / non-pregnant women, aged 40-85 years, primary knee OA (defined by the deterioration and abrasion of the articular cartilage (joint space narrowing) or by the formation of a new bone (osteophytes) at the knee joint surface)<br>Exclusions: digestion problems, fever and/or under treatment with antibiotics, taking any pain killers and/or vitamin B6	2 capsules per day with meals<br>1) 2x 28.5mg<br>2) 2x 56.5mg<br>3) 2x 113mg<br>p) fructose placebo	1) 19<br>2) 18<br>3) 17<br>p) 18	1) 68.2 (6.6)<br>2) 59.8 (8.8)<br>3) 64.8 (10)<br>p) 67.6 (5.5)	1) 12 (63.2)<br>2) 8 (44.4)<br>3) 11 (64.7)<br>p) 12 (66.7)	**Industry** (Natural Research, Ltd. (Romania))

ACR = American College of Rheumatology, BMI = body mass index, mg = milligram, N = number, NSAIDs = non-steroidal anti-inflammatory drugs, OA = osteoarthritis, RCT = randomised controlled trial, SD = standard deviation

Table – Minerals and supplements (OA), description of included studies

Author (date) [country]	Study design	Type of OA	Inclusion criteria	Exposure detail	N	Age, mean (SD) years	N (%) female	Funders
Roy (2005) [Canada] [67]	RCT	Knee	Primary OA, undergoing total knee replacement, no previous major knee surgery, not receiving workers' compensation benefits<br>Exclusions: coronary artery disease, congestive heart failure, diabetes, renal failure, previous stroke or motor loss, hypertension, inability to give consent, COPD	1) 10g creatine for 10 days before surgery and 30 days after surgery<br>p) Dextrose powder	1) 18<br>p) 19	1) 63.7 (10.0)<br>p) 63.3 (10.2)	1) 9 (50.0)<br>p) 11 (57.9)	**Industry** (Physician Services Inc), **NGO** (Canadian Foundation for Innovation), **Government** (Natural Sciences and Engineering Research Council of Canada, Hamilton Health Sciences)
Bansal (2014) [India] [68]	Single arm int.	Knee	Primary knee OA, aged >50 years, daily pain for 3 months, analgesic use at least once per week, <30 mins morning stiffness, WOMAC ≤75 in target knee, Brandt radiographic score I-II	Supplement with over 72 natural minerals in ionic form (e.g. boron, zinc, copper, selenium, magnesium, manganese, sulphur), taken twice daily for 6 months. Dose gradually increased to 40 drops	43	57.4	16 (37.2)	Not reported

COPD = chronic obstructive pulmonary disease, mg = milligram, N = number, NGO = non-governmental organisation, OA = osteoarthritis, RCT = randomised controlled trial, SD = standard deviation, WOMAC = Western Ontario and McMaster Universities Osteoarthritis Index

Supplementary table 46 – Calcium fructobate and OA progression, results

Table – Calcium fructobate (OA), results and quality assessment

Outcome	Study (date) [study type]	Standardised result, SMD (95% CI) unless otherwise stated	Natural result	AMSTAR2 quality	Rand. Seq.	Alloc. Conc.	Blind. Part.	Blind. Asses.
CRP	Scorei (2011) [RCT] [66]	28.5mg vs placebo at 2 weeks<br>SMD -9.05 (-11.26, -6.83) §<br>56.5mg vs placebo at 2 weeks<br>SMD -1.25 (-1.96, -0.53)<br>113mg vs placebo at 2 weeks<br>SMD -2.33 (-3.20, -1.46)	CRP, BL / 2 weeks, mean (SD)<br>28.5mg: 0.78 (0.2) / 0.31 (0.02) [sic]<br>56.5mg: 0.75 (0.2) / 0.55 (0.24)<br>113mg: 0.57 (0.19) / 0.47 (0.17)<br>Placebo: 0.73 (0.12) / 0.77 (0.07) [sic]		H/UC	H/UC	H/UC	H/UC
ESR	Scorei (2011) [RCT] [66]	28.5mg vs placebo at 2 weeks<br>SMD -2.62 (-3.51, -1.73)<br>56.5mg vs placebo at 2 weeks<br>SMD -2.06 (-2.87, -1.24)<br>113mg vs placebo at 2 weeks<br>SMD -2.54 (-3.44, -1.63)	ESR, BL / 2 weeks, mean (SD)<br>28.5mg: 19.5 (3.5) / 17.5 (2.7)<br>56.5mg: 18.5 (6.4) / 16.3 (5.9)<br>113mg: 18.9 (2.3) / 17.3 (3.1)<br>Placebo: 19.8 (3.2) / 27 (4.4)		H/UC	H/UC	H/UC	H/UC

§ Using the standard deviation in the published paper. Authors confirmed this was correct.

Alloc. Conc. = allocation concealment, AMSTAR2 = Assessing the Methodological Quality of Systematic Reviews, BL = baseline, Blind. Asses. = Blinded assessors, Blind. Part. = blinded participants, CI = confidence interval, CRP = C-reactive protein, ESR = erythrocyte sedimentation rate, H/UC = high / unclear risk of bias, L = low risk of bias, OA = osteoarthritis, Rand. Seq. = random sequence generation, RCT = randomised controlled trial, SD = standard deviation, SMD = Standardised mean difference,

Supplementary table 47 – Chondroitin and OA progression, results

Table – Chondroitin (OA), results and quality assessment

Outcome	Study (date) [study type]	Standardised result, SMD (95% CI) unless otherwise stated	Natural result	AMSTAR2 quality	Rand. Seq.	Alloc. Conc.	Blind. Part.	Blind. Asses.
Pain	Liu (2018) [MA] [14]	Chondroitin vs placebo<br>Short term: SMD -0.34 (-0.49, -0.19)		Moderate				
	Singh (2015) [MA] [62]	Chondroitin vs placebo<br>Short term: SMD -0.51 (-0.74, -0.28)		High				
	Gallagher (2015) [SR] [63]		Concluded that chondroitin resulted in no change in pain scores	Moderate				
	Percope de Andrade (2015) [SR] [38]		Identified one MA showing no evidence that chondroitin reduces pain	Moderate				
	McAlindon (2014) [SR] [39]		Reported large variation in pain estimates, ranging from SMD -0.13 (-0.27, 0.00) to SMD -0.75 (-0.99, -0.50)	Moderate				
Function	Liu (2018) [MA] [14]	Chondroitin vs placebo<br>Short term: SMD -0.36 (-0.58, -0.13)		Moderate				
	Singh (2015) [MA] [62]	Chondroitin vs placebo<br>Short term: SMD 0.11 (-0.47, 0.68)		High				
Structural progression	Gallagher (2015) [SR] [63]		3/4 studies reported a reduction in structural progression for chondroitin vs placebo	Moderate				

Alloc. Conc. = allocation concealment, AMSTAR2 = Assessing the Methodological Quality of Systematic Reviews, Blind. Asses. = Blinded assessors, Blind. Part. = blinded participants, CI = confidence interval, H/UC = high / unclear risk of bias, L = low risk of bias, MA = meta-analysis, OA = osteoarthritis,Rand. Seq. = random sequence generation, SMD = Standardised mean difference, SR = systematic review

Supplementary table 48 – Creatine and OA progression, results

Table – Creatine (OA), results and quality assessment

Outcome	Study (date) [study type]	Standardised result, SMD (95% CI) unless otherwise stated	Natural result	AMSTAR2 quality	Rand. Seq.	Alloc. Conc.	Blind. Part.	Blind. Asses.
Pain	Neves (2011) [RCT] [65]	Creatine vs placebo at 12 weeks<br>SMD -0.80 (-1.64, 0.03)	WOMAC pain, BL / 12 weeks, mean (SD)<br>Creatine: 5.8 (4.9) / 3.2 (2.0)<br>Placebo: 8.0 (2.9) / 5.3 (3.2)		L	L	L	L
Function	Neves (2011) [RCT] [65]	Creatine vs placebo at 12 weeks<br>SMD -0.82 (-1.66, 0.02)	WOMAC function, BL / 12 weeks, mean (SD)<br>Creatine: 15.1 (13.9) / 9.0 (7.1)<br>Placebo: 23.3 (10.8) / 15.9 (9.8)		L	L	L	L
Stiffness	Neves (2011) [RCT] [65]	Creatine vs placebo at 12 weeks<br>SMD -1.22 (-2.10, -0.34)	WOMAC stiffness, BL / 12 weeks, mean (SD)<br>Creatine: 2.7 (1.7) / 1.3 (1.1)<br>Placebo: 3.2 (1.3) / 2.7 (1.2)		L	L	L	L
Grip strength	Roy (2005) [RCT] [67]	Creatine vs placebo at 30 days<br>SMD 0.48 (-0.17, 1.14)	Grip strength at 30 days, mean (SD)<br>Creatine: 38.2 (10.4)<br>Placebo: 33.4 (9.6)		H/UC	L	L	L

Alloc. Conc. = allocation concealment, AMSTAR2 = Assessing the Methodological Quality of Systematic Reviews, BL = baseline, Blind. Asses. = Blinded assessors, Blind. Part. = blinded participants, CI = confidence interval, H/UC = high / unclear risk of bias, L = low risk of bias, OA = osteoarthritis, Rand. Seq. = random sequence generation, RCT = randomised controlled trial, SD = standard deviation, SMD = Standardised mean difference, WOMAC = Western Ontario and McMaster Universities Osteoarthritis Index

Supplementary table 49 – Glucosamine and OA progression, results

Table – Glucosamine (OA), results and quality assessment

Outcome	Study (date) [study type]	Standardised result, SMD (95% CI) unless otherwise stated	Natural result	AMSTAR2 quality	Rand. Seq.	Alloc. Conc.	Blind. Part.	Blind. Asses.
Pain	Liu (2018) [MA] [14]	Glucosamine vs placebo<br>SMD -0.28 (-0.52, -0.04)		Moderate				
	Percope de Andrade (2015) [SR] [38]		Identified one MA reporting no reduction, one Cochrane review reporting an relative risk [sic] of 0.47 (0.23, 0.72), 1 large RCT reporting no benefit	Moderate				
	McAlindon (2014) [SR] [39]		Reported large variation in pain estimates, ranging from SMD -0.17 (-0.05, -0.28) to SMD -0.47 (-0.72, -0.23)	Moderate				
Function	Liu (2018) [MA] [14]	Glucosamine vs placebo<br>SMD -0.45 (-0.73, -0.17)		Moderate				

Alloc. Conc. = allocation concealment, AMSTAR2 = Assessing the Methodological Quality of Systematic Reviews, Blind. Asses. = Blinded assessors, Blind. Part. = blinded participants, CI = confidence interval, MA = meta-analysis, OA = osteoarthritis, Rand. Seq. = random sequence generation, RCT = randomised controlled trial, SMD = Standardised mean difference, SR = systematic review

Supplementary table 50 – L-carnitine and OA progression, results

Table – L-carnitine (OA), results and quality assessment

Outcome	Study (date) [study type]	Standardised result, SMD (95% CI) unless otherwise stated	Natural result	AMSTAR2 quality	Rand. Seq.	Alloc. Conc.	Blind. Part.	Blind. Asses.
Pain	Liu (2018) [MA] [14]	L-Carnitine vs placebo<br>SMD -0.96 (-1.46, -0.46)		Moderate				
Function	Liu (2018) [MA] [14]	L-Carnitine vs placebo<br>SMD -1.15 (-1.66, -0.64)		Moderate				

Alloc. Conc. = allocation concealment, AMSTAR2 = Assessing the Methodological Quality of Systematic Reviews, Blind. Asses. = Blinded assessors, Blind. Part. = blinded participants, CI = confidence interval, H/UC = high / unclear risk of bias, L = low risk of bias, MA = meta-analysis, OA = osteoarthritis, Rand. Seq. = random sequence generation, SMD = Standardised mean difference

Supplementary table 51 – Lactobacillus Casei Shirota and OA progression, results

Table – Lactobacillus Casei Shirota (OA), results and quality assessment

Outcome	Study (date) [study type]	Standardised result, SMD (95% CI) unless otherwise stated	Natural result	AMSTAR2 quality	Rand. Seq.	Alloc. Conc.	Blind. Part.	Blind. Asses.
Pain	Lei (2017) [RCT][64]	Probiotic vs placebo at 6 months<br>SMD -0.93 (-1.12, -0.73)	WOMAC pain, BL / 6 months, mean (SD)<br>Probiotic: 10.3 (4.5) / 6.2 (3.3)<br>Placebo: 10.7 (5.3) / 9.7 (4.2); p=0.008		L	H/UC	L	L
Function	Lei (2017) [RCT][64]	Probiotic vs placebo at 6 months<br>SMD -1.51 (-1.72, -1.29)	WOMAC function, BL / 6 months, mean (SD)<br>Probiotic: 32.1 (13.4) / 16.1 (9.6)<br>Placebo: 33.2 (12.9) / 31.9 (11.3); p<0.001		L	H/UC	L	L
Stiffness	Lei (2017) [RCT][64]	Probiotic vs placebo at 6 months<br>SMD -0.49 (-0.68, -0.30)	WOMAC stiffness, BL / 6 months, mean (SD)<br>Probiotic: 1.27 (1.14) / 0.22 (0.51)<br>Placebo: 1.52 (1.31) / 0.47 (0.51); p=0.040		L	H/UC	L	L

Alloc. Conc. = allocation concealment, AMSTAR2 = Assessing the Methodological Quality of Systematic Reviews, BL = baseline, Blind. Asses. = Blinded assessors, Blind. Part. = blinded participants, CI = confidence interval, H/UC = high / unclear risk of bias, L = low risk of bias, OA = osteoarthritis, Rand. Seq. = random sequence generation, RCT = randomised controlled trial, SD = standard deviation, SMD = Standardised mean difference, WOMAC = Western Ontario and McMaster Universities Osteoarthritis Index

Supplementary table 52 – Methylsulfonylmethane and OA progression, results

Table – Methylsulfonylmethane (OA), results and quality assessment

Outcome	Study (date) [study type]	Standardised result, SMD (95% CI) unless otherwise stated	Natural result	AMSTAR2 quality	Rand. Seq.	Alloc. Conc.	Blind. Part.	Blind. Asses.
Pain	Liu (2018) [MA] [14]	Methylsulfonylmethane vs placebo<br>Short term: SMD -0.47 (-0.80, -0.14)		Moderate				
Function	Liu (2018) [MA] [14]	Methylsulfonylmethane vs placebo<br>Short term: SMD -1.10 (-1.81, -0.38)		Moderate				

Alloc. Conc. = allocation concealment, AMSTAR2 = Assessing the Methodological Quality of Systematic Reviews, Blind. Asses. = Blinded assessors, Blind. Part. = blinded participants, CI = confidence interval, H/UC = high / unclear risk of bias, L = low risk of bias, MA = meta-analysis, OA = osteoarthritis, Rand. Seq. = random sequence generation, SMD = Standardised mean difference

Supplementary table 53 – Multi-minerals and OA progression, results

Table – Multi-mineral (OA), results and quality assessment

Outcome	Study (date) [study type]	Standardised result, SMD (95% CI) unless otherwise stated	Natural result	AMSTAR2 quality	Rand. Seq.	Alloc. Conc.	Blind. Part.	Blind. Asses.
Pain	Bansal (2014) [Single arm] [68]		WOMAC pain, change from BL to 1 year, mean -4.5					
Function	Bansal (2014) [Single arm] [68]		WOMAC function, change from BL to 1 year, mean -15					
Stiffness	Bansal (2014) [Single arm] [68]		WOMAC stiffness, change from BL to 1 year, mean -1.5					

Alloc. Conc. = allocation concealment, AMSTAR2 = Assessing the Methodological Quality of Systematic Reviews, BL = baseline, Blind. Asses. = Blinded assessors, Blind. Part. = blinded participants, CI = confidence interval, OA = osteoarthritis, Rand. Seq. = random sequence generation, SMD = Standardised mean difference, WOMAC = Western Ontario and McMaster Universities Osteoarthritis Index

Supplementary table 54 – Description of reviews of vitamins in OA

Table – Vitamin D, description of reviews

Authors (date)	Review type	Study type included	Type of OA	Exposure detail	Number of studies included	Funders
Liu (2018) [14]	MA	RCTs	Hip, knee or hand	Vitamin D<br>Vitamin E	4<br>1	**Government** (NHMRC program grant, Department of education grant), **Industry** (PuraPharm postgrad scholarship), author disclosures (Flexion, Nestle, Merck)
Diao (2017) [69]	MA	RCTs	Knee	Vitamin D	4	Not reported – authors declare no conflicts of interest
Gao (2017) [70]	MA	RCTs	Knee	Vitamin D	4	None
Hussain (2017) [71]	SR	RCTs	Knee	Vitamin D	5	None
Bastick (2015) [72]	SR	Observational studies	Knee	Vitamin D	3	**Charity** (Dutch Arthritis Foundation)
Gallagher (2015) [63]	SR	RCTs	Knee	Vitamin D<br>Vitamin E	1<br>1	**No funding**

MA = meta-analysis, NHMRC = National Health and Medical Research Council, OA = osteoarthritis, RCT = randomised controlled trial, SR = systematic review

Supplementary table 55 – Description of studies of vitamins in OA

Table – Vitamins (OA), description of included studies

Author (date) [country]	Study design	Type of OA	Inclusion criteria	Exposure detail	N	Age, mean (SD) years	N (%) female	Funders
Bischoff-Ferrari (2018) [Switzerland] [73]	RCT	Knee	Age ≥60 years, underwent total knee replacement, no plans for bilateral surgery for 2 years, willingness to stop current vitamin D / calcium supplement during trial, write in German, mini-mental state ≥24<br>Exclusions: inflammatory arthritis, inability to walk at least 3m with or without walking aid	1) 2000 IU vitamin D<br>2) 800 IU vitamin D	1) 137<br>2) 136	1) 70.2 (6.8)<br>2) 70.5 (6.0)	1) 69 (50.4)<br>2) 77 (56.6)	**Government** (Swiss National Science Foundation), **Charity** (Velux Stiftung, Baugarten Foundation)
Arden (2016) [UK] [74]	RCT	Knee	Aged >50 years, ambulatory, radiographic OA, KL grade II-III, joint space width >1mm, knee pain most days of last month	1) 800 IU of vitamin D<br>p) matched placebo	1) 237<br>p) 237	1) 64 (8)<br>p) 64 (8)	1) 144 (60.8)<br>p) 145 (61.2)	**Charity** (Arthritis Research UK), **Government** (NIHR)
Jin (2016) [Australia] [75]	RCT	Knee	Aged 50-79 years, ACR criteria OA for ≥6 months, pain VAS 20-80mm, ACR functional class 1-3, physical likert good health score 0-2 (range 0-4), serum vitamin D level 12.5-60 nmol/l<br>Exclusions: Grade 3 radiographic changes (Altman & Gold) severe knee pain on standing (≥80mm on VAS), contraindication to MRI, rheumatoid arthritis, psoriatic arthritis, lupus, cancer, severe cardiac or renal impairment, hypersensitivity to vitamin D, conditions affecting oral drug absorption, anticipated knee surgery in next 2 years, history of knee trauma, taking vitamin D or investigational drug in last 30 days	1) monthly capsule of 50,000 IU vitamin D<br>p) inert placebo	1) 209<br>p) 204	1) 63.5 (6.9)<br>p) 62.9 (7.2)	1) 106 (50.7)<br>p) 102 (50.0)	**Government** (Australian National Health and Medical Research Council)
McAlindon (2013) [USA] [76]	RCT	Knee	Symptomatic knee OA, aged ≥45 years, KL grade II, ACR criteria for OA, mild pain on WOMAC<br>Exclusions: supplemental intake of vitamin D >800 IU, serum calcium >10.5 mg/dl, hypercalcuria, use of supplements or medications with purported effects on cartilage, intraarticular therapy in last 3 months, chronic oral steroid use, lymphoma, sarcoidosis, tuberculosis, hyperparathyroidism, malabsorption disorders, glomerular filtration rate <30, history of inflammatory joint disease, pregnancy, any conditions precluding MRI	1) 2000 IU vitamin D<br>p) placebo	1) 73<br>p) 73	1) 61.8 (7.7)<br>p) 63.0 (9.3)	1) 49 (67.1)<br>p) 40 (54.8)	**Government** (National Institute for Arthritis and Musculoskeletal Disorders, Office for Dietary Supplements, National Center for Research Resources)

ACR = American College of Rheumatology, AIDS = acquired immunodeficiency syndrome, HIV = human immunodeficiency virus, KL = Kellgren-Lawrence, pros = prospective, MRI = magnetic resonance imaging, N = number, NIHR = National Institutes for Health Research, OA = osteoarthritis, RCT = randomised controlled trial, SD = standard deviation, VAS = visual analogue scale, WOMAC = Western Ontario and McMaster Universities Arthritis Index

Table – Vitamins (OA), description of included studies

Author (date) [country]	Study design	Type of OA	Inclusion criteria	Exposure detail	N	Age, mean (SD) years	N (%) female	Funders
Medhi (2011) [India] [77]	RCT	Knee	Aged >40 years, knee OA<br>Exclusions: history of knee trauma, joint deformity, previous joint surgery, neurological or vascular disease affecting joints, peptic ulcer, hepatic or renal insufficiency, prior intolerance or hypersensitivity to NSAIDs, anaemia, bleeding diasthesis, unstable medical condition (e.g. diabetes, heart failure), other concomitant medication	1) Vitamin C + Vitamin E + paracetamol<br>p) paracetamol only	1) 50<br>p) 50	1) 54.8 (10.6)<br>p) 52.8 (9.2)	1) 84%<br>p) 64%	Not reported
Colker (2002) [USA] [78]	RCT	Knee	Age >35 years, knee OA diagnosed by physician, daily/almost daily pain, willing to avoid other dietary supplements<br>Exclusions: rheumatoid arthritis, anti-inflammatory medication for OA, recent use of steroids/hyaluronic acid injections, pain prescription medication, allergy to milk, cancer, HIV, AIDS, congestive heart failure	1) Refrigerated beverage, milk based, fortified with vitamins B12, C, E and iron and zinc<br>p) Refrigerated grape juice with no added vitamins	1) 16<br>p) 15	1) 51.5 (19.0)<br>p) 59.0 (21.0)	1) 11 (68.8)<br>p) 9 (60.0)	**Industry** (NuVim, Inc.)
Jonas (1996) [USA] [79]	RCT	Unspe c-ified	Clinical and radiological OA (of ≥2 joints), daily use of anti-inflammatory medication, aged >40 years, symptom duration ≥5 years, joint pain requiring NSAID use<br>Exclusions: pregnancy, morning stiffness lasting >30 minutes, palpable warmth of affected joints, severe liver disease, diabetes, gout, peptic or gastric ulcers, taking steroid medication, inability to understand questionnaire.	1) Niacinamide (vitamin B3) tablets 6x per day<br>p) Placebo tablets	1) 31<br>p) 29	1) 64 (6.4)<br>p) 65 (8.9)	1) 22 (71.0)<br>p) 17 (58.6)	**Professional body** (American Academy of Family Practice)
Flynn (1994) [USA] [80]	RCT§	Hand	ARA OA criteria, hand OA diagnosed by chronic hand pain and stiffness signs of hypertrophic changes, subchondral sclerosis, non-uniform joint space narrowing	1) Vitamin B12 + folate<br>p) folate only	26	Range: 52-82	23 (88.5)	**Charity** (Wallace Genetic Foundation), **University** (University of Missouri-Columbia)

§ Crossover design

ARA = American Rheumatism Association, AIDS = acquired immunodeficiency syndrome, HIV = human immunodeficiency virus, pros = prospective, N = number, NSAID = non-steroidal anti-inflammatory drugs, OA = osteoarthritis, RCT = randomised controlled trial, SD = standard deviation, USA = United States of America

Table – Vitamins (OA), description of included studies

Author (date) [country]	Study design	Type of OA	Inclusion criteria	Exposure detail	N	Age, mean (SD) years	N (%) female	Funders
Peregoy (2011) [USA] [81]	Pros. Cohort	Knee	Aged >40 years, KL grade >2<br>Exclusions: rheumatoid arthritis, systemic lupus erythematosus, ankylosing spondylitis, gout, disabling neuralgic disease, confined to wheelchair, mental incompetency, multivitamin use	Self-reported vitamin C supplementation	157	66.5 (8.7)	88 (56.1)	"Private funding"
Wilder (2009) [USA] [82]	Pros. Cohort	Knee	Aged ≥40 years<br>Exclusions: rheumatoid arthritis, systemic lupus erythematosus, ankylosing spondylitis, gout, disabling neurological disease, confined to wheel chair, mental incompetency	Cumulative number of years of self-reported vitamin supplement use	217	65.9 (9.6)	133 (61.3)	**Not reported**
McAlindon (1996) [USA] [83]	Pros. cohort	Knee	Radiographic OA	Self-reported vitamin D intake (food frequency questionnaire)	62	70.3	37%	**University** (Boston University Arthritis Center), **Charity** (Arthritis and Rheumatism Council)

KL = Kellgren-Lawrence, pros = prospective, N = number, OA = osteoarthritis, RCT = randomised controlled trial, SD = standard deviation, USA = United States of America

Supplementary table 56 – Multi-vitamins and OA progression, results

Table – Multi-vitamin/vitamin supplementation (OA), results and quality assessment

Outcome	Study (date) [study type]	Standardised result, SMD (95% CI) unless otherwise stated	Natural result	AMSTAR2 quality	Rand. Seq.	Alloc. Conc.	Blind. Part.	Blind. Asses.
Pain	Colker (2002) [RCT] [78]	Multivitamin vs placebo at 6 weeks<br>SMD -0.26 (-0.97, 0.44)	Pain VAS, at 6 weeks, mean (SD*)<br>Multivitamin: 3.17 (1.64)<br>Placebo: 3.77 (2.79)		H/UC	H/UC	H/UC	H/UC
QoL	Colker (2002) [RCT] [78]	Multivitamin vs placebo at 6 weeks<br>SMD -0.32 (-1.03, 0.39)	QoL KOOS, at 6 weeks, mean (SD*)<br>Multivitamin: 50.4 (22.0)<br>Placebo: 57.9 (25.17)		H/UC	H/UC	H/UC	H/UC

*SD calculated from standard error in paper

Alloc. Conc. = allocation concealment, AMSTAR2 = Assessing the Methodological Quality of Systematic Reviews, Blind. Asses. = Blinded assessors, Blind. Part. = blinded participants, CI = confidence interval, H/UC = high / unclear risk of bias, KOOS = Knee Injury and Osteoarthritis Outcome Score, L = low risk of bias, OA = osteoarthritis, QoL = quality of life, Rand. Seq. = random sequence generation, RCT = randomised controlled trial, SD = standard deviation, SMD = Standardised mean difference, VAS = visual analogue scale

Table – Multi-vitamin/vitamin supplementation cont. (OA), results and quality assessment

Outcome (outcome measure)	Study (date) [study type]	Standardised result, SMD (95% CI) unless otherwise stated	Natural result	Study Pop.	Attr.	Prog. Meas.	Outc. Meas.	Conf.	Stats.
Radiographic progression	Wilder (2009) [Pros. Obs.] [82]		Relative risk per year increase in supplementation (95% CI)<br>Unadjusted: 0.88 (0.84, 0.93)<br>Fully adjusted: 0.93 (0.87, 0.99)	L	L	M	L	M	L

Attr. = attrition, CI = confidence interval, Conf. = confounding, HR = hazard ratio, L = low risk of bias, M = moderate risk of bias, OA = osteoarthritis, Outc. Meas = outcome measurement, Prog. Meas. = prognostic factor measurement, Pros. Obs. = prospective observational, SMD = standardised mean difference, Stats. = statistical analysis, Study Pop. = study population

Supplementary table 57 – Vitamin B3 and OA progression, results

Table – Vitamin B3 (OA), results and quality assessment

Outcome	Study (date) [study type]	Standardised result, SMD (95% CI) unless otherwise stated	Natural result	AMSTAR2 quality	Rand. Seq.	Alloc. Conc.	Blind. Part.	Blind. Asses.
Pain	Jonas (1996) [RCT] [79]		AIMS pain, change from BL to week 12, mean<br>Vitamin B3: 0.10<br>Placebo: 0.82, p=0.1		L	L	L	L
ESR	Jonas (1996) [RCT] [79]		ESR, change from BL to week 12, mean<br>Vitamin B3: -6.4<br>Placebo: 3.3, p=0.004		L	L	L	L

AIMS = Arthritis Impact Measurement Scales, Alloc. Conc. = allocation concealment, AMSTAR2 = Assessing the Methodological Quality of Systematic Reviews, BL = baseline, Blind. Asses. = Blinded assessors, Blind. Part. = blinded participants, CI = confidence interval, ESR= erythrocyte sedimentation rate, H/UC = high / unclear risk of bias, L = low risk of bias, Rand. Seq. = random sequence generation, RCT = randomised controlled trial, SMD = Standardised mean difference,

Supplementary table 58 – Vitamin B12 and OA progression, results

Table – Vitamin B12 (OA), results and quality assessment

Outcome	Study (date) [study type]	Standardised result, SMD (95% CI) unless otherwise stated	Natural result	AMSTAR2 quality	Rand. Seq.	Alloc. Conc.	Blind. Part.	Blind. Asses.
Pain	Flynn (1994) [RCT] [80]		<u>Pain, mean score at end of cross-over phase</u><br>Vitamin B12: 1.0<br>Placebo: 1.0		H/UC	H/UC	L	H/UC
Tender joint count	Flynn (1994) [RCT] [80]		<u>Tender joint count, mean score at end of cross-over phase</u><br>Vitamin B12: 3.4<br>Placebo: 3.7 p=0.02		H/UC	H/UC	L	H/UC
Patient global	Flynn (1994) [RCT] [80]		<u>Patient global, mean score at end of cross-over phase</u><br>Vitamin B12: 3.1<br>Placebo: 3.5		H/UC	H/UC	L	H/UC

Alloc. Conc. = allocation concealment, AMSTAR2 = Assessing the Methodological Quality of Systematic Reviews, Blind. Asses. = Blinded assessors, Blind. Part. = blinded participants, CI = confidence interval, H/UC = high / unclear risk of bias, L = low risk of bias, OA = osteoarthritis, Rand. Seq. = random sequence generation, RCT = randomised controlled trial, SMD = Standardised mean difference

Supplementary table 59 – Vitamin C and OA progression, results

Table – Vitamin C (OA), results and quality assessment

Outcome (outcome measure)	Study (date) [study type]	Standardised result, SMD (95% CI) unless otherwise stated	Natural result	Study Pop.	Attr.	Prog. Meas.	Outc. Meas.	Conf.	Stats.
Radiographic progression	Peregoy (2009) [Pros. Obs.] [81]		Relative risk per year increase in supplementation (95% CI)<br>Unadjusted: 0.91 (0.79, 1.04)<br>Fully adjusted: 0.94 (0.79, 1.12)	L	M	M	L	L	L

Attr. = attrition, CI = confidence interval, Conf. = confounding, L = low risk of bias, M = moderate risk of bias, OA = osteoarthritis, Outc. Meas = outcome measurement, Prog. Meas. = prognostic factor measurement, Pros. Obs. = prospective observational, SMD = standardised mean difference, Stats. = statistical analysis, Study Pop. = study population

Supplementary table 60 – Vitamin C + E and OA progression, results

Table – Vitamin C + E (OA), results and quality assessment

Outcome	Study (date) [study type]	Standardised result, SMD (95% CI) unless otherwise stated	Natural result	AMSTAR2 quality	Rand. Seq.	Alloc. Conc.	Blind. Part.	Blind. Asses.
Pain	Medhi (2011) [RCT] [77]	Vitamin C + E vs placebo at 8 weeks<br>SMD -0.46 (-0.86, -0.07)	Pain VAS at 8 weeks, mean (SD)<br>Vitamin C + E: 4.12 (1.62)<br>Placebo: 4.88 (1.66)		H/UC	H/UC	H/UC	H/UC

Alloc. Conc. = allocation concealment, AMSTAR2 = Assessing the Methodological Quality of Systematic Reviews, Blind. Asses. = Blinded assessors, Blind. Part. = blinded participants, CI = confidence interval, H/UC = high / unclear risk of bias, L = low risk of bias, OA = osteoarthritis, Rand. Seq. = random sequence generation, SD = standard deviation, SMD = Standardised mean difference, VAS = visual analogue scale

Supplementary table 61 – Vitamin D and OA progression, results

Table – Vitamin D (OA), results and quality assessment

Outcome	Study (date) [study type]	Standardised result, SMD (95% CI) unless otherwise stated	Natural result	AMSTAR2 quality	Rand. Seq.	Alloc. Conc.	Blind. Part.	Blind. Asses.
Pain	Liu (2018) [MA] [14]	Vitamin D vs placebo<br>Long term: SMD -0.19 (-0.31, -0.06)		Moderate				
	Diao (2017) [MA] [69]	Vitamin D vs placebo<br>SMD -0.32 (-0.63, -0.02)		Moderate				
	Gao (2017) [MA] [70]		Vitamin D vs placebo [WOMAC]<br>MD -1.65 (-2.16, -1.14)	Low				
	Hussain (2017) [SR] [71]		1/4 studies reported a significant between group difference in pain scores	Moderate				
	Gallagher (2015) [SR] [63]		1 study reported no between group difference in pain	Moderate				
	Bischoff-Ferrari (2018) [RCT] [73]	2000 IU vitamin D vs 800 IU vitamin D at 24 months<br>SMD -0.02 (-0.25, 0.22)	WOMAC pain, BL / 24 months, mean (SD*)<br>2000 IU vitamin D: 28.9 (11.0) / 6.2 (11.9)<br>800 IU vitamin D: 28.0 (11.3) / 6.4 (11.9)		L	L	L	L
	Arden (2016) [RCT] [74]		WOMAC pain, mean (95% CI) difference<br>-0.79 (-2.31, 0.74)		L	L	L	L
	Jin (2016) [RCT] [75]	Vitamin D vs placebo at 24 months<br>SMD -0.11 (-0.31, 0.08)	WOMAC pain, BL / 24 months, mean (SD)<br>Vitamin D: 137.9 (88.8) / 87.0 (90.1)<br>Placebo: 134.7 (83.4) / 97.2 (87.5); p=0.10		L	L	L	L
	McAlindon (2013) [RCT] [76]	Vitamin D vs placebo, change from BL – 2 years<br>SMD -0.22 (-0.54, 0.11)	WOMAC pain, mean (SD*) change BL – 2 years<br>Vitamin D: -2.31 (4.05)<br>Placebo: -1.46 (3.77); p=0.17		L	L	L	L
Function	Liu (2018) [MA] [14]	Vitamin D vs placebo<br>Long term: SMD -0.36 (-0.61, -0.11)		Moderate				
	Gao (2017) [MA] [70]		Vitamin D vs placebo [WOMAC]<br>MD -1.87 (-2.58, -1.17)	Low				
	Hussain (2017) [SR] [71]		2/3 studies reported significant between group difference in function scores, final study p=0.07	Moderate				
	Bischoff-Ferrari (2018) [RCT] [73]	2000 IU vitamin D vs 800 IU vitamin D at 24 months<br>SMD 0.04 (-0.20, 0.27)	WOMAC function, BL / 24 months, mean (SD*)<br>2000 IU vitamin D: 26.3 (10.5) / 7.0 (11.0)<br>800 IU vitamin D: 25.0 (10.4) / 6.6 (11.0)		L	L	L	L
	Arden (2016) [RCT] [74]		WOMAC function, mean (95% CI) difference<br>-0.65 (-2.09, 0.79)		L	L	L	L
	Jin (2016) [RCT] [75]	Vitamin D vs placebo at 24 months<br>SMD -0.18 (-0.37, 0.02)	WOMAC function, BL / 24 months, mean (SD)<br>Vitamin D: 487.9 (318.1) / 306.4 (303.7)<br>Placebo: 467.6 (292.8) / 361.8 (322.8); p=0.008		L	L	L	L
	McAlindon (2013) [RCT] [76]	Vitamin D vs placebo, change from BL – 2 years<br>SMD -0.29 (-0.62, 0.04)	WOMAC function, mean (SD*) change BL – 2 years<br>Vitamin D: -6.97 (12.16)<br>Placebo: -3.82 (9.33); p=0.07		L	L	L	L

* SD calculated from 95% CI in paper; Alloc. Conc. = allocation concealment, AMSTAR2 = Assessing the Methodological Quality of Systematic Reviews, BL = baseline, Blind. Asses. = Blinded assessors, Blind. Part. = blinded participants, CI = confidence interval, H/UC = high / unclear risk of bias, L = low risk of bias, MA = meta-analysis, MD = mean difference, OA = osteoarthritis, Rand. Seq. = random sequence generation, RCT = randomised controlled trial, SD = standard deviation, SMD = Standardised mean difference, SR = systematic review, WOMAC = Western Ontario and McMaster Universities Osteoarthritis Index

Table [cont.] – Vitamin D (OA), results and quality assessment

Outcome	Study (date) [study type]	Standardised result, SMD (95% CI) unless otherwise stated	Natural result	AMSTAR2 quality	Rand. Seq.	Alloc. Conc.	Blind. Part.	Blind. Asses.
Stiffness	Gao (2017) [MA] [70]		Vitamin D vs placebo [WOMAC]<br>MD 0.03 (-0.17, 0.24)	Low				
	Arden (2016) [RCT] [74]		WOMAC stiffness, mean (95% CI) difference<br>-1.52 (-3.24, 0.21)		L	L	L	L
	Jin (2016) [RCT] [75]	Vitamin D vs placebo at 24 months<br>SMD -0.11 (-0.30, 0.09)	WOMAC stiffness, BL / 24 months, mean (SD)<br>Vitamin D: 61.5 (41.5) / 41.1 (44.1)<br>Placebo: 61.7 (40.1) / 45.7 (41.1); p=0.31		L	L	L	L
Structural progression	Bastick (2015) [SR] [72]		Moderate evidence that vitamin D is inversely associated with progression of knee OA (3/6 studies)	Moderate				
	Gallagher (2015) [SR] [63]		1 study reported no between group difference in structural progression	Moderate				

Alloc. Conc. = allocation concealment, AMSTAR2 = Assessing the Methodological Quality of Systematic Reviews, BL = baseline, Blind. Asses. = Blinded assessors, Blind. Part. = blinded participants, CI = confidence interval, H/UC = high / unclear risk of bias, L = low risk of bias, MA = meta-analysis, OA = osteoarthritis,Rand. Seq. = random sequence generation, RCT = randomised controlled trial, SMD = Standardised mean difference, SR = systematic review, WOMAC = Western Ontario and McMaster Universities Osteoarthritis Index

Table – Vitamin D (OA), results and quality assessment from observational studies

Outcome (outcome measure)	Study (date) [study type]	Standardised result, SMD (95% CI) unless otherwise stated	Natural result	Study Pop.	Attr.	Prog. Meas.	Outc. Meas.	Conf.	Stats.
Radiographic progression	McAlindon (1996) [Pros. Obs.] [83]		Radiographic progression, OR (95% CI)<br>Highest tertile of vitamin D intake: ref<br>Middle tertile: 2.99 (1.06, 8.49)<br>Lowest tertile: 4.05 (1.40, 11.6)	M	na.	M	L	L	L

Attr. = attrition, CI = confidence interval, Conf. = confounding, HR = hazard ratio, L = low risk of bias, M = moderate risk of bias, OA = osteoarthritis, OR = odds ratio, Outc. Meas = outcome measurement, Prog. Meas. = prognostic factor measurement, Pros. Obs. = prospective observational, SMD = standardised mean difference Stats. = statistical analysis, Study Pop. = study population

Supplementary table 62 – Vitamin E and OA progression, results

Table – Vitamin E (OA), results and quality assessment

Outcome	Study (date) [study type]	Standardised result, SMD (95% CI) unless otherwise stated	Natural result	AMSTAR2 quality	Rand. Seq.	Alloc. Conc.	Blind. Part.	Blind. Asses.
Pain	Liu (2018) [MA] [14]	Vitamin E vs placebo<br>SMD 0.01 (-0.44, 0.45)		Moderate				
	Gallagher (2015) [SR] [63]		1 study reported no between group difference in pain	Moderate				
Function	Liu (2018) [MA] [14]	Vitamin E vs placebo<br>SMD -0.10 (-0.55, 0.35)		Moderate				
Structural progression	Gallagher (2015) [SR] [63]		1 study reported no between group difference in structural progression	Moderate				

Alloc. Conc. = allocation concealment, AMSTAR2 = Assessing the Methodological Quality of Systematic Reviews, Blind. Asses. = Blinded assessors, Blind. Part. = blinded participants, CI = confidence interval, H/UC = high / unclear risk of bias, L = low risk of bias, MA = meta-analysis, OA = osteoarthritis, Rand. Seq. = random sequence generation, SMD = Standardised mean difference, SR = systematic review

Supplementary table 63 – Description of reviews of animal products in RA

Table – Animal products (RA), description of reviews

Authors (date)	Review type	Study type included	Exposure detail	Number of studies included	Funders
Gioxari (2018)[84]	MA	RCTs	Omega 3	20	**Government** (State Scholarship Foundation)
Senftleber (2017)[15]	MA	RCTs	Marine oil supplements	32	**Charity** (Oak Foundation [indirectly funded]), **Government** (National Institute of Arthritis and Musculoskeletal and Skin Diseases, NIH [individual fellowship of an author])
Cramp (2013)[85]	MA	RCTs	Omega 3	1	**Charity** (Arthritis Research UK)
Abdulrazaq (2017)[86]	SR	RCTs	Omega 3	18	Not reporting, authors declare no conflicts of interest

MA = meta-analysis, NIH = National Institutes of Health, RA = rheumatoid arthritis, RCT = randomised controlled trial, SR = systematic review

Supplementary table 64 – Description of studies of animal products in RA

Table – Animal products (RA), description of included studies

Author (date) [country]	Study design	Inclusion criteria	Exposure detail	N	Age, mean (SD) years	N (%) female	Funders
Lindqvist (2018) [Sweden][87]	RCT §	Aged 25-65 years, >2 years symptom duration, DAS28 >3.0	1) One meal a day replaced by intervention meal, with included blue mussels from Denmark<br>p) Same as intervention but with meat instead of mussels	23	Median (IQR) 55 (46, 63)	23 (100)	**Charity** (Hakansson Foundation), **Government** (Swedish government under the ALF-funds)
Fu (2015) [China][88]	RCT	ACR criteria (no reference), symptom duration ≥6 months, ≥4 of the following: ≥4 swollen joints, ≥6 tender joints, ESR >28 mm/hr, morning stiffness last ≥45 mins, CRP >2 mg/dL	1) Capsules of hard-shelled mussel extract<br>p) Placebo capsules	1) 18<br>p) 24	1) 56.6 (2.8)<br>p) 58.3 (2.18)	1) 12 (66.7)<br>p) 18 (75.0)	**Government** (National Natural Science Foundation of China, Ningbo Natural Science Foundation, PhD. Programs Foundation of Ministry of Education of China, National Basic Research Program of China)
Rajaei (2015) [Iran][89]	RCT	1987 RA criteria, RA diagnosed by two rheumatologists<br>Exclusions: diagnosis >6 months, bone deformities, severe concomitant diseases (e.g. metabolic, gastrointestinal), functional class IV, use of omega 3 supplementation, digestive intolerance, severe infections, AST, ALT or creative >1.5x ULN, bilirubin >1.8mg/dL	1) 2 omega 3 capsules 3x per day<br>p) placebo tablets	1) 30<br>p) 30	Not reported	1) 25 (83.3)<br>p) 24 (80.0)	**University** (Ahvaz Jundishapur University of Medical Sciences)
Reed (2014) [Canada][90]	RCT	1987 RA, Functional Class I-III, aged 18-85, ≥3 swollen joints, ≥6 tender joints, ESR ≥28, stable DMARDs<br>Exclusions: investigation drugs within one month of BL, already taking borage seed / fish oil ≥2000mg/d for 2 months before BL, intraarticular steroids within 6 months of BL, ALT/AST >1.5x ULN, bilirubin >1.8mg/dL	1) Fish oil<br>2) Borage seeds<br>3) Fish oil + Borage seeds	1) 53<br>2) 52<br>3) 45	1) 57.3 (12.3)<br>2) 60.3 (9.2)<br>3) 60.5 (13.0)	1) 46 (86.8)<br>2) 40 (76.9)<br>3) 36 (80.0)	Not reported, authors declare no conflicts of interest

§ Cross-over design
ACR = American College of Rheumatology, ALT = alanine aminotransferase, AST = Aspartate transaminase, BL = baseline, CRP = C-reactive protein, DAS28 = Disease Activity Score (28), DMARD = disease modifying anti-rheumatic drug, ESR = erythrocyte sedimentation rate, IQR = interquartile range, N = number, RA = rheumatoid arthritis, RCT = randomised controlled trial, SD = standard deviation, ULN = upper limit of normal

Table – Animal products (RA), description of included studies

Author (date) [country]	Study design	Inclusion criteria	Exposure detail	N	Age, mean (SD) years	N (%) female	Funders
Arborelius (1999) [Finland][91]	RCT	1987 RA criteria, aged >16 years, active disease (at least one of: ≥9 tender joints, ≥6 swollen joints, CRP ≥11)<br>Exclusions: Pregnancy, wheel chair bound, functional class IV, participation in another clinical trial in which non-registered drugs are used, allergy to orange juice, glutein induced enteropathy	1) Collagen from pig skins – turned into powder and taken with orange juice<br>p) Placebo made from wheat – powder almost identical to collagen	36§	57.0 (10)	26 (72.2)	**University** (Helsinki University Central Hospital), **Industry** (Extraco AB)
Skoldstam (1992) [Sweden][92]	RCT	1987 RA criteria, Functional Class II-III, Stable disease history and treatment for preceding three months	1) Fish oil capsules<br>p) placebo capsules	1) 22<br>p) 21	1) 58 (range 40-73)<br>p) 55 (range 28-70)	1) 18 (81.8)<br>p) 14 (66.6)	**Government** (Swedish Council for Planning and Coordination of Research)
Tulleken (1990) [The Netherlands][93]	RCT	1958 RA criteria, stable treatment for 3 months	1) 4 fish oil capsules 3x per day<br>p) Coconut oil with fish flavouring	1) 13<br>p) 14	1) 52 (range: 29, 66)<br>p) 58 (range: 43, 68)	1) 12 (92.3)<br>p) 12 (85.7)	**NGO** (Dutch League Against Rheumatism)
van der Tempel (1990) [The Netherlands][94]	RCT §	Classical or definite RA	1) Fish oil capsules<br>p) Coconut oil with fish flavouring	16	53	9 (56.3)	**NGO** (Dutch League Against Rheumatism)
Cleland (1988) [Australia][95]	RCT	Classical or definite RA	1) Fish oil capsules<br>p) Olive oil	1) 23<br>p) 23	1) 51 (range: 22-71)<br>p) 50 (25-74)	1) 16 (69.5)<br>p) 16 (69.5)	**Government** (National Health and Medical Research Council of Australia), **Charity** (Arthritis Fund of Australia, Royal Adelaide Hospital Research Fund, Queen Elizabeth Hospital Research Foundation)

§ Cross-over design
CRP = C-reactive protein, N = number, NGO = non-governmental organisation, RA = rheumatoid arthritis, RCT = randomised controlled trial, SD = standard deviation

Table – Animal products (RA), description of included studies

Author (date) [country]	Study design	Inclusion criteria	Exposure detail	N	Age, mean (SD) years	N (%) female	Funders
Magaro (1988) [Italy][96]	RCT	Classical or definite RA, active disease: morning stiffness ≥30 mins, ≥6 tender joints, ≥3 swollen joints, ≥30mm/h ESR<br>Exclusion: systemic steroids or immunosuppressive drugs in three months before BL	1) Max EPA – high in unsaturated fatty acids<br>p) diet high in saturated fatty acids	1) 6<br>p) 6	1) 36<br>(range 20-50)<br>p) 37<br>(range 20 55)	1) 6 (100)<br>p) 6 (100	Not reported
Kremer (1987) [USA][97]	RCT §	Classical or definite RA, Functional class I-III, had at least three of the following four criteria: morning stiffness of at least 30 minutes duration; ≥6 tender joints; ≥3 swollen joints; ≥28 ESR	1) Daily fish oil supplements<br>p) Placebo supplement	33	56.8<br>(range 23-74)	25 (75.8)	**Government** (NIH, Research Service of the Veterans Administration)
Cleland (2006) [Australia][98]	NRT	Aged >18 years, 1987 RA criteria, symptoms <12 months	1) Bottle fish oil juice / capsules depending on preference<br>p) Those not taking fish oil regularly	1) 18<br>p) 15	1) 61.8 (9.9)<br>p) 51.1 (15.9)	1) 67%<br>p) 76%	**Government** (National Health and Medical Research Council of Australia)

§ Cross-over design

BL = baseline, CRP = C-reactive protein, EPA = eicosapentaenoic acid, ESR = erythrocyte sedimentation rate, N = number, NIH = National Institutes of Health, NRT = non-randomised trial, RA = rheumatoid arthritis, RCT = randomised controlled trial, SD = standard deviation

Supplementary table 65 – Collagen and RA progression, results

Table – Collagen (RA), results and quality assessment

Outcome	Study (date) [study type]	Standardised result, SMD (95% CI) unless otherwise stated	Natural result	AMSTAR2 quality	Rand. Seq.	Alloc. Conc.	Blind. Part.	Blind. Asses.
Pain	Arborelius (1999) [RCT][91]		Pain VAS, mean difference between intervention and control<br>-0.69, p = NS		L	H/UC	L	H/UC
Function	Arborelius (1999) [RCT][91]		HAQ (0-24), mean difference between intervention and control<br>-3.88, p=NS		L	H/UC	L	H/UC
Tender joints	Arborelius (1999) [RCT][91]		Ritchie Index, mean difference between intervention and control<br>1.51, p=NS		L	H/UC	L	H/UC
Swollen joints	Arborelius (1999) [RCT][91]		Swollen joint count (54), mean difference between intervention and control<br>-1.6, p=NS		L	H/UC	L	H/UC
Disease activity	Arborelius (1999) [RCT][91]		DAS, mean difference between intervention and control<br>-0.54, p=NS		L	H/UC	L	H/UC
Acute Phase Reactants	Arborelius (1999) [RCT][91]		CRP / ESR, mean difference between intervention and control<br>1.48, p=NS / -3.65, p=NS		L	H/UC	L	H/UC
Patient global	Arborelius (1999) [RCT][91]		Patient global VAS, mean difference between intervention and control<br>-1.1, p=NS		L	H/UC	L	H/UC

Alloc. Conc. = allocation concealment, AMSTAR2 = Assessing the Methodological Quality of Systematic Reviews, Blind. Asses. = Blinded assessors, Blind. Part. = blinded participants, CI = confidence interval, CRP = C-reactive protein, DAS = Disease Activity Score, ESR = erythrocyte sedimentation rate, H/UC = high / unclear risk of bias, HAQ = Health Assessment Questionnaire, L = low risk of bias, NS = non-significant, RA = rheumatoid arthritis, Rand. Seq. = random sequence generation, RCT = randomised controlled trial, SMD = Standardised mean difference, VAS = visual analogue scale

Supplementary table 66 – Fish oil / omega 3 and RA progression, results

Table – Fish oils / omega 3 (RA), results and quality assessment

Outcome	Study (date) [study type]	Standardised result, SMD (95% CI) unless otherwise stated	Natural result	AMSTAR2 quality	Rand. Seq.	Alloc. Conc.	Blind. Part.	Blind. Asses.
Pain	Gioxari (2018) [MA][84]	Omega 3 vs placebo<br>SMD -0.32 (-0.59, -0.05)		Moderate				
	Senftleber (2017) [MA][15]	Marine oil supplements vs placebo<br>SMD -0.21 (-0.42, -0.00)		High				
	Abdulrazaq (2017) [SR][86]	10/18 studies reported reduction a in pain from omega-3. Of these 10, only 4 were compared to placebo and 6 were comparisons to baseline scores		Moderate				
	Rajaei (2015) [RCT][89]		Pain VAS, BL / 12 weeks, mean<br>Omega 3: 9 / 4<br>Placebo: 8 / 8		H/UC	H/UC	L	H/UC
	Skoldstam (1992) [RCT][92]	Fish oil vs placebo, change BL-6 months<br>SMD -0.21 (-0.81, 0.39)	Pain VAS, BL-6 months, mean (SD §)<br>Fish oil: 0.02 (0.66)<br>Placebo: 0.17 (0.78)		H/UC	H/UC	L	L
	Tulleken (1990) [RCT][93]	Fish oil vs placebo<br>SMD -0.46 (-1.22, 0.31)	Pain VAS, BL / 3 months, mean (SD †)<br>Fish oil: 3.7 (1.7) / 3.1 (2.2)<br>Placebo: 4.6 (1.9) / 4.1 (2.2)		H/UC	H/UC	L	H/UC
	van der Tempel (1990) [RCT][94]	Fish oil vs placebo at 12 weeks<br>SMD -0.53 (-1.24, 0.17)	Pain VAS, 12 weeks, mean (SD §)<br>Fish oil: 2.7 (2.0)<br>Placebo: 4 (2.8)		H/UC	H/UC	L	L
	Cleland (1988) [RCT][95]	Fish oil vs placebo at 3 months<br>SMD -0.02 (-0.60, 0.56)	Pain VAS, BL / 3 months, mean (SD)<br>Fish oil: 9.6 (5.8) / 7.0 (4.6)<br>Placebo: 9.8 (4.6) / 7.1 (5.1)		H/UC	H/UC	L	H/UC
	Kremer (1987) [RCT][97]	Fish oil vs placebo, change BL-14 weeks<br>SMD -0.28 (-0.77, 0.20)	Pain, change BL – 14 weeks, mean (SD*)<br>Fish oil: -0.21 (0.91)<br>Placebo: 0.0 (0.53)		H/UC	H/UC	L	L
	Bespoke meta-analysis [92-95;97]	Fish oil vs placebo<br>SMD -0.27 (-0.54, 0.00), I^2 0%						
Function	Gioxari (2018) [MA][84]	Omega 3 vs placebo<br>SMD -0.26 (-0.46, -0.06)		Moderate				
	Senftleber (2017) [MA][15]	Marine oil supplements vs placebo<br>SMD 0.05 (-0.11, 0.21)		High				
	Skoldstam (1992) [RCT][92]	Fish oil vs placebo, change BL-6 months<br>SMD -0.35 (-0.95, 0.26)	HAQ, BL-6 months, mean (SD §)<br>Fish oil: -0.07 (0.42)<br>Placebo: 0.06 (0.32)		H/UC	H/UC	L	L
	Cleland (2006) [NRT][98]	Fish oil vs control at 3 years<br>SMD -0.86 (-1.61, -0.12)	mHAQ, BL / 3 years, mean (SD)<br>Fish oil: 6.6 (3.2) / 1.2 (1.7)<br>Control: 7.1 (4.2) / 3.3 (3.2)					

* Calculated from 95% CI in paper § Calculated from standard error in paper † Mean (SD) calculated from median (range) using published formula [61] Alloc. Conc. = allocation concealment, AMSTAR2 = Assessing the Methodological Quality of Systematic Reviews, BL = baseline, Blind. Asses. = Blinded assessors, Blind. Part. = blinded participants, CI = confidence interval, H/UC = high / unclear risk of bias, HAQ = Health Assessment Questionnaire, L = low risk of bias, MA = meta-analysis, mHAQ = modified Health Assessment Questionnaire, NRT = non-randomised trial, RA = rheumatoid arthritis, Rand. Seq. = random sequence generation, RCT = randomised controlled trial, SD = standard deviation, SMD = Standardised mean difference, VAS = visual analogue scale

Table – Fish oils / omega 3 (RA), results and quality assessment

Outcome	Study (date) [study type]	Standardised result, SMD (95% CI) unless otherwise stated	Natural result	AMSTAR2 quality	Rand. Seq.	Alloc. Conc.	Blind. Part.	Blind. Asses.
Disease activity	Rajaei (2015) [RCT][89]		DAS28, ≤3.2 / 3.2-5.1 / >5.1 at 12 weeks<br>Omega 3: 20 / 5 / 0<br>Placebo: 0 / 24 / 0;		H/UC	H/UC	L	H/UC
	Reed (2014) [RCT][90]	Fish oil vs borage seed, change from BL to 18 months<br>SMD 0.12 (-0.26, 0.50)	DAS28, change from BL to 18 months, mean (SD*)<br>Fish oil: -1.28 (2.25)<br>Borage seed: -1.53 (1.91)<br>Fish oil + Borage seed: -1.45 (1.92)		H/UC	H/UC	L	L
	Cleland (2006) [NRT][98]	Fish oil vs control at 3 years<br>SMD -1.27 (-2.06, -0.49)	DAS28, BL / 3 years, mean (SD)<br>Fish oil: 5.0 (1.5) / 2.1 (0.9)<br>Control: 5.7 (0.9) / 3.3 (1.0)					
Tender joints	Gioxari (2018) [MA][84]	Omega 3 vs placebo<br>SMD -0.24 (-0.39, -0.095)		Moderate				
	Rajaei (2015) [RCT][89]		Tender joint count, BL / 12 weeks, mean<br>Omega 3: 21 / 5<br>Placebo: 24 / 20; p<0.05		H/UC	H/UC	L	H/UC
	Skoldstam (1992) [RCT][92]	Fish oil vs placebo, change BL-6 months<br>SMD -0.02 (-0.62, 0.58)	Ritchie Index, BL-6 months, mean (SD §)<br>Fish oil: -2.6 (5.2)<br>Placebo: -2.5 (6.0)		H/UC	H/UC	L	L
	Tulleken (1990) [RCT][93]	Fish oil vs placebo<br>SMD 0.11 (-0.64, 0.87)	Ritchie Index, BL / 3 months, mean (SD †)<br>Fish oil: 22 (13.7) / 15.3 (14.6)<br>Placebo: 15.8 (6.4) / 14 (7.3)		H/UC	H/UC	L	H/UC
	Cleland (1988) [RCT][95]		Tender joint count, BL / 3 months, mean (range)<br>Fish oil: 13 (4-41) / 9.5 (1-31)<br>Placebo: 13 (3-36) / 12 (0-41)		H/UC	H/UC	L	H/UC
	Magaro (1988) [RCT][96]	Max EPA vs placebo at 30 days<br>SMD -1.32 (-2.60, -0.05)	Ritchie index, 30 days, mean (SD §)<br>Max EPA: 10.6 (8.5)<br>Control: 21.4 (7.8)		H/UC	H/UC	H/UC	H/UC
	Kremer (1987) [RCT][97]	Fish oil vs placebo, change BL-14 weeks<br>SMD -0.81 (-1.32, -0.31)	Tender joint count, change BL – 14 weeks, mean (SD*)<br>Fish oil: -3.5 (5.0)<br>Placebo: 0.01 (3.5)		H/UC	H/UC	L	L
	Cleland (2006) [NRT][98]	Fish oil vs control at 3 years<br>SMD -1.06 (-1.82, -0.29)	Tender joint count, BL / 3 years, mean (SD)<br>Fish oil: 6.4 (6.2) / 0.7 (1.1)<br>Control: 8.8 (3.6) / 3.5 (3.9)					
	Bespoke MA[92;93;96;97]	Fish oil vs placebo<br>SMD -0.42 (-1.01, 0.16), I^2 62.3%						

* Calculated from 95% CI in paper, § Calculated from standard error in paper, † Mean (SD) calculated from median (range) using published formula [61] Alloc. Conc. = allocation concealment, AMSTAR2 = Assessing the Methodological Quality of Systematic Reviews, BL = baseline, Blind. Asses. = Blinded assessors, Blind. Part. = blinded participants, CI = confidence interval, DAS28 = Disease activity score 28, EPA = eicosapentaenoic acid, H/UC = high / unclear risk of bias, L = low risk of bias, MA = meta-analysis, NRT = non-randomised trial, RA = rheumatoid arthritis, Rand. Seq. = random sequence generation, RCT = randomised controlled trial, SD = standard deviation, SMD = Standardised mean difference

Table – Fish oils / omega 3 (RA), results and quality assessment

Outcome	Study (date) [study type]	Standardised result, SMD (95% CI) unless otherwise stated	Natural result	AMSTAR2 quality	Rand. Seq.	Alloc. Conc.	Blind. Part.	Blind. Asses.
Swollen joints	Gioxari (2018) [MA][84]	<u>Omega 3 vs placebo</u><br>SMD -0.08 (-0.23, 0.07)		Moderate				
	Rajaei (2015) [RCT][89]		<u>Swollen joint count, BL / 12 weeks, mean</u><br>Omega 3: 10 / 3<br>Placebo: 7 / 5; p<0.05		H/UC	H/UC	L	H/UC
	Tulleken (1990) [RCT][93]	<u>Fish oil vs placebo</u><br>SMD -0.11 (-0.87, 0.64)	<u>Swollen joint count, BL / 3 months, mean (SD †)</u><br>Fish oil: 9 (7.2) / 5.8 (4.5)<br>Placebo: 6.3 (3.2) / 6.3 (4.4)		H/UC	H/UC	L	H/UC
	van der Tempel (1990) [RCT][94]	<u>Fish oil vs placebo at 12 weeks</u><br>SMD -0.67 (-1.38, 0.04)	<u>Joint swelling, 12 weeks, mean (SD §)</u><br>Fish oil: 2 (4)<br>Placebo: 8 (12)		H/UC	H/UC	L	L
	Cleland (1988) [RCT][95]		<u>Swollen joint count, BL / 3 months, mean (range)</u><br>Fish oil: 3.5 (0-12) / 3.6 (0-9)<br>Placebo: 3.8 (0-8) / 3.5 (0-12)		H/UC	H/UC	L	H/UC
	Kremer (1987) [RCT][97]	<u>Fish oil vs placebo, change BL-14 weeks</u><br>SMD -0.41 (-0.90, 0.08)	<u>Swollen joint count, change BL – 14 weeks, mean (SD*)</u><br>Fish oil: -2.8 (4.4)<br>Placebo: -1.0 (4.4)		H/UC	H/UC	L	L
	Cleland (2006) [NRT][98]	<u>Fish oil vs control at 3 years</u><br>SMD 0.42 (-0.30, 1.14)	<u>Swollen joint count, BL / 3 years, mean (SD)</u><br>Fish oil: 5.4 (5.5) / 0.9 (1.8)<br>Control: 6.9 (4.7) / 0.3 (0.6)					
Inflammation	Senftleber (2017) [MA][15]	<u>Marine oil supplements vs placebo</u><br>SMD -0.20 (-0.42, 0.03)		High				

* Calculated from 95% CI in paper
§ Calculated from standard error in paper
† Mean (SD) calculated from median (range) using published formula [61]
Alloc. Conc. = allocation concealment, AMSTAR2 = Assessing the Methodological Quality of Systematic Reviews, BL = baseline, Blind. Asses. = Blinded assessors, Blind. Part. = blinded participants, CI = confidence interval, H/UC = high / unclear risk of bias, L = low risk of bias, MA = meta-analysis, NRT = non-randomised trial, RA = rheumatoid arthritis, Rand. Seq. = random sequence generation, RCT = randomised controlled trial, SD = standard deviation, SMD = Standardised mean difference

Table – Fish oils / omega 3 (RA), results and quality assessment

Outcome	Study (date) [study type]	Standardised result, SMD (95% CI) unless otherwise stated	Natural result	AMSTAR2 quality	Rand. Seq.	Alloc. Conc.	Blind. Part.	Blind. Asses.
Morning stiffness	Gioxari (2018) [MA][84]	Omega 3 vs placebo<br>SMD -0.42 (-0.68, -0.16)		Moderate				
	Rajaei (2015) [RCT][89]		Morning stiffness, BL / 12 weeks, mean<br>Omega 3: 128 / 40<br>Placebo: 116 / 94; p<0.05		H/UC	H/UC	L	H/UC
	Tulleken (1990) [RCT][93]	Fish oil vs placebo<br>SMD -0.66 (-1.44, 0.12)	Morning stiffness, BL / 3 months, mean (SD †)<br>Fish oil: 45 (17.9) / 45 (35.9)<br>Placebo: 52.5 (35.1) / 75 (52.7)		H/UC	H/UC	L	H/UC
	van der Tempel (1990) [RCT][94]	Fish oil vs placebo at 12 weeks<br>SMD -0.89 (-1.62, -0.16)	Morning stiffness, 12 weeks, mean (SD §)<br>Fish oil: 15 (20)<br>Placebo: 50 (52)		H/UC	H/UC	L	L
	Cleland (1988) [RCT][95]		Morning stiffness, BL / 3 months, mean (range)<br>Fish oil: 48 (0-240) / 25 (0-120)<br>Placebo: 63 (5-240) / 38 (0-180)		H/UC	H/UC	L	H/UC
	Magaro (1988) [RCT][96]	Max EPA vs placebo at 30 days<br>SMD -0.61 (-1.77, 0.55)	Morning stiffness, 30 days, mean (SD §)<br>Max EPA: 22 (20.7)<br>Control: 36 (24.9)		H/UC	H/UC	H/UC	H/UC
	Kremer (1987) [RCT][97]	Fish oil vs placebo, change BL-14 weeks<br>SMD -0.42 (-0.90, 0.07)	Morning stiffness (mins), change BL – 14 weeks, mean (SD*)<br>Fish oil: -5.9 (48.9)<br>Placebo: 49.4 (182.0)		H/UC	H/UC	L	L
	Bespoke MA [93;94;96;97]	Fish oil vs placebo<br>SMD -0.59 (-0.93, -0.24), I^2 0%						

* Calculated from 95% CI in paper, § Calculated from standard error in paper, † Mean (SD) calculated from median (range) using published formula [61]

Alloc. Conc. = allocation concealment, AMSTAR2 = Assessing the Methodological Quality of Systematic Reviews, BL = baseline, Blind. Asses. = Blinded assessors, Blind. Part. = blinded participants, CI = confidence interval, EPA = eicosapentaenoic acid, ESR = erythrocyte sedimentation rate, H/UC = high / unclear risk of bias, L = low risk of bias, MA = meta-analysis, RA = rheumatoid arthritis, Rand. Seq. = random sequence generation, RCT = randomised controlled trial, SD = standard deviation, SMD = Standardised mean difference

Table – Fish oils / omega 3 (RA), results and quality assessment

Outcome	Study (date) [study type]	Standardised result, SMD (95% CI) unless otherwise stated	Natural result	AMSTAR2 quality	Rand. Seq.	Alloc. Conc.	Blind. Part.	Blind. Asses.
Fatigue	Gioxari (2018) [MA][84]	Omega 3 vs placebo<br>SMD -0.10 (-0.55, 0.34)		Moderate				
	Cramp (2013) [MA][85]	Omega 3 vs placebo<br>SMD 0.93 (0.47, 1.39) in favour of control						
	Kremer (1987) [RCT][97]	Fish oil vs placebo, change BL-14 weeks<br>SMD 0.57 (0.08, 1.06)	Time to fatigue (mins), change BL – 14 weeks, mean (SD*)<br>Fish oil: 176.8 (274.9)<br>Placebo: 8.4 (314.5)		H/UC	H/UC	L	L
Patient global	Skoldstam (1992) [RCT][92]	Fish oil vs placebo, change BL-6 months<br>SMD -0.53 (-1.13, 0.08)	Patient global, change BL-6 months, mean (SD §)<br>Fish oil: 0.01 (0.66)<br>Placebo: 0.40 (0.82)		H/UC	H/UC	L	L
	Kremer (1987) [RCT][97]	Fish oil vs placebo, change BL-14 weeks<br>SMD -0.19 (-0.67, 0.30)	Patient global, change BL – 14 weeks, mean (SD*)<br>Fish oil: -0.11 (0.70)<br>Placebo: 0.0 (0.47)		H/UC	H/UC	L	L

* Calculated from 95% CI in paper, § Calculated from standard error in paper, † Mean (SD) calculated from median (range) using published formula [61]

Alloc. Conc. = allocation concealment, AMSTAR2 = Assessing the Methodological Quality of Systematic Reviews, BL = baseline, Blind. Asses. = Blinded assessors, Blind. Part. = blinded participants, C-reactive protein, CI = confidence interval, H/UC = high / unclear risk of bias, L = low risk of bias, MA = meta-analysis, RA = rheumatoid arthritis, Rand. Seq. = random sequence generation, RCT = randomised controlled trial, SD = standard deviation, SMD = Standardised mean difference

Table – Fish oils / omega 3 (RA), results and quality assessment

Outcome	Study (date) [study type]	Standardised result, SMD (95% CI) unless otherwise stated	Natural result	AMSTAR2 quality	Rand. Seq.	Alloc. Conc.	Blind. Part.	Blind. Asses.
CRP	Gioxari (2018) [MA][84]	Omega 3 vs placebo<br>SMD 0.44 (-0.13, 1.00)		Moderate				
	Skoldstam (1992) [RCT][92]	Fish oil vs placebo, change BL-6 months<br>SMD -0.17 (-0.77, 0.43)	CRP, change BL-6 months, mean (SD §)<br>Fish oil: 7 (18.8)<br>Placebo: 12 (36.7)		H/UC	H/UC	L	L
	Tulleken (1990) [RCT][93]	Fish oil vs placebo<br>SMD -0.63 (-1.41, 0.14)	CRP, BL / 3 months, mean (SD †)<br>Fish oil: 18.3 (15.2) / 20.3 (15.2)<br>Placebo: 42 (31.0) / 35 (28.7)		H/UC	H/UC	L	H/UC
	van der Tempel (1990) [RCT][94]	Fish oil vs placebo at 12 weeks<br>SMD -0.16 (-0.86, 0.53)	CRP, 12 weeks, mean (SD †)<br>Fish oil: 26.5 (18.7)<br>Placebo: 29.5 (18.7)		H/UC	H/UC	L	L
	Cleland (2006) [NRT][98]		CRP, BL / 3 years, mean (range)<br>Fish oil: 30.8 (1, 140) / 4.0 (0.3, 19)<br>Control: 17.2 (4, 34) / 6.6 (3, 15)					
	Bespoke MA [92-94]	Fish oil vs placebo<br>SMD -0.29 (-0.68, 0.11)						
ESR	Gioxari (2018) [MA][84]	Omega 3 vs placebo<br>SMD -0.16 (0.32, -0.00)		Moderate				
	Rajaei (2015) [RCT][89]		ESR, BL / 12 weeks, mean<br>Omega 3: 39 / 16<br>Placebo: 35 / 33; p<0.05		H/UC	H/UC	L	H/UC
	Skoldstam (1992) [RCT][92]	Fish oil vs placebo, change BL-6 months<br>SMD 0.00 (-0.60, 0.60)	ESR, change BL-6 months, mean (SD §)<br>Fish oil: 6 (14.1)<br>Placebo: 6 (18.3)		H/UC	H/UC	L	L
	Tulleken (1990) [RCT][93]	Fish oil vs placebo<br>SMD -1.10 (-1.92, -0.29)	ESR, BL / 3 months, mean (SD †)<br>Fish oil: 38.5 (19.7) / 27.3 (15.8)<br>Placebo: 56.5 (18.2) / 49.5 (23.4)		H/UC	H/UC	L	H/UC
	Kremer (1987) [RCT][97]	Fish oil vs placebo, change BL-14 weeks<br>SMD 0.08 (-0.40, 0.56)	ESR, change BL – 14 weeks, mean (SD*)<br>Fish oil: -0.8 (17.6)<br>Placebo: -2.07 (14.1)		H/UC	H/UC	L	L
	Cleland (2006) [NRT][98]		ESR, BL / 3 years, mean (range)<br>Fish oil: 43.1 (1, 91) / 8.5 (2, 34)<br>Control: 36.5 (4, 80) / 21.5 (8, 46)					
	Bespoke meta-analysis[92;93;97]	Fish oil vs placebo<br>SMD -0.27 (-0.91, 0.37), I^2 68.6%						

* Calculated from 95% CI in paper, § Calculated from standard error in paper, † Mean (SD) calculated from median (range) using published formula [61]

Alloc. Conc. = allocation concealment, AMSTAR2 = Assessing the Methodological Quality of Systematic Reviews, BL = baseline, Blind. Asses. = Blinded assessors, Blind. Part. = blinded participants, CRP = C-reactive protein, CI = confidence interval, ESR = erythrocyte sedimentation rate, H/UC = high / unclear risk of bias, L = low risk of bias, MA = meta-analysis, NRT = non-randomised trial, RA = rheumatoid arthritis, Rand. Seq. = random sequence generation, RCT = randomised controlled trial, SD = standard deviation, SMD = Standardised mean difference

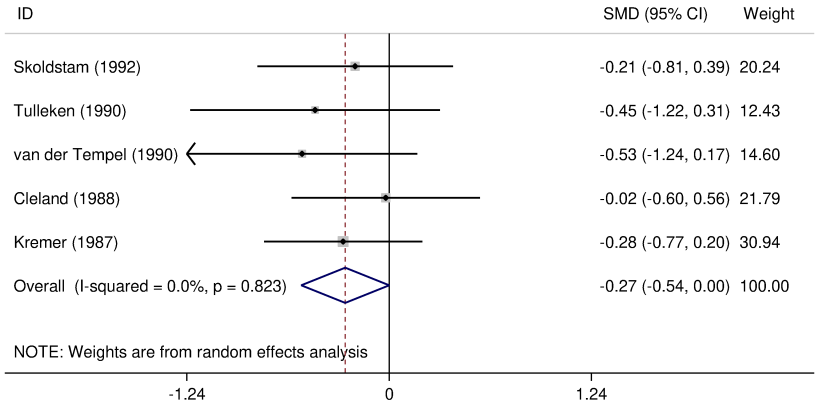

Supplementary figure 3 – Fish oil / omega 3 (RA), bespoke meta-

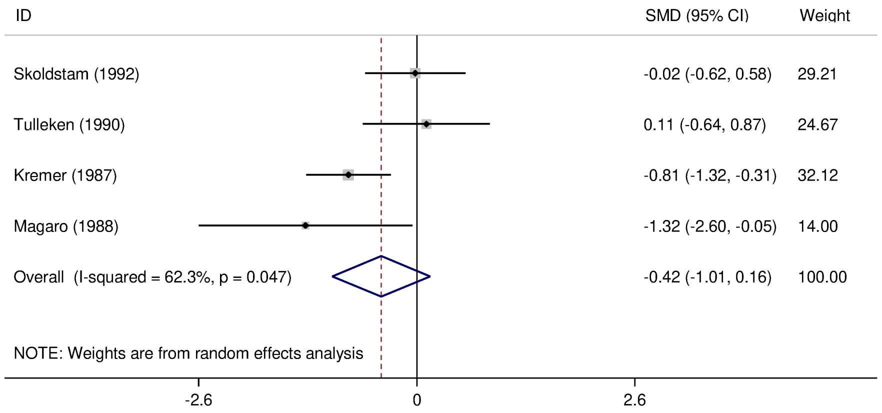

Supplementary figure 4 – Fish oil / omega 3 (RA), bespoke meta-analysis for tender joint count

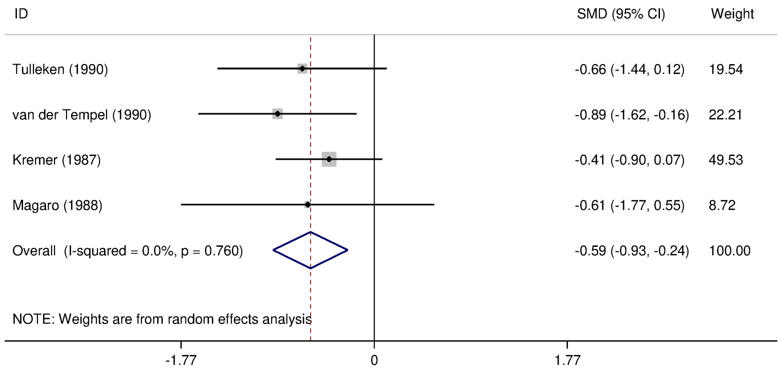

Supplementary figure 5 – Fish oil / omega 3 (RA), bespoke meta-analysis for morning stiffness

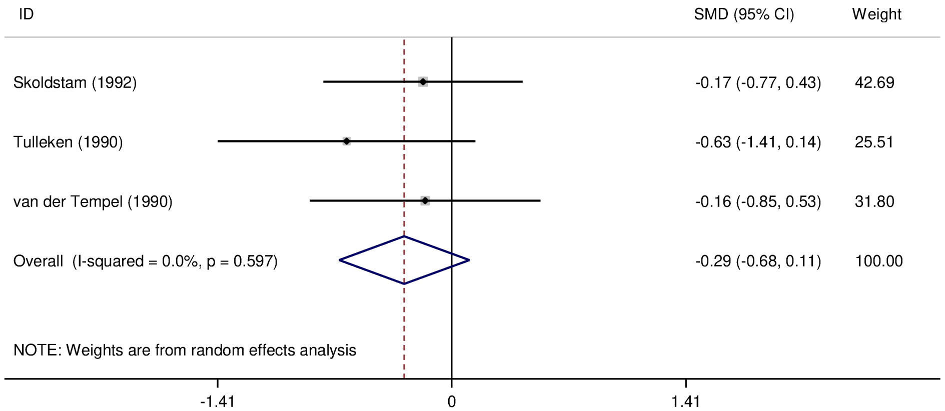

Supplementary figure 6 – Fish oil / omega 3 (RA), bespoke meta-analysis for CRP

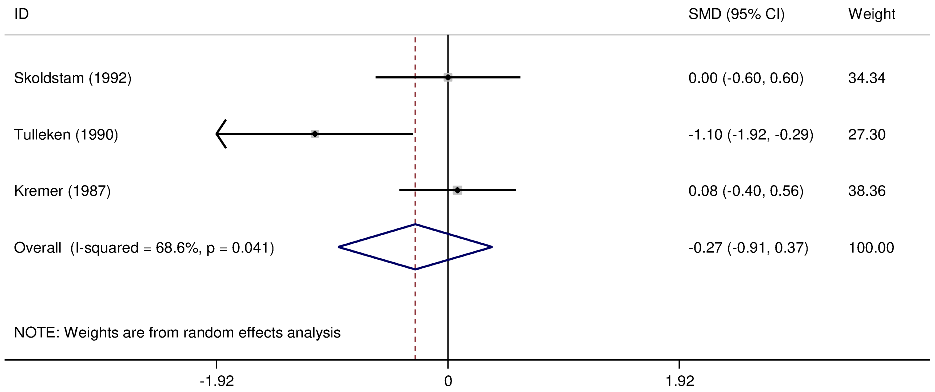

Supplementary figure 7– Fish oil / omega 3 (RA), bespoke meta-analysis for ESR

Supplementary table 67 – Mussel extracts and RA progression, results

Table – Mussels (RA), results and quality assessment

Outcome	Study (date) [study type]	Standardised result, SMD (95% CI) unless otherwise stated	Natural result	AMSTAR2 quality	Rand. Seq.	Alloc. Conc.	Blind. Part.	Blind. Asses.
Pain	Lindqvist (2018) [RCT][87]	Mussels vs control at 11 weeks<br>SMD -0.37 (-0.96, 0.21)	Pain VAS, BL / 11 weeks, mean (SD §)<br>Mussels: 51 (33.2) / 31.3 (30.8)<br>Control: 31.3 (30.8) / 44.3 (38.7)		L	H/UC	H/UC	H/UC
Function	Lindqvist (2018) [RCT][87]	Mussels vs control at 11 weeks<br>SMD -0.20 (-0.78, 0.38)	HAQ, BL / 11 weeks, mean (SD §)<br>Mussels: 0.93 (0.61) / 0.80 (0.91)<br>Control: 0.95 (0.66) / 0.96 (0.70)		L	H/UC	H/UC	H/UC
Disease activity	Lindqvist (2018) [RCT][87]		DAS28, BL / 11 weeks, median (IQR)<br>Mussels: 3.75 (3.15, 4.53) / 3.40 (2.41, 3.73)<br>Control: 3.81 (3.16, 3.73 [sic]) / 3.77 (2.69, 4.22);		L	H/UC	H/UC	H/UC
	Fu (2015) [RCT][88]	Mussels vs control at 11 weeks<br>SMD -0.94 (-1.58, -0.29)	DAS28, BL / 6 months, mean (SD*)<br>Mussels: 5.80 (0.51) / 4.69 (0.51)<br>Control: 5.71 (0.73) / 5.07 (0.69); p<0.01		L	H/UC	L	L
Tender joints	Lindqvist (2018) [RCT][87]	Mussels vs control at 11 weeks<br>SMD -0.22 (-0.80, 0.36)	Tender joint count, BL / 11 weeks, mean (SD §)<br>Mussels: 4.3 (4.0) / 2.7 (3.2)<br>Control: 5.3 (8.7) / 3.7 (5.5)		L	H/UC	H/UC	H/UC
	Fu (2015) [RCT][88]	Mussels vs control at 11 weeks<br>SMD -0.79 (-1.43, -0.16)	Tender joint count, BL / 6 months, mean (SD*)<br>Mussels: 10.6 (1.7) / 5.1 (2.1)<br>Control: 9.5 (2.9) / 6.9 (2.4); p<0.01		L	H/UC	L	L
Swollen joints	Lindqvist (2018) [RCT][87]	Mussels vs control at 11 weeks<br>SMD -0.40 (-0.98, 0.19)	Swollen joint count, BL / 11 weeks, mean (SD §)<br>Mussels: 2 (1.6) / 1 (1.6)<br>Control: 2 (1.6) / 2 (3.2)		L	H/UC	H/UC	H/UC
	Fu (2015) [RCT][88]	Mussels vs control at 11 weeks<br>SMD -0.46 (-1.08, 0.16)	Swollen joint count, BL / 6 months, mean (SD*)<br>Mussels: 7.3 (3.0) / 4.1 (2.1)<br>Control: 7.8 (3.6) / 5.3 (2.9); p=0.053		L	H/UC	L	L
Morning stiffness	Fu (2015) [RCT][88]	Mussels vs control at 11 weeks<br>SMD -0.58 (-1.20, 0.04)	Morning stiffness, BL / 6 months, mean (SD*)<br>Mussels: 69.7 (26.7) / 40.6 (29.7)<br>Control: 72.7 (39.2) / 58.1 (32.3); p=0.016		L	H/UC	L	L
Fatigue	Lindqvist (2018) [RCT][87]	Mussels vs control at 11 weeks<br>SMD -0.49 (-1.08, 0.10)	Fatigue VAS, BL / 11 weeks, mean (SD §)<br>Mussels: 65.0 (26.1) / 46.3 (38.7)<br>Control: 59.0 (30.8) / 61.3 (19.8)		L	H/UC	H/UC	H/UC
Patient global	Lindqvist (2018) [RCT][87]	Mussels vs control at 11 weeks<br>SMD -0.51 (-1.10, 0.07)	Patient global VAS, BL / 11 weeks, mean (SD §)<br>Mussels: 54.3 (25.3) / 33.0 (37.1)<br>Control: 49.0 (26.9) / 47.0 (10.3)		L	H/UC	H/UC	H/UC
	Fu (2015) [RCT][88]	Mussels vs control at 11 weeks<br>SMD 0.18 (-0.43, 0.79)	Patient global VAS, BL / 6 months, mean (SD*)<br>Mussels: 53.5 (11.5) / 42.4 (9.8)<br>Control: 47.9 (12.7) / 40.6 (10.3); p=0.135		L	H/UC	L	L

§ Calculated from median (IQR) using published formula
* Calculated from standard error in paper
Alloc. Conc. = allocation concealment, AMSTAR2 = Assessing the Methodological Quality of Systematic Reviews, BL = baseline, Blind. Asses. = Blinded assessors, Blind. Part. = blinded participants, CI = confidence interval, DAS28 = Disease Activity Score 28, H/UC = high / unclear risk of bias, HAQ = Health Assessment Questionnaire, IQR = interquartile range, L = low risk of bias, RA = rheumatoid arthritis, Rand. Seq. = random sequence generation, RCT = randomised controlled trial, SD = standard deviation, SMD = Standardised mean difference, VAS = visual analogue scale

Table – Mussels (RA) [cont.], results and quality assessment

Outcome	Study (date) [study type]	Standardised result, SMD (95% CI) unless otherwise stated	Natural result	AMSTAR2 quality	Rand. Seq.	Alloc. Conc.	Blind. Part.	Blind. Asses.
CRP	Lindqvist (2018) [RCT][87]	Mussels vs control at 11 weeks<br>SMD 0.00 (-0.58, 0.58)	CRP, BL / 11 weeks, mean (SD §)<br>Mussels: 2.7 (3.2) / 1.7 (1.6)<br>Control: 1.7 (2.4) / 1.7 (2.4)		L	H/UC	H/UC	H/UC
	Fu (2015) [RCT][88]	Mussels vs control at 11 weeks<br>SMD -0.48 (-1.10, 0.14)	CRP, BL / 6 months, mean (SD*)<br>Mussels: 14.4 (7.2) / 11.4 (6.4)<br>Control: 16.3 (9.8) / 14.9 (7.8); p0=0.273		L	H/UC	L	L
ESR	Lindqvist (2018) [RCT][87]	Mussels vs control at 11 weeks<br>SMD -0.15 (-0.73, 0.43)	ESR, BL / 11 weeks, mean (SD §)<br>Mussels: 14.3 (16.2) / 11.0 (11.5)<br>Control: 14.0 (15.8) / 13.0 (15.0)		L	H/UC	H/UC	H/UC
	Fu (2015) [RCT][88]	Mussels vs control at 11 weeks<br>SMD -0.22 (-0.83, 0.40)	ESR, BL / 6 months, mean (SD*)<br>Mussels: 50.8 (27.2) / 34.7 (19.9)<br>Control: 53.3 (29.9) / 38.9 (19.1); p=0.571		L	H/UC	L	L

§ Calculated from median (IQR) using published formula
* Calculated from standard error in paper
Alloc. Conc. = allocation concealment, AMSTAR2 = Assessing the Methodological Quality of Systematic Reviews, BL = baseline, Blind. Asses. = Blinded assessors, Blind. Part. = blinded participants, CI = confidence interval, CRP = C-reactive protein, ESR = erythrocyte sedimentation rate, H/UC = high / unclear risk of bias, IQR = interquartile range, L = low risk of bias, RA = rheumatoid arthritis, Rand. Seq. = random sequence generation, RCT = randomised controlled trial, SD = standard deviation, SMD = Standardised mean difference

Table – Mussels (RA), SF36 results at final follow-up

Author (date)	PCS	MCS	GH	PF	RP	RE	SF	BP	V	MH
Lindqvist (2018) [Mussels][87]	39.3 (12.6)	51 (7.1)								
Lindqvist (2018) [Control][87]	38 (8.7)	47 (10.3)								

BP = bodily pain, GH = general health, MCS = mental component score, MH = mental health, PCS = physical component score, PF = physical function, RA = rheumatoid arthritis, RE = role emotional, RP = role physical, SF = social functioning, V = vitality

Supplementary table 68 – Description of reviews of experimental diets in RA

Table – Experimental diet (RA), description of reviews

Authors (date)	Review type	Study type included	Exposure detail	Number of studies included	Funders
Cramp (2013)[85]	MA	RCTs	Mediterranean diet	1	**Charity** (Arthritis Research UK)

MA = meta-analysis, RA = rheumatoid arthritis, RCT = randomised controlled trial

Supplementary table 69 – Description of studies of experimental diets in RA

Table – Experimental diets (RA), description of included studies

Author (date) [country]	Study design	Inclusion criteria	Exposure detail	N	Age, mean (SD) years	N (%) female	Funders
Podas (2007) [UK][99]	RCT	1987 RA criteria, active RA (≥3 of: ≥3 swollen joints, ≥6 tender joints, >45 mins morning stiffness, >28mm ESR), stable DMARDs for 6 weeks<br>Exclusions: pregnancy, diabetes, other systemic illnesses	1) Liquid elemental diet E028<br>p) Oral steroids	1) 21<br>p) 9	1) 47<br>p) 48	1) 16 (76.2)<br>p) 6 (66.7)	**Industry** (Scientific Hospital Supplies Ltd)
Skoldstam (2003) [Sweden][100]	RCT	1987 RA criteria, symptom duration >2 years, stable disease under adequate control<br>Exclusions: DMARDs unchanged for >3 months, steroids for >4 weeks, and NSAIDs >0 days. Daily dose oral steroids not >12.5, DAS28 >2.0, no other comorbidities that demand active medical attention, vegetarians, those already eating Mediterranean-like diet	1) Mediterranean diet<br>p) Continue regular diet	1) 26<br>p) 25	1) 58<br>(range: 33-73)<br>p) 59<br>(range: 35-75)	1) 21 (80.8)<br>p) 20 (80)	**University** (Faculty of Social Sciences of Umea University), **Public Foundation** (Swedish Foundation for Health Care Sciences and Allergy Research), **Government** (Health Research Council), **Charity** (Swedish Nutrition Foundation, the JC Kempe Memorial Scholarship Fund, the Borgerskapet i Umeå Fund, and the Uppsala Hemsysterskola Fund.
Hafstrom (2001) [Sweden][101]	RCT	1987 RA criteria, aged 20-69 years, symptom duration 2-10 years, not tried dietary manipulation, no history of food sensitivity, active disease, stable dose of DMARDs	1) Vegan diet with no gluten<br>p) Non-vegan diet – well balanced	1) 38<br>p) 28	1) 49.5 (9.6)<br>p) 50.8 (11.9)	Not reported	**Charity** (Axel and Margaret Ax:son Johnson Foundation, Swedish Rheumatism Association), **Government** (Swedish Medical Research Council)
Sarzi-Puttini (2000) [Italy][102]	RCT	1987 RA criteria, aged 25-70 years, Steinbrocker functional class I-II, stable therapy for 12 weeks, ≥4 of the following: ≥5 painful joints, ≥3 swollen joints, ≥4 pain VAS, ≥45 mins morning stiffness, ≥30 mm/hr ESR	1) Hypoallergenic diet (rice, cornmeal, cornbread, hydrolysed milk, fresh pineapple, cooked apple) with no: wheal meal, eggs, milk, strawberries and acid fruit, tomato, chocolate, crustacean, dried fruit.<br>p) Same calorie content but containing allergenic food.	1) 22<br>p) 21	1) 49.56 (range: 32-64)<br>p) 50.28 (range: 29-70)	1) 19 (76)<br>p) 20 (80)	Not reported

* estimated from median and range in paper using published formula[61]

DAS28 = Disease Activity Score 28, DMARDs = disease modifying anti-rheumatoid drugs, ESR = erythrocyte sedimentation rate, hr = hour, mm = millimetres, N = number, NGO = non-governmental organisation, NSAID = non-steroidal anti-inflammatory drugs, RA = rheumatoid arthritis, RCT = randomised controlled trial, SD = standard deviation, UK = United Kingdom, VAS = visual analogue scale

Table – Experimental diets (RA), description of included studies

Author (date) [country]	Study design	Inclusion criteria	Exposure detail	N	Age, mean (SD) years	N (%) female	Funders
Holst-Jensen (1998) [Denmark][103]	RCT	1987 RA criteria, aged 18-75 years, symptom duration ≥6 months, active RA (at least three of: ≥3 swollen joints, ≥6 tender joints, ≥28mm/hr ESR, ≥45 mins morning stiffness), stable DMARDs for 3 months<br>Exclusions: Signs or symptoms of any other severe disease, pacemaker, prosthetic joint, electrolyte derangement, edema	1) Liquid elemental diet, no solids<br>p) Continue normal diet	1) 15<br>p) 15	Median (10th / 90th percentiles):<br>1) 46 (29/72)<br>p) 56 (34/70)	1) 14 (93)<br>p) 10 (67)	**NGO** (Danish Rheumatoid Association), **Industry** (Ferrosan Ltd.)
Nenonen (1998) [Finland][104]	RCT	1987 RA, Steinbrocker functional class II-III, >3 swollen joints or >5 tender joints, >20 ESR or >10 CRP	1) Uncooked, lactobacilli rich, vegan diet<br>p) Continue normal diet	1) 19<br>p) 20	1) 49.1 (7.1)<br>p) 55.6 (10.8)	1) 18 (94.7)<br>p) 19 (95.0)	**Charity (**Juho Vainio Foundation)
Kavanagh (1995) [UK][105]	RCT	Definite RA<br>Exclusions: Taking steroids / DMARDs	1) Liquid elemental diet E028 + chicken, fish, rice, carrots, runner beans and bananas<br>p) E028 + normal diet (elemental diet to replace some drinks)	1) 24<br>p) 23	1) 42.8 (10.5)<br>p) 48.5 (13.7)	1) 18 (75)<br>p) 19 (82.6)	**Charity** (Arthritis Rheumatism Council)
Haugen (1994) [Norway][106]	RCT	1987 RA criteria, active RA (at least three of: : ≥3 swollen joints, ≥6 tender joints, ≥28mm/hr ESR, ≥45 mins morning stiffness), stable DMARDs for 3 months, steroid dose ≤7.5mg per day and stable for 4 weeks	1) Liquid elemental diet E028<br>P) Soup	1) 10<br>p) 7	1) 50.3 (13.3) *<br>p) 53.5 (13.9) *	1) 9 (90)<br>p) 5 (71.4)	**Charity** (The Norwegian Women's Public Health Association, Anders Jahres Legacy, Grethe Harbitz Legacy, Eckbo Legacy, Olga Imerslund legacy)
van de Laar (1992) [The Netherlands][107]	RCT	Met ≥6 ARA 1958 criteria (1 had to be RF+), ≥3 of the following: >28mm/h ESR, >45 mins morning stiffness, >5 tender joints, >2 swollen joints<br>Exclusions: function class IV	1) Allergy / additive / preservative free diet<br>2) Allergy free other than milk allergens and azo colourings	1) 45<br>2) 49	1) 57.7<br>2) 58.6	1) 30 (66.7)<br>p) 36 (73.5)	**Industry** (het Praeventiefonds)
Panush (1983) [USA][108]	RCT	Definite Stage I-III, RA after 16 years, stable medication regime, ≥3 of the following: ≥6 tender joints, ≥3 swollen joints, ≥45 minutes morning stiffness, >228mm/hr ESR	1) Diet consisting or little meat except fish and occasional fowl, no fruit, no herbs, no spices, no dairy products, no alcohol, no additives, no preservatives, supplemental iron and vitamins<br>p) Placebo diet – excluded select items from food groups, but included those eliminated from experimental diet	1) 11<br>p) 15	1) 53.6<br>p) 56.3	1) 5 (45.5)<br>p) 4 (26.7)	**Charity** (Arthritis Foundation), **Government** (Veterans Administration)

* estimated from median and range in paper using published formula[61]

ARA = American Rheumatism Association, CRP = C-reactive protein, DMARDs = disease modifying anti-rheumatoid drugs, ESR = erythrocyte sedimentation rate, hr = hour, mm = millimetres, N = number, NGO = non-governmental organisation, RA = rheumatoid arthritis, RCT = randomised controlled trial, RF = rheumatoid factor, SD = standard deviation, UK = United Kingdom, USA = United States of America

Table – Experimental diets (RA), description of included studies

Author (date) [country]	Study design	Inclusion criteria	Exposure detail	N	Age, mean (SD) years	N (%) female	Funders
Sundqvist (1982) [Sweden][109]	RCT	1958 RA criteria, Functional class I-II, stable medication for last 2 months	1) Fasted for 10 days then vegetarian diet with no alcohol, tobacco or coffee/tea<br>p) Normal diet	1) 5<br>p) 5	Not reported	Not reported	**Charity** (Swedish National Association Against Rheumatism)
Skoldstam (1979) [Sweden][110]	RCT	1958 RA criteria, low-moderate inflammatory activity, functional classes I-II, taking NSAIDs, stable treatment in months preceding trial	1) Fasting for 7-10 days followed by lactovegetarian diet (no animal/fish, yoghurt ok by milk/cream discouraged<br>p) No diet intervention	1) 16<br>p) 10	1) 52<br>(range: 35-66)<br>p) 54<br>(range: 43-65)	1) 10 (62.5)<br>p) 9 (90.0)	**Charity** (Swedish National Association Against Rheumatism)
Abendroth (2010) [Germany][111]	NRT	1987 RA criteria<br>Exclusions: antibiotics in last 4 weeks, malnutrition, BMI <19 or >40, renal insufficiency, pregnancy, malignant disorders, mental inability to co-operate, participation in another study	1) Mediterranean diet<br>2) Fasting (800kcal per day)	1) 28<br>2) 22	1) 60.0 (12.1)<br>2) 55.7 (7.2)	1) 26 (92.9)<br>2) 21 (95.5)	Not reported (authors declare no conflicts)
McKellar (2007) [UK][112]	NRT	Aged 30-70	1) Went on Mediterranean diet cooking course and then given recipes and information on healthy eating<br>p) Received freely available information on healthy eating only	1) 75<br>p) 55	Median (IQR)<br>1) 58 (47, 64)<br>p) 52 (45, 61)	1) 75 (100)<br>p) 55 (100)	**Professional body** (Scottish Society of Physicians)
Adam (2003) [Germany][113]	NRT	1987 RA, Stable medication for 4 weeks for NSAIDs and 8 weeks for DMARDs<br>Exclusions: gastrointestinal or metabolic diseases, alcohol abuse, known allergies	1) Modified lactovegetarian diet (only plant derived fats and oils, no egg yolk, dairy products with reduced fat, limited meta intake)<br>p) Western diet	1) 30<br>p) 30	1) 58.0 (12.5)<br>p) 56.8 (13.3)	Adam (2003) [Germany][113]	**Government** (Governmental Ministry of Research and Technology of Germany)
Fraser (2000) [Norway][114]	NRT	1987 RA criteria	1) Ketogenic<br>2) Fasting (<865 kJ)	1) 13<br>2) 10	1) 44<br>(range: 25-69)<br>2) 49<br>(range: 31-65)	1) 12 (92)<br>2) 9 (90)	**Charity** (Norwegian Women's Public Health Association)
Denissov (1992) [Russia] [115]	NRT	Classical or definite RA, Stable treatment 6-12 months before trial	1) Hypoallergenic, anti-inflammatory diet<br>p) Conventional therapy only	1) 68<br>p) 24	47.7 (1.3)	1) 65 (95.6)<br>p) 20 (83.3)	Not reported
McDougall (2002) [USA][116]	Single Arm int.	Moderate to severe RA, Stable medication<br>Exclusions: not following vegan / dairy free diet, diabetes, heart disease, high blood pressure, cancer, other chronic disease	Vegan diet, with no added fats or oils	24	56 (11)	22 (91.6)	**Charity** (Betty Wood Estate)
Kjeldsen-Kragh (1994) [Norway][117]	RCT - extension	Classic or definite RA	1) Vegetarian diet – responders<br>2) Vegetarian diet – non-responders<br>p) Control	1) 10<br>2) 12<br>p) 21	1) 50<br>(range: 30-63)<br>2) 54<br>(range: 37-63)<br>p) 55<br>(range: 38-78)	1) 9 (90.0)<br>2) 10 (83.3)<br>p) 19 (95.0)	**Charity** (Norwegian Women's Public Health Association, The Anders Jahre's Fund for Promotion of Science, The Isberg's Legacy, The Grethe Harbitz Legacy and The Eckbo's Legacy.)

* estimated from median and range in paper using published formula[61], BMI = body mass index, DMARDs = disease modifying anti-rheumatoid drugs, ESR = erythrocyte sedimentation rate, hr = hour, kcal = kilocalories, kJ = kilojoules, mm = millimetres, N = number, NGO = non-governmental organisation, NRT = non-randomised trial, NSAID = non-steroidal anti-inflammatory drug, RA = rheumatoid arthritis, RCT = randomised controlled trial, SD = standard deviation, UK = United Kingdom

Supplementary table 70 – Elemental diet and RA progression, results

Table – Elemental diet (RA), results and quality assessment

Outcome	Study (date) [study type]	Standardised result, SMD (95% CI) unless otherwise stated	Natural result	AMSTAR2 quality	Rand. Seq.	Alloc. Conc.	Blind. Part.	Blind. Asses.
Pain	Podas (2007) [RCT][99]	Elemental diet vs steroids at 2 weeks<br>SMD 1.36 (0.51, 2.22) [in favour of steroids]	Pain VAS, BL / 2 weeks, mean* (SD*)<br>Elemental diet: 6.90 (1.38) / 5.05 (1.85)<br>Steroids: 4.35 (2.07) / 2.58 (1.71)		L	H/UC	H/UC	L
	Holst-Jensen (1998) [RCT][103]		Pain (0-30), BL / 6 months, median (10/90 centiles)<br>Elemental diet: 17.0 (5.4, 23.6) / 17.0 (6.4, 22.4)<br>Placebo: 15.0 (3.6, 23.6) / 14.0 (4.6, 22.4)		H/UC	H/UC	H/UC	L
Function	Podas (2007) [RCT][99]	Elemental diet vs steroids at 2 weeks<br>SMD 0.49 (-0.30, 1.28)	HAQ, BL / 2 weeks, mean* (SD*)<br>Elemental diet: 1.88 (0.66) / 1.68 (0.76)<br>Steroids: 1.90 (0.40) / 1.30 (0.80)		L	H/UC	H/UC	L
	Holst-Jensen (1998) [RCT][103]		HAQ, BL / 6 months, median (10/90 centiles)<br>Elemental diet: 1.00 (0.68, 2.03) / 1.00 (0.50, 2.20)<br>Placebo: 1.19 (0.32, 1.88) / 1.19 (0.00, 2.19)		H/UC	H/UC	H/UC	L
	Kavanagh (1995) [RCT][105]	Elemental diet vs control at 4 weeks<br>SMD -0.13 (-0.70, 0.44)	‘Functional score’, BL / 4 weeks, mean (SD)<br>Elemental diet: 10.65 (5.66) / 9.7 (6.3)<br>Control: 9.32 (4.92) / 10.5 (5.9)		H/UC	H/UC	H/UC	H/UC
Morning stiffness	Podas (2007) [RCT][99]	Elemental diet vs steroids at 2 weeks<br>SMD 1.22 (0.37, 2.06) [in favour of steroids]	Morning stiffness, BL / 2 weeks, mean* (SD*)<br>Elemental diet: 443 (373) / 414 (380)<br>Steroids: 188 (151) / 23 (25) [sic]		L	H/UC	H/UC	L
	Holst-Jensen (1998) [RCT][103]		Morning stiffness, BL / 6 months, median (10/90 centiles)<br>Elemental diet: 2.0 (1.0, 7.8) / 3.0 (1.0, 6.0)<br>Placebo: 3.5 (1.0, 7.5) / 2.5 (1.0, 6.0)		H/UC	H/UC	H/UC	L
Tender joints	Podas (2007) [RCT][99]	Elemental diet vs steroids at 2 weeks<br>SMD 0.52 (-0.27, 1.31)	Ritchie Index, BL / 2 weeks, mean* (SD*)<br>Elemental diet: 31.5 (16.9) / 26 (16.9)<br>Steroids: 29.8 (18.4) / 17.8 (12.4)		L	H/UC	H/UC	L
	Holst-Jensen (1998) [RCT][103]		Ritchie Index, BL / 6 months, median (10/90 centiles)<br>Elemental diet: 9.5 (4.0, 21.5) / 10.0 (5.3, 16.4)<br>Placebo: 12.5 (7.3, 33) / 10.0 (3.6, 23.0)		H/UC	H/UC	H/UC	L
	Haugen (1994) [RCT][106]	Elemental diet vs control change from baseline to 4 weeks<br>SMD -0.32 (-1.30, 0.65)	Tender joint count, mean (SD) change baseline to 4 weeks §<br>Elemental diet: -4.5 (5.72)<br>Placebo: -2.4 (7.46)		H/UC	H/UC	H/UC	H/UC
	Kavanagh (1995) [RCT][105]	Elemental diet vs control at 4 weeks<br>SMD -0.43 (-1.01, 0.15)	Ritchie Index, BL / 4 weeks, mean (SD)<br>Elemental diet: 12.6 (6.8) / 10.3 (6.9)<br>Control: 10.4 (7.2) / 14.1 (10.5)		H/UC	H/UC	H/UC	H/UC
	Bespoke MA of:<br>Kavanagh (1995)<br>Haugen (1994)	Elemental diet vs control<br>SMD -0.40 (-0.90, 0.10)<br>I^2 0%						

* Estimated from median and range in paper using published formula[61]; § Calculated by reviewers from data published in the paper; Alloc. Conc. = allocation concealment, AMSTAR2 = Assessing the Methodological Quality of Systematic Reviews, BL = baseline, Blind. Asses. = Blinded assessors, Blind. Part. = blinded participants, CI = confidence interval, H/UC = high / unclear risk of bias, HAQ = Health Assessment Questionnaire, L = low risk of bias, MA = meta-analysis, RA = rheumatoid arthritis, Rand. Seq. = random sequence generation, RCT = randomised controlled trial, SD = standard deviation, SMD = Standardised mean difference

Table – Elemental diet (RA) [cont.], results and quality assessment

Outcome	Study (date) [study type]	Standardised result, SMD (95% CI) unless otherwise stated	Natural result	AMSTAR2 quality	Rand. Seq.	Alloc. Conc.	Blind. Part.	Blind. Asses.
Swollen joints	Podas (2007) [RCT][99]	Elemental diet vs steroids at 2 weeks<br>SMD -0.17 (-0.95, 0.61)	Swollen joint count, BL / 2 weeks, mean* (SD*)<br>Elemental diet: 42 (22.8) / 41 (23.8)<br>Steroids: 64.5 (31.5) / 45 (22.8)		L	H/UC	H/UC	L
	Holst-Jensen (1998) [RCT][103]		Swollen joint count, BL / 6 months, median (10/90 centiles)<br>Elemental diet: 9.0 (5.2, 13.8) / 7.0 (5.0, 12.0)<br>Placebo: 11.0 (5.8, 23.4) / 9.0 (3.4, 23.6)		H/UC	H/UC	H/UC	L
	Haugen (1994) [RCT][106]	Elemental diet vs control change from baseline to 4 weeks<br>SMD -0.26 (-1.23, 0.71)	Swollen joint count, mean (SD) change baseline to 4 weeks §<br>Elemental diet: -2.6 (3.86)<br>Placebo: -1.7 (2.87)		H/UC	H/UC	H/UC	H/UC
CRP	Podas (2007) [RCT][99]	Elemental diet vs steroids at 2 weeks<br>SMD 1.33 (0.47, 2.18) [in favour of steroids]	CRP, BL / 2 weeks, mean* (SD*)<br>Elemental diet: 5.5 (3.8) / 6.4 (4.6)<br>Steroids: 4.4 (1.6) / 1.2 (0.9)		L	H/UC	H/UC	L
	Holst-Jensen (1998) [RCT][103]		CRP, BL / 6 months, median (10/90 centiles)<br>Elemental diet: 11 (5, 57) / 11 (4, 59)<br>Placebo: 25 (10, 78) / 15 (4, 142)		H/UC	H/UC	H/UC	L
	Haugen (1994) [RCT][106]	Elemental diet vs control change from baseline to 4 weeks<br>SMD 0.23 (-0.74, 1.20)	CRP, mean (SD) change baseline to 4 weeks §<br>Elemental diet: 5.7 (25.43)<br>Placebo: 1.14 (4.18)		H/UC	H/UC	H/UC	H/UC
	Kavanagh (1995) [RCT][105]	Elemental diet vs control at 4 weeks<br>SMD 0.10 (-0.47, 0.67)	CRP, BL / 4 weeks, mean (SD)<br>Elemental diet: 16.4 (18.7) / 12.3 (12.4)<br>Control: 8.6 (8.3) / 11.4 (1.7 [sic])		H/UC	H/UC	H/UC	H/UC
	Bespoke MA of:<br>Kavanagh (1995)<br>Haugen (1994)	Elemental diet vs control<br>SMD 0.13 (-0.36, 0.63)<br>I^2 0%						

* estimated from median and range in paper using published formula[61]; § Calculated by reviewers from data published in the paper

Alloc. Conc. = allocation concealment, AMSTAR2 = Assessing the Methodological Quality of Systematic Reviews, BL = baseline, Blind. Asses. = Blinded assessors, Blind. Part. = blinded participants, CI = confidence interval, CRP = C-reactive protein, H/UC = high / unclear risk of bias, L = low risk of bias, MA = meta-analysis, RA = rheumatoid arthritis, Rand. Seq. = random sequence generation, RCT = randomised controlled trial, SD = standard deviation, SMD = Standardised mean difference,

Supplementary table 71 – Hypoallergenic diet and RA progression, results

Table – Hypoallergenic diet (RA), results and quality assessment

Outcome	Study (date) [study type]	Standardised result, SMD (95% CI) unless otherwise stated	Natural result	AMSTAR2 quality	Rand. Seq.	Alloc. Conc.	Blind. Part.	Blind. Asses.
Pain	Sarzi-Puttini (2000) [RCT][102]	Hypoallergenic diet vs control at 24 weeks<br>SMD -0.16 (-0.76, 0.44)	Pain VAS, BL / 24 weeks, mean (SD)<br>Hypoallergenic diet: 46.8 (16.1) / 37.6 (12.3)<br>Control: 44.2 (18.7) / 40.4 (21.5)		H/UC	H/UC	H/UC	H/UC
	Denissov (1992) [non-randomised trial][115]		Pain (0-3), BL / 4 weeks, mean (SD/SE*)<br>Hypoallergenic diet: 1.75 (0.1) / 1.1 (0.07)<br>Control: 1.6 (0.13) / 1.0 (0.09)					
Tender joints	Sarzi-Puttini (2000) [RCT][102]	Hypoallergenic diet vs control at 24 weeks<br>SMD -0.22 (-0.82, 0.38)	Ritchie Index, BL / 24 weeks, mean (SD)<br>Hypoallergenic diet: 13.2 (4.4) / 9.2 (3.8)<br>Control: 11.7 (4.3) / 10.1 (4.5)		H/UC	H/UC	H/UC	H/UC
	Denissov (1992) [non-randomised trial][115]		Ritchie Index, BL / 4 weeks, mean (SD/SE*)<br>Hypoallergenic diet: 15.7 (1.2) / 10.6 (0.9)<br>Control: 15.9 (1.7) / 10.1 (1.5)					
	van de Laar (1992) [RCT][107]	Hypoallergenic diet vs Hypoallergenic diet + milk, change from BL-12 weeks<br>SMD 0.02 (-0.39, 0.42)	Ritchie Index, mean (SD) change BL-12 weeks<br>Hypoallergenic diet: -1.9 (6.8)<br>Hypoallergenic diet + milk: -2.0 (6.1)		H/UC	H/UC	H/UC	H/UC
Swollen joints	Sarzi-Puttini (2000) [RCT][102]	Hypoallergenic diet vs control at 24 weeks<br>SMD -0.15 (-0.75, 0.45)	Swollen joint count, BL / 24 weeks, mean (SD)<br>Hypoallergenic diet: 6.4 (3.1) / 5.1 (2.3)<br>Control: 5.7 (2.7) / 5.5 (3.0)		H/UC	H/UC	H/UC	H/UC
Morning stiffness	Sarzi-Puttini (2000) [RCT][102]	Hypoallergenic diet vs control at 24 weeks<br>SMD -0.14 (-0.74, 0.46)	Morning stiffness, BL / 24 weeks, mean (SD)<br>Hypoallergenic diet: 62.5 (51.9) / 40.6 (34.2)<br>Control: 51.4 (42.1) / 45.8 (40.3)		H/UC	H/UC	H/UC	H/UC
	van de Laar (1992) [RCT][107]	Hypoallergenic diet vs Hypoallergenic diet + milk, change from BL-12 weeks<br>SMD 0.10 (-0.30, 0.51)	Morning stiffness, mean (SD) change BL-12 weeks<br>Hypoallergenic diet: -23.4 (39.1)<br>Hypoallergenic diet + milk: -27.3 (38.0)		H/UC	H/UC	H/UC	H/UC
	Denissov (1992) [non-randomised trial][115]		Morning stiffness (mins), BL / 4 weeks, mean (SD/SE*)<br>Hypoallergenic diet: 115.4 (25.3) / 56.7 (19.4)<br>Control: 89.2 (18.5) / 38.1 (13.9)					
Fatigue	van de Laar (1992) [RCT][107]	Hypoallergenic diet vs Hypoallergenic diet + milk, change from BL-12 weeks<br>SMD -0.28 (-0.69, 0.12)	Fatigue VAS, mean (SD) change BL-12 weeks<br>Hypoallergenic diet: 0.7 (1.3)<br>Hypoallergenic diet + milk: 1.1 (1.5)		H/UC	H/UC	H/UC	H/UC
Patient global	van de Laar (1992) [RCT][107]	Hypoallergenic diet vs Hypoallergenic diet + milk, change from BL-12 weeks<br>SMD -0.31 (-0.72, 0.10)	Patient global VAS, mean (SD) change BL-12 weeks<br>Hypoallergenic diet: 0.7 (1.3)<br>Hypoallergenic diet + milk: 1.1 (1.3)		H/UC	H/UC	H/UC	H/UC

* Unclear whether the paper reported standard deviations or standard errors – hence have not calculated an SMD
Alloc. Conc. = allocation concealment, AMSTAR2 = Assessing the Methodological Quality of Systematic Reviews, BL = baseline, Blind. Asses. = Blinded assessors, Blind. Part. = blinded participants, CI = confidence interval, CRP = C-reactive protein, ESR = erythrocyte sedimentation rate, H/UC = high / unclear risk of bias, L = low risk of bias, RA = rheumatoid arthritis, Rand. Seq. = random sequence generation, RCT = randomised controlled trial, SD = standard deviation, SE = standard error, SMD = Standardised mean difference, VAS = visual analogue scale

Table – Hypoallergenic diet (RA) [cont.], results and quality assessment

Outcome	Study (date) [study type]	Standardised result, SMD (95% CI) unless otherwise stated	Natural result	AMSTAR2 quality	Rand. Seq.	Alloc. Conc.	Blind. Part.	Blind. Asses.
CRP	van de Laar (1992) [RCT][107]	Hypoallergenic diet vs Hypoallergenic diet + milk, change from BL-12 weeks<br>SMD 0.27 (-0.14, 0.68)	CRP, mean (SD) change BL-12 weeks<br>Hypoallergenic diet: -1.7 (15.7)<br>Hypoallergenic diet + milk: -5.5 (12.2)		H/UC	H/UC	H/UC	H/UC
ESR	Sarzi-Puttini (2000) [RCT][102]	Hypoallergenic diet vs control at 24 weeks<br>SMD -0.08 (-0.67, 0.52)	ESR, BL / 24 weeks, mean (SD)<br>Hypoallergenic diet: 36.2 (18.8) / 28.9 (18.9)<br>Control: 33.1 (20.1) / 30.6 (25.8)		H/UC	H/UC	H/UC	H/UC
	van de Laar (1992) [RCT][107]	Hypoallergenic diet vs Hypoallergenic diet + milk, change from BL-12 weeks<br>SMD 0.20 (-0.21, 0.61)	ESR, mean (SD) change BL-12 weeks<br>Hypoallergenic diet: 2.0 (10.9)<br>Hypoallergenic diet + milk: 0.2 (6.9)		H/UC	H/UC	H/UC	H/UC
Grip strength	van de Laar (1992) [RCT][107]	Hypoallergenic diet vs Hypoallergenic diet + milk, change from BL-12 weeks<br>*Left*<br>SMD 0.24 (-0.17, 0.64)<br>*Right*<br>SMD 0.22 (-0.19, 0.62)	Grip strength, mean (SD) change BL-12 weeks<br>*Left*<br>Hypoallergenic diet: 4.4 (7.1)<br>Hypoallergenic diet + milk: 2.7 (7.2)<br>*Right*<br>Hypoallergenic diet: 2.5 (9.2)<br>Hypoallergenic diet + milk: 0.8 (6.4)		H/UC	H/UC	H/UC	H/UC
Grip strength	Denissov (1992) [non-randomised trial][115]		Grip strength, BL / 4 weeks, mean (SD/SE*)<br>*Left*<br>Hypoallergenic diet: 210.6 (17.1) / 239.5 (15.0)<br>Control: 114.9 (21.1) 134.9 (19.0)<br>*Right*<br>Hypoallergenic diet: 204.5 (16.0) / 247.3 (14.6)<br>Control: 131.2 (20) / 147.1 (20.9)					

* Unclear whether the paper reported standard deviations or standard errors – hence have not calculated an SMD

Alloc. Conc. = allocation concealment, AMSTAR2 = Assessing the Methodological Quality of Systematic Reviews, BL = baseline, Blind. Asses. = Blinded assessors, Blind. Part. = blinded participants, CI = confidence interval, CRP = C-reactive protein, ESR = erythrocyte sedimentation rate, H/UC = high / unclear risk of bias, L = low risk of bias, RA = rheumatoid arthritis, Rand. Seq. = random sequence generation, RCT = randomised controlled trial, SD = standard deviation, SE = standard error, SMD = Standardised mean difference

Supplementary table 72 – Ketogenic diet and RA progression, results

Table – Ketogenic diet (RA), results and quality assessment

Outcome	Study (date) [study type]	Standardised result, SMD (95% CI) unless otherwise stated	Natural result	AMSTAR2 quality	Rand. Seq.	Alloc. Conc.	Blind. Part.	Blind. Asses.
Tender joints	Fraser (2000) [NRT][114]		Tender joint count (28), BL / day 7, median (95% CI)<br>Ketogenic diet: 12 (6, 16) / 8 (5, 14)<br>Fasting: 14 (8, 21) / 10 (2, 17)					
CRP	Fraser (2000) [NRT][114]		CRP, BL / day 7, median (95% CI)<br>Ketogenic diet: 13 (5, 61) / 19 (9, 56)<br>Fasting: 25 (13, 47) / 13 (7, 33)					
ESR	Fraser (2000) [NRT][114]		ESR, BL / day 7, median (95% CI)<br>Ketogenic diet: 28 (20, 48) / 28 (16, 40)<br>Fasting: 33 (22, 54) / 21 (10, 48)					

Alloc. Conc. = allocation concealment, AMSTAR2 = Assessing the Methodological Quality of Systematic Reviews, BL = baseline, Blind. Asses. = Blinded assessors, Blind. Part. = blinded participants, CI = confidence interval, CRP = C-reactive protein, ESR = erythrocyte sedimentation rate, H/UC = high / unclear risk of bias, L = low risk of bias, NRT = non-randomised trial, RA = rheumatoid arthritis, Rand. Seq. = random sequence generation, SMD = Standardised mean difference,

Supplementary table 73 – Mediterranean diet and RA progression, results

Table – Mediterranean diet (RA), results and quality assessment

Outcome	Study (date) [study type]	Standardised result, SMD (95% CI) unless otherwise stated	Natural result	AMSTAR2 quality	Rand. Seq.	Alloc. Conc.	Blind. Part.	Blind. Asses.
Pain	Skoldstam (2003) [RCT][100]	Mediterranean diet vs usual diet<br>SMD -0.81 (-1.38, -0.23)	Pain VAS, BL / 12 weeks, mean (SD)<br>Mediterranean diet: 32 (20) / 20 (13)<br>Usual diet: 31 (20) / 34 (21); p=0.006		H/UC	H/UC	H/UC	H/UC
	McKellar (2007) [NRT][112]		Pain VAS, BL / 6 months, median<br>Mediterranean diet: 50 / 50<br>Healthy eating info: 55 / 63, p=0.049					
Function	Skoldstam (2003) [RCT][100]	Mediterranean diet vs usual diet<br>SMD -0.39 (-0.95, 0.16)	HAQ, BL / 12 weeks, mean (SD)<br>Mediterranean diet: 0.7 (0.5) / 0.6 (0.4)<br>Usual diet: 0.8 (0.6) / 0.8 (0.6); p=0.012		H/UC	H/UC	H/UC	H/UC
	Abendroth (2010) [NRT][111]	Mediterranean diet vs fasting<br>SMD 0.58 (0.01, 1.15) in favour of fasting	HAQ, BL / 7 days, mean (SD)<br>Mediterranean diet: 2.4 (0.8) / 2.2 (0.8)<br>Fasting: 2.0 (0.6) / 1.8 (0.5); p=0.571					
	McKellar (2007) [NRT][112]		HAQ, BL / 6 months, median<br>Mediterranean diet: 1.75 / 1.625<br>Healthy eating info: 1.75 / 1.875, p=NS					
Disease Activity	Skoldstam (2003) [RCT][100]	Mediterranean diet vs usual diet<br>SMD -0.30 (-0.85, 0.26)	DAS28, BL / 12 weeks, mean (SD)<br>Mediterranean diet: 4.4 (1.2) / 3.9 (1.2)<br>Usual diet: 4.3 (1.4) / 4.3 (1.5); p=0.047		H/UC	H/UC	H/UC	H/UC
	McKellar (2007) [NRT][112]		DAS28, BL / 6 months, median<br>Mediterranean diet: 4.7 / 4.4<br>Healthy eating info: 5.0 / 4.8					
SF36-physical	Abendroth (2010) [NRT][111]	Mediterranean diet vs fasting<br>SMD 0.00 (-0.56, 0.56)	SF36-physical, BL / 7 days, mean (SD)<br>Mediterranean diet: -2.0 (0.8) / -1.5 (0.9)<br>Fasting: -2.1 (0.9) / -1.5 (1.1)					
Tender joints	Skoldstam (2003) [RCT][100]	Mediterranean diet vs usual diet<br>SMD -0.28 (-0.83, 0.28)	Tender joint count, BL / 12 weeks, mean (SD)<br>Mediterranean diet: 6.8 (5.9) / 4.5 (5.1)<br>Usual diet: 6.9 (6.3) / 6.1 (6.4); p=0.212		H/UC	H/UC	H/UC	H/UC
	McKellar (2007) [NRT][112]		Tender joint count (28), BL / 6 months, median<br>Mediterranean diet: 5 / 4<br>Healthy eating info: 6 / 6					
Swollen joints	Skoldstam (2003) [RCT][100]	Mediterranean diet vs usual diet<br>SMD -0.43 (-0.98, 0.13)	Swollen joint count, BL / 12 weeks, mean (SD)<br>Mediterranean diet: 7.0 (5.6) / 5.2 (5.1)<br>Usual diet: 6.9 (5.0) / 7.5 (5.7); p=0.001		H/UC	H/UC	H/UC	H/UC
	McKellar (2007) [NRT][112]		Swollen joint count (28), BL / 6 months, median<br>Mediterranean diet: 6 / 4<br>Healthy eating info: 6 / 5					

Alloc. Conc. = allocation concealment, AMSTAR2 = Assessing the Methodological Quality of Systematic Reviews, BL = baseline, Blind. Asses. = Blinded assessors, Blind. Part. = blinded participants, CI = confidence interval, DAS28 = Disease Activity Score (28), H/UC = high / unclear risk of bias, HAQ = Health Assessment Questionnaire, L = low risk of bias, NRT = non-randomised trial, NS = non-significant, RA = rheumatoid arthritis, Rand. Seq. = random sequence generation, RCT = randomised controlled trial, SD = standard deviation, SMD = Standardised mean difference, VAS = visual analogue scale

Table – Mediterranean diet (RA) [cont.], results and quality assessment

Outcome	Study (date) [study type]	Standardised result, SMD (95% CI) unless otherwise stated	Natural result	AMSTAR2 quality	Rand. Seq.	Alloc. Conc.	Blind. Part.	Blind. Asses.
Patient global	Skoldstam (2003) [RCT][100]	Mediterranean diet vs usual diet<br>SMD -0.52 (-1.08, 0.04)	Patient global VAS, BL / 12 weeks, mean (SD)<br>Mediterranean diet: 30 (22) / 18 (13)<br>Usual diet: 28 (20) / 27 (21); p=0.061		H/UC	H/UC	H/UC	H/UC
	McKellar (2007) [NRT][112]		Patient global VAS, BL / 6 months, median<br>Mediterranean diet: 50 / 45<br>Healthy eating info: 54 / 63 p=0.002					
Fatigue	Cramp (2013) [MA][85]	Mediterranean diet vs control<br>SMD 0.37 (-0.18, 0.93)		High				
SF36-mental	Abendroth (2010) [NRT][111]	Mediterranean diet vs fasting<br>SMD -1.18 (-1.79, -0.57)	SF36-mental, BL / 7 days, mean (SD)<br>Mediterranean diet: -1.2 (1.1) / -1.1 (1.1)<br>Fasting: -0.2 (1.1) / 0.1 (0.9)					
Morning stiffness	Skoldstam (2003) [RCT][100]	Mediterranean diet vs usual diet<br>SMD -0.45 (-1.00, 0.11)	Morning stiffness, BL / 12 weeks, mean (SD)<br>Mediterranean diet: 49 (42) / 44 (52)<br>Usual diet: 64 (38) / 70 (64); p=0.367		H/UC	H/UC	H/UC	H/UC
	McKellar (2007) [NRT][112]		Morning stiffness (mins), BL / 6 months, median<br>Mediterranean diet: 30 / 15<br>Healthy eating info: 60 / 30 p=0.041					
CRP	Skoldstam (2003) [RCT][100]	Mediterranean diet vs usual diet<br>SMD -0.22 (-0.77, 0.33)	Morning stiffness, BL / 12 weeks, mean (SD)<br>Mediterranean diet: 17 (20) / 12 (15)<br>Usual diet: 15 (14) / 15 (12)		H/UC	H/UC	H/UC	H/UC
	Abendroth (2010) [NRT][111]	Mediterranean diet vs fasting<br>SMD 0.53 (-0.04, 1.09) in favour of fasting	CRP, BL / 7 days, mean (SD)<br>Mediterranean diet: 2.0 (2.7) / 1.6 (2.2)<br>Fasting: 0.8 (1.0) / 0.7 (0.7)					
	McKellar (2007) [NRT][112]		CRP, BL / 6 months, median<br>Mediterranean diet: 10 / 10<br>Healthy eating info: 8.5 / 8					
ESR	Skoldstam (2003) [RCT][100]	Mediterranean diet vs usual diet<br>SMD 0.00 (-0.55, 0.55)	ESR, BL / 12 weeks, mean (SD)<br>Mediterranean diet: 24 (15) / 25 (15)<br>Usual diet: 23 (15) / 25 (19)		H/UC	H/UC	H/UC	H/UC
	McKellar (2007) [NRT][112]		ESR, BL / 6 months, median<br>Mediterranean diet: 19 / 16<br>Healthy eating info: 19 / 16					
Grip strength	Skoldstam (2003) [RCT][100]	Mediterranean diet vs usual diet<br>SMD -0.08 (-0.63, 0.47)	Grip strength, BL / 12 weeks, mean (SD)<br>Mediterranean diet: 26 (13) / 23 (13)<br>Usual diet: 23 (8) / 24 (11)		H/UC	H/UC	H/UC	H/UC

Alloc. Conc. = allocation concealment, AMSTAR2 = Assessing the Methodological Quality of Systematic Reviews, BL = baseline, Blind. Asses. = Blinded assessors, Blind. Part. = blinded participants, CI = confidence interval, CRP = C-reactive protein, ESR = erythrocyte sedimentation rate, H/UC = high / unclear risk of bias, L = low risk of bias, MA = meta-analysis, NRT = non-randomised trial, NS = non-significant, RA = rheumatoid arthritis, Rand. Seq. = random sequence generation, RCT = randomised controlled trial, SD = standard deviation, SMD = Standardised mean difference, VAS = visual analogue scale

Table – Mediterranean diet, SF36 – results are mean change from baseline to 12 weeks

Author (date)	PCS	MCS	GH	PF	RP	RE	SF	BP	V	MH
Skoldstam (2003) [100] [Mediterranean diet]			5.7 (14.6)	2.5 (15.2)	16.3 (43.6)	9.0 (39.5)	4.8 (19.0)	4.5 (24.3)	11..3 (20.7)	6.5 (16.5)
Skoldstam (2003) [100] [Usual diet]			0.7 (21.7)	1.4 (13.4)	-11.0 (38.2)	1.4 (27.9)	-5.4 (18.8)	4.0 (20.1)	4.2 (16.3)	3.7 (12.9)

BP = bodily pain, GH = general health, MCS = mental component score, MH = mental health, PCS = physical component score, PF = physical function, RE = role emotional, RP = role physical, SF = social functioning, V = vitality

Supplementary table 74 – Vegetarian / vegan diet and RA progression, results

Table – Vegetarian / vegan diet (RA), results and quality assessment

Outcome	Study (date) [study type]	Standardised result, SMD (95% CI) unless otherwise stated	Natural result	AMSTAR2 quality	Rand. Seq.	Alloc. Conc.	Blind. Part.	Blind. Asses.
ACR20	Hafstrom (2001) [RCT][101]		ACR20, N achieved (%) at 12 months<br>Vegan diet: 12 (34.2%)<br>Control: 1 (3.8%) p=0.005§		H/UC	H/UC	H/UC	H/UC
Pain	Skoldstam (1979) [RCT][110]	Lactovegetarian diet vs control, BL-12 weeks<br>SMD -0.32 (-1.11, 0.48)	Pain VAS, BL / change from BL-12 weeks, mean (SD)<br>Lactovegetarian diet: 3.5 (1.9) / -1.2 (3.2)<br>Control: 2.7 (1.7) / -0.3 (2.1)		H/UC	H/UC	H/UC	H/UC
	Kjeldsen-Kragh (1994) [RCT-extension][117]		Pain VAS, 1 year, mean (SD*)<br>Vegetarian – responders: 1.54 (1.33)<br>Vegetarian – non-responders: 5.05 (2.49)<br>Control: 5.84 (2.25)					
	McDougall (2002) [Single arm int.][116]		Pain, BL / 4 weeks, mean (SD)<br>49 (20) / 34 (20), p<0.004					
Function	Skoldstam (1979) [RCT][110]	Lactovegetarian diet vs control, BL-12 weeks<br>SMD 0.18 (-0.62, 0.97)	Functional capacity (0-99), BL / change from BL-12 weeks, mean (SD)<br>Lactovegetarian diet: 31 (3) / 1.2 (7.0)<br>Control: 34 (14) / -1.0 (18.3)		H/UC	H/UC	H/UC	H/UC
	Kjeldsen-Kragh (1994) [RCT-extension][117]		HAQ, 1 year, mean (SD*)<br>Vegetarian – responders: 0.56 (0.51)<br>Vegetarian – non-responders: 1.16 (0.62)<br>Control: 1.06 (0.60)					
	McDougall (2002) [Single arm int.][116]		Function, BL / 4 weeks, mean (SD)<br>47 (25) / 29 (22) p<0.001					
Disease activity	Nenonen (1998) [RCT][104]	Vegan diet vs control at 3 months<br>SMD -0.47 (-1.10, 0.17)	DAS, BL / 3 months, mean (SD†)<br>Vegan diet: 3.26 (0.83) / 3.13 (0.97)<br>Control: 3.44 (1.14) / 3.56 (0.87)		H/UC	H/UC	H/UC	H/UC

§ Calculated by reviewer based on numbers in paper
* Calculated from standard error in the paper
† Calculated from 95% CI in paper
ACR20 = American College of Rheumatology 20 (composite measure of outcome), Alloc. Conc. = allocation concealment, AMSTAR2 = Assessing the Methodological Quality of Systematic Reviews, BL = baseline, Blind. Asses. = Blinded assessors, Blind. Part. = blinded participants, CI = confidence interval, CRP = C-reactive protein, DAS = Disease Activity Score, H/UC = high / unclear risk of bias, HAQ = Health Assessment Questionnaire, int. = intervention, L = low risk of bias, N = number, NRT = non-randomised trial, NS = non-significant, RA = rheumatoid arthritis, Rand. Seq. = random sequence generation, RCT = randomised controlled trial, SD = standard deviation, SMD = Standardised mean difference, VAS = visual analogue scale

Table – Vegetarian / vegan diet (RA) [cont.], results and quality assessment

Outcome	Study (date) [study type]	Standardised result, SMD (95% CI) unless otherwise stated	Natural result	AMSTAR2 quality	Rand. Seq.	Alloc. Conc.	Blind. Part.	Blind. Asses.
Tender joints	Panush (1983) [RCT][108]		Tender joint count, BL / 10 weeks, mean<br>Experimental diet: 28 / 23<br>Placebo diet: 19 / 17, p=NS		H/UC	H/UC	H/UC	H/UC
	Sundqvist (1982) [RCT][109]	Experimental diet vs control at 10 weeks<br>SMD 0.42 (-0.84, 1.68)	Tender joint count, BL / 10 weeks, mean (SD)<br>Experimental diet: 19.8 (2.5) / 18.8 (3.0)<br>control: 16.8 (2.5) / 17.6 (2.7)		H/UC	H/UC	H/UC	H/UC
	Skoldstam (1979) [RCT][110]	Lactovegetarian diet vs control, BL-12 weeks<br>SMD -0.51 (-1.32, 0.29)	Ritchie Index, BL / change from BL-12 weeks, mean (SD)<br>Lactovegetarian diet: 16 (8) / -2.5 (5.6)<br>Control: 13 (5) / 0.2 (4.7)		H/UC	H/UC	H/UC	H/UC
	Kjeldsen-Kragh (1994) [RCT-extension][117]		Tender joint count, 1 year, mean (SD*)<br>Vegetarian – responders: 13.5 (8.2)<br>Vegetarian – non-responders: 22.6 (11.8)<br>Control: 29.6 (9.8)					
	McDougall (2002) [Single arm int.][116]		Joint tenderness, BL / 4 weeks, mean (SD)<br>24 (12) / 17 (16) p<0.01					
Swollen joints	Panush (1983) [RCT][108]		Swollen joint count, BL / 10 weeks, mean<br>Experimental diet: 12 / 9<br>Placebo diet: 13 / 10, p=NS		H/UC	H/UC	H/UC	H/UC
	Kjeldsen-Kragh (1994) [RCT-extension][117]		Swollen joint count, 1 year, mean (SD*)<br>Vegetarian – responders: 5.3 (3.8)<br>Vegetarian – non-responders: 9.5 (6.2)<br>Control: 11.7 (7.8)					
	McDougall (2002) [Single arm int.][116]		Joint swelling, BL / 4 weeks, mean (SD)<br>27 (9) / 22 (8) p<0.02					
Morning stiffness	Panush (1983) [RCT][108]		Morning stiffness, BL / 10 weeks, mean<br>Experimental diet: 80 / 91<br>Placebo diet: 114 / 91, p=NS		H/UC	H/UC	H/UC	H/UC
	Kjeldsen-Kragh (1994) [RCT-extension][117]		Morning stiffness, 1 year, mean (SD*)<br>Vegetarian – responders: 0.77 (1.01)<br>Vegetarian – non-responders: 2.31 (1.94)<br>Control: 2.67 (1.70)					
	McDougall (2002) [Single arm int.][116]		Morning stiffness, BL / 4 weeks, mean (SD)<br>104 (71) / 99 (116), p>0.05					

* Calculated from standard error in the paper

† Calculated from 95% CI in paper

Alloc. Conc. = allocation concealment, AMSTAR2 = Assessing the Methodological Quality of Systematic Reviews, BL = baseline, Blind. Asses. = Blinded assessors, Blind. Part. = blinded participants, CI = confidence interval, H/UC = high / unclear risk of bias, int. = intervention, L = low risk of bias, NS = non-significant, RA = rheumatoid arthritis, Rand. Seq. = random sequence generation, RCT = randomised controlled trial, SD = standard deviation, SMD = Standardised mean difference, VAS = visual analogue scale

Table – Vegetarian / vegan diet (RA) [cont.], results and quality assessment

Outcome	Study (date) [study type]	Standardised result, SMD (95% CI) unless otherwise stated	Natural result	AMSTAR2 quality	Rand. Seq.	Alloc. Conc.	Blind. Part.	Blind. Asses.
Patient global	Panush (1983) [RCT][108]		<u>Patient global VAS, BL / 10 weeks, mean</u><br>Experimental diet: 2.6 / 3.1<br>Placebo diet: 2.6 / 2.7, p=NS		H/UC	H/UC	H/UC	H/UC
	Kjeldsen-Kragh (1994) [RCT-extension][117]		<u>Patient global VAS, 1 year, mean (SD*)</u><br>Vegetarian – responders: 1.7 (1.52)<br>Vegetarian – non-responders: 0.2 (1.11)<br>Control: -0.4 (1.01)					
CRP	Adam (2003) [NRT][113]	<u>Lactovegetarian diet vs control at 3 months</u><br>SMD -0.38 (-0.90, 0.13)	<u>CRP, BL / 3 months, mean (SD)</u><br>Lactovegetarian diet: 1.6 (1.5) / 1.5 (1.6)<br>Control: 2.2 (2.5) / 2.4 (2.9)					
	McDougall (2002) [Single arm int.][116]		<u>CRP, BL / 4 weeks, mean (SD)</u><br>2.08 (1.8) / 1.74 (1.7), p>0.05					

* Calculated from standard error in the paper

Alloc. Conc. = allocation concealment, AMSTAR2 = Assessing the Methodological Quality of Systematic Reviews, BL = baseline, Blind. Asses. = Blinded assessors, Blind. Part. = blinded participants, CI = confidence interval, H/UC = high / unclear risk of bias, L = low risk of bias, NRT = non-randomised trial, NS = non-significant, RA = rheumatoid arthritis, Rand. Seq. = random sequence generation, RCT = randomised controlled trial, SD = standard deviation, SMD = Standardised mean difference, VAS = visual analogue scale

Supplementary table 75 – Description of reviews of fruits, vegetables and other plant based interventions in RA

Table – Fruits, vegetables and other plant based interventions (RA), description of reviews

Authors (date)	Review type	Study type included	Exposure detail	Number of studies included	Funders
Cramp (2013)[85]	MA	RCTs	Andrographis Paniculata	1	**Charity** (Arthritis Research UK)

MA = meta-analysis, RA = rheumatoid arthritis, RCT = randomised controlled trial

Supplementary table 76 – Description of studies of fruits, vegetables and other plant based interventions in RA

Table – Fruits, vegetables and other plant based interventions (RA), description of included studies

Author (date) [country]	Study design	Inclusion criteria	Exposure detail	N	Age, mean (SD) years	N (%) female	Funders
Dawczynski (2017) [Germany][118]	RCT §	2010 ACR/EULAR RA criteria, DAS28>2.4<br>Exclusions: gastrointestinal or metabolic disease, alcohol abuse, dietary supplement intake, known food allergy/intolerance	1) Intervention food projects (sausage, tomato spread, milk powder) enriched with microalgae oil<br>p) Intervention products enriched with sunflower oil	38	61.3 (12.8)	32 (84.2)	**Government** (German Federal Ministry of Education and Research)
Ghavipour (2017) [Iran][119]	RCT	1987 RA criteria, aged ≥40, active RA<br>Exclusions: diabetes, hyperlipidaemia, hypertension, liver disease, kidney disease, severe infections, food intolerance or allergies, alcohol abuse, daily intake of any other drugs or vitamins / mineral supplements	1) Pomegranate extract<br>p) Placebo made from cellulose	1) 30<br>p) 25	1) 48.4 (11.4)<br>p) 49.1 (12.2)	1) 20 (66.7)<br>p) 20 (80.0)	**University** (Shiraz University of Medical Science)
Javadi (2017) [Iran][120]	RCT	1987 ACR RA criteria, aged 19-70 years<br>Exclusions: acute heart, kidney, liver disease, not taking antioxidants, type and dose of medications change in month prior to study, smokers, pregnancy / lactating,	1) Quercetin capsules<br>p) Placebo capsules	1) 20<br>p) 20	1) 46.6 (9.9)<br>p) 48.0 (8.4)	1) 20 (100)<br>p) 20 (100)	**Government** (Iran University of Medical Sciences)
Hemmati (2016) [Iran][121]	RCT	Aged ≥18 years, 2010 RA criteria, symptoms uncontrolled by DMARDs, prednisolone and hydroxychloroquine<br>Exclusions: pregnancy, kidney or liver failure, using other drugs that may affect disease activity	1) Curcumex capsules containing ginger, curcumin and black pepper<br>p) placebo	1) 30<br>p) 30	Not reported	Not reported	**University** (Ahvaz Jundishapur University of Medical Sciences)
Javadi (2014) [Iran][122]	RCT	1987 ACR RA criteria, aged 19-70 years, no changes in treatment<br>Exclusions: other disease that require special treatment or increasing severity of arthritis, smoking, acute illnesses	1) Quercetin capsules<br>p) Placebo capsules	1) 20<br>p) 20	1) 46.6 (9.9)<br>p) 48.0 (8.4)	1) 20 (100)<br>p) 20 (100)	**Government** (Iran University of Medical Sciences)
Willich (2010) [Denmark][123]	RCT	Aged >18 years, 1987 ACR RA criteria<br>Exclusions: Lupus erythematosus, known allergies to plant products, kidney or liver disease, drug abuse, psychiatric disease, pregnancy	1) 10 capsules per day of 0.5g rose hip powder<br>p) Placebo capsules of similar taste	1) 44<br>p) 45	1) 57.0 (10.6)<br>p) 56.1 (12.0)	1) 86%<br>p) 93%	**Industry** (Dansk Droge, Hyben Vital ApS)
Bae (2009) [South Korea][124]	RCT §	1987 ACR RA criteria	1) Quercetin<br>2) Alpha Lipoic acid<br>p) Cornstarch	20	52.1 (10.3)	19 (95.0)	**University** (Sookmyung Women's University Research Grants)

§ Cross-over design

ACR = American College of Rheumatology, DAS28 = Disease Activity Score 28, DMARD = disease modifying anti-rheumatic drug, EULAR = European League Against Rheumatism, N = number, RA = rheumatoid arthritis, RCT = randomised controlled trial, SD = standard deviation

Table – Fruits, vegetables and other plant based interventions (RA) [cont.], description of included studies

Author (date) [country]	Study design	Inclusion criteria	Exposure detail	N	Age, mean (SD) years	N (%) female	Funders
Li (2007) [Hong Kong][125]	RCT	1987 ACR RA criteria, stable sDMARD dose for 3 months<br>Exclusions: 18 years of age, pregnancy, use of intraarticular steroids within 4 weeks of study, any severe chronic or uncontrolled disease, wheelchair bound	1) G Lucidum and San Miao San tablets (Chinese herbal medicine)<br>p) placebo tablets	1) 32<br>p) 33	1) 50 (10)<br>p) 50 (13)	1) 27 (84.4)<br>p) 29 (87.9)	Not reported
Gheita (2012) [Egypt][126]	NRT§	2010 ACR/EULAR criteria	500mg twice daily	40	42.8 (12.5)	40 (100)	Not reported, authors declare no conflicts of interest
Kamal (2018) [Sudan][127]	Single arm int.	Aged 18-70 years, RF and anti-CCP positive, clinical stable, stable treatment<br>Exclusions: Abnormal values of complete blood count, liver function test, renal function test, hepatic disease, infectious or autoimmune liver disease, chronic kidney disease, chronic respiratory disease, malignancy, connective tissue disease	1) Gum Arabic powder mixed into 200ml water and consumed in the morning	40	Men: 47.8 (2.8)<br>Women: 55 (2.8)	38 (95)	**University** (University of Khartoum)
Kumar (2015) [India][128]	Single arm int.	Aged 18-60 years, 1987 ACR RA criteria<br>Exclusions: unstable angina, myocardial infarction, heart failure or stroke, uncontrolled hypertension, uncontrolled diabetes, ALT or AST >2x ULN, impaired renal function, pregnancy / lactation, patients taking other Ayurvedic drugs	1) Ashwagandha powder mixed with water for 3 weeks, then Sidh Makardhwag with honey for 4 weeks	78	Women: 45.7 (8.6)<br>Men: 49.8 (7.9)	45 (52.3)	**Government** (Central Council for Research in Ayurveda and Sidha (CCRAS), Department of AYUSH, Ministry of Health and Family Welfare, Government of India)
Matsuno (2009) [Japan][129]	Single arm int.	1987 ACR RA criteria<br>Exclusions: history of synovial fluid drainage, intra-articular steroid in previous 2 months before baseline	Quercetin, glucosamine and chondroitin together	22	58.0 (10.0)	20 (90.9)	Not reported

§ Cross-over design

ACR = American College of Rheumatology, ALT = alanine aminotransferase, Anti-CCP = anti-cyclic citrullinated peptide, AST = Aspartate transaminase, DMARD = disease modifying anti-rheumatic drug, EULAR = European League Against Rheumatism , N = number, NRT = non-randomised trial, RA = rheumatoid arthritis, RCT = randomised controlled trial, RF = rheumatoid factor, SD = standard deviation

Supplementary table 77 – Andrographis Paniculata and RA progression, results

Table – Andrographis Paniculata (RA), results and quality assessment

Outcome	Study (date) [study type]	Standardised result, SMD (95% CI) unless otherwise stated	Natural result	AMSTAR2 quality	Rand. Seq.	Alloc. Conc.	Blind. Part.	Blind. Asses.
Fatigue	Cramp (2013) [MA][85]	Andrographis Paniculata vs placebo<br>SMD -0.25 (-0.77, 0.27)		High				

Alloc. Conc. = allocation concealment, AMSTAR2 = Assessing the Methodological Quality of Systematic Reviews, Blind. Asses. = Blinded assessors, Blind. Part. = blinded participants, CI = confidence interval, H/UC = high / unclear risk of bias, L = low risk of bias, MA = meta-analysis, RA = rheumatoid arthritis, Rand. Seq. = random sequence generation, SMD = Standardised mean difference,

Supplementary table 78 – Ginger / curcumin / black pepper and RA progression, results

Table – Ginger / curcumin / black pepper (RA), results and quality assessment

Outcome	Study (date) [study type]	Standardised result, SMD (95% CI) unless otherwise stated	Natural result	AMSTAR2 quality	Rand. Seq.	Alloc. Conc.	Blind. Part.	Blind. Asses.
Disease activity	Hemmati (2016) [RCT][121]	Curcumex vs placebo at 8 weeks<br>SMD -2.74 (-3.45, -2.03)	DAS28 at 8 weeks, mean (SD)<br>Curcumex: 3.29 (0.89)<br>Placebo: 5.51 (0.72); p<0.001		H/UC	H/UC	H/UC	H/UC
Tender joints	Hemmati (2016) [RCT][121]	Curcumex vs placebo at 8 weeks<br>SMD -2.75 (-3.46, -2.03)	Tender joint count at 8 weeks, mean (SD)<br>Curcumex: 2.27 (1.96)<br>Placebo: 10.33 (3.66); p<0.001		H/UC	H/UC	H/UC	H/UC
Swollen joints	Hemmati (2016) [RCT][121]	Curcumex vs placebo at 8 weeks<br>SMD -2.14 (-2.77, -1.50)	Swollen joint count at 8 weeks, mean (SD)<br>Curcumex: 1.07 (1.17)<br>Placebo: 7.13 (3.84); p<0.001		H/UC	H/UC	H/UC	H/UC
ESR	Hemmati (2016) [RCT][121]	Curcumex vs placebo at 8 weeks<br>SMD -1.05 (-1.60, -0.51)	ESR at 8 weeks, mean (SD)<br>Curcumex: 21.50 (12.67)<br>Placebo: 38.47 (18.92); p<0.001		H/UC	H/UC	H/UC	H/UC

Alloc. Conc. = allocation concealment, AMSTAR2 = Assessing the Methodological Quality of Systematic Reviews, Blind. Asses. = Blinded assessors, Blind. Part. = blinded participants, CI = confidence interval, DAS28 = Disease Activity Score 28, ESR = erythrocyte sedimentation rate, H/UC = high / unclear risk of bias, L = low risk of bias, RA = rheumatoid arthritis, Rand. Seq. = random sequence generation, RCT = randomised controlled trial, SD = standard deviation, SMD = Standardised mean difference

Supplementary table 79 – Gum Arabic and RA progression, results

Table – Gum Arabic (RA), results and quality assessment

Outcome	Study (date) [study type]	Standardised result, SMD (95% CI) unless otherwise stated	Natural result	AMSTAR2 quality	Rand. Seq.	Alloc. Conc.	Blind. Part.	Blind. Asses.
Disease activity	Kamal (2018) [single arm int.][127]		DAS28, BL / 12 weeks, mean (SD)<br>5.43 (1.49) / 3.8 (1.26), p<0.01					
Tender joints	Kamal (2018) [single arm int.][127]		Tender joint count, BL / 12 weeks, mean (SD)<br>10.66 (9.6) / 2.97 (6.03), p<0.01					
Swollen joints	Kamal (2018) [single arm int.][127]		Swollen joint count, BL / 12 weeks, mean (SD)<br>5.4 (6.5) / 2.05 (4.7), p<0.01					
Patient global	Kamal (2018) [single arm int.][127]		Patient global VAS, BL / 12 weeks, mean (SD)<br>4.85 (2.17) / 2.1 (1.9) p<0.01					

Alloc. Conc. = allocation concealment, AMSTAR2 = Assessing the Methodological Quality of Systematic Reviews, BL = baseline, Blind. Asses. = Blinded assessors, Blind. Part. = blinded participants, CI = confidence interval, DAS28 = Disease Activity Score 28, H/UC = high / unclear risk of bias, int. = intervention, L = low risk of bias, RA = rheumatoid arthritis, Rand. Seq. = random sequence generation, SD = standard deviation, SMD = Standardised mean difference, VAS = visual analogue scale

Supplementary table 80 – Herbal medicine and RA progression, results

Table – Herbal medicine (RA), results and quality assessment

Outcome	Study (date) [study type]	Standardised result, SMD (95% CI) unless otherwise stated	Natural result	AMSTAR2 quality	Rand. Seq.	Alloc. Conc.	Blind. Part.	Blind. Asses.
Pain	Li (2007) [RCT][125]	Herbal medicine vs placebo at 6 months<br>SMD -0.25 (-0.74, 0.24)	Pain VAS, BL / 6 months, mean (SD)<br>Herbal medicine: 4.9 (2.3) / 3.9 (2.5)<br>Placebo: 4.8 (2.4) / 4.5 (2.3)		L	L	L	L
	Kumar (2015) [single arm int.][128]		Pain VAS, BL / 7 weeks, mean (SD)<br>Men: 6.2 (0.7) / 4.4 (0.4)<br>Women: 6.2 (0.7) / 4.4 (0.5)					
Function	Li (2007) [RCT][125]	Herbal medicine vs placebo at 6 months<br>SMD 0.14 (-0.34, 0.63)	HAQ, BL / 6 months, mean (SD*)<br>Herbal medicine: 1.2 (0.8) / 1.3 (0.7)<br>Placebo: 1.1 (0.8) / 1.2 (0.7)		L	L	L	L
	Kumar (2015) [single arm int.][128]		Disability index, BL / 7 weeks, mean (SD)<br>Men: 3.3 (1.1) / 2.5 (0.9)<br>Women: 3.3 (1.3) / 2.6 (0.9)					
Disease activity	Kumar (2015) [single arm int.][128]		DAS28, BL / 7 weeks, mean (SD)<br>Men: 5.0 (0.4) / 4.3 (0.2)<br>Women: 5.1 (0.3) / 4.3 (0.2)					
Tender joints	Li (2007) [RCT][125]	Herbal medicine vs placebo at 6 months<br>SMD -0.08 (-0.56, 0.41)	Tender joint count, BL / 6 months, mean (SD*)<br>Herbal medicine: 2.7 (3.1) / 2.0 (3.1)<br>Placebo: 2.3 (0.8) / 2.3 (4.6)		L	L	L	L
	Kumar (2015) [single arm int.][128]		Tender joint count, BL / 7 weeks, mean (SD)<br>Men: 6.6 (1.3) / 4.8 (0.8)<br>Women: 6.6 (1.2) / 4.8 (0.6)					
Swollen joints	Li (2007) [RCT][125]	Herbal medicine vs placebo at 6 months<br>SMD -0.18 (-0.67, 0.31)	Swollen joint count, BL / 6 months, mean (SD*)<br>Herbal medicine: 3.3 (2.3) / 4.0 (3.1)<br>Placebo: 3.7 (3.1) / 4.7 (4.6)		L	L	L	L
	Kumar (2015) [single arm int.][128]		Swollen joint count, BL / 7 weeks, mean (SD)<br>Men: 3.4 (1.7) / 2.5 (1.0)<br>Women: 3.9 (1.8) / 2.7 (1.0)					
Patient global	Li (2007) [RCT][125]	Herbal medicine vs placebo at 6 months<br>SMD -0.04 (-0.53, 0.45)	Patient global VAS, BL / 6 months, mean (SD)<br>Herbal medicine: 5.7 (2.5) / 4.7 (2.6)<br>Placebo: 5.4 (2.3) / 4.8 (2.5)		L	L	L	L
	Kumar (2015) [single arm int.][128]		Patient global VAS, BL / 7 weeks, mean (SD)<br>Men: 52.1 (11.1) / 35.2 (8.0)<br>Women: 53.6 (11.5) / 34.4 (7.9)					

* mean (SD) calculated from median (IQR) using publish formula[61]

Alloc. Conc. = allocation concealment, AMSTAR2 = Assessing the Methodological Quality of Systematic Reviews, BL = baseline, Blind. Asses. = Blinded assessors, Blind. Part. = blinded participants, CI = confidence interval, DAS28 = Disease Activity Score 28, H/UC = high / unclear risk of bias, HAQ = Health Assessment Questionnaire, L = low risk of bias, RA = rheumatoid arthritis, Rand. Seq. = random sequence generation, RCT = randomised controlled trial, SD = standard deviation, SMD = Standardised mean difference, VAS = visual analogue scale

Table – Herbal medicine (RA) [cont.], results and quality assessment

Outcome	Study (date) [study type]	Standardised result, SMD (95% CI) unless otherwise stated	Natural result	AMSTAR2 quality	Rand. Seq.	Alloc. Conc.	Blind. Part.	Blind. Asses.
ESR	Li (2007) [RCT][125]	Herbal medicine vs placebo at 6 months<br>SMD -0.44 (-0.93, 0.05)	ESR, BL / 6 months, mean (SD*)<br>Herbal medicine: 37.3 (21.7) / 36.0 (28.7)<br>Placebo: 46 (44.9) / 49.7 (33.3)		L	L	L	L
	Kumar (2015) [single arm int.][128]		ESR, BL / 7 weeks, mean (SD)<br>Men: 28.8 (3.3) / 21.6 (1.9)<br>Women: 31.2 (3.1) / 22.1 (1.4)					
CRP	Li (2007) [RCT][125]	Herbal medicine vs placebo at 6 months<br>SMD -0.28 (-0.77, 0.21)	CRP, BL / 6 months, mean (SD*)<br>Herbal medicine: 11.5 (14.9) / 9.9 (9.7)<br>Placebo: 15.3 (23.2) / 13.0 (12.1)		L	L	L	L

* mean (SD) calculated from median (IQR) using publish formula[61]

Alloc. Conc. = allocation concealment, AMSTAR2 = Assessing the Methodological Quality of Systematic Reviews, BL = baseline, Blind. Asses. = Blinded assessors, Blind. Part. = blinded participants, CI = confidence interval, CRP = C-reactive protein, ESR = erythrocyte sedimentation rate, H/UC = high / unclear risk of bias, IQR = interquartile range, L = low risk of bias, RA = rheumatoid arthritis, Rand. Seq. = random sequence generation, RCT = randomised controlled trial, SD = standard deviation, SMD = Standardised mean difference

Supplementary table 81 – Microalgae oil and RA progression, results

Table – Microalgae oil (RA), results and quality assessment

Outcome	Study (date) [study type]	Standardised result, SMD (95% CI) unless otherwise stated	Natural result	AMSTAR2 quality	Rand. Seq.	Alloc. Conc.	Blind. Part.	Blind. Asses.
Function	Dawczynski (2017) [RCT][118]	Microalgae oil vs placebo at 10 weeks<br>SMD -0.26 (-0.72, 0.19)	HAQ at 10 weeks, mean (SD)<br>Microalgae oil: 1.07 (0.64)<br>Placebo: 1.26 (0.79)		H/UC	H/UC	L	H/UC
Disease activity	Dawczynski (2017) [RCT][118]	Microalgae oil vs placebo at 10 weeks<br>SMD -0.21 (-0.66, 0.24)	DAS28 at 10 weeks, mean (SD)<br>Microalgae oil: 3.88 (1.17)<br>Placebo: 4.13 (1.2)		H/UC	H/UC	L	H/UC
Tender joints	Dawczynski (2017) [RCT][118]	Microalgae oil vs placebo at 10 weeks<br>SMD -0.42 (-0.87, 0.04)	Tender joint count (66) at 10 weeks, mean (SD)<br>Microalgae oil: 6.00 (5.01)<br>Placebo: 8.79 (8.05)		H/UC	H/UC	L	H/UC
Swollen joints	Dawczynski (2017) [RCT][118]	Microalgae oil vs placebo at 10 weeks<br>SMD -0.08 (-0.53, 0.37)	Swollen joint count (66) at 10 weeks, mean (SD)<br>Microalgae oil: 3.92 (3.49)<br>Placebo: 4.21 (3.72)		H/UC	H/UC	L	H/UC
Morning stiffness	Dawczynski (2017) [RCT][118]	Microalgae oil vs placebo at 10 weeks<br>SMD -0.25 (-0.70, 0.20)	Morning stiffness at 10 weeks, mean (SD)<br>Microalgae oil: 27.2 (30.7)<br>Placebo: 35.8 (37.1)		H/UC	H/UC	L	H/UC
Patient global	Dawczynski (2017) [RCT][118]	Microalgae oil vs placebo at 10 weeks<br>SMD -0.27 (-0.72, 0.19)	Patient global VAS at 10 weeks, mean (SD)<br>Microalgae oil: 42.8 (22.33)<br>Placebo: 38.67 (20.31)		H/UC	H/UC	L	H/UC
CRP	Dawczynski (2017) [RCT][118]	Microalgae oil vs placebo at 10 weeks<br>SMD 0.16 (-0.29, 0.61)	CRP at 10 weeks, mean (SD)<br>Microalgae oil: 7.57 (7.62)<br>Placebo: 6.51 (5.58)		H/UC	H/UC	L	H/UC
ESR	Dawczynski (2017) [RCT][118]	Microalgae oil vs placebo at 10 weeks<br>SMD 0.05 (-0.40, 0.50)	ESR at 10 weeks, mean (SD)<br>Microalgae oil: 26.9 (21.7)<br>Placebo: 25.8 (20.7)		H/UC	H/UC	L	H/UC
Erosions	Dawczynski (2017) [RCT][118]	Microalgae oil vs placebo at 10 weeks<br>SMD 0.00 (-0.45, 0.45)	erosions at 10 weeks, mean (SD)<br>Microalgae oil: 2.78 (2.47)<br>Placebo: 2.78 (2.58)		H/UC	H/UC	L	H/UC

Alloc. Conc. = allocation concealment, AMSTAR2 = Assessing the Methodological Quality of Systematic Reviews, Blind. Asses. = Blinded assessors, Blind. Part. = blinded participants, CI = confidence interval, CRP = C-reactive protein, DAS28 = Disease Activity Score 28, ESR = erythrocyte sedimentation rate, H/UC = high / unclear risk of bias, HAQ = Health Assessment Questionnaire, L = low risk of bias, RA = rheumatoid arthritis, Rand. Seq. = random sequence generation, RCT = randomised controlled trial, SD = standard deviation, SMD = Standardised mean difference, VAS = visual analogue scale

Supplementary table 82 – Nigella Sativa oil and RA progression, results

Table – Nigella Sativa oil (RA), results and quality assessment

Outcome	Study (date) [study type]	Standardised result, SMD (95% CI) unless otherwise stated	Natural result	AMSTAR2 quality	Rand. Seq.	Alloc. Conc.	Blind. Part.	Blind. Asses.
Pain	Gheita (2012) [NRT§][126]	Nigella Sativa oil vs placebo after each period<br>SMD -0.47 (-0.91, -0.02)	Pain VAS, BL/after placebo/after intervention, mean (SD)<br>60.25 (12.71) / 60.25 (12.71) / 52.75 (18.81)					
Disease activity	Gheita (2012) [NRT§][126]	Nigella Sativa oil vs placebo after each period<br>SMD -0.57 (-1.02, -0.12)	DAS28, BL/after placebo/after intervention, mean (SD)<br>4.98 (0.79) / 4.99 (0.72) / 4.55 (0.82)					
Tender joints	Gheita (2012) [NRT§][126]	Nigella Sativa oil vs placebo after each period<br>SMD -0.53 (-0.97, -0.08)	Ritchie Index, BL/after placebo/after intervention, mean (SD)<br>6.58 (4.17) / 6.43 (3.88) / 4.68 (2.66)					
Swollen joints	Gheita (2012) [NRT§][126]	Nigella Sativa oil vs placebo after each period<br>SMD -0.92 (-1.38, -0.46)	Swollen joint count, BL/after placebo/after intervention, mean (SD)<br>2.4 (1.17) / 2.3 (1.14) / 1.35 (0.92)					
Morning stiffness	Gheita (2012) [NRT§][126]	Nigella Sativa oil vs placebo after each period<br>SMD -0.63 (-1.08, -0.18)	Morning stiffness, BL/after placebo/after intervention, mean (SD)<br>30.63 (28.04) / 30.63 (28.04) / 17.13 (11.6)					
ESR	Gheita (2012) [NRT§][126]	Nigella Sativa oil vs placebo after each period<br>SMD -0.23 (-0.67, 0.21)	ESR, BL/after placebo/after intervention, mean (SD)<br>36.25 (18.43) / 36.48 (18.6) / 32.75 (13.38)					

§ The study design had all patients taking a placebo for 1 months followed by the intervention

Alloc. Conc. = allocation concealment, AMSTAR2 = Assessing the Methodological Quality of Systematic Reviews, BL = baseline, Blind. Asses. = Blinded assessors, Blind. Part. = blinded participants, CI = confidence interval, DAS28 = Disease Activity Score 28, ESR = erythrocyte sedimentation rate, H/UC = high / unclear risk of bias, L = low risk of bias, NRT = non-randomised trial, RA = rheumatoid arthritis, Rand. Seq. = random sequence generation, SD = standard deviation, SMD = Standardised mean difference, VAS = visual analogue scale

Supplementary table 83 – Pomegranate and RA progression, results

Table – Pomegranate (RA), results and quality assessment

Outcome	Study (date) [study type]	Standardised result, SMD (95% CI) unless otherwise stated	Natural result	AMSTAR2 quality	Rand. Seq.	Alloc. Conc.	Blind. Part.	Blind. Asses.
Pain	Ghavipour (2017) [RCT][119]	Pomegranate vs placebo, change from BL to 56 days<br>SMD -0.15 (-0.68, 0.38)	Pain VAS, BL / change at 56 days, mean (SD*)<br>Pomegranate: 59.3 (143.0) / -17.6 (136.4)<br>Placebo: 51.0 (124.5) / -1.6 (51.0); p=0.003		L	L	L	L
Function	Ghavipour (2017) [RCT][119]	Pomegranate vs placebo, change from BL to 56 days<br>SMD -0.16 (-0.69, 0.38)	HAQ, BL / change at 56 days, mean (SD*)<br>Pomegranate: 1.2 (3.3) / -0.4 (2.2)<br>Placebo: 1.3 (3.5) / -0.1 (1.5); p=0.007		L	L	L	L
Tender joints	Ghavipour (2017) [RCT][119]	Pomegranate vs placebo, change from BL to 56 days<br>SMD -0.17 (-0.71, 0.35)	Tender joint count, BL / change at 56 days, mean (SD*)<br>Pomegranate: 5.8 (21.2) / -2.1 (17.0)<br>Placebo: 7.0 (27.0) / 0.9 (16.5); p=0.001		L	L	L	L
Swollen joints	Ghavipour (2017) [RCT][119]	Pomegranate vs placebo, change from BL to 56 days<br>SMD -0.22 (-0.75, 0.31)	Swollen joint count, BL / change at 56 days, mean (SD*)<br>Pomegranate: 5.7 (17.0) / -2.6 (14.8)<br>Placebo: 4.4 (13.5) / 0.08 (8.0); p<0.001		L	L	L	L
CRP	Ghavipour (2017) [RCT][119]	Pomegranate vs placebo, change from BL to 56 days<br>SMD -0.06 (-0.59, 0.47)	CRP, BL / change at 56 days, mean (SD*)<br>Pomegranate: 8.0 (23.0) / -0.8 (17.0)<br>Placebo: 6.6 (22.5) / 0.4 (23.5); p=0.6		L	L	L	L
ESR	Ghavipour (2017) [RCT][119]	Pomegranate vs placebo, change from BL to 56 days<br>SMD -0.11 (-0.64, 0.42)	ESR, BL / change at 56 days, mean (SD*)<br>Pomegranate: 29.0 (85.4) / -4.3 (60.2)<br>Placebo: 30.6 (98.0) / 3.5 (79.5);p =0.03		L	L	L	L

* Calculated from standard error in paper. Concern that there is a miss-print in the paper and this is in fact the standard deviation.

Alloc. Conc. = allocation concealment, AMSTAR2 = Assessing the Methodological Quality of Systematic Reviews, BL = baseline, Blind. Asses. = Blinded assessors, Blind. Part. = blinded participants, CI = confidence interval, CRP = C-reactive protein, ESR = erythrocyte sedimentation rate, H/UC = high / unclear risk of bias, HAQ = Health Assessment Questionnaire, L = low risk of bias, RA = rheumatoid arthritis, Rand. Seq. = random sequence generation, RCT = randomised controlled trial, SD = standard deviation, SMD = Standardised mean difference, VAS = visual analogue scale

Supplementary table 84 – Quercetin and RA progression, results

Table – Quercetin (RA), results and quality assessment

Outcome	Study (date) [study type]	Standardised result, SMD (95% CI) unless otherwise stated	Natural result	AMSTAR2 quality	Rand. Seq.	Alloc. Conc.	Blind. Part.	Blind. Asses.
Pain	Javadi (2017) [RCT][120]	Quercetin vs placebo at week 8<br>SMD -0.85 (-1.50, -0.20)	Morning pain VAS, BL / 8 weeks, mean (SD)<br>Quercetin: 36.7 (19.1) / 21.5 (15.9)<br>Placebo: 35.1 (24.4) / 40.3 (27.0); p=0.01		L	L	L	L
	Bae (2009) [RCT][124]	Quercetin vs placebo at week 4<br>SMD -0.10 (-0.73, 0.52)	Pain VAS, BL / 4 weeks, mean (SD*)<br>Quercetin: 28.75 (19.95) / 30.00 (31.91)<br>Placebo: 32.25 (27.92) / 33.33 (31.91); p=0.34		H/UC	H/UC	L	H/UC
	Matsuno (2009) [single arm int.][129]		Pain, BL / 3 months, mean (SD)<br>32.5 (25.4) / 27.4 (20.9) p=0.32					
	Bespoke MA<br>Javadi (2017)[120]<br>Bae (2017)[124]	Quercetin vs placebo<br>SMD -0.47 (-1.20, 0.26), I^2 62.1%						
Function	Javadi (2017) [RCT][120]	Quercetin vs placebo at week 8<br>SMD -0.94 (-1.60, -0.29)	HAQ, BL / 8 weeks, mean (SD)<br>Quercetin: 0.59 (0.37) / 0.35 (0.28)<br>Placebo:0.67 (0.42) / 0.68 (0.41); p=0.008		L	L	L	L
	Bae (2017) [RCT][124]	Quercetin vs placebo at week 4<br>SMD -0.43 (-1.06, 0.20)	KHAQ, BL / 4 weeks, mean (SD*)<br>Quercetin: 0.42 (0.56) / 0.36 (0.40)<br>Placebo: 0.47 (0.40) / 0.59 (0.64); p=0.25		H/UC	H/UC	L	H/UC
	Bespoke MA<br>Javadi (2017)[120]<br>Bae (2017)[124]	Quercetin vs placebo<br>SMD -0.68 (-1.18, -0.18), I^2 17.4%						
Disease activity	Javadi (2017) [RCT][120]	Quercetin vs placebo at week 8<br>SMD -0.40 (-1.03, 0.23)	DAS28, BL / 8 weeks, mean (SD)<br>Quercetin: 3.22 (0.93) / 2.65 (0.98)<br>Placebo: 3.13 (1.10) / 3.11 (1.29); p=0.04		L	L	L	L
Tender joints	Javadi (2017) [RCT][120]	Quercetin vs placebo at week 8<br>SMD -0.40 (-1.02, 0.23)	Tender joint count, BL / 8 weeks, mean (SD*)<br>Quercetin: 1.3 (2.2) / 0.3 (0.8)<br>Placebo: 0.8 (1.6) / 0.8 (1.6); p=0.33		L	L	L	L
Swollen joints	Javadi (2017) [RCT][120]	Quercetin vs placebo at week 8<br>SMD -0.24 (-0.86, 0.39)	Swollen joint count, BL / 8 weeks, mean (SD*)<br>Quercetin: 0.7 (1.6) / 0.3 (0.8)<br>Placebo: 0.7 (0.8) / 0.6 (1.6); p=0.36		L	L	L	L
	Matsuno (2009) [single arm int.][129]		Joint swelling score, BL / 3 months, mean (SD)<br>46.1 (22.1) / 39.7 (25.3) p=0.35					
Morning stiffness	Javadi (2017) [RCT][120]		Morning stiffness, BL / 8 weeks, mean (SD*)<br>Quercetin: 10.4 (18.9) / 0 (0)<br>Placebo: 11.3 (18.9) / 8.2 (16.0)		L	L	L	L
	Matsuno (2009) [single arm int.][129]		Morning stiffness, BL / 3 months, mean (SD)<br>94.6 (168.4) / 89.4 (171.8) p=0.97					

* mean (SD) calculated from median (IQR) using published formula[61], Alloc. Conc. = allocation concealment, AMSTAR2 = Assessing the Methodological Quality of Systematic Reviews, BL = baseline, Blind. Asses. = Blinded assessors, Blind. Part. = blinded participants, CI = confidence interval, DAS28 = Disease Activity Score 28, H/UC = high / unclear risk of bias, HAQ = Health Assessment Questionnaire, IQR = interquartile range, MA = meta-analysis, KHAQ = Korean Health Assessment Questionnaire, L = low risk of bias, RA = rheumatoid arthritis, Rand. Seq. = random sequence generation, RCT = randomised controlled trial, SD =standard deviation, SMD = Standardised mean difference, VAS = visual analogue scale

Table – Quercetin (RA) [cont.], results and quality assessment

Outcome	Study (date) [study type]	Standardised result, SMD (95% CI) unless otherwise stated	Natural result	AMSTAR2 quality	Rand. Seq.	Alloc. Conc.	Blind. Part.	Blind. Asses.
CRP	Javadi (2014) [RCT][122]	Quercetin vs placebo at week 8<br>SMD -0.21 (-0.83, 0.41)	CRP, BL / 8 weeks, mean (SD)<br>Quercetin: 2.9 (3.0) / 2.2 (2.3)<br>Placebo: 3.3 (2.3) / 2.7 (2.4); p=NS		H/UC	H/UC	L	H/UC
	Bae (2009) [RCT][124]	Quercetin vs placebo at week 4<br>SMD -0.22 (-0.84, 0.40)	CRP, BL / 4 weeks, mean (SD*)<br>Quercetin: 2.57 (4.96) / 1.63 (2.56)<br>Placebo: 1.71 (2.97) / 2.33 (3.71)		H/UC	H/UC	L	H/UC
	Matsuno (2009) [single arm int.][129]		CRP, BL / 3 months, mean (SD)<br>2.8 (2.4) / 3.3 (2.7) p=0.30					
	Bespoke MA<br>Javadi (2014)[122]<br>Bae (2017)[124]	Quercetin vs placebo<br>SMD -0.22 (-0.66, 0.22), I^2 0%						
ESR	Javadi (2017) [RCT][120]	Quercetin vs placebo at week 8<br>SMD -0.36 (-0.99, 0.26)	ESR, BL / 8 weeks, mean (SD)<br>Quercetin: 19.0 (8.6) / 16.9 (9.6)<br>Placebo: 21.1 (12.4) / 22.0 (17.5); p=0.35		L	L	L	L
	Matsuno (2009) [single arm int.][129]		ESR, BL / 3 months, mean (SD)<br>66.0 (27.7) / 69.2 (28.7); p=0.46					

* mean (SD) calculated from median (IQR) using published formula[61]

Alloc. Conc. = allocation concealment, AMSTAR2 = Assessing the Methodological Quality of Systematic Reviews, BL = baseline, Blind. Asses. = Blinded assessors, Blind. Part. = blinded participants, CI = confidence interval, CRP = C-reactive protein, ESR = erythrocyte sedimentation rate, H/UC = high / unclear risk of bias, IQR = interquartile range, L = low risk of bias, MA = meta-analysis, RA = rheumatoid arthritis, Rand. Seq. = random sequence generation, RCT = randomised controlled trial, SD =standard deviation, SMD = Standardised mean difference

Supplementary table 85 – Rose hip and RA progression, results

Table – Rose hip (RA), results and quality assessment

Outcome	Study (date) [study type]	Standardised result, SMD (95% CI) unless otherwise stated	Natural result	AMSTAR2 quality	Rand. Seq.	Alloc. Conc.	Blind. Part.	Blind. Asses.
Pain	Willich (2010) [RCT][123]	Rose hip vs placebo<br>SMD -0.25 (-0.67, 0.17)	Pain VAS, BL / 6 months, mean (SD)<br>Rose hip: 44.73 (22.75) / 39.82 (23.44)<br>Placebo: 45.56 (21.98) / 45.71 (23.47)		L	H/UC	L	H/UC
Function	Willich (2010) [RCT][123]	Rose hip vs placebo<br>SMD -0.18 (-0.60, 0.24)	HAQ, BL / 6 months, mean (SD)<br>Rose hip: 1.13 (0.55) / 1.03 (0.58)<br>Placebo: 1.11 (0.76) / 1.15 (0.74)		L	H/UC	L	H/UC
Disease activity	Willich (2010) [RCT][123]	Rose hip vs placebo<br>SMD -0.36 (-0.78, 0.06)	DAS28, BL / 6 months, mean (SD)<br>Rose hip: 4.82 (1.33) / 3.93 (1.56)<br>Placebo: 4.71 (1.01) / 4.42 (1.17)		L	H/UC	L	H/UC
Patient global	Willich (2010) [RCT][123]	Rose hip vs placebo<br>SMD -0.31 (-0.73, 0.11)	Patient global VAS, BL / 6 months, mean (SD)<br>Rose hip: 47.55 (25.96) / 39.57 (25.01)<br>Placebo: 47.13 (21.28) / 47.18 (24.13)		L	H/UC	L	H/UC
QoL	Willich (2010) [RCT][123]	Rose hip vs placebo<br>SMD -0.13 (-0.55, 0.29)	RAQOL, BL / 6 months, mean (SD)<br>Rose hip: 11.57 (6.36) / 10.18 (7.22)<br>Placebo: 10.87 (6.68) / 11.09 (6.89)		L	H/UC	L	H/UC
Mental Health	Willich (2010) [RCT][123]	Rose hip vs placebo<br>SMD -0.02 (-0.43, 0.40)	SF-12 Mental, BL / 6 months, mean (SD)<br>Rose hip: 49.30 (10.44) / 48.46 (10.85)<br>Placebo: 49.13 (9.34) / 48.64 (9.46)		L	H/UC	L	H/UC

Alloc. Conc. = allocation concealment, AMSTAR2 = Assessing the Methodological Quality of Systematic Reviews, BL = baseline, Blind. Asses. = Blinded assessors, Blind. Part. = blinded participants, CI = confidence interval, DAS28 = Disease Activity Score 28, H/UC = high / unclear risk of bias, HAQ = Health Assessment Questionnaire, L = low risk of bias, QoL = Quality of life, RA = rheumatoid arthritis, RAQOL = Rheumatoid Arthritis Quality of Life Measure, Rand. Seq. = random sequence generation, RCT = randomised controlled trial, SMD = Standardised mean difference, VAS = visual analogue scale

Supplementary table 86 – Description of reviews of minerals and supplements in RA

Table – Minerals and supplements (RA), description of reviews

Authors (date)	Review type	Study type included	Exposure detail	Number of studies included	Funders
Aqaeinezhad Rudbane (2018) [Iran][130]	MA	RCTs	Probiotics	5	**University** (Shiraz University of Medical Sciences)
Mohammed et al (2017) [Egypt][131]	MA	RCTs	Probiotics	6	Not reported, authors declare no conflict of interest

MA = meta-analysis, RA = randomised controlled trial, RCT = randomised controlled trial, SR = systematic review

Supplementary table 87 – Description of studies of minerals and supplements in RA

Table – Minerals and supplements (RA), description of included studies

Author (date) [country]	Study design	Inclusion criteria	Exposure detail	N	Age, mean (SD) years	N (%) female	Funders
Zamani (2017) [Iran][132]	RCT	1987 ACR RA, symptom duration >6 months, DAS28>3.2, aged 25-70 years<br>Exclusions: chronic renal failure, pregnancy / lactation, symptoms or history of cardiovascular disease, diabetes, consumption of antihyperglycaemic agents including metformin, unable to read numbers / mark scales, unlikely to come to follow-up, taking probiotics / synbiotics, antioxidants and/or anti-inflammatory supplements such as vitamin E, vitamin C, taking antibiotics	1) synbiotic supplements - Lactobacillus acidophilus, Lactobacillus casei and Bifidobacterium bifidum<br>p) Placebo (starch)	1) 27<br>p) 27	1) 49.3 (11.0)<br>p) 49.5 (12.9)	1) 22 (81.5)<br>p) 24 (88.9)	**University** (Vice-chancellor for Research, KUMS and Iran)
Wilkinson (2016) [UK][133]	RCT	2010 ACR/EULAR RA criteria, aged ≥18 years, stable medication for 3 months, not cognitively impaired, free from cachectic conditions, have an eGFR ≥60, no anabolic supplementation, no regular high-intensity exercise, not pregnant	1) Drink containing creatine<br>p) Drink containing placebo	1) 15<br>p) 20	1) 63.0 (10.0)<br>p) 57.2 (10.4)	1) 10 (66.7)<br>p) 14 (70.0)	**University** (Betsi Cadwaladr University Health Board Small Grants Committee)
Abdollahzad (2015) [Iran][134]	RCT	Aged 18-65 years, DAS28>3.2, 1987 ACR RA criteria<br>Exclusions: liver, kidney, diabetes, RA symptom duration <6 months, consumption of other antioxidants or fatty acid supplements one month before BL, smoking, warfarin, pregnancy/lactation, oral contraceptives	1) Co-enzyme Q10<br>p) Wheat starch placebo	1) 22<br>p) 23	1) 48.8 (11.6)<br>p) 50.6 (11.1)	1) 19 (86.4)<br>p) 20 (87.0)	**University** (Tabriz University of Medical Sciences)
Mirtaheri (2015) [Iran][135]	RCT	2010 ACR/EULAR criteria, aged 20-50 years, DAS28<5.1, stable medication for 1 month, no anti-oxidants<br>Exclusions: other rheumatic diseases, cancer, diabetes, endocrine disorders, thyroid disorders, vitamin/mineral deficiency, BMI>40, hypertension, renal failure, hepatic diseases, gastrointestinal disorders, other autoimmune/inflammatory diseases, pregnancy/lactation, postmenopause, hormore replacement therapy, oral contraceptives, smoking,	1) Alpha-lipoic acid before breakfast and dinner<br>p) Maltodextrin	1) 33<br>p) 32	1) 36.1 (8.8)<br>p) 38.3 (8.6)	1) 33 (100)<br>p) 32 (100)	Not reported

ACR = American College of Rheumatology, N = number, RA = rheumatoid arthritis, SD = standard deviation, USA = United States of America

Table – Minerals and supplements (RA), description of included studies

Author (date) [country]	Study design	Inclusion criteria	Exposure detail	N	Age, mean (SD) years	N (%) female	Funders
Alavi (2011) [UK][136]	RCT	Aged ≥18 years, 1987 ACR RA criteria, stable medication for ≥2 months<br>Exclusions: quiescent disease, acute severe RA or severe concomitant disease requiring immunosuppressive or immunomodifying drugs, pregnant, breastfeeding, herbal remedies	1) Ambrotose complex – contains aloe vera, arabinogalactan, gum ghatti, gum tragacanth, glucosamine<br>p) identical placebo (rice flower)	1) 33<br>p) 36	Not reported §	Not reported §	**Industry** (Mannatech incorporated)
Bae (2009) [South Korea][124]	RCT §	1987 ACR RA criteria	1) Alpha-lipoic acid<br>p) Cornstarch	20	52.1 (10.3)	19 (95.0)	**University** (Sookmyung Women's University Research Grants)
Aryaeian (2008) [Iran][137]	RCT	1987 ACR RA criteria for >2 years, aged 19-69 years<br>Exclusions: abnormal renal/hepatic function, smoking, myocardial infarction, pregnancy, vitamins/mineral supplements, hyperlipidemia, taking thyroid hormones, estrogens, progesterone, diuretics or β-blockers	1) Linoleic acid capsules<br>2) Linoleic acid capsules + vitamin E<br>p) Sunflower and corn oil	1) 22<br>2) 22<br>p) 22	1) 46.2 (2.4)<br>2) 43.8 (12.8 [sic])<br>p) 48.0 (2.4)	1) 19 (86.3)<br>2) 17 (77.2)<br>p) 19 (86.3)	**University** (Tehran University of Medical Sciences)
Rastmanesh (2008) [Iran][138]	RCT	Women, aged 18-60 years, hypokalemic, 1987 ACR RA criteria, active disease: >4 swollen joints, >4 tender joints, ESR >30 or CRP >1, stable treatment for ≥2 months<br>Exclusions: inflammatory bowel disease, atrophic gastritis, and stoma, malignancy, and use of dietary supplements containing fish oil and/or antioxidants. Individuals with pre-existing renal disease, hyperkalemia, acidosis or insulin deficiency, using potassium-sparing diuretics, beta-adrenergic blockers, angiotensin-converting enzyme inhibitors, and digitalis	1) Enriched white grape juice containing potassium<br>p) Placebo grape juice	1) 18<br>p) 18	1) 49.5 (7.0)<br>p) 47.8 (5.1)	1) 18 (100)<br>p) 18 (100)	**Government** (Iranian National Nutrition and Food Technology Research Institute)
Nakamura (2007) [Japan][139]	RCT	1987 ACR RA criteria, stable medicine for 6 months, stable RA activity	1) Glucosamine tablets<br>P Placebo tablets	1) 25<br>p) 26	1) 61.4 (41-81)<br>p) 62.6 (43-81)§	1) 22 (88.0)<br>p) 22 (84.6)	Not reported

ACR = American College of Rheumatology, N = number, RA = rheumatoid arthritis, SD = standard deviation, USA = United States of America

Table – Minerals and supplements (RA), description of included studies

Author (date) [country]	Study design	Inclusion criteria	Exposure detail	N	Age, mean (SD) years	N (%) female	Funders
Marcora (2005) [UK][140]	RCT	1987 ACR RA criteria, stable medication for 3 months<br>Exclusions: any condition prevention safe participation of physical function tests or if an increase in nitrogen is contraindicated, cognitive impairment, presence of cachectic disease, taking drugs or nutritional supplements known to affect skeletal muscle mass (exception: steroids), participation in regular, intense exercise	1) Beta-hydroxy-beta-methylbutyrate, glutamine and arginine in a sachet – patients mixed powder with water<br>p) Placebo = isonitrogenous and isocaloric mixture of other, nonessential amino acids	1) 20<br>p) 20	1) 54 (10)<br>p) 57 (8)	1) 12 (60.0)<br>p) 13 (65.0)	Not reported
Mattingly (1982) [UK][141]	RCT	Classical or definite RA, symptom duration >1 year<br>Exclusions: receiving gold, D-penicillamine, chloroquine, levamisole and immunosuppressants	1) Zinc sulphate tablets (220mg)<br>p) Placebo tablets	1) 14<br>p) 13	1) 51<br>p) 57	1) 11 (78.5)<br>p) 10 (76.9)	Not reported
Simkin (1976) [USA][142]	RCT	Classical or definite RA, active disease	1) Zinc sulphate tablets (220mg)<br>p) Placebo tablets	24	54.3 (11.2)	Not reported	**Government** (National Institute of Arthritis and Musculoskeletal and Skin Diseases), **Charity** (Arthritis Foundatoin)
Bepler (1957) [USA][143]	RCT	1958 ACR RA criteria – definite cases only	1) Manganese glycerophosphate capsules<br>p) lactose placebo	1) 9<br>p) 9	1) 52.4 (range: 31-60)<br>p) 52.5 (range: 40-70)	Not reported	Not reported
Rasker (1982) [The Netherlands][144]	Single arm int.	Severe RA who failed antimalarials, gold, d-penicillamine, azathioprine	Zinc sulphate tablets (220mg)	22	57.6 (10.8)	20 (80)	Not reported

ACR = American College of Rheumatology, N = number, RA = rheumatoid arthritis, SD = standard deviation, USA = United States of America

Supplementary table 88 – Alpha-lipoic acid and RA progression, results

Table – Alpha-lipoic acid (RA), results and quality assessment

Outcome	Study (date) [study type]	Standardised result, SMD (95% CI) unless otherwise stated	Natural result	AMSTAR2 quality	Rand. Seq.	Alloc. Conc.	Blind. Part.	Blind. Asses.
Pain	Bae (2009) [RCT][124]	Alpha-lipoic acid vs placebo at week 4<br>SMD -0.12 (-0.74, 0.50)	Pain VAS, BL / 4 weeks, mean (SD*)<br>Alpha-lipoic acid: 35.12 (31.91) / 30.00 (23.93)<br>Placebo: 32.25 (27.92) / 33.33 (31.91)		H/UC	H/UC	L	H/UC
Function	Bae (2009) [RCT][124]	Alpha-lipoic acid vs placebo at week 4<br>SMD -0.30 (-0.93, 0.32)	KHAQ, BL / 4 weeks, mean (SD*)<br>Alpha-lipoic acid: 0.49 (0.32) / 0.43 (0.39)<br>Placebo: 0.47 (0.40) / 0.59 (0.64)		H/UC	H/UC	L	H/UC
CRP	Mirtaheri (2015) [RCT][135]	Alpha-lipoic acid vs placebo at week 8<br>SMD -0.21 (-0.70, 0.28)	CRP, BL / 8 weeks, mean (SD*)<br>Alpha-lipoic acid: 4.7 (7.0) / 2.7 (3.3)<br>Placebo: 4.6 (6.7) / 3.5 (4.2)		L	H/UC	L	H/UC
	Bae (2009) [RCT][124]	Alpha-lipoic acid vs placebo at week 4<br>SMD -0.32 (-0.94, 0.30)	CRP, BL / 4 weeks, mean (SD*)<br>Alpha-lipoic acid: 1.75 (3.30) / 1.33 (2.40)<br>Placebo: 1.71 (2.97) / 2.33 (3.71)		H/UC	H/UC	L	H/UC
	Bespoke MA<br>Mirataheri[135]<br>Bae[124]	Alpha-lipoic acid vs placebo<br>SMD -0.25 (-0.64, 0.13) I^2 0.0%						

* mean (SD) calculated from median (IQR) using published formula[61]

Alloc. Conc. = allocation concealment, AMSTAR2 = Assessing the Methodological Quality of Systematic Reviews, BL = baseline, Blind. Asses. = Blinded assessors, Blind. Part. = blinded participants, CI = confidence interval, DAS28 = Disease Activity Score 28, ESR = erythrocyte sedimentation rate, H/UC = high / unclear risk of bias, HAQ = Health Assessment Questionnaire, IQR = interquartile range, int. = intervention, KHAQ = Korean Health Assessment Questionnaire, L = low risk of bias, RA = rheumatoid arthritis, Rand. Seq. = random sequence generation, RCT = randomised controlled trial, SD =standard deviation, SMD = Standardised mean difference, VAS = visual analogue scale

Supplementary table 89 – Ambrotose and RA progression, results

Table – Ambrotose (RA), results and quality assessment

Outcome	Study (date) [study type]	Standardised result, SMD (95% CI) unless otherwise stated	Natural result	AMSTAR2 quality	Rand. Seq.	Alloc. Conc.	Blind. Part.	Blind. Asses.
Pain	Alavi (2011) [RCT][136]	Ambrotose vs placebo, change BL-6 months<br>SMD -0.44 (-0.92, 0.04)	SF36 – pain, mean change BL-6 months (SD)<br>Ambrotose: -4.83 (19.38)<br>Placebo: 4.28 (21.61)		L	L	L	L
Function	Alavi (2011) [RCT][136]	Ambrotose vs placebo, change BL-6 months<br>SMD -0.11 (-0.58, 0.37)	SF36 – function, mean change BL-6 months (SD)<br>Ambrotose: 2.17 (20.16)<br>Placebo: 4.22 (18.97)		L	L	L	L
Disease activity	Alavi (2011) [RCT][136]		DAS28, mean difference at 6 months adjusted for baseline (SE)<br>0.63 (0.23) p=0.009		L	L	L	L
Patient global	Alavi (2011) [RCT][136]		Patient global VAS, mean difference at 6 months adjusted for baseline (SE)<br>10.5 (4.4) p=0.02		L	L	L	L
Fatigue	Alavi (2011) [RCT][136]	Ambrotose vs placebo, change BL-6 months<br>SMD 0.02 (-0.45, 0.49)	SF36 – vitality, mean change BL-6 months (SD)<br>Ambrotose: -15.75 (14.61)<br>Placebo: -16.13 (20.14)		L	L	L	L
QoL	Alavi (2011) [RCT][136]	Ambrotose vs placebo, change BL-6 months<br>SMD -0.03 (-0.50, 0.45)	WHO QoL, mean change BL-6 months (SD)<br>Ambrotose: 1.41 (5.70)<br>Placebo: 1.53 (3.86)		L	L	L	L
Anxiety	Alavi (2011) [RCT][136]	Ambrotose vs placebo, change BL-6 months<br>SMD 0.15 (-0.33, 0.62)	HADS anxiety, mean change BL-6 months (SD)<br>Ambrotose: 0.33 (2.32)<br>Placebo: -0.06 (2.95)		L	L	L	L
Depression	Alavi (2011) [RCT][136]	Ambrotose vs placebo, change BL-6 months<br>SMD 0.40 (-0.07, 0.88)	HADS Depression, mean change BL-6 months (SD)<br>Ambrotose: 0.10 (1.58)<br>Placebo: -0.64 (2.04)		L	L	L	L

Alloc. Conc. = allocation concealment, AMSTAR2 = Assessing the Methodological Quality of Systematic Reviews, BL = baseline, Blind. Asses. = Blinded assessors, Blind. Part. = blinded participants, CI = confidence interval, H/UC = high / unclear risk of bias, HADS = Hospital Anxiety and Depression Scale, L = low risk of bias, QoL = Quality of Life, RA = rheumatoid arthritis, Rand. Seq. = random sequence generation, RCT = randomised controlled trial, SD = standard deviation, SE = standard error, SMD = Standardised mean difference, WHO = World Health Organisation

Supplementary table 90 – Co-enzyme Q10 and RA progression, results

Table – Co-enzyme Q10 (RA), results and quality assessment

Outcome	Study (date) [study type]	Standardised result, SMD (95% CI) unless otherwise stated	Natural result	AMSTAR2 quality	Rand. Seq.	Alloc. Conc.	Blind. Part.	Blind. Asses.
CRP	Abdollahzad (2015) [RCT][134]	Co-enzyme Q10 vs placebo at 2 months<br>SMD -0.43 (-1.02, 0.16)	CRP, BL / 2 months, mean (SD)<br>Co-enzyme Q10: 19.9 (18.0) / 14.7 (11.7)<br>Placebo: 24.3 (19.9) / 21.3 (18.2)		L	L	L	L

Alloc. Conc. = allocation concealment, AMSTAR2 = Assessing the Methodological Quality of Systematic Reviews, BL = baseline, Blind. Asses. = Blinded assessors, Blind. Part. = blinded participants, CI = confidence interval, CRP = C-reactive protein, H/UC = high / unclear risk of bias, L = low risk of bias, RA = rheumatoid arthritis, Rand. Seq. = random sequence generation, RCT = randomised controlled trial, SD = standard deviation, SMD = Standardised mean difference,

Supplementary table 91 – Creatine and RA progression, results

Table – Creatine (RA), results and quality assessment

Outcome	Study (date) [study type]	Standardised result, SMD (95% CI) unless otherwise stated	Natural result	AMSTAR2 quality	Rand. Seq.	Alloc. Conc.	Blind. Part.	Blind. Asses.
Function	Wilkinson (2016) [RCT][133]	Creatine vs placebo, change over 12 weeks<br>SMD 0.00 (-0.67, 0.67)	mHAQ, change from BL-12 weeks, mean (SD)<br>Creatine: -0.1 (0.1)<br>Placebo: -0.1 (0.1); p=0.836		L	L	L	L
Disease activity	Wilkinson (2016) [RCT][133]	Creatine vs placebo, change over 12 weeks<br>SMD 0.00 (-0.67, 0.67)	DAS28, change from BL-12 weeks, mean (SD)<br>Creatine: -0.1 (0.2)<br>Placebo: -0.1 (0.2); p=0.990		L	L	L	L

Alloc. Conc. = allocation concealment, AMSTAR2 = Assessing the Methodological Quality of Systematic Reviews, Blind. Asses. = Blinded assessors, Blind. Part. = blinded participants, CI = confidence interval, DAS28 = Disease Activity Score 28, H/UC = high / unclear risk of bias, L = low risk of bias, mHAQ = modified Health Assessment Questionnaire, RA = rheumatoid arthritis, Rand. Seq. = random sequence generation, RCT = randomised controlled trial, SD = standard deviation, SMD = Standardised mean difference,

Supplementary table 92 – Glucosamine and RA progression, results

Table – Glucosamine (RA), results and quality assessment

Outcome	Study (date) [study type]	Standardised result, SMD (95% CI) unless otherwise stated	Natural result	AMSTAR2 quality	Rand. Seq.	Alloc. Conc.	Blind. Part.	Blind. Asses.
CRP	Nakamura (2007) [RCT][139]	<u>CRP vs placebo at 12 weeks</u><br>SMD 0.03 (-0.52, 0.57)	<u>CRP, BL / 12 weeks, mean (SD*)</u><br>Glucosamine: 0.81 (4.5) / 1.07 (7.9)<br>Placebo: 1.13 (6.9) / 0.91 (4.4)		H/UC	H/UC	H/UC	H/UC
ESR	Nakamura (2007) [RCT][139]	<u>CRP vs placebo at 12 weeks</u><br>SMD 0.00 (-0.55, 0.55)	<u>ESR, BL / 12 weeks, mean (SD*)</u><br>Glucosamine: 29.9 (71.5) / 30.4 (83.0)<br>Placebo: 31.4 (109.6) / 30.5 (96.9)		H/UC	H/UC	H/UC	H/UC

* Calculated from standard error in paper

Alloc. Conc. = allocation concealment, AMSTAR2 = Assessing the Methodological Quality of Systematic Reviews, Blind. Asses. = Blinded assessors, Blind. Part. = blinded participants, CI = confidence interval, H/UC = high / unclear risk of bias, L = low risk of bias, RA = rheumatoid arthritis, Rand. Seq. = random sequence generation, RCT = randomised controlled trial, SD = standard deviation, SMD = Standardised mean difference,

Supplementary table 93 – Linoleic acid and RA progression, results

Table – Linoleic acid (RA), results and quality assessment

Outcome	Study (date) [study type]	Standardised result, SMD (95% CI) unless otherwise stated	Natural result	AMSTAR 2 quality	Rand. Seq.	Alloc. Conc.	Blind. Part.	Blind. Asses.
CRP	Aryaeian (2008) [RCT][137]	Linoleic acid vs placebo<br>SMD -0.00 (-0.60, 0.59)<br>Linoleic acid + vitamin E vs placebo<br>SMD -0.49 (-1.08, 0.12)	CRP, BL / 12 weeks, mean (SD*)<br>Linoleic acid: 7.18 (10.1) / 5.46 (5.5)<br>Linoleic acid + vitamin E: 5.23 (6.4) / 3.17 (3.9)<br>Placebo: 6.44 (7.9) / 5.48 (5.6)		H/UC	H/UC	H/UC	H/UC
ESR	Aryaeian (2008) [RCT][137]	Linoleic acid vs placebo<br>SMD -0.52 (-1.12, 0.08)<br>Linoleic acid + vitamin E vs placebo<br>SMD -0.58 (-1.19, 0.02)	CRP, BL / 12 weeks, mean (SD*)<br>Linoleic acid: 26.81 (11.2) / 19.14 (10.1)<br>Linoleic acid + vitamin E: 28.45 (17.3) / 17.77 (12.2)<br>Placebo: 28.36 (21.5) / 27.04 (18.9)		H/UC	H/UC	H/UC	H/UC

*SD calculated from standard error reporting in paper

Alloc. Conc. = allocation concealment, AMSTAR2 = Assessing the Methodological Quality of Systematic Reviews, Blind. Asses. = Blinded assessors, Blind. Part. = blinded participants, CI = confidence interval, CRP = C-reactive protein, H/UC = high / unclear risk of bias, L = low risk of bias, RA = rheumatoid arthritis, Rand. Seq. = random sequence generation, RCT = randomised controlled trial, SD = standard deviation, SMD = Standardised mean difference,

Supplementary table 94 – Manganese and RA progression, results

Table – Manganese (RA), results and quality assessment

Outcome	Study (date) [study type]	Standardised result, SMD (95% CI) unless otherwise stated	Natural result	AMSTAR2 quality	Rand. Seq.	Alloc. Conc.	Blind. Part.	Blind. Asses.
Disease severity	Bepler (1957) [RCT][143]		Number improved / got worse after 2 months<br>1) 5 / 4<br>p) 5 / 4		H/UC	H/UC	H/UC	H/UC

Alloc. Conc. = allocation concealment, AMSTAR2 = Assessing the Methodological Quality of Systematic Reviews, Blind. Asses. = Blinded assessors, Blind. Part. = blinded participants, CI = confidence interval, H/UC = high / unclear risk of bias, L = low risk of bias, RA = rheumatoid arthritis, Rand. Seq. = random sequence generation, RCT = randomised controlled trial, SMD = Standardised mean difference,

Supplementary table 95 – Potassium and RA progression, results

Table – Potassium (RA), results and quality assessment

Outcome	Study (date) [study type]	Standardised result, SMD (95% CI) unless otherwise stated	Natural result	AMSTAR2 quality	Rand. Seq.	Alloc. Conc.	Blind. Part.	Blind. Asses.
Pain	Rastmanesh (2008) [RCT][138]	Potassium vs placebo, change BL-28 days<br>SMD -2.63 (-3.54, -1.73)	Pain VAS, change BL-28 days, mean (SD)<br>Potassium: -27.5 (8.7)<br>Placebo: -3.4 (9.6); p<0.01		L	L	L	L
Disease activity	Rastmanesh (2008) [RCT][138]	Potassium vs placebo, change BL-28 days<br>SMD -2.98 (-3.94, -2.02)	DAS28, change BL-28 days, mean (SD)<br>Potassium: -0.69 (0.23)<br>Placebo: -0.10 (0.16); p<0.01		L	L	L	L
Tender joints	Rastmanesh (2008) [RCT][138]	Potassium vs placebo, change BL-28 days<br>SMD -2.20 (-3.03, -1.36)	Tender joint count, change BL-28 days, mean (SD)<br>Potassium: -3.1 (1.65)<br>Placebo: -0.31 (0.70); p<0.01		L	L	L	L
Swollen joints	Rastmanesh (2008) [RCT][138]	Potassium vs placebo, change BL-28 days<br>SMD -2.76 (-3.69, -1.84)	Swollen joint count, change BL-28 days, mean (SD)<br>Potassium: -2.93 (1.12)<br>Placebo: -0.43 (0.62); p<0.03		L	L	L	L
Patient global	Rastmanesh (2008) [RCT][138]	Potassium vs placebo, change BL-28 days<br>SMD -0.86 (-1.55, -0.18)	Patient global VAS, change BL-28 days, mean (SD)<br>Potassium: -6.2 (7.6)<br>Placebo: -0.93 (4.1); p<0.02		L	L	L	L
CRP	Rastmanesh (2008) [RCT][138]	Potassium vs placebo, change BL-28 days<br>SMD -0.80 (-1.48, -0.12)	CRP, change BL-28 days, mean (SD)<br>Potassium: -3.25 (4.70)<br>Placebo: -0.09 (3.00); p<0.02		L	L	L	L
ESR	Rastmanesh (2008) [RCT][138]	Potassium vs placebo, change BL-28 days<br>SMD -1.93 (-2.73, -1.13)	ESR, change BL-28 days, mean (SD)<br>Potassium: -14.30 (7.15)<br>Placebo: -2.06 (5.40); p<0.001		L	L	L	L

Alloc. Conc. = allocation concealment, AMSTAR2 = Assessing the Methodological Quality of Systematic Reviews, BL = baseline, Blind. Asses. = Blinded assessors, Blind. Part. = blinded participants, CI = confidence interval, DAS28 = Disease Activity Score 28, H/UC = high / unclear risk of bias, L = low risk of bias, RA = rheumatoid arthritis, Rand. Seq. = random sequence generation, RCT = randomised controlled trial, SD = standard deviation, SMD = Standardised mean difference, VAS = visual analogue scale

Supplementary table 96 – Probiotics and RA progression, results

Table – Probiotics (RA), results and quality assessment

Outcome	Study (date) [study type]	Standardised result, SMD (95% CI) unless otherwise stated	Natural result	AMSTAR2 quality	Rand. Seq.	Alloc. Conc.	Blind. Part.	Blind. Asses.
Pain	Zamani (2017) [RCT][132]	Synbiotic vs placebo at 8 weeks<br>SMD -0.41 (-0.95, 0.13)	Pain VAS, mean (SD) at 8 weeks<br>Synbiotics: 27.0 (15.6)<br>Control: 35.9 (26.8)		L	H/UC	L	L
Function	Aqaeinezhad Rudbane (2018) [MA][130]	Probiotics vs placebo<br>SMD -0.30 (-0.89, 0.29)		Low				
	Mohammed (2017) [MA][131]	Probiotics vs placebo<br>MD -0.11 (-0.23, 0.01)		Moderate				
Disease activity	Aqaeinezhad Rudbane (2018) [MA][130]	Probiotics vs placebo<br>SMD -0.58 (-0.97, -0.19)		Low				
	Mohammed (2017) [MA][131]	Probiotics vs placebo<br>MD 0.02 (-0.58, 0.63)		Moderate				
	Zamani (2017) [RCT][132]	Synbiotic vs placebo at 8 weeks<br>SMD -0.65 (-1.20, -0.10)	DAS28, mean (SD) at 8 weeks<br>Synbiotics: 2.6 (0.7)<br>Control: 3.2 (1.1)		L	H/UC	L	L
Tender joints	Aqaeinezhad Rudbane (2018) [MA][130]	Probiotics vs placebo<br>SMD -0.21 (-0.53, 0.11)		Low				
Swollen joints	Aqaeinezhad Rudbane (2018) [MA][130]	Probiotics vs placebo<br>SMD -0.30 (-0.62, 0.02)		Low				
	Mohammed (2017) [MA][131]	Probiotics vs placebo<br>MD 0.17 (-0.39, 0.73)		Moderate				
CRP	Aqaeinezhad Rudbane (2018) [MA][130]	Probiotics vs placebo<br>SMD -0.32 (-0.65, 0.00)		Low				
	Mohammed (2017) [MA][131]	Probiotics vs placebo<br>MD -1.40 (-4.06, 1.26)		Moderate				
	Zamani (2017) [RCT][132]	Synbiotic vs placebo at 8 weeks<br>SMD -0.74 (-1.30, -0.19)	CRP, mean (SD) at 8 weeks<br>Synbiotics: 4609.2 (2711.7)<br>Control: 8474.1 (6829.7)		L	H/UC	L	L
ESR	Aqaeinezhad Rudbane (2018) [MA][130]	Probiotics vs placebo<br>SMD -0.17 (-0.76, 0.42)		Low				

Alloc. Conc. = allocation concealment, AMSTAR2 = Assessing the Methodological Quality of Systematic Reviews, Blind. Asses. = Blinded assessors, Blind. Part. = blinded participants, CI = confidence interval, CRP = C-reactive protein, DAS28 = Disease Activity Score 28, ESR = erythrocyte sedimentation rate, H/UC = high / unclear risk of bias, L = low risk of bias, MA = meta-analysis, RA = rheumatoid arthritis, Rand. Seq. = random sequence generation, SD = standard deviation, SMD = Standardised mean difference, VAS = visual analogue scale

Supplementary table 97 – Zinc and RA progression, results

Table – Zinc (RA), results and quality assessment

Outcome	Study (date) [study type]	Standardised result, SMD (95% CI) unless otherwise stated	Natural result	AMSTAR2 quality	Rand. Seq.	Alloc. Conc.	Blind. Part.	Blind. Asses.
Pain	Mattingly (1982) [RCT][141]		Pain VAS (0-20), BL / 6 months, mean<br>Zinc: 7.83 / 5.00<br>Placebo: 11.56 / 8.56		H/UC	H/UC	L	H/UC
Tender joints	Mattingly (1982) [RCT][141]		Ritchie Index, BL / 6 months, mean<br>Zinc: 21.2 / 19.6<br>Placebo: 27.8 / 26.3		H/UC	H/UC	L	H/UC
	Simkin (1976) [RCT][142]		Tenderness, BL / 12 weeks, mean (SE)<br>Zinc: 28 (5) / 24 (5)<br>Placebo: 28 (5) / 29 (9)		H/UC	L	L	H/UC
Swollen joints	Simkin (1976) [RCT][142]		Swelling, BL / 12 weeks, mean (SE)<br>Zinc: 27 (3) / 20 (3)<br>Placebo: 14 (2) / 13 (3) p<0.02		H/UC	L	L	H/UC
Joint score	Rasker (1982) [Single arm int.] [144]		Joint score*, BL / 2 months, mean (SD)<br>17 (7) / 19 (8)					
Morning stiffness	Mattingly (1982) [RCT][141]		Morning stiffness, BL / 6 months, mean<br>Zinc: 1.92 / 1.58<br>Placebo: 2.56 / 3.22		H/UC	H/UC	L	H/UC
	Simkin (1976) [RCT][142]		Stiffness, BL / 12 weeks, mean (SE)<br>Zinc: 4.0 (0.4) / 3.0 (0.8)<br>Placebo: 3.5 (0.4) / 3.6 (0.5)		H/UC	L	L	H/UC
Patient global	Mattingly (1982) [RCT][141]		Patient global VAS, BL / 6 months, mean<br>Zinc: 2.92 / 3.42<br>Placebo: 2.67 / 3.11		H/UC	H/UC	L	H/UC
	Simkin (1976) [RCT][142]		Patient global VAS, BL / 12 weeks, mean (SE)<br>Zinc: 3.3 (0.2) / 3.1 (0.3)<br>Placebo: 3.1 (0.1) / 3.2 (0.2)		H/UC	L	L	H/UC
ESR	Mattingly (1982) [RCT][141]		ESR, BL / 6 months, mean<br>Zinc: 49.4 / 44.7<br>Placebo: 61.2 / 64.3		H/UC	H/UC	L	H/UC
	Rasker (1982) [Single arm int.] [144]		ESR, BL / 2 months, mean (SD)<br>20.3 (28.9) / 53.8 (27.8)					
Grip strength	Mattingly (1982) [RCT][141]		Grip strength, BL / 6 months, mean<br>Zinc: 367 / 411<br>Placebo: 300 / 337		H/UC	H/UC	L	H/UC
	Simkin (1976) [RCT][142]		Grip strength, BL / 12 weeks, mean (SE)<br>Zinc: 100 (16) / 98 (14)<br>Placebo: 85 (12) / 84 (11)		H/UC	L	L	H/UC

* Joint score from Rasker = Number of affected joints, counting MCP, PIP and MTP joints of each limb as one.

Alloc. Conc. = allocation concealment, AMSTAR2 = Assessing the Methodological Quality of Systematic Reviews, Blind. Asses. = Blinded assessors, Blind. Part. = blinded participants, CI = confidence interval, ESR = erythrocyte sedimentation rate, H/UC = high / unclear risk of bias, int. = intervention, L = low risk of bias, MCP = metacarpophalangeal, MTP = metatarsophalangeal, PIP = proximal interphalangeal, RA = rheumatoid arthritis, Rand. Seq. = random sequence generation, RCT = Randomised Controlled Trial, SD = standard deviation, SE = standard error, SMD = Standardised mean difference, VAS = visual analogue scale

Supplementary table 98 – Combined supplements and RA progression, results

Table – Combined supplements (RA), results and quality assessment

Outcome	Study (date) [study type]	Standardised result, SMD (95% CI) unless otherwise stated	Natural result	AMSTAR2 quality	Rand. Seq.	Alloc. Conc.	Blind. Part.	Blind. Asses.
Disease Activity	Marcora (2005) [RCT][140]	Supplements vs placebo at 12 weeks<br>SMD -0.69 (-1.33, -0.05)	RADAI, BL / 12 weeks, mean (SD)<br>Supplements: 2.8 (1.1) / 3.0 (1.2)<br>Placebo: 3.8 (1.4) / 3.9 (1.4); p=0.00 [sic]		L	L	L	L
Function	Marcora (2005) [RCT][140]	Supplements vs placebo at 12 weeks<br>SMD -0.50 (-1.13, 0.13)	mHAQ, BL / 12 weeks, mean (SD)<br>Supplements: 1.5 (0.4) / 1.4 (0.4)<br>Placebo: 1.5 (0.3) / 1.6 (0.4); p=0.03		L	L	L	L
Fatigue	Marcora (2005) [RCT][140]	Supplements vs placebo at 12 weeks<br>SMD -0.86 (-1.51, -0.21)	Fatigue (0-10), BL / 12 weeks, mean (SD)<br>Supplements: 3.9 (3.0) / 3.1 (2.7)<br>Placebo: 5.2 (1.7) / 5.5 (2.9); p=0.06		L	L	L	L
Psychological status	Marcora (2005) [RCT][140]	Supplements vs placebo at 12 weeks<br>SMD -0.39 (-1.02, 0.23)	Psychological status (1-4), BL / 12 weeks, mean (SD)<br>Supplements: 1.6 (0.5) / 1.5 (0.4)<br>Placebo: 1.6 (0.4) / 1.7 (0.6); p=0.02		L	L	L	L
ESR	Marcora (2005) [RCT][140]	Supplements vs placebo at 12 weeks<br>SMD -0.13 (-0.75, 0.49)	ESR, BL / 12 weeks, mean (SD)<br>Supplements: 27.4 (22.6) / 23.3 (19.4)<br>Placebo: 22.7 (14.6) / 25.4 (12.1); p=0.07		L	L	L	L
Grip strength	Marcora (2005) [RCT][140]	Supplements vs placebo at 12 weeks<br>SMD 0.43 (-0.20, 1.06)	Grip strength, BL / 12 weeks, mean (SD)<br>Supplements: 169 (126) / 181 (116)<br>Placebo: 142 (103) / 137 (87); p=0.01		L	L	L	L

Alloc. Conc. = allocation concealment, AMSTAR2 = Assessing the Methodological Quality of Systematic Reviews, Blind. Asses. = Blinded assessors, Blind. Part. = blinded participants, CI = confidence interval, ESR = erythrocyte sedimentation rate, H/UC = high / unclear risk of bias, L = low risk of bias, mHAQ = modified Health Assessment Questionnaire, RA = rheumatoid arthritis, RADAI = Rheumatoid Arthritis Disease Activity Index, Rand. Seq. = random sequence generation, RCT = randomised controlled trial, SD =standard deviation, SMD = Standardised mean difference,

Supplementary table 99 – Description of reviews of vitamins in RA

Table – Vitamins (RA), description of reviews

Authors (date)	Review type	Study type included	Exposure detail	Number of studies included	Funders
Franco (2017)[145]	MA	RCT	Vitamin D	5	**Charity** (São Paulo Research Foundation, Federico Foundation), **Government** (National Council for Scientific and Technological Development)

MA = meta-analysis, RCT = randomised controlled trial

Supplementary table 100 – Description of studies of vitamins in RA

Table – Vitamins (RA), description of included studies

Author (date) [country]	Study design	Inclusion criteria	Exposure detail	N	Age, mean (SD) years	N (%) female	Funders
Batooei (2018) [Iran][146]	RCT	Aged 18-65 years, 2010 ACR/EULAR criteria, active RA, stable renal function, absence of liver disease, can take oral intervention<br>Exclusions: other inflammatory disease, receiving anti-inflammatory or antioxidant medications in past month, pregnancy/breast feeding	1) 600mg N-acetylcysteine (antioxidant) as effervescent tablets twice a day<br>p) Identical effervescent placebo	1) 27<br>p) 24	1) 53.2 (12.5)<br>p) 51.6 (11.3)	1) 22 (81.5)<br>p) 23 (95.8)	**University (**Hamadan University of Medical Sciences)
Huang (2010) [Taiwan][147]	RCT	1987 ACR RA criteria, adults<br>Exclusions: Pregnant, anaemia, thrombocytopenia, abnormal liver function, renal insufficiency, diabetes, cancer	1) 100mg/day vitamin B6 + folic acid<br>p) Folic acid only	1) 20<br>p) 15	1) 53.9 (2.0)<br>p) 53.0 (2.0)	1) 17 (85.0)<br>p) 13 (86.7)	**Government** (National Science Council, Taiwan)
Nourmohammadi (2010) [Iran][148]	RCT	1987 ACR RA criteria, "inactive RA"<br>Exclusions: chronic diseases: renal, diabetes, hepatic, hypertension, dyslipidaemia, inflammatory diseases, infection, malnutrition, obesity, smoking, alcohol	1) 300mg vitamin C, 5mg zinc, 25000 IU vitamin A every other day for 12 weeks<br>p) Conventional treatment only (no placebo)	1) 24<br>p) 25	1) 48.8 (12.6)<br>p) 48.8 (12.7)	1) 20 (83.3)<br>p) 21 (84.0)	**University** (Iran University of Medical Sciences)
Aryaeian (2008) [Iran][137]	RCT	1987 ACR RA criteria for >2 years, aged 19-69 years<br>Exclusions: abnormal renal/hepatic function, smoking, myocardial infarction, pregnancy, vitamins/mineral supplements, hyperlipidemia, taking thyroid hormones, estrogens, progesterone, diuretics or β-blockers	1) Vitamin E<br>2) vitamin E + Linoleic acid capsules<br>p) Sunflower and corn oil	1) 21<br>2) 22<br>p) 22	1) 46.2 (2.4)<br>2) 43.8 (12.8 [sic])<br>p) 48.0 (2.4)	1) 19 (86.3)<br>2) 17 (77.2)<br>p) 19 (86.3)	**University** (Tehran University of Medical Sciences)
Chiang (2005) [USA][149]	RCT	Aged >18 years, 1987 ACR RA criteria, vitamin B6 deficient<br>Exclusions: pregnancy, oral contraceptive use, anaemia , thrombocytopenia, renal insufficiency, diabetes, cancer	1) 50mg vitamin B6<br>p) Identical placebo tablet	1) 14<br>p) 14	1) 53.9 (12.6)<br>p) 57.5 (11.0)	1) 12 (85.7)<br>p) 9 (64.3)	**Government** (National Science Council, Taiwan, US Department of Agriculture), **Charity** (Arthritis Foundation)

ACR = American College of Rheumatology, DMARD = disease modifying anti-rheumatic drug, EULAR = European League Against Rheumatism, mg = milligrams, N = number, NRT = non-randomised trial, NSAID = non-steroidal anti-inflammatory drug, RA = rheumatoid arthritis, RF = rheumatoid factor, SD = standard deviation

Table – Vitamins (RA), description of included studies

Author (date) [country]	Study design	Inclusion criteria	Exposure detail	N	Age, mean (SD) years	N (%) female	Funders
Edmonds (1997) [UK][150]	RCT	1987 ACR RA criteria, aged 18-80 years, Ritchie index ≥6 or morning stiffness ≥1 hour, receiving NSAIDs or DMARDs<br>Exclusions: already taking vitamin E, vitamin E hypersensitivity, pregnancy, malabsorption, malignancy	1) Vitamin E<br>p) Identical placebo	1) 20<br>p) 19	1) 55.4 (15.1)<br>p) 52.0 (10.3)	1) 16 (80.0)<br>p) 15 (78.9)	**Not reported**
Helmy (2001) [Egypt][151]	NRT	1987 ACR RA criteria<br>Exclusions: endocrine, hepatic or renal disorders, malignancy or overt infections	1) Selenium, medicinal yeast, vitamin A, ascorbic acid, vitamin E<br>2) Same as 1), plus high dose of vitamin E<br>p) standard treatment only	1) 10<br>2) 10<br>p) 10	1) 37.1 (8.8)<br>2) 39.5 (1.1) [sic]<br>p) 43.9 (12.9)	1) 8 (80.0)<br>2) 8 (80.0)<br>p) 7 (70.0)	**Not reported**
Jalili (2014) [Iran][152]	Single arm int.	1987 ACR RA criteria, aged 40-60 years, stable treatment ≥2 months<br>Exclusions: diabetes, hypertension, thyroid disorders, liver and kidney failure, Cushing syndrome, severe infection, gastric illness, smoking	1) "Selenplus" capsule - 50 μg selenium, 8 mg zinc, 400 μg vitamin A, 125 mg vitamin C, and 40 mg vitamin E.	39	52.6 (5.3)	39 (100)	No source of funding, no conflicts of interest
van Vugt (2008) [The Netherlands][153]	Single arm int.	RF+, 1987 ACR RA criteria, non-smokers, not obese, NSAID/DMARD therapy for ≥3 months	Antioxidant enriched margerine, The spread contained a mix of a-tocopherol (400 mg), lycopene (10 mg), palm oil carotenoids (5 mg; mainly α-carotene) and lutein (10 mg). Further, patients received vitamin C (200 mg daily) as a supplement.	8	Not reported	8 (100)	**Not reported**

ACR = American College of Rheumatology, DMARD = disease modifying anti-rheumatic drug, EULAR = European League Against Rheumatism, mg = milligrams, N = number, NRT = non-randomised trial, NSAID = non-steroidal anti-inflammatory drug, RA = rheumatoid arthritis, RF = rheumatoid factor, SD = standard deviation

Supplementary table 101 – Antioxidants and RA progression, results

Table – Antioxidants (RA), results and quality assessment

Outcome	Study (date) [study type]	Standardised result, SMD (95% CI) unless otherwise stated	Natural result	AMSTAR2 quality	Rand. Seq.	Alloc. Conc.	Blind. Part.	Blind. Asses.
Pain	Batooei (2018) [RCT][146]	Antioxidants vs placebo at 12 weeks<br>SMD -1.17 (-1.77, -0.57)	Pain VAS, BL / 12 weeks, mean (SD)<br>Antioxidant: 77.6 (10.9) / 50 (7.8)<br>Placebo: 77.9 (17.7) / 66.9 (19.4); p=0.001		H/UC	H/UC	L	L
Function	Batooei (2018) [RCT][146]	Antioxidants vs placebo at 12 weeks<br>SMD -0.86 (-1.44, -0.28)	HAQ, BL / 12 weeks, mean (SD)<br>Antioxidant: 22.6 (13.1) / 13.9 (9.6)<br>Placebo: 28.7 (11.7) / 24.1 (14); p<0.01		H/UC	H/UC	L	L
Disease activity	Batooei (2018) [RCT][146]	Antioxidants vs placebo at 12 weeks<br>SMD -0.26 (-0.81, 0.29)	DAS28, BL / 12 weeks, mean (SD)<br>Antioxidant: 5.1 (1.2) / 4.35 (1.2)<br>Placebo: 5.3 (1.1) / 4.7 (1.5); p=0.4		H/UC	H/UC	L	L
	Nourmohammadi (2018) [RCT][148]	Antioxidants vs control at 12 weeks<br>SMD -0.85 (-1.43, -0.26)	RADAI, BL / 12 weeks, mean (SD)<br>Antioxidants: 5.06 (1.32) / 2.59 (0.95)<br>Control: 4.96 (1.23) / 3.52 (1.22); p=0.005		H/UC	H/UC	H/UC	H/UC
	Jalili (2014) [Single arm int.][152]		DAS28, BL / 12 weeks, mean (SD)<br>Antioxidants: 2.71 (1.19) / 2.65 (1.17); p=0.019					
	van Vugt (2008) [Single arm int.][153]		DAS28, BL / 10 weeks, mean<br>Antioxidants: 5.84 / 4.82					
Tender joints	Batooei (2018) [RCT][146]	Antioxidants vs placebo at 12 weeks<br>SMD -0.11 (-0.66, 0.44)	Tender joint count, BL / 12 weeks, mean (SD)<br>Antioxidant: 10.6 (7.7) / 6.9 (5.5)<br>Placebo: 10.9 (7) / 7.6 (7.5); p=0.7		H/UC	H/UC	L	L
	Helmy (2001) [NRT][151]	Antioxidants vs Control at 2 months<br>SMD -1.32 (-2.30, -0.35)<br>Antioxidants + vit E vs Control at 2 months<br>SMD -1.19 (-2.15, -0.30)	Ritchie Index, BL / 2 months, mean (SD)<br>Antioxidants: 37.0 (11.6) / 7.0 (6.3)<br>Antioxidants + vit E: 32.5 (1.4) [sic] / 8.5 (5.8)<br>Control: 26.5 (17.6) / 20.0 (12.4)					
	Jalili (2014) [Single arm int.][152]		Tender joint count, BL / 12 weeks, median (range)<br>Antioxidants: 1 (0-17) / 1 (0-14); p=0.839					
Swollen joints	Batooei (2018) [RCT][146]	Antioxidants vs placebo at 12 weeks<br>SMD -0.15 (-0.71, 0.40)	Swollen joint count, BL / 12 weeks, mean (SD)<br>Antioxidant: 8.4 (6.2) / 6.3 (4.9)<br>Placebo: 9.1 (5.7) / 7.1 (5.5); p=0.4		H/UC	H/UC	L	L
	Jalili (2014) [Single arm int.][152]		Swollen joint count, BL / 12 weeks, median (range)<br>Antioxidants: 0 (0-15) / 0 (0-14); p=0.736					
Patient global	Batooei (2018) [RCT][146]	Antioxidants vs placebo at 12 weeks<br>SMD -0.70 (-1.26, -0.13)	Patient global VAS, BL / 12 weeks, mean (SD)<br>Antioxidant: 31.7 (11.3) / 23.6 (15)<br>Placebo: 37.7 (14.7) / 35.6 (19.5); p<0.01		H/UC	H/UC	L	L

Alloc. Conc. = allocation concealment, AMSTAR2 = Assessing the Methodological Quality of Systematic Reviews, Blind. Asses. = Blinded assessors, Blind. Part. = blinded participants, CI = confidence interval, CRP = C-reactive protein, DAS28 = Disease Activity Score 28, ESR = erythrocyte sedimentation rate, H/UC = high / unclear risk of bias, HAQ = Health Assessment Questionnaire, L = low risk of bias, NRT = non-randomised trial, RA = rheumatoid arthritis, Rand. Seq. = random sequence generation, RCT = randomised controlled trial, SD = standard deviation, SMD = Standardised mean difference, VAS = visual analogue scale

Table – Antioxidants (RA) cont., results and quality assessment

Outcome	Study (date) [study type]	Standardised result, SMD (95% CI) unless otherwise stated	Natural result	AMSTAR2 quality	Rand. Seq.	Alloc. Conc.	Blind. Part.	Blind. Asses.
Morning stiffness	Helmy (2001) [NRT][151]	Antioxidants vs Control at 2 months<br>SMD -1.40 (-2.39, -0.42)<br>Antioxidants + vit E vs Control at 2 months<br>SMD -1.51 (-2.52, -0.50)	Morning stiffness (mins), BL / 2 months, mean (SD)<br>Antioxidants: 67.5 (30.8) / 10.0 (12.5)<br>Antioxidants + vit E: 41.0 (37.8) / 7.5 (13.2)<br>Control: 54.5 (37.5) / 39.0 (26.4)					
CRP	Jalili (2014) [Single arm int.][152]		CRP, BL / 12 weeks, mean (SD)<br>Antioxidants: 5.50 (0.5) / 4.20 (0.51); p=0.003					
ESR	Batooei (2018) [RCT][146]	Antioxidants vs placebo at 12 weeks<br>SMD -0.12 (-0.67, 0.43)	ESR, BL / 12 weeks, mean (SD)<br>Antioxidant: 31.4 (19.6) / 25.2 (19.8)<br>Placebo: 29.2 (19.3) / 27.8 (23.7); p=0.6		H/UC	H/UC	L	L
	Helmy (2001) [NRT][151]	Antioxidants vs Control at 2 months<br>SMD -1.52 (-2.53, -0.51)<br>Antioxidants + vit E vs Control at 2 months<br>SMD -1.11 (-2.06, -0.16)	ESR, BL / 2 months, mean (SD)<br>Antioxidants: 63.0 (27.1) / 14.0 (7.0)<br>Antioxidants + vit E: 71.5 (21.1) / 18.0 (15.3)<br>Control: 54.5 (29.3) / 39.5 (22.7)					

Alloc. Conc. = allocation concealment, AMSTAR2 = Assessing the Methodological Quality of Systematic Reviews, Blind. Asses. = Blinded assessors, Blind. Part. = blinded participants, CI = confidence interval, CRP = C-reactive protein, DAS28 = Disease Activity Score 28, ESR = erythrocyte sedimentation rate, H/UC = high / unclear risk of bias, HAQ = Health Assessment Questionnaire, L = low risk of bias, NRT = non-randomised trial, RA = rheumatoid arthritis, Rand. Seq. = random sequence generation, RCT = randomised controlled trial, SD = standard deviation, SMD = Standardised mean difference, VAS = visual analogue scale

Supplementary table 102 – Vitamin B6 and RA progression, results

Table – Vitamin B6 (RA), results and quality assessment

Outcome	Study (date) [study type]	Standardised result, SMD (95% CI) unless otherwise stated	Natural result	AMSTAR2 quality	Rand. Seq.	Alloc. Conc.	Blind. Part.	Blind. Asses.
Disease activity	Huang (2010) [RCT][147]	Vitamin B6 vs control at 12 weeks<br>SMD -0.33 (-1.01, 0.34)	DAS28, BL / week 12, mean (SD)<br>Vitamin B6: 4.1 (0.3) / 4.2 (0.3)<br>Control: 4.1 (0.2) / 4.3 (0.3)		H/UC	H/UC	H/UC	H/UC
Tender joints	Huang (2010) [RCT][147]	Vitamin B6 vs control at 12 weeks<br>SMD -0.58 (-1.26, 0.10)	Tender joint count, BL / week 12, mean (SD)<br>Vitamin B6: 12.3 (4.1) / 11.6 (2.8)<br>Control: 9.2 (2.2) / 13.3 (3.1)		H/UC	H/UC	H/UC	H/UC
Swollen joints	Huang (2010) [RCT][147]	Vitamin B6 vs control at 12 weeks<br>SMD 0.55 (-0.13, 1.23)	Swollen joint count, BL / week 12, mean (SD)<br>Vitamin B6: 5.3 (2.6) / 3.3 (1.9)<br>Control: 2.6 (0.8) / 2.4 (1.2)		H/UC	H/UC	H/UC	H/UC
CRP	Huang (2010) [RCT][147]	Vitamin B6 vs control at 12 weeks<br>SMD 0.20 (-0.47, 0.87)	CRP, BL / week 12, mean (SD)<br>Vitamin B6: 0.3 (0.4) / 0.4 (0.4)<br>Control: 0.3 (0.2) / 0.3 (0.6)		H/UC	H/UC	H/UC	H/UC
	Chiang (2005) [RCT][149]		CRP, BL / 30 days, median (95% CI)<br>Vitamin B6: 2.0 (0.1, 17.2) / 3.0 (0.6, 14.8)<br>Placebo: 13.0 (19.4, 52.6) / 7.0 (4.4, 27.5); p<0.0001		H/UC	L	L	H/UC
ESR	Chiang (2005) [RCT][149]		ESR, BL / 30 days, median (95% CI)<br>Vitamin B6: 27.5 (18.8, 41.6) / 31.0 (22.4, 38.9)<br>Placebo: 31.0 (19.4, 52.6) / 32.0 (24.0, 49.7); p<0.0001)		H/UC	L	L	H/UC

Alloc. Conc. = allocation concealment, AMSTAR2 = Assessing the Methodological Quality of Systematic Reviews, BL = baseline, Blind. Asses. = Blinded assessors, Blind. Part. = blinded participants, CI = confidence interval, CRP = C-reactive protein, DAS28 = Disease Activity Score 28, ESR = erythrocyte sedimentation rate, H/UC = high / unclear risk of bias, L = low risk of bias, RA = rheumatoid arthritis, Rand. Seq. = random sequence generation, RCT = randomised controlled trial, SD = standard deviation, SMD = Standardised mean difference,

Supplementary table 103 – Vitamin D and RA progression, results

Table – Vitamin D (RA), results and quality assessment

Outcome	Study (date) [study type]	Standardised result, SMD (95% CI) unless otherwise stated	Natural result	AMSTAR2 quality	Rand. Seq.	Alloc. Conc.	Blind. Part.	Blind. Asses.
Pain	Franco (2017) [MA][145]		Pain<br>MD 2.79 (-1.87, 7.44)	Moderate				
Disease Activity	Franco (2017) [MA][145]		DAS<br>MD -0.31 (-0.86, 0.25)	Moderate				

Alloc. Conc. = allocation concealment, AMSTAR2 = Assessing the Methodological Quality of Systematic Reviews, Blind. Asses. = Blinded assessors, Blind. Part. = blinded participants, CI = confidence interval, DAS = Disease Activity Score, H/UC = high / unclear risk of bias, L = low risk of bias, MA = meta-analysis, MD = mean difference, RA = rheumatoid arthritis, Rand. Seq. = random sequence generation, SMD = Standardised mean difference,

Supplementary table 104 – Vitamin E and RA progression, results

Table – Vitamin E (RA), results and quality assessment

Outcome	Study (date) [study type]	Standardised result, SMD (95% CI) unless otherwise stated	Natural result	AMSTAR 2 quality	Rand. Seq.	Alloc. Conc.	Blind. Part.	Blind. Asses.
Pain	Edmonds (1997) [RCT][150]	Vitamin E vs placebo: pain in morning / evening / after chosen activity after 12 weeks<br>SMD -0.82 (-1.47, -0.16) / -0.68 (-1.32, -0.03) / -0.56 (-1.20, 0.08)	Pain in morning / evening / after chosen activity, mean (SD) change from bl<br>Vitamin E: -0.56 (1.53) / -0.56 (1.43) / -0.68 (1.52)<br>Placebo: 0.54 (1.12) / 0.28 (1.00) / 0.09 (1.19)		H/UC	H/UC	L	H/UC
Tender joints	Edmonds (1997) [RCT][150]	Vitamin E vs placebo<br>SMD 0.12 (-0.51, 0.75)	Ritchie Index, BL / 12 weeks, mean (SD)<br>Vitamin E: 15.9 (7.7) / 15.3 (10.0)<br>Placebo: 14.9 (8.8) / 14.0 (12.1)		H/UC	H/UC	L	H/UC
Swollen joints	Edmonds (1997) [RCT][150]	Vitamin E vs placebo<br>SMD -0.06 (-0.69, 0.57)	Swollen joint count, BL / 12 weeks, mean (SD)<br>Vitamin E: 9.2 (3.4) / 9.9 (5.0)<br>Placebo: 9.8 (5.4) / 10.2 (5.6)		H/UC	H/UC	L	H/UC
Morning stiffness	Edmonds (1997) [RCT][150]		Morning stiffness, BL / 12 weeks, median<br>Vitamin E: 45 / 30<br>Placebo: 30 / 20		H/UC	H/UC	L	H/UC
CRP	Aryaeian (2008) [RCT][137]	Vitamin E vs placebo<br>SMD -0.28 (-0.88, 0.32)<br>Linoleic acid + vitamin E vs placebo<br>SMD -0.49 (-1.08, 0.12)	CRP, BL / 12 weeks, mean (SD*)<br>Vitamin E: 9.06 (14.3) / 4.07 (4.5)<br>Vitamin E + linoleic acid: 5.23 (6.4) / 3.17 (3.9)<br>Placebo: 6.44 (7.9) / 5.48 (5.6)		H/UC	H/UC	H/UC	H/UC
ESR	Aryaeian (2008) [RCT][137]	Linoleic acid vs placebo<br>SMD 0.25 (-0.35, 0.85)<br>Linoleic acid + vitamin E vs placebo<br>SMD -0.58 (-1.19, 0.02)	CRP, BL / 12 weeks, mean (SD*)<br>Vitamin E: 40.43 (26.2) / 32.28 (23.0)<br>Vitamin E + linoleic acid: 28.45 (17.3) / 17.77 (12.2)<br>Placebo: 28.36 (21.5) / 27.04 (18.9)		H/UC	H/UC	H/UC	H/UC

*SD calculated from standard error reporting in paper

Alloc. Conc. = allocation concealment, AMSTAR2 = Assessing the Methodological Quality of Systematic Reviews, Blind. Asses. = Blinded assessors, Blind. Part. = blinded participants, CI = confidence interval, CRP = C-reactive protein, H/UC = high / unclear risk of bias, L = low risk of bias, RA = rheumatoid arthritis, Rand. Seq. = random sequence generation, RCT = randomised controlled trial, SD = standard deviation, SMD = Standardised mean difference,

Supplementary table 105 – Description of reviews of animal products in SLE

Table – Animal products (SLE), description of reviews

Authors (date)	Review type	Study type included	Exposure detail	Number of studies included	Funders
Rodriguez Huerta (2016)[154]	SR	RCTs, observational	Omega 3 consumption	3	**Government** (Spanish Ministry of Health, Social Affairs and Equality)

RCT = randomised controlled trial, SLE = systemic lupus erythematosus, SR = systematic review

Supplementary table 106 – Description of studies of animal products in SLE

Table – Animal products (SLE), description of included studies

Author (date) [country]	Study design	Inclusion criteria	Exposure detail	N	Age, mean (SD) years	N (%) female	Funders
Curado Borges (2017) [Brazil][155]	RCT	Aged 18-60 years, SLE ACR criteria, stable medication for SLE over last three months<br>Exclusions: pregnancy, disease duration <1 year, allergy to fish, fish oil or any omega-3 product, omega 3 use in the last 6 months, diabetes, liver disease, active nephritis, chronic renal failure, any type of infection	1) Two omega 3 tablets (540mg of EPA and 100mg of DHA)<br>p) No intervention and no placebo	1) 22<br>p) 27	Median (IQR)<br>37 (29-48)	1) 22 (100)<br>p) 27 (100)	**Government** (Fundac¸ão de Amparo à Pesquisa do Estadode Minas Gerais)
Arriens (2015) [USA][156]	RCT	Aged 18-64 years, 1997 ACR SLE criteria<br>Exclusions: Allergy to fish or fish oil, fish oil use within last two months, warfarin or heparin use, pregnancy	1) 6 fish oil tablets, taken as one or two doses per day (2.25g EPA and 2.25g DHA)<br>p) Olive oil	1) 18<br>p) 14	median (IQR)<br>1) 46.2 (36.8-49.1)<br>P) 35.6 (26.3-42.7)	1) 14 (77.8)<br>p) 11 (78.6)	**Government** (NIH)
Bello (2013) [USA][157]	RCT	Revised ACR SLE criteria<br>Exclusions: pregnancy, pregnancy plans, nursing, warfarin or heparin use, liver enzymes >2x ULN, allergy to fish, fish oil or omega 3 products, omega 3 use in previous 6 months, established coronary artery disease	1) 3g of omega 3 (1.8g EPA, 1.2g DHA)<br>p) Placebo made of corn starch	1) 42<br>p) 43	1) 48.9 (10.6)<br>p) 45.5 (10.8)	1) 41 (97.6)<br>p) 39 (90.7)	**Government** (NIAMS), **University** (Johns Hopkins University School of Medicine General Clinical Research Center)
Wright (2008) [UK][158]	RCT	ACR criteria for SLE<br>Exclusions: diabetes, hypertension, significant pulmonary, hepatic or renal disease, typical angina or myocardial infarction, cerebrovascular disease, history of transient ischaemic attack, use of antihypertensive, oral hypoglycaemic or lipid lowering agents, steroids >10mg prednisolone equivalent, pregnant / lactating women	1) 4 capsules of omega 3 per day (1.8g EPA and 1.2g DHA<br>p) Identical capsules containing olive oil	1) 30<br>p) 30	1) 48.5 (9.1)<br>p) 47.6 (9.6)	1) 29 (96.7)<br>p) 27 (90.0)	**Charity** (The Wellcome Trust, Lupus UK)

§ crossover design

ACR = American College of Rheumatology, ARA = American Rheumatism Association, DHA = docosahexaenoic acid, EPA = eicosapentaenoic acid, IQR = interquartile range, N = number, NIAMS = National Institute of Arthritis and Musculoskeletal and Skin Diseases, NIH = National Institutes of Health, NRT = non-randomised trial, RCT = randomised controlled trial, SD = standard deviation, SLE = systemic lupus erythematosus, UK = United Kingdom, USA = United States of America

Table – Animal products (SLE) cont., description of included studies

Author (date) [country]	Study design	Inclusion criteria	Exposure detail	N	Age, mean (SD) years	N (%) female	Funders
Duffy (2004) [UK][159]	RCT	Aged 18-80 years, active, stable SLE, SLE revised criteria<br>Exclusions: ongoing treatment for potentially life threatening disease, >10mg steroids, immunosuppressive drugs, vitamin or mineral supplements, taking omega 3 or copper supplements in previous 6 months, allergy to fish or copper	1) Fish oil (180mg EPA, 120mg DHA) and copper<br>2) Fish oil and placebo copper<br>3) Copper and placebo fish oil<br>p) Placebo fish oil and copper	1) 13<br>2) 14<br>3) 13<br>p) 12	1) 46 (13.17)<br>2) 50.7 (15.2)<br>3) 43.2 (15.8)<br>p) 43.2 (10.8)	9:1 female to male ratio	**Not reported**
Westberg (1990) [Sweden][160]	RCT §	ARA SLE criteria, no immunosuppressive drugs in last 6 months<br>Exclusions: no clinical signs of SLE	1) MaxEPA (omega 3)<br>p) Placebo	17	44.2 (6.6)	15 (88.2)	**Industry** (Seven Seas provided intervention)
Lozovoy (2015) [Brazil][161]	NRT	1997 ACR SLE criteria, stable prednisone treatment for 4 months<br>Exclusions: anti-hypertensive drugs	1) Fish oil<br>p) No fish oil	1) 41<br>p) 21	median (IQR)<br>1) 43.0 (32.0-51.0)<br>p) 42.5 (34.0-60.0)	1) 37 (90.2)<br>p) 20 (95.2)	**Government** (National Council of Brazilian Research), **Charity** (Araucária Foundation from the state of Paraná)

§ crossover design

ACR = American College of Rheumatology, ARA = American Rheumatism Association, DHA = docosahexaenoic acid, EPA = eicosapentaenoic acid, IQR = interquartile range, N = number, NIAMS = National Institute of Arthritis and Musculoskeletal and Skin Diseases, NIH = National Institutes of Health, NRT = non-randomised trial, RCT = randomised controlled trial, SD = standard deviation, SLE = systemic lupus erythematosus, UK = United Kingdom, USA = United States of America

Supplementary table 107 – Fish oil / omega 3 and SLE progression, results

Table – Fish oil / omega 3 (SLE), results and quality assessment

Outcome	Study (date) [study type]	Standardised result, SMD (95% CI) unless otherwise stated	Natural result	AMSTAR2 quality	Rand. Seq.	Alloc. Conc.	Blind. Part.	Blind. Asses.
Pain	Arriens (2015) [RCT][156]	Fish oil vs placebo at 6 months<br>SMD -1.00 (-1.74, -0.26)	SF36 pain score, 6 months, mean (SD §)<br>Fish oil: 37.5 (28.2)<br>Placebo: 70.4 (38.1)		L	H/UC	L	H/UC
Function	Arriens (2015) [RCT][156]	Fish oil vs placebo at 6 months<br>SMD -1.24 (-2.01, -0.48)	SF36 function score, 6 months, mean (SD §)<br>Fish oil: 32.1 (29.2)<br>Placebo: 74.2 (39.1)		L	H/UC	L	H/UC
Disease activity	Rodriguez Huerta (2016) [SR][154]		2/3 studies reported reductions in disease activity	Moderate				
	Bello (2013) [RCT][157]	Omega 3 vs placebo, change from BL-12 weeks<br>SMD -0.34 (-0.76, 0.09)	SLEDAI, change BL-12 weeks, mean (SD)<br>Omega 3: -0.17 (1.87)<br>Placebo: 0.51 (2.18); p=0.1122		H/UC	H/UC	H/UC	H/UC
	Duffy (2004) [RCT][159]		SLAM-R<br>Patients taking fish oil had a significant improvement in disease activity compared to those not taking fish oil		H/UC	H/UC	L	H/UC
	Westberg (1990) [RCT][160]	Omega 3 vs placebo at 3 months<br>SMD -0.20 (-0.88, 0.47)	Clinical score†, BL / 3 months, mean (SD)<br>Omega 3: 1.49 (1.03) / 1.36 (1.28)<br>Placebo: 1.41 (0.943) / 1.64 (1.50)		L	L	H/UC	L
	Lozovoy (2015) [NRT][161]	Omega 3 vs control at 120 days<br>SMD 0.00 (-0.53, 0.53)	SLEDAI, BL / 120 days, mean (SD §)<br>Omega 3: 4.0 (7.7) / 2.0 (4.6)<br>Control: 2.0 (3.2) / 2.0 (3.2)					
Fatigue	Arriens (2015) [RCT][156]	Fish oil vs placebo at 6 months<br>SMD -0.49 (-1.20, 0.22)	SF36 fatigue score, 6 months, mean (SD §)<br>Fish oil: 37.9 (29.2)<br>Placebo: 52.5 (30.9)		L	H/UC	L	H/UC
Physician global	Bello (2013) [RCT][157]	Omega 3 vs placebo, change from BL-12 weeks<br>SMD -0.29 (-0.71, 0.14)	Physician global assessment, change BL-12 weeks, mean (SD)<br>Omega 3: 0.07 (0.54)<br>Placebo: 0.21 (0.44); p=0.2914		H/UC	H/UC	H/UC	H/UC

§ Mean (SD) estimated from median (interquartile range) using published formula[61]

† Clinical score made up of fatigue, pain, comorbidities, joint involvement and morning stiffness

Alloc. Conc. = allocation concealment, AMSTAR2 = Assessing the Methodological Quality of Systematic Reviews, Anti-dsDNA = anti double strand deoxyribonucleic acid, BL = baseline, Blind. Asses. = Blinded assessors, Blind. Part. = blinded participants, CI = confidence interval, CRP = C-reactive protein, ESR = erythrocyte sedimentation rate, H/UC = high / unclear risk of bias, L = low risk of bias, Rand. Seq. = random sequence generation, SLAM-R = Systemic lupus activity measure – revised, SLE = systemic lupus erythematosus, SLEDAI = systemic lupus erythematosus disease activity index, SMD = Standardised mean difference, SR = systematic review

Table – Fish oil / omega 3 (SLE) cont., results and quality assessment

Outcome	Study (date) [study type]	Standardised result, SMD (95% CI) unless otherwise stated	Natural result	AMSTAR2 quality	Rand. Seq.	Alloc. Conc.	Blind. Part.	Blind. Asses.
CRP	Curado Borges (2017) [RCT][155]	Omega 3 vs placebo at 12 weeks<br>SMD -0.37 (-0.94, 0.20)	CRP, BL / 12 weeks, mean (SD §)<br>Omega 3: 6.0 (2.5) / 5.7 (1.8)<br>Control: 7.6 (5.2) / 7.2 (5.2); p=0.370		H/UC	H/UC	H/UC	H/UC
	Wright (2008) [RCT][158]	Omega 3 vs placebo at 24 weeks<br>SMD 0.14 (-0.37, 0.65)	CRP, BL / 24 weeks, mean (SD)<br>Omega 3: 9 (13) / 6 (6)<br>Placebo: 5 (9) / 5 (8); p=0.988		H/UC	L	L	L
	Bespoke meta-analysis	Omega 3 vs placebo<br>SMD -0.10 (-0.60, 0.40), I^2 = 42.4%						
ESR	Wright (2008) [RCT][158]	Omega 3 vs placebo at 24 weeks<br>SMD 0.47 (-0.05, 0.98)	ESR, BL / 24 weeks, mean (SD)<br>Omega 3: 33 (30) / 32 (31)<br>Placebo: 19 (14) / 20 (19); p=0.868		H/UC	L	L	L
Anti-dsDNA	Wright (2008) [RCT][158]	Omega 3 vs placebo at 24 weeks<br>SMD 0.03 (-0.47, 0.54)	Anti-dsDNA, BL / 24 weeks, mean (SD)<br>Omega 3: 110 (75) / 126 (761) [sic]<br>Placebo: 95 (55) / 108 (83); p=0.521		H/UC	L	L	L
	Lozovoy (2015) [NRT][161]	Omega 3 vs control at 120 days<br>SMD -0.29 (-0.82, 0.24)	Anti-dsDNA, BL / 120 days, mean (SD §)<br>Omega 3: 6.7 (15.4) / 1.7 (3.8)<br>Control: 3.3 (8.0) / 3.3 (8.0)					

§ Mean (SD) estimated from median (interquartile range) using published formula[61]

† Clinical score made up of fatigue, pain, comorbidities, joint involvement and morning stiffness

Alloc. Conc. = allocation concealment, AMSTAR2 = Assessing the Methodological Quality of Systematic Reviews, Anti-dsDNA = anti double strand deoxyribonucleic acid, BL = baseline, Blind. Asses. = Blinded assessors, Blind. Part. = blinded participants, CI = confidence interval, CRP = C-reactive protein, ESR = erythrocyte sedimentation rate, H/UC = high / unclear risk of bias, L = low risk of bias, Rand. Seq. = random sequence generation, SLE = systemic lupus erythematosus, SLEDAI = systemic lupus erythematosus disease activity index, SMD = Standardised mean difference, SR = systematic review

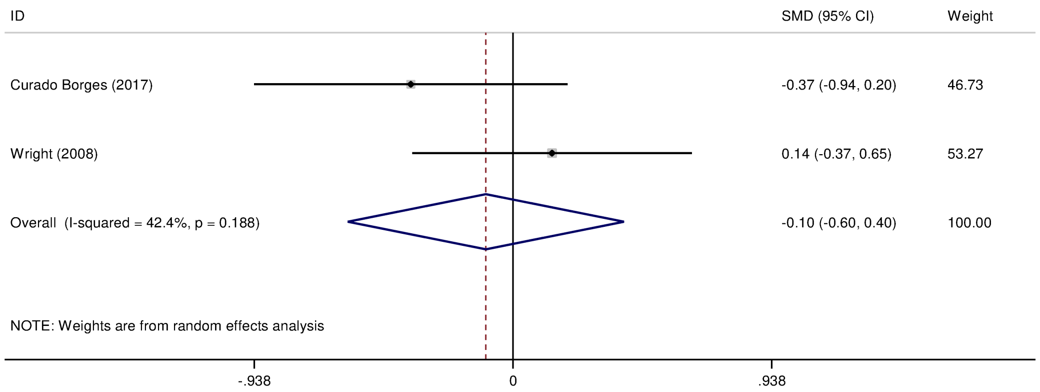

Supplementary figure 11 – Omega 3, bespoke meta-analysis for CRP [SLE]

Supplementary table 108 – Description of reviews of experimental diets in SLE

Table – Experimental diets (SLE), description of reviews

Authors (date)	Review type	Study type included	Exposure detail	Number of studies included	Funders
del Pino-Sedeno (2016)[162]	SR	RCTs, observational	Low glycaemic vs low calorie diet	1	**Government** (Spanish Ministry of Economy and Finance)
Rodriguez Huerta (2016)[154]	SR	RCTs, observational	Low glycaemic vs low calorie diet / dietary education program	2	**Government** (Spanish Ministry of Health, Social Affairs and Equality)
Yuen (2014)[163]	SR	RCTs	Low glycaemic vs low calorie diet	1	**Non reported** – authors declare no conflict of interest

RCT = randomised controlled trial, SLE = systemic lupus erythematosus, SR = systematic review

Supplementary table 109 – Description of studies of experimental diets in SLE

Table – Cholesterol lowering diet (SLE), description of included studies

Author (date) [country]	Study design	Inclusion criteria	Exposure detail	N	Age, mean (SD) years	N (%) female	Funders
Shah (2002) [USA][164]	RCT	Symptom duration >6 months, LDL cholesterol >100 mg/dl, able to read to 5th grade level<br>Exclusions: pregnant, lactating, taking ≥20mg of prednisone per day, ≥20 units per week of alcohol, inadequate cognitive ability	1) Educated using the National Cholesterol Education Program via group counselling and telephone<br>p) No dietary advice	1) 8<br>p) 8	1) 44.1 (9.3)<br>p) 45.3 (11.7)	1) 8 (100)<br>p) 8 (100)	**University** (University of Southwestern Medical Center)

N = number, SD = standard deviation, SLE = systemic lupus erythematosus, USA = United States of America

Supplementary table 110 – Experimental diets and SLE progression, results

Table – Cholesterol lowering diet (SLE), results and quality assessment

Outcome	Study (date) [study type]	Standardised result, SMD (95% CI) unless otherwise stated	Natural result	AMSTAR2 quality	Rand. Seq.	Alloc. Conc.	Blind. Part.	Blind. Asses.
Disease activity	Rodriguez Huerta (2016) [SR][154]		No difference between diet and control	Moderate				
Fatigue	del Pino-Sedeno (2016) [SR][162]		Both diets reduced fatigue equally effectively, neither reduced fatigue more than the MCID	Moderate				
	Rodriguez Huerta (2016) [SR][154]		Both diets reduced fatigue equally effectively, neither reduced fatigue more than the MCID	Moderate				
	Yuen (2014) [SR][163]		Both diets reduced fatigue equally effectively, neither reduced fatigue more than the MCID	Low				
QoL	Shah (2002) [RCT][164]	Diet intervention vs control at 12 weeks<br>SMD 1.04 (-0.01, 2.10)	QoL §, BL / 12 weeks, mean (SD)<br>Diet intervention: 59.4 (7.8) / 68.4 (7.8)<br>Control: 56.3 (15.1) / 53.8 (18.2)		H/UC	H/UC	H/UC	H/UC

§ Assessed using a VAS, higher scores = greater quality of life

Alloc. Conc. = allocation concealment, AMSTAR2 = Assessing the Methodological Quality of Systematic Reviews, BL = baseline, Blind. Asses. = Blinded assessors, Blind. Part. = blinded participants, CI = confidence interval, H/UC = high / unclear risk of bias, L = low risk of bias, MCID = minimum clinically important difference, QoL = Quality of life, Rand. Seq. = random sequence generation, RCT = randomised controlled trial, SD = standard deviation, SLE = systemic lupus erythematosus, SMD = Standardised mean difference, SR = systematic review

Supplementary table 111 – Description of studies of food components in SLE

Table – Food elements (SLE), description of included studies

Author (date) [country]	Study design	Inclusion criteria	Exposure detail	N	Age, mean (SD) years	N (%) female	Funders
Minami (2011) [Japan][165]	Pros. Cohort	SLE	Various dietary components from the food frequency questionnaire	216	40.6 (13.3)	216 (100)	**Government** (Ministry of Education, Culture, Sports, Science and Technology, Japan)
Minami (2003) [Japan][166]	Pros. Cohort	1982 SLE criteria<br>Exclusions: patients with serious symptoms (e.g. terminal symptoms and severe neuropsychiatric symptoms)	Various dietary components from the food frequency questionnaire	279	40.6 (13.7)	279 (100)	**Government** (Ministry of Health, Labour and Welfare, Japan
Karlson (1997) [USA][167]	Retro. Cohort	ACR criteria for SLE, all seen within 7 years of diagnosis	Adequacy of diet based on Food Frequency Questionnaire	200	52	186 (93.0)	**Government** (NIH), **Charity** (Arthritis Foundation)

N = number, SD = standard deviation, SLE = systemic lupus erythematosus

Supplementary table 112 – Food components and SLE progression, results

Table – Food elements (SLE), results and quality assessment

Outcome (outcome measure)	Study (date) [study type]	Standardised result, SMD (95% CI) unless otherwise stated	Natural result	Study Pop.	Attr.	Prog. Meas.	Outc. Meas.	Conf.	Stats.
Active disease	Minami (2011) [Pros. Obs.] [165]		Risk of active disease, middle tertile / upper tertile [lower tertile = ref], HR (95% CI)<br>Vitamin B6: 0.73 (0.35, 1.50) / 0.41 (0.18, 0.97)<br>Vitamin B12: 1.21 (0.58, 2.52) / 1.06 (0.49, 2.33)<br>Folate: 0.93 (0.45, 1.90) / 0.58 (0.25, 1.33)<br>Total fibre: 0.86 (0.44, 1.71) / 0.29 (0.11, 0.78)<br>Soluble fibre: 0.67 (0.33, 1.36) / 0.43 (0.18, 0.99)<br>Insoluble fibre: 0.98 (0.49, 1.96) / 0.39 (0.15, 0.97)	L	L	M	M	M	L
	Minami (2003) [Pros. Obs.][166]		Risk of active disease, middle tertile / upper tertile [lower tertile = ref], RR (95% CI)<br>total energy: 0.63 (0.30,1.32) / 0.84 (0.40, 1.76)<br>total protein*: 0.89 (0.42,1.90) / 0.90 (0.43, 1.89)<br>Total fat*: 1.86 (0.82, 4.24) / 1.49 (0.62, 3.58)<br>Cholesterol: 1.57 (0.72, 3.42) / 1.29 (0.59, 2.84)<br>Calcium: 0.97 (0.45, 2.12) / 1.07 (0.51, 2.27)<br>Salt: 1.11 (0.54, 2.27) / 0.81 (0.37, 1.79)<br>Crude fibre: 0.99 (0.49, 2.02) / 0.43 (0.18, 1.05)<br>Vit A: 0.65 (0.32, 1.34) / 0.50 (0.22, 1.14)<br>Retinol: 1.61 (0.74, 3.53) / 0.97 (0.43, 2.19)<br>Carotene: 0.59 (0.27, 1.26) / 0.68 (0.32, 1.46)<br>Vit B1: 1.00 (0.48, 2.07) / 0.59 (0.25, 1.36)<br>Vit B2: 1.10 (0.53, 2.28) / 0.75 (0.34, 1.67)<br>Niacin: 1.11 (0.53, 2.29) / 0.83 (0.37, 1.86)<br>Vit C: 0.52 (0.25, 1.08) / 0.26 (0.10, 0.67)<br>Vit D: 1.29 (0.60, 2.76) / 0.95 (0.43, 2.09)<br>Vit E: 0.62 (0.30, 1.32) / 0.56 (0.25, 1.25)	L	L	M	M	M	L
Atherosclerotic vascular events	Minami (2011) [Pros. Obs.] [165]		Risk of atherosclerotic vascular events, middle tertile / upper tertile [lower tertile = ref], HR (95% CI)<br>Vitamin B6: 1.04 (0.35, 3.10) / 0.41 (0.10, 1.72)<br>Vitamin B12: 0.87 (0.23, 3.35) / 1.86 (0.60, 5.82)<br>Folate: 0.56 (0.16, 1.99) / 0.83 (0.23, 2.99)<br>Total dietary fibre: 1.69 (0.48, 6.02) / 0.89 (0.21, 3.74)<br>Soluble dietary fibre: 1.61 (0.46, 5.66) / 0.83 (0.22, 3.15)<br>Insoluble dietary fibre: 0.90 (0.28, 2.91) / 0.39 (0.10, 1.51)	L	L	M	M	M	L

Attr. = attrition, CI = confidence interval, Conf. = confounding, HR = hazard ratio, L = low risk of bias, M = moderate risk of bias, Outc. Meas = outcome measurement, Prog. Meas. = prognostic factor measurement, Pros. Obs. = prospective observational, RR = risk ratio, SLE = systemic lupus erythematosus, Stats. = statistical analysis, Study Pop. = study population

Supplementary table 113 – Poor nutrition and SLE progression, results

Table – Poor nutrition (SLE), results and quality assessment

Outcome (outcome measure)	Study (date) [study type]	Standardised result, SMD (95% CI) unless otherwise stated	Natural result	Study Pop.	Attr.	Prog. Meas.	Outc. Meas.	Conf.	Stats.
Organ damage	Karlson (1997) [Retro. Cohort][167]		<u>Organ damage</u><br>Lower calorie intake: beta = 0.81, p=0.0018	L	na	M	L	L	L
Mental health	Karlson (1997) [Retro. Cohort][167]		<u>Worse mental health</u><br>lower % protein in diet: p=0.01, t=2.5	L	na	M	L	L	L

Attr. = attrition, CI = confidence interval, Conf. = confounding, HR = hazard ratio, L = low risk of bias, M = moderate risk of bias, Outc. Meas = outcome measurement, Prog. Meas. = prognostic factor measurement, Pros. Obs. = prospective observational, RR = risk ratio, SLE = systemic lupus erythematosus, Stats. = statistical analysis, Study Pop. = study population, t = t-statistic

Supplementary table 114 – Description of studies of fruits, vegetables and other plant based interventions in SLE

Table – Fruits, vegetables and other plant based interventions (SLE), description of included studies

Author (date) [country]	Study design	Inclusion criteria	Exposure detail	N	Age, mean (SD) years	N (%) female	Funders
Shamekhi (2017) [Iran][168]	RCT	Aged 15-55 years, 2012 ACR criteria<br>Exclusions: any change in medication because of disease exacerbation or any other reason, pregnancy or lactation, smoking, alcohol and drug abuse, antioxidants or vitamin supplementation within last 6 months, engaged in heavy exercise or weight reduction programs, history of autoimmune disease	1) 1000mg green tea extract<br>p) Starch	1) 32<br>p) 36	1) 38.9 (10.4)<br>p) 39.3 (10.5)	1) 32 (100)<br>p) 36 (100)	**University** (Ahvaz Jundishapur University of Medical Sciences)
Singgih Wahono (2017) [Indonesia][169]	RCT	SLE 1997 ACR criteria, SLEDAI >3, 25(OH)D level <30<br>Exclusions: Pregnant, taking supplements containing curcumin and vitamin D, had liver function disorders, impaired renal function, severe infections such as tuberculosis, pneumonia or HIV	1) Curcumin + vitamin D<br>p) Placebo + vitamin D	1) 19<br>p) 20	1) 27.9 (7.9)<br>p) 30.3 (10.0)	Not reported	Not reported – no conflicts of interest stated

ACR = American College of Rheumatology, HIV = human immunodeficiency virus, N = number, RCT = randomised controlled trial, SD = standard deviation, SLE = systemic lupus erythematosus, SLEDAI = systemic lupus erythematosus disease activity index

Supplementary table 115 – Curcumin and SLE progression, results

Table – Curcumin (SLE), results and quality assessment

Outcome	Study (date) [study type]	Standardised result, SMD (95% CI) unless otherwise stated	Natural result	AMSTAR2 quality	Rand. Seq.	Alloc. Conc.	Blind. Part.	Blind. Asses.
Disease activity	Singgih Wahono (2017) [RCT][169]	Curcumin vs placebo at 3 months<br>SMD 0.02 (-0.61, 0.64)	SLEDAI at 3 months, mean(SD)<br>Curcumin: 9.2 (7.4)<br>Placebo: 9.1 (5.6)		H/UC	H/UC	H/UC	H/UC

Alloc. Conc. = allocation concealment, AMSTAR2 = Assessing the Methodological Quality of Systematic Reviews, Blind. Asses. = Blinded assessors, Blind. Part. = blinded participants, CI = confidence interval, H/UC = high / unclear risk of bias, L = low risk of bias, Rand. Seq. = random sequence generation, RCT = randomised controlled trial, SD = standard deviation, SLE = systemic lupus erythematosus, SLEDAI = systemic lupus erythematosus disease activity index, SMD = Standardised mean difference,

Supplementary table 116 – Green tea extract and SLE progression, results

Table – Green tea extract (SLE), results and quality assessment

Outcome	Study (date) [study type]	Standardised result, SMD (95% CI) unless otherwise stated	Natural result	AMSTAR2 quality	Rand. Seq.	Alloc. Conc.	Blind. Part.	Blind. Asses.
Disease activity	Shamekhi (2017) [RCT][168]	Green tea extract vs placebo at 12 weeks<br>SMD -0.03 (-0.50, 0.45)	SLEDAI, BL / 12 weeks, mean (SD)<br>Green tea extract: 4.66 (3.32) / 2.78 (3.2)<br>Placebo: 3.17 (3.21) / 2.86 (3.16); p=0.78		L	H/UC	L	L

Alloc. Conc. = allocation concealment, AMSTAR2 = Assessing the Methodological Quality of Systematic Reviews, Blind. Asses. = Blinded assessors, Blind. Part. = blinded participants, CI = confidence interval, H/UC = high / unclear risk of bias, L = low risk of bias, Rand. Seq. = random sequence generation, RCT = randomised controlled trial, SD = standard deviation, SLE = systemic lupus erythematosus, SMD = Standardised mean difference,

Table – Green tea extract, SF12 results at final follow-up, mean (SD) / median (IQR)

Author (date)	PCS	MCS	GH	PF	RP	RE	SF	BP	V	MH
Shamekhi (2017)[168] – Green tea extract			54.3 (20.1)	89.8 (76.3, 0[sic])	69.8 (23.0)	55.6 (27.4)	54.6 (32.0, 78.4)	63.1 (44.8, 84.3)	81 (63.1, 95.5)	60.7 (24.9)
Shamekhi (2017)[168] - Placebo			37.9 (28.8)	55 (21.2, 86.4)	54.6 (30.4)	55.5 (27.8)	58.9 (33.6, 85.2)	35.9 (37.7, 79.6)	56.2 (28.1, 84.3)	58.7 (28.1)

BP = bodily pain, GH = general health, IQR = interquartile range, MCS = mental component score, MH = mental health, PCS = physical component score, PF = physical function, RE = role emotional, RP = role physical, SD = standard deviation, SF = social functioning, V = vitality

Supplementary table 117 – Description of studies of minerals and supplements in SLE

Table – Minerals and supplements (SLE), description of included studies

Author (date) [country]	Study design	Inclusion criteria	Exposure detail	N	Age, mean (SD) years	N (%) female	Funders
Duffy (2004) [UK][159]	RCT	Aged 18-80 years, active, stable SLE, SLE revised criteria<br>Exclusions: ongoing treatment for potentially life threatening disease, >10mg steroids, immunosuppressive drugs, vitamin or mineral supplements, taking omega 3 or copper supplements in previous 6 months, allergy to fish or copper	1) Fish oil (180mg EPA, 120mg DHA) and copper<br>2) Fish oil and placebo copper<br>3) Copper and placebo fish oil<br>p) Placebo fish oil and copper	1) 13<br>2) 14<br>3) 13<br>p) 12	1) 46 (13.17)<br>2) 50.7 (15.2)<br>3) 43.2 (15.8)<br>p) 43.2 (10.8)	9:1 female to male ratio	**Not reported**
Al-Kushi (2018) [Saudi Arabia][170]	NRT	Exclusions: Patients who had malabsorption, renal and liver disease, chronic diarrheal illnesses and irritable bowel syndrome, antifungal or anticonvulsant medications, received vitamin D and / or calcium supplementation past 6 months	1) 1250mg calcium + 1400 IU vitamin D + steroids<br>p1) no treatment and no supplementation<br>p2) received steroids but no supplementation	1) 30<br>p1) 21<br>p2) 30	1) 37.7 (8.9)<br>p1) 36.4 (7.6)<br>p2) 35.2 (8.7)	66 (81.5)	No financial support

IU = International Units, mg = milligram, N = number, NRT = Non-randomised trial, SD = standard deviation, SLE = systemic lupus erythematosus

Supplementary table 118 – Calcium + vitamin D and SLE progression, results

Table – Calcium + vitamin D (SLE), results and quality assessment

Outcome	Study (date) [study type]	Standardised result, SMD (95% CI) unless otherwise stated	Natural result	AMSTAR2 quality	Rand. Seq.	Alloc. Conc.	Blind. Part.	Blind. Asses.
Disease Activity	Al-Kushi (2018) [NRT][170]	Calcium + vitamin D vs no treatment no supplements at 6 months<br>SMD -1.11 (-1.70, -0.51)<br>Calcium + vitamin D vs steroids & no supplements at 6 months<br>SMD 0.00 (-0.51, 0.51)	SLEDAI at 6 months, mean (SD)<br>Calcium + vitamin D: 4.5 (0.5)<br>No treatment, no supplementation: 5.1 (0.6)<br>Steroids, no supplementation: 4.5 (0.6)					
ESR	Al-Kushi (2018) [NRT][170]	Calcium + vitamin D vs no treatment no supplements at 6 months<br>SMD -0.68 (-1.26, -0.11)<br>Calcium + vitamin D vs steroids & no supplements at 6 months<br>SMD -0.09 (-0.60, 0.41)	ESR at 6 months, mean (SD)<br>Calcium + vitamin D: 45.2 (16.5)<br>No treatment, no supplementation: 56.7 (17.4)<br>Steroids, no supplementation: 46.6 (13.7)					
Anti-dsDNA	Al-Kushi (2018) [NRT][170]	Calcium + vitamin D vs no treatment no supplements at 6 months<br>SMD -0.15 (-0.71, 0.41)<br>Calcium + vitamin D vs steroids & no supplements at 6 months<br>SMD 0.00 (-0.51, 0.51)	Anti-dsDNA at 6 months, mean (SD)<br>Calcium + vitamin D: 50.6 (28.8)<br>No treatment, no supplementation: 55.2 (31.9)<br>Steroids, no supplementation: 50.6 (22.4)					

Alloc. Conc. = allocation concealment, AMSTAR2 = Assessing the Methodological Quality of Systematic Reviews, Anti-dsDNA = anti double strand deoxyribonucleic acid, Blind. Asses. = Blinded assessors, Blind. Part. = blinded participants, CI = confidence interval, ESR = erythrocyte sedimentation rate, H/UC = high / unclear risk of bias, L = low risk of bias, NRT = Non-randomised trial, Rand. Seq. = random sequence generation, SLE = systemic lupus erythematosus, SLEDAI = systemic lupus erythematosus disease activity index, SMD = Standardised mean difference,

Supplementary table 119 – Copper and SLE progression, results

Table – Copper (SLE), results and quality assessment

Outcome	Study (date) [study type]	Standardised result, SMD (95% CI) unless otherwise stated	Natural result	AMSTAR2 quality	Rand. Seq.	Alloc. Conc.	Blind. Part.	Blind. Asses.
Disease Activity	Duffy (2004) [RCT][159]		SLAM-R at 24 weeks<br>Copper vs no copper: no significant change		H/UC	H/UC	L	H/UC

Alloc. Conc. = allocation concealment, AMSTAR2 = Assessing the Methodological Quality of Systematic Reviews, Blind. Asses. = Blinded assessors, Blind. Part. = blinded participants, CI = confidence interval, H/UC = high / unclear risk of bias, L = low risk of bias, Rand. Seq. = random sequence generation, SLAM-R = Systemic lupus activity measure – revised , SLE = systemic lupus erythematosus, SMD = Standardised mean difference,

Supplementary table 120 – Description of reviews of vitamins in SLE

Table – Vitamins (SLE), description of reviews

Authors (date)	Review type	Study type included	Exposure detail	Number of studies included	Funders
Franco (2017)[145]	MA	RCTs	Vitamin D	3	**Charity** (São Paulo Research Foundation, Federico Foundation), **Government** (National Council for Scientific and Technological Development)
Yuen (2014)[163]	SR	RCTs	Vitamin D	1	**Non reported** – authors declare no conflict of interest

MA = meta-analysis, RCT = randomised controlled trial, SLE = systemic lupus erythematosus

Supplementary table 121 – Description of studies of vitamins in SLE

Table – Vitamins (SLE), description of included studies

Author (date) [country]	Study design	Inclusion criteria	Exposure detail	N	Age, mean (SD) years	N (%) female	Funders
Karimzadeh (2017) [Iran][171]	RCT	Aged >18 years, fulfilled 4 of the ACR 1982 criteria, had vitamin D levels <30ng/ml<br>Exclusions: history of any chronic systematic or inflammatory disease which affects vitamin D absorption, cirrhosis, myocardial infarction, malignancy, renal stones, hypercalcemia, hospitalisation due to complications of SLE	1) Vitamin D – 50,000 units/weekly for 12 weeks and then 50,000 units/month for 6 months<br>p) No details	1) 45<br>p) 45	1) 33.8 (6.2)<br>p) 35.7 (6.8)	1) 40 (88.9)<br>p) 41 (91.1)	No financial support and no conflicts of interest
Andreoli (2015) [Italy][172]	RCT	Premenopause, 1997 SLE criteria, absence of disease flare, SLEDAI <6, no vitamin D supplements for 1 month	1) 300,000 IU vitamin D at baseline and 50,000 monthly thereafter<br>p) 25,000 IU vitamin D monthly	1) 18<br>p) 16	Median (range)<br>1) 34 (24, 43)<br>2) 26 (19,44)	1) 18 (100)<br>p) 16 (100)	**Government** (Government of Lombardy), **University** (University of Brescia)

N = number, RCT = randomised controlled trial, SD = standard deviation, SLE = systemic lupus erythematosus, SLEDAI = systemic lupus erythematosus disease activity index

Supplementary table 122 – Vitamin D and SLE progression, results

Table – Vitamin D (SLE), results and quality assessment

Outcome	Study (date) [study type]	Standardised result, SMD (95% CI) unless otherwise stated	Natural result	AMSTAR2 quality	Rand. Seq.	Alloc. Conc.	Blind. Part.	Blind. Asses.
Disease activity	Franco (2017) [MA][145]		SLEDAI / ECLAM, mean<br>Intervention: 3.00 / 1.80<br>Control: 5.4 / 2.75, p=0.06 / 0.121	Moderate				
	Karimzadeh (2017) [RCT][171]	Vitamin D vs control and 6 months<br>SMD -0.18 (-0.60, 0.23)	SLEDAI BL / 6 months, mean (SD)<br>Vitamin D: 3.09 (2.36) / 1.62 (1.25)<br>Control: 3.09 (1.2) / 1.98 (2.47)		H/UC	H/UC	H/UC	H/UC
Fatigue	Yuen (2014) [SR][163]		One study reported a reduction in fatigue, but less than the MCID	Low				
Anti-dsDNA	Franco (2017) [MA][145]		Anti-dsDNA, risk difference<br>-0.10 (-0.18, -0.03); p=0.005	Moderate				
	Andreoli (2015) [RCT][172]	High dose vitamin D vs low dose vitamin D at 1 year<br>SMD 0.04 (-0.63, 0.72)	Anti-dsDNA, BL / 1 year, mean (SD §)<br>High dose vitamin D: 12.2 (16.8) / 13.4 (19.9)<br>Low dose vitamin D: 11.9 (14.7) / 12.6 (18.7)		H/UC	H/UC	H/UC	H/UC

§ mean (SD) estimated from median (interquartile range) using published forumla[61]

Alloc. Conc. = allocation concealment, AMSTAR2 = Assessing the Methodological Quality of Systematic Reviews, Anti-dsDNA = anti double strand deoxyribonucleic acid, Blind. Asses. = Blinded assessors, Blind. Part. = blinded participants, CI = confidence interval, ECLAM = European Consensus Lupus Activity Measurement, H/UC = high / unclear risk of bias, L = low risk of bias, MA = meta-analysis, MCID = minimum clinically important difference, Rand. Seq. = random sequence generation, RCT = randomised controlled trial, SD = standard deviation, SLE = systemic lupus erythematosus, SLEDAI = systemic lupus erythematosus disease activity index, SMD = Standardised mean difference

Supplementary table 123 – Description of reviews of food components in AS

Table – Food components (AS), description of reviews

Authors (date)	Review type	Study type included	Exposure detail	Number of studies included	Funders
Macfarlane (2018)[173]	SR	RCTs, observational	Diet	16	**Charity** (National Ankylosing Spondylitis Society)

AS = ankylosing spondylitis, MA = meta-analysis, RCT = randomised controlled trial

Supplementary table 124 – Food components and AS progression , results

Table – Food components (AS), results and quality assessment

Outcome	Study (date) [study type]	Standardised result, SMD (95% CI) unless otherwise stated	Natural result	AMSTAR2 quality	Rand. Seq.	Alloc. Conc.	Blind. Part.	Blind. Asses.
Disease activity	Macfarlane (2018) [SR][173]		BASDAI<br>Alpha-linoleic acid = no association<br>Carbohydrates = no association<br>Fat = One study reported an association in females only, the other reported no association<br>Linoleic acid = no association<br>Long-chain omega 3 fatty acids = no association<br>Polyunsaturated fatty acids = no association<br>Protein = no association<br>Saturated fatty acids = no association	Moderate				
CRP	Macfarlane (2018) [SR][173]		BASDAI<br>Alpha-linoleic acid = no association<br>Carbohydrates = no association<br>Fat = no association<br>Linoleic acid = no association<br>Long-chain omega 3 fatty acids = no association<br>Polyunsaturated fatty acids = no association<br>Protein = no association<br>Saturated fatty acids = no association	Moderate				
ESR	Macfarlane (2018) [SR][173]		BASDAI<br>Alpha-linoleic acid = no association<br>Carbohydrates = no association<br>Fat = no association<br>Linoleic acid = no association<br>Long-chain omega 3 fatty acids = sign. association<br>Polyunsaturated fatty acids = sign. association<br>Protein = no association<br>Saturated fatty acids = no association	Moderate				

Alloc. Conc. = allocation concealment, AMSTAR2 = Assessing the Methodological Quality of Systematic Reviews, AS = ankylosing spondylitis, BASDAI = Bath Ankylosing Spondylitis Disease Activity Index, Blind. Asses. = Blinded assessors, Blind. Part. = blinded participants, CI = confidence interval, CRP = C-reactive protein, ESR = erythrocyte sedimentation rate, H/UC = high / unclear risk of bias, L = low risk of bias, Rand. Seq. = random sequence generation, SMD = Standardised mean difference,

Supplementary table 125 – Description of studies of minerals and supplements in AS

Table – Minerals and supplements (AS), description of included studies

Author (date) [country]	Study design	Inclusion criteria	Exposure detail	N	Age, mean (SD) years	N (%) female	Funders
Jenks (2010) [New Zealand][174]	RCT	European Spondylarthropathy Study Group criteria, BASDAI ≥3, BASFI ≥3, Maastricht Ankylosing Spondylitis Enthesitis Score ≥2 or peripheral joint count ≥2<br>Exclusions: Pregnant, <18 years of age, diagnosis of irritable bowel disease, severe immunosuppression or current gastrointestinal infection	1) Probiotic formulation containing 3 strains of bacteria<br>p) Placebo powder	1) 32<br>p) 31	1) 45.5 (15)<br>p) 41.1 (10)	1) 13 (40.6)<br>p) 10 (32.3)	**Charity** (Arthritis New Zealand, Tony Hocken Research Scholarship)

AS = ankylosing spondylitis, BASDAI = Bath Ankylosing Spondylitis Disease Activity Index, BASFI = Bath Ankylosing Spondylitis Function Index, N = number, RCT = randomised controlled trial, SD = standard deviation

Supplementary table 126 – Probiotics and AS progression, results

Table – Probiotics (AS), results and quality assessment

Outcome	Study (date) [study type]	Standardised result, SMD (95% CI) unless otherwise stated	Natural result	AMSTAR2 quality	Rand. Seq.	Alloc. Conc.	Blind. Part.	Blind. Asses.
Pain	Jenks (2010) [RCT][174]	Probiotic vs placebo at week 12<br>SMD 0.04 (-0.45, 0.54)	Pain VAS, BL / week 12, mean (SD)<br>Probiotic: 2.9 (2.3) / 2.7 (2.5)<br>Placebo: 3.0 (2.6) / 2.6 (2.2)		L	L	L	L
Function	Jenks (2010) [RCT][174]	Probiotic vs placebo at week 12<br>SMD -0.10 (-0.59, 0.40)	BASFI, BL / week 12, mean (SD)<br>Probiotic: 3.5 (2.0) / 2.9 (1.9)<br>Placebo: 3.6 (1.9) / 3.1 (2.2)		L	L	L	L
Disease activity	Jenks (2010) [RCT][174]	Probiotic vs placebo at week 12<br>SMD -0.33 (-0.82, 0.17)	BASDAI, BL / week 12, mean (SD)<br>Probiotic: 4.2 (2.2) / 3.2 (2.1)<br>Placebo: 4.5 (2.0) / 3.9 (2.2)		L	L	L	L
Tender joints	Jenks (2010) [RCT][174]	Probiotic vs placebo at week 12<br>SMD -0.34 (-0.84, 0.16)	TJC, BL / week 12, mean (SD)<br>Probiotic: 2.0 (2.1) / 3.1 (3.9)<br>Placebo: 2.6 (2.6) / 5.4 (8.8)		L	L	L	L
Swollen joints	Jenks (2010) [RCT][174]	Probiotic vs placebo at week 12<br>SMD 0.07 (-0.43, 0.56)	SJC, BL / week 12, mean (SD)<br>Probiotic: 0.4 (0.9) / 0.25 (0.9)<br>Placebo: 0.5 (1.1) / 0.2 (0.5)		L	L	L	L
Spinal mobility	Jenks (2010) [RCT][174]	Probiotic vs placebo at week 12<br>SMD -0.04 (-0.53, 0.46)	BASMI, BL / week 12, mean (SD)<br>Probiotic: 2.7 (2.6) / 2.3 (2.3)<br>Placebo: 2.7 (3.0) / 2.4 (3.0)		L	L	L	L

Alloc. Conc. = allocation concealment, AMSTAR2 = Assessing the Methodological Quality of Systematic Reviews, AS = ankylosing spondylitis, ASQOL = Ankylosing Spondylitis Quality of Life, BASDAI = Bath Ankylosing Spondylitis Disease Activity Index, BASFI = Bath Ankylosing Spondylitis Function Index, BASMI = Bath Ankylosing Spondylitis Metrology Index, Blind. Asses. = Blinded assessors, Blind. Part. = blinded participants, CI = confidence interval, CRP = C-reactive protein, H/UC = high / unclear risk of bias, L = low risk of bias, MAF = Multidimensional Assessment of Fatigue, MASES = Maastricht Ankylosing Spondylitis Enthesitis Score, Rand. Seq. = random sequence generation, RCT = randomised controlled trial, SJC = swollen joint count, SMD = Standardised mean difference, TJC = tender joint count, VAS = visual analogue scale

Supplementary table 127 – Description of studies of animal products in PsA

Table – Animal products (PsA), description of included studies

Author (date) [country]	Study design	Inclusion criteria	Exposure detail	N	Age, mean (SD) years	N (%) female	Funders
Kristensen (2018) [Denmark][175]	RCT	CASPAR criteria for PsA, aged >18 years<br>Exclusions: Pregnancy, treatment with bDMARD or oral steroids	1) 3g of n-3 polyunsaturated fatty acids (50% EPA and 50% DHA) per day<br>p) olive oil placebo	1) 72<br>p) 71	1) 53.2 (11.4)<br>p) 50.7 (11.5)	1) 40 (55.6)<br>p) 43 (60.6)	**University** (Aalborg University Hospital Research Foundation), **Charity** (Medical Research Foundation of the Northern Denmark Region, Danish Rheumatism Association, Danish Psoriasis Foundation, Aage Bang Foundation, Abbvie Foundation, Heinrich Kopps Foundation, Jacob Madsen and wide Olga Madsen's Foundation)
Madland (2006) [Norway][176]	RCT	Polyarticular PsA (≥5 swollen joints in a patient with psoriasis and RF-)	1) Seal oil (containing polyunsaturated fatty acids – 2.4g EPA, 1.1g of DPA and 2.6g DHA)<br>p) Soy oil placebo	1) 20<br>p) 20	1) 56.9 (11.5)<br>p) 53.0 (10.6)	1) 10 (50)<br>p) 12 (60)	**Charity** (The Foundation of Astri and Edvard Riisøen)
Veale (1994) [UK][177]	RCT	RF-, had joint involvement in at least 1 joint	1) Efamol oil (combination of fish oil and primrose oil) (240mg EPA, 132mg DHA)<br>p) Liquid paraffin	1) 19<br>p) 19	Median (range)<br>1) 40 (18-76)<br>p) 40 (25-58)	1) 12 (63.2)<br>p) 12 (63.2)	**Industry** (Scotia Pharmaceuticals), Action Research

bDMARD = biologic disease modifying anti-rheumatic drugs, CASPAR = Classification Criteria for Psoriatic Arthritis, DHA = docosahexaenoic acid, DPA = docosapentaenoic acid, EPA = eicosapentaenoic acid, N = number, PsA = psoriatic arthritis, RCT = randomised controlled trial, RF = rheumatoid factor, SD = standard deviation, UK = United Kingdom

Supplementary table 128 – Marine animal oil / omega 3 and PsA progression, results

Table – Marine animal oil / omega 3 (PsA), results and quality assessment

Outcome	Study (date) [study type]	Standardised result, SMD (95% CI) unless otherwise stated	Natural result	AMSTAR2 quality	Rand. Seq.	Alloc. Conc.	Blind. Part.	Blind. Asses.
Pain	Kristensen (2018) [RCT][175]		Pain VAS, mean at week 24<br>Fish oil: 30.12<br>Control: 34.45, p=0.36		L	L	L	L
	Madland (2006) [RCT][176]	Seal oil vs control at 6 weeks<br>SMD 0.09 (-0.53, 0.71)	Pain VAS, BL / 6 weeks, mean (SD §)<br>Seal oil: 50.3 (23.3) / 38.3 (22.2)<br>Control: 41.5 (21.1) / 36.5 (18.5)		H/UC	H/UC	L	H/UC
Function	Kristensen (2018) [RCT][175]		HAQ, mean at week 24<br>Fish oil: 0.70<br>Control: 0.78, p=0.81		L	L	L	L
	Madland (2006) [RCT][176]	Seal oil vs control at 6 weeks<br>SMD 0.61 (-0.02, 1.25)	MHAQ, BL / 6 weeks, mean (SD §)<br>Seal oil: 1.73 (0.40) / 1.75 (0.48)<br>Control: 1.58 (0.35) / 1.50 (0.32)		H/UC	H/UC	L	H/UC
Disease activity	Kristensen (2018) [RCT][175]		DAS28-CRP, mean at week 24<br>Fish oil: 2.34<br>Control: 2.71, p=0.20		L	L	L	L
	Kristensen (2018) [RCT][175]		ASDAS, mean at week 24<br>Fish oil: 1.95<br>Control: 2.26, p=0.96		L	L	L	L
	Kristensen (2018) [RCT][175]		BASDAI, mean at week 24<br>Fish oil: 11.29<br>Control: 14.37, p=0.42		L	L	L	L
Tender joints	Kristensen (2018) [RCT][175]		TJC, mean at week 24<br>Fish oil: 2.67<br>Control: 4.10, p=0.08		L	L	L	L
	Madland (2006) [RCT][176]	Seal oil vs control at 6 weeks<br>SMD -0.03 (-0.65, 0.59)	TJC. BL / 6 weeks, mean (SD §)<br>Seal oil: 13.8 (9.9) / 9.8 (7.8)<br>Control: 13.0 (7.2) / 10.0 (7.0)		H/UC	H/UC	L	H/UC
	Veale (1994) [RCT][177]	Efamol oils vs control at 12 months<br>SMD 0.29 (-0.35, 0.93)	Ritchie Index at 12 months, mean (SD §)<br>Efamol oil: 12 (7.04)<br>Control: 10.25 (4.61)		H/UC	H/UC	L	H/UC
	Bespoke meta-analysis	Marine oils vs control<br>SMD 0.13 (-0.32, 0.57) I^2 0.0%						

§ Mean (SD) estimated from median (range) using published formula[61]

Alloc. Conc. = allocation concealment, AMSTAR2 = Assessing the Methodological Quality of Systematic Reviews, ASDAS = Ankylosing Spondylitis Disease Activity Score, BASDAI = Bath Ankylosing Spondylitis Disease Activity Index, Blind. Asses. = Blinded assessors, Blind. Part. = blinded participants, CI = confidence interval, CRP = C-reactive protein, DAS28 = Disease Activity Score, ESR = erythrocyte sedimentation rate, H/UC = high / unclear risk of bias, HAQ = Health Assessment Questionnaire, L = low risk of bias, LEI = Leeds Enthesitis Index, PASI = Psoriasis Area and Severity Index, PsA = psoriatic arthritis, Rand. Seq. = random sequence generation, RCT = randomised controlled trial, SJC = swollen joint count, SMD = Standardised mean difference, SPARCC = Spondyloarthritis Research Consortium of Canada Enthesitis Index, TJC = tender joint count, VAS = visual analogue scale

Table – Marine animal oil / omega 3 (PsA), results and quality assessment

Outcome	Study (date) [study type]	Standardised result, SMD (95% CI) unless otherwise stated	Natural result	AMSTAR2 quality	Rand. Seq.	Alloc. Conc.	Blind. Part.	Blind. Asses.
Swollen joints	Kristensen (2018) [RCT][175]		SJC, mean at week 24<br>Fish oil: 0.30<br>Control: 0.84, p=0.41		L	L	L	L
	Madland (2006) [RCT][176]	Seal oil vs control at 6 weeks<br>SMD -0.67 (-1.31, -0.03)	SJC. BL / 6 weeks, mean (SD §)<br>Seal oil: 3.8 (2.9) / 2.3 (1.6)<br>Control: 3.5 (2.7) / 4.0 (3.2)		H/UC	H/UC	L	H/UC
Enthesitis	Kristensen (2018) [RCT][175]		LEI, mean at week 24<br>Fish oil: 0.83<br>Control: 0.84, p=0.94		L	L	L	L
	Kristensen (2018) [RCT][175]		SPARCC, mean at week 24<br>Fish oil: 1.85<br>Control: 1.94, p=0.89		L	L	L	L
Psoriasis severity	Kristensen (2018) [RCT][175]		PASI, mean at week 24<br>Fish oil: 1.61<br>Control: 2.04, p=0.47		L	L	L	L
Patient global	Madland (2006) [RCT][176]	Seal oil vs control at 6 weeks<br>SMD 0.09 (-0.53, 0.71)	Patient global VAS. BL / 6 weeks, mean (SD §)<br>Seal oil: 50.3 (23.3) / 38.3 (22.2)<br>Control: 41.5 (21.1) / 36.5 (18.5)		H/UC	H/UC	L	H/UC
CRP	Veale (1994) [RCT][177]		"No significant difference"		H/UC	H/UC	L	H/UC
ESR	Madland (2006) [RCT][176]	Seal oil vs control at 6 weeks<br>SMD -0.30 (-0.92, 0.32)	ESR. BL / 6 weeks, mean (SD §)<br>Seal oil: 14.3 (10.2) / 15.8 (11.5)<br>Control: 13.3 (8.3) / 20.0 (16.1)		H/UC	H/UC	L	H/UC
	Veale (1994) [RCT][177]		"No significant difference"		H/UC	H/UC	L	H/UC

§ Mean (SD) estimated from median (range) using published formula[61]

Alloc. Conc. = allocation concealment, AMSTAR2 = Assessing the Methodological Quality of Systematic Reviews, ASDAS = Ankylosing Spondylitis Disease Activity Score, BASDAI = Bath Ankylosing Spondylitis Disease Activity Index, Blind. Asses. = Blinded assessors, Blind. Part. = blinded participants, CI = confidence interval, CRP = C-reactive protein, DAS28 = Disease Activity Score, ESR = erythrocyte sedimentation rate, H/UC = high / unclear risk of bias, HAQ = Health Assessment Questionnaire, L = low risk of bias, LEI = Leeds Enthesitis Index, PASI = Psoriasis Area and Severity Index, PsA = psoriatic arthritis, Rand. Seq. = random sequence generation, RCT = randomised controlled trial, SJC = swollen joint count, SMD = Standardised mean difference, SPARCC = Spondyloarthritis Research Consortium of Canada Enthesitis Index, TJC = tender joint count, VAS = visual analogue scale

Supplementary table 129 – Description of studies of minerals and supplements in PsA

Table – Minerals and supplements (PsA), description of included studies

Author (date) [country]	Study design	Inclusion criteria	Exposure detail	N	Age, mean (SD) years	N (%) female	Funders
Kharaeva (2009) [Russia][178]	RCT	Joint involvement, history of psoriasis, radiographic presentation of polyarthritis	1) Selenium, co-enzyme Q10 and vitamin E<br>p) Soy based placebo	1) 15<br>p) 15	1) 43.1 (7.6)<br>p) 44.0 (6.9)	1) 7 (46.7)<br>p) 9 (60.0)	**Government** (Italian Ministry for Health)

N = number, PsA = psoriatic arthritis, RCT = randomised controlled trial, SD = standard deviation

Supplementary table 130 – Selenium / co-enzyme Q10 / vitamin E and PsA progression, results

Table – Selenium / coenzyme Q10 / vitamin E (PsA), results and quality assessment

Outcome	Study (date) [study type]	Standardised result, SMD (95% CI) unless otherwise stated	Natural result	AMSTAR2 quality	Rand. Seq.	Alloc. Conc.	Blind. Part.	Blind. Asses.
Disease severity	Kharaeva (2009) [RCT][178]	Selenium / coenzyme Q10 / vitamin E vs control at 30 days<br>SMD -8.03 (-10.25, -5.81)	Severity score §, day 30, mean (SD †)<br>Selenium / coenzyme Q10 / vitamin E: 1.9 (0.39)<br>Control: 6.8 (0.77)		H/UC	H/UC	H/UC	H/UC
Psoriasis severity	Kharaeva (2009) [RCT][178]	Selenium / coenzyme Q10 / vitamin E vs control at 30 days<br>SMD -0.09 (-0.81, 0.62)	PASI, day 30, mean (SD †)<br>Selenium / coenzyme Q10 / vitamin E: 16 (23.2)<br>Control: 29 (38.7)		H/UC	H/UC	H/UC	H/UC

§ composite score of 4 point scales assessing desquamation of psoriatic plaques, hyperemia of psoriatic plaques, inflammation of psoriatic plaques, nail dystrophy and pain in joints
† calculated from standard error
Alloc. Conc. = allocation concealment, AMSTAR2 = Assessing the Methodological Quality of Systematic Reviews, Blind. Asses. = Blinded assessors, Blind. Part. = blinded participants, CI = confidence interval, H/UC = high / unclear risk of bias, L = low risk of bias, PASI = Psoriasis Area and Severity Index, PsA = psoriatic arthritis, Rand. Seq. = random sequence generation, SMD = Standardised mean difference,

Supplementary table 131 – Description of studies of experimental diets in SSc

Table – Experimental diets (SSc), description of included studies

Author (date) [country]	Study design	Inclusion criteria	Exposure detail	N	Age, mean (SD) years	N (%) female	Funders
Doerfler (2017) [USA][179]	Single arm int.	Aged >18 years, referred from rheumatologist to a university affiliated gastroenterologist practice for GI symptoms and unintentional weight-loss Exclusions: Pregnant, deemed too ill to participate, unwilling to travel to study	1) individualised plan based on several themes (calorie and protein intake, modified textures, lifestyle modifications) intended to prevent further weight loss and address a spectrum of motility issues (e.g. gastroparesis, diarrhoea, dysphagia) and fatigue management	18	51.3 (11)	16 (88.9)	**Government** (National Cancer Institute, Cancer Education and Career Development Program), **Charity** (American Dietetic Association Foundation)
Ortiz-Santamaria (2014) [Spain][180]	Single arm int.	Aged ≥18 years, LeRoy and Medsger criteria for SSc, read Catalan/Castilian, ≥1 on MUST screening Exclusions: neoplastic process, other conditions that interfere with the nutritional status of the patient, mental or cognitive psychiatric impairment	Supplements for deficiencies (iron, vitamin D), met dietician to discuss diet, encouraged to eat healthily	9	62.6 (11.7)	8 (88.9)	Not reported – authors declare no conflicts of interest

int. = intervention, MUST = Malnutrition Universal Screening Tool, N = number, SD = standard deviation, SSc = systemic sclerosis, USA = United States of America

Supplementary table 132 – Medical nutrition therapy and SSc progression, results

Table – Medical nutrition therapy (SSc), results and quality assessment

Outcome	Study (date) [study type]	Standardised result, SMD (95% CI) unless otherwise stated	Natural result	AMSTAR2 quality	Rand. Seq.	Alloc. Conc.	Blind. Part.	Blind. Asses.
Patient global	Doerfler (2017) [Single arm int.][179]		Abridged patient-generated subjective global assessment, BL / 6 weeks, mean (SD)<br>13.1 (7.2) / 7.6 (5.2), p=0.01					
Quality of life	Doerfler (2017) [Single arm int.][179]		HRQoL, BL / 6 weeks, mean (SD)<br>7.7 (6.6) / 6.6 (6.5), p=0.34					

Alloc. Conc. = allocation concealment, AMSTAR2 = Assessing the Methodological Quality of Systematic Reviews, BL = baseline, Blind. Asses. = Blinded assessors, Blind. Part. = blinded participants, CI = confidence interval, H/UC = high / unclear risk of bias, HRQoL = Health related quality of life, L = low risk of bias, Rand. Seq. = random sequence generation, SD = standard deviation, SMD = Standardised mean difference, SSc = systemic sclerosis

Table – Medical nutrition therapy (SSc), SF36 results at BL / final follow-up

Author (date)	PCS	MCS	GH	PF	RP	RE	SF	BP	V	MH
Ortiz-Santamaria (2014)[180]	32.6 (6.9) / 38.3 (2.1)	38.4 (14.4) / 35.33 (18.4)	31.7 (8.4) / 33.0 (3.6)	31.6 (8.03) / 44.0 (6.6)	37.0 (11.2) / 30.3 (3.21)	35.8 (51.6) / 31.0 (12.1)	41.0 (15.1) / 32.0 (8.2)	37.6 (7.2) / 29.7 (5.77)	37.7 (10.5) / 29.7 (5.8)	33.1 (12.8) / 20.0 (3.6)

BL = baseline, BP = bodily pain, GH = general health, MCS = mental component score, MH = mental health, PCS = physical component score, PF = physical function, RE = role emotional, RP = role physical, SF = social functioning, SSc = systemic sclerosis, V = vitality

Supplementary table 133 – Description of studies of vitamins in SSc

Table – Vitamins (SSc), description of included studies

Author (date) [country]	Study design	Inclusion criteria	Exposure detail	N	Age, mean (SD) years	N (%) female	Funders
Ostojic (2011) [Serbia][181]	RCT	Early diffuse SSc, symptom duration <15 months, positive antibodies against topoisomerase, high skin thickness progression rate (≥12/year), decreased lung diffusing capacity (≤75%)	1) cyclophosphamide, and antioxidants (alpha-tocopherol [vitamin E] 400 IU / day and ascorbic acid [vitamin C] 1000 mg per day)<br>p) cyclophosphamide only<br>[All patients treated with prednisolone, metoclopramide, ranitidine and nifedipine	1) 6<br>p) 7	1) 51.3 (10.1)<br>p) 46.6 (9.1)	1) 4 (66.7)<br>p) 4 (57.1)	None declared – authors reported no conflicts of interest
Herrick (2000) [UK][182]	RCT§	Limited cutaneous SSc, 26 patients met the ARA SSc classification, other 7 all suffered Raynauds and were considered SSc on the following basis: (a) Sclerodactyly and abnormal nailfold microscopy (4 patients, 2 of whom were positive for anticentromere antibody); (b) Calcinosis, abnormal nail-fold microscopy and positive anticentromere antibody (1 patient); (c) Sclerodactyly and reduced peristalsis on barium swallow (1 patient); (d) Digital pitting, abnormal nail-fold microscopy and positive anticentromere antibody (1 patient).<br>Exclusions: cigarette smokers, vitamin supplementation <10 weeks before study entry	1) 300mg selenium, 28.8mg beta-carotene, 188mg vitamin E, approx. 600mg vitamin C, approx. 1.6g methionine<br>p) Matching placebo tablets	33	47<br>(range: 25-68)	30 (90.9)	**Charity** (Raynaud's and Sclerosis Association)
Hulshof (2000) [The Netherlands][183]	RCT	Morphea or SSc according to criteria<br>Exclusion: use of any systemic or topical therapy for SSc <1 month prior to start of study, use of medication likely to interfere with safety of treatment, clinically relevant abnormalities, serological evidence of Borrelia Burgdorferi	1) Vitamin D – 0.75 μg/day calcitriol for 6 months and 1.25 μg/day for an additional 3 months<br>p) Placebo	1) 10<br>p) 10	1) 41.8 (19.1)<br>p) 55.5 (14.6)	1) 10 (100)<br>p) 9 (90.0)	Not reported

§ Crossover design
N = number, RCT = randomised controlled trial, SD = standard deviation, SSc = systemic sclerosis, UK = United Kingdom

Supplementary table 134 – Antioxidants and SSc progression, results

Table – Antioxidants (SSc), results and quality assessment

Outcome	Study (date) [study type]	Standardised result, SMD (95% CI) unless otherwise stated	Natural result	AMSTAR2 quality	Rand. Seq.	Alloc. Conc.	Blind. Part.	Blind. Asses.
Skin score	Ostojic (2011) [RCT][181]	Antioxidants vs control at 1 month<br>SMD -1.07 (-2.25, 0.11)	Modified Rodnan Skin Score, BL / 1 month, mean (SD)<br>Antioxidants: 15.7 (6.0) / 16.4 (4.1)<br>Control: 17.9 (6.7) / 23.6 (8.3)		H/UC	H/UC	H/UC	H/UC
Raynaud's	Herrick (2000) [RCT][182]	Antioxidants vs control at 10 weeks<br>SMD 0.05 (-0.44, 0.53)	Raynaud's attacks by 10 weeks, mean (SD §)<br>Antioxidants: 143.7 (70.5)<br>Control: 139 (130.19); p=0.88		H/UC	H/UC	L	L

§ Estimated from median (IQR) using published equation[61]

Alloc. Conc. = allocation concealment, AMSTAR2 = Assessing the Methodological Quality of Systematic Reviews, Blind. Asses. = Blinded assessors, Blind. Part. = blinded participants, CI = confidence interval, H/UC = high / unclear risk of bias, L = low risk of bias, Rand. Seq. = random sequence generation, SMD = Standardised mean difference, SSc = systemic sclerosis

Supplementary table 135 – Vitamin D and SSc progression, results

Table – Vitamin D (SSc), results and quality assessment

Outcome	Study (date) [study type]	Standardised result, SMD (95% CI) unless otherwise stated	Natural result	AMSTAR2 quality	Rand. Seq.	Alloc. Conc.	Blind. Part.	Blind. Asses.
Skin score	Hulshof (2000) [RCT][183]	Vitamin D vs placebo at 9 months<br>SMD -2.50 (-4.65, -0.35)	Rodnan skin score, mean (SD) † at 9 months<br>Vitamin D: 3.66 (5.51)<br>Control: 21.75 (8.18)		H/UC	L	L	H/UC

† Calculated from results in paper to exclude patients with morphea (N=7)

Alloc. Conc. = allocation concealment, AMSTAR2 = Assessing the Methodological Quality of Systematic Reviews, Blind. Asses. = Blinded assessors, Blind. Part. = blinded participants, CI = confidence interval, H/UC = high / unclear risk of bias, L = low risk of bias, MA = meta-analysis, Rand. Seq. = random sequence generation, SMD = Standardised mean difference, SSc = systemic sclerosis

Supplementary table 136 – Description of reviews of animal products in gout

Table – Animal products (gout), description of reviews

Authors (date)	Review type	Study type included	Exposure detail	Number of studies included	Funders
Andres (2014)[184]	SR	RCTs	Enriched skimmed milk powder	1	**Hospital** (Hospital General Universitario de Alicante, Hospital General Universitario de Elda, Cabrini Hospital), **University** (Columbia University Medical Center, Monash University, Universidad Camilo José Cela)
Moi et al (2013)[185]	SR	RCTs	Enriched skimmed milk powder	1	**Hospital** (The Royal Melbourne Hospital, Cabrini Hospital, Southampton General Hospital), **University** (Monash University)

MA = meta-analysis, RCT = randomised controlled trial, SR = systematic review

Supplementary table 137 – Enriched milk powder and gout progression, results

Table – Enriched milk powder (gout), results and quality assessment

Outcome	Study (date) [study type]	Standardised result, SMD (95% CI) unless otherwise stated	Natural result	AMSTAR2 quality	Rand. Seq.	Alloc. Conc.	Blind. Part.	Blind. Asses.
Pain	Andres (2014) [SR][184] & Moi (2013)[185]		Pain, mean difference between intervention and placebo<br>-1.03 (-1.89, -0.17)	High				
Function	Andres (2014) [SR][184] & Moi (2013)[185]		Function, mean difference between intervention and placebo<br>-0.03 (-0.14, 0.08)	High				
Uric acid	Andres (2014) [SR][184] & Moi (2013)[185]		Serum uric acid, mean difference between intervention and placebo<br>-0.01 (-0.04, 0.01)	High				
Gout flare	Andres (2014) [SR][184] & Moi (2013)[185]		Gout flare, mean difference between intervention and placebo<br>-0.21 (-0.76, 0.34)	High				

Alloc. Conc. = allocation concealment, AMSTAR2 = Assessing the Methodological Quality of Systematic Reviews, Blind. Asses. = Blinded assessors, Blind. Part. = blinded participants, CI = confidence interval, H/UC = high / unclear risk of bias, L = low risk of bias, Rand. Seq. = random sequence generation, SMD = Standardised mean difference

Supplementary table 138 – Description of studies of fruits, vegetables and other plant based intervention studies in gout

Table – Fruits, vegetables and other plant based intervention (gout), description of included studies

Author (date) [country]	Study design	Inclusion criteria	Exposure detail	N	Age, mean (SD) years	N (%) female	Funders
Yu (2018) [China][186]	RCT	Physician diagnosed gout, hyperuricemia (serum uric acid >420 micromol/L) aged 18-70 years, “dampness heat pouring downward pattern” (Chinese medicine)<br>Exclusions: Pregnancy or lactation, allergic constitution, serum creatine >1.5mg/dL, ALT>2x upper limit of normal, severe deformity of stiffness of gouty arthropathy resulting in disability, arrhythmia of clinical significance, history of alcohol abuse, severe cerebrovascular, kidney, liver or hematopoietic system comorbidities, cancer, mental disorders, taking hypouricemic medications, azathioprine, 6-mercaptopurine, medications containing aspirin (>325mg) or salicylate, or had participated in other clinical trials with last 3 months	1) “Yellow-Dragon Wonderful Seed Formula” containing Earthworm, cardamon, Phellodendron bark, Atractylodes, sword-like attractylodes rhizome, Chinese atractylodes rhizome, Coix seeds, Job’s tears, Cyathula, medicinal cyathula root<br>2) Same as 1) + gypsum<br>p) allopurinol	1) 24<br>2) 24<br>p) 24	1) 45.3 (9.9)<br>2) 46.1 (10.8)<br>p) 49.2 (9.5	1) 0 (0)<br>2) 0 (0)<br>p) 0 (0)	**Government** (National TCM Clinical Research Base for Diabetes Mellitus)

N = number, SD = standard deviation, TCM = traditional Chinese medicine, USA = United States of America

Supplementary table 139 – Herbal medicine and gout progression, results

Table – Herbal medicine (gout), results and quality assessment

Outcome	Study (date) [study type]	Standardised result, SMD (95% CI) unless otherwise stated	Natural result	AMSTAR2 quality	Rand. Seq.	Alloc. Conc.	Blind. Part.	Blind. Asses.
Uric acid	Yu (2018) [RCT][186]	Yellow-dragon Wonderful seed vs allopurinol at 4 weeks<br>SMD 0.30 (-0.27, 0.87)<br>Yellow-dragon Wonderful seed + gypsum vs allopurinol at 4 weeks<br>SMD 0.48 (-0.09, 1.06)	Serum uric acid, BL / 4 weeks, mean (SD)<br>Yellow-dragon Wonderful seed: 562.29 (108.30) / 526.29 (156.15)<br>Yellow-dragon Wonderful seed + gypsum: 585.46 (100.06) / 566.29 (206.08)<br>Allopurinol: 618.00 (114.27) / 480.83 (144.34)		L	L	H/UC	H/UC
CRP	Yu (2018) [RCT][186]	Yellow-dragon Wonderful seed vs allopurinol at 4 weeks<br>SMD -0.18 (-0.74, 0.39)<br>Yellow-dragon Wonderful seed + gypsum vs allopurinol at 4 weeks<br>SMD 0.11 (-0.46, 0.68)	Serum uric acid, BL / 4 weeks, mean (SD)<br>Yellow-dragon Wonderful seed: 13.13 (2.63) / 10.33 (4.34)<br>Yellow-dragon Wonderful seed + gypsum: 14.03 (3.40) / 11.64 (4.62)<br>Allopurinol: 13.15 (1.13) / 11.13 (4.77)		L	L	H/UC	H/UC

Alloc. Conc. = allocation concealment, AMSTAR2 = Assessing the Methodological Quality of Systematic Reviews, BL = baseline, Blind. Asses. = Blinded assessors, Blind. Part. = blinded participants, CI = confidence interval, CRP = C-reactive protein, H/UC = high / unclear risk of bias, L = low risk of bias, Rand. Seq. = random sequence generation, SD = standard deviation, SMD = Standardised mean difference

Supplementary table 140 – Description of studies of vitamins in gout

Table – Vitamins (Gout), description of included studies

Author (date) [country]	Study design	Inclusion criteria	Exposure detail	N	Age, mean (SD) years	N (%) female	Funders
Stamp (2013) [New Zealand][187]	RCT	ACR gout criteria, serum uric acid >0.36 mmoles/litre<br>Exclusions: taking over the counter vitamin supplements	1) Vitamin C, 500mg/day<br>p) Allopurinol	1) 20<br>p) 20	1) 61.2 (range 39-86)<br>p) 55 (range 27-78)	1) 18 (90.0)<br>p) 18 (90.0)	**Government** (Health Research Council of New Zealand)
Azzeh (2017) [Saudi Arabia][188]	Single arm int.	Exclusions: aged <20 years, history of dialysis, alcohol consumption, pregnant/lactating women, mutli-vitamin supplements during last 3 months, diuretic drug and/or any uricosuric agent (e.g. allopurinol)	1) Vitamin C, 500mg/day	15	52.9 (11.4)	6 (40.0)	Not reported – authors declare no conflict of interest

int. = intervention, N = number, SD = standard deviation

Supplementary table 141 – Vitamin C and gout progression, results

Table – Vitamin C (gout), results and quality assessment

Outcome	Study (date) [study type]	Standardised result, SMD (95% CI) unless otherwise stated	Natural result	AMSTAR2 quality	Rand. Seq.	Alloc. Conc.	Blind. Part.	Blind. Asses.
Uric acid	Stamp (2013) [RCT][187]	Vitamin C vs Allopurinol, mean change BL-8 weeks<br>SMD 0.12 (-0.50, 0.74)	Serum uric acid, mean (SD) change BL-8 weeks<br>Vitamin C: -0.014 (0.23)<br>Allopurinol: -0.188 (1.98); p<0.001		H/UC	H/UC	H/UC	H/UC
	Azzeh (2017) [single arm int.][188]		Serum uric acid, BL / 8 weeks, mean (SD)<br>8.09 (1.09) / 8.4 (1.15)					

Alloc. Conc. = allocation concealment, AMSTAR2 = Assessing the Methodological Quality of Systematic Reviews, BL = baseline, Blind. Asses. = Blinded assessors, Blind. Part. = blinded participants, CI = confidence interval, H/UC = high / unclear risk of bias, L = low risk of bias, Rand. Seq. = random sequence generation, SD = standard deviation, SMD = Standardised mean difference

Supplementary table 142 – Description of studies including more than one RMD

Table –Studies of more than one RMD, description of included studies

Author (date) [country]	Study design	Inclusion criteria	Exposure detail	N	Age, mean (SD) years	N (%) female	Funders
Jantti (1985) [Finland][189]	NRT	RA or spondyloarthritis, 1 reactive arthritis, two months prior to trial patients didn't receive DMARDs, 2 weeks before – no NSAIDs / paracetamol allowed.	1) Linoleic acid<br>p) Olive oil	1) 6<br>p) 4	Not reported	Not reported	**Government** (Academy of Finland), **Charity** (Yrjo Jahnsson Foundation)
Bradley (1990) [USA][190]	Single arm int.	Obese patients	Powdered meal replacement consumed twice daily with one regular meal	30	64.6	25 (83.3)	Not reported

int. = intervention, OA = osteoarthritis, N = number, RA = rheumatoid arthritis, RMD = rheumatic and musculoskeletal disease, SD = standard deviation, USA = United States of America

Supplementary table 143 – Results of studies including more than one RMD

Table – Elemental diet (RA and OA), results and quality assessment

Outcome	Study (date) [study type]	Standardised result, SMD (95% CI) unless otherwise stated	Natural result	AMSTAR2 quality	Rand. Seq.	Alloc. Conc.	Blind. Part.	Blind. Asses.
Function	Bradley (1990) [single arm int.][190]		50ft walk test (seconds), BL / 6 weeks, mean<br>12.0 / 9.7					

Alloc. Conc. = allocation concealment, AMSTAR2 = Assessing the Methodological Quality of Systematic Reviews, Blind. Asses. = Blinded assessors, Blind. Part. = blinded participants, CI = confidence interval, H/UC = high / unclear risk of bias, int. = intervention, L = low risk of bias, OA = osteoarthritis, RA = rheumatoid arthritis, Rand. Seq. = random sequence generation, SMD = Standardised mean difference

Table – Linoleic acid (RA and AS), results and quality assessment

Outcome	Study (date) [study type]	Standardised result, SMD (95% CI) unless otherwise stated	Natural result	AMSTAR2 quality	Rand. Seq.	Alloc. Conc.	Blind. Part.	Blind. Asses.
Tender joints	Jantti (1985) [NRT][189]		Tender joint count, change from BL-21 days, mean (range)<br>Linoleic acid: 3 (-1, 15)<br>Placebo: 2 (-1, 5)					
Swollen joints	Jantti (1985) [NRT][189]		Swollen joint count, change from BL-21 days, mean (range)<br>Linoleic acid: 1 (0, 2)<br>Placebo: 0 (-1, 1)					
Morning stiffness	Jantti (1985) [NRT][189]		Morning stiffness, change from BL-21 days, mean (range)<br>Linoleic acid: 10 (-60, 90)<br>Placebo: -6 (-20, 0)					
Grip strength	Jantti (1985) [NRT][189]		Grip strength (left), change from BL-21 days, mean (range)<br>Linoleic acid: -0.1 (-0.2, 0.1)<br>Placebo: -0.1 (-0.2, 0.2)<br>Grip strength (right), change from BL-21 days, mean (range)<br>Linoleic acid: 0 (-0.2, 0.2)<br>Placebo: 0 (-0.1, 0.1)					
ESR	Jantti (1985) [NRT][189]		ESR, change from BL-21 days, mean (range)<br>Linoleic acid: -1 (-10, 7)<br>Placebo: -2 (-12, 8)					

Alloc. Conc. = allocation concealment, AMSTAR2 = Assessing the Methodological Quality of Systematic Reviews, BL = baseline, Blind. Asses. = Blinded assessors, Blind. Part. = blinded participants, CI = confidence interval, ESR = erythrocyte sedimentation rate, H/UC = high / unclear risk of bias, L = low risk of bias, NRT = nonrandomised trial, Rand. Seq. = random sequence generation, SMD = Standardised mean difference

Reference List

(1) Mease PJ. Measures of psoriatic arthritis: Tender and Swollen Joint Assessment, Psoriasis Area and Severity Index (PASI), Nail Psoriasis Severity Index (NAPSI), Modified Nail Psoriasis Severity Index (mNAPSI), Mander/Newcastle Enthesitis Index (MEI), Leeds Enthesitis Index (LEI), Spondyloarthritis Research Consortium of Canada (SPARCC), Maastricht Ankylosing Spondylitis Enthesis Score (MASES), Leeds Dactylitis Index (LDI), Patient Global for Psoriatic Arthritis, Dermatology Life Quality Index (DLQI), Psoriatic Arthritis Quality of Life (PsAQOL), Functional Assessment of Chronic Illness Therapy-Fatigue (FACIT-F), Psoriatic Arthritis Response Criteria (PsARC), Psoriatic Arthritis Joint Activity Index (PsAJAI), Disease Activity in Psoriatic Arthritis (DAPSA), and Composite Psoriatic Disease Activity Index (CPDAI). *Arthritis Care Res (Hoboken)* 2011; 63 Suppl 11:S64-S85.

(2) Zochling J. Measures of symptoms and disease status in ankylosing spondylitis: Ankylosing Spondylitis Disease Activity Score (ASDAS), Ankylosing Spondylitis Quality of Life Scale (ASQoL), Bath Ankylosing Spondylitis Disease Activity Index (BASDAI), Bath Ankylosing Spondylitis Functional Index (BASFI), Bath Ankylosing Spondylitis Global Score (BAS-G), Bath Ankylosing Spondylitis Metrology Index (BASMI), Dougados Functional Index (DFI), and Health Assessment Questionnaire for the Spondylarthropathies (HAQ-S). *Arthritis Care Res (Hoboken)* 2011; 63 Suppl 11:S47-S58.

(3) Romero-Diaz J, Isenberg D, Ramsey-Goldman R. Measures of adult systemic lupus erythematosus: updated version of British Isles Lupus Assessment Group (BILAG 2004), European Consensus Lupus Activity Measurements (ECLAM), Systemic Lupus Activity Measure, Revised (SLAM-R), Systemic Lupus Activity Questionnaire for Population Studies (SLAQ), Systemic Lupus Erythematosus Disease Activity Index 2000 (SLEDAI-2K), and Systemic Lupus International Collaborating Clinics/American College of Rheumatology Damage Index (SDI). *Arthritis Care Res (Hoboken)* 2011; 63 Suppl 11:S37-S46.

(4) Khanna D, Lovell DJ, Giannini E, Clements PJ, Merkel PA, Seibold JR et al. Development of a provisional core set of response measures for clinical trials of systemic sclerosis. *Ann Rheum Dis* 2008; 67(5):703-709.

(5) Taylor WJ, Schumacher HR, Jr., Singh JA, Grainger R, Dalbeth N. Assessment of outcome in clinical trials of gout--a review of current measures. *Rheumatology (Oxford)* 2007; 46(12):1751-1756.

(6) Dalbeth N, McQueen FM, Singh JA, MacDonald PA, Edwards NL, Schumacher HR, Jr. et al. Tophus measurement as an outcome measure for clinical trials of chronic gout: progress and research priorities. *J Rheumatol* 2011; 38(7):1458-1461.

(7) Andrews JS, Trupin L, Schmajuk G, Barton J, Margaretten M, Yazdany J et al. Muscle strength, muscle mass, and physical disability in women with systemic lupus erythematosus. *Arthritis Care Res (Hoboken)* 2015; 67(1):120-127.

(8) Alvarez-Hernandez E, Pelaez-Ballestas I, Vazquez-Mellado J, Teran-Estrada L, Bernard-Medina AG, Espinoza J et al. Validation of the Health Assessment Questionnaire disability index in patients with gout. *Arthritis Rheum* 2008; 59(5):665-669.

(9) OARSI. Outcome Measures [https://www.oarsi.org/research/outcome-measures]. 2013. 14-5-2018.
Ref Type: Online Source

(10) Singh JA, Taylor WJ, Simon LS, Khanna PP, Stamp LK, McQueen FM et al. Patient-reported outcomes in chronic gout: a report from OMERACT 10. *J Rheumatol* 2011; 38(7):1452-1457.

(11) Whalley D, McKenna SP, de JZ, van der Heijde D. Quality of life in rheumatoid arthritis. *Br J Rheumatol* 1997; 36(8):884-888.

(12) Doward LC, Spoorenberg A, Cook SA, Whalley D, Helliwell PS, Kay LJ et al. Development of the ASQoL: a quality of life instrument specific to ankylosing spondylitis. *Ann Rheum Dis* 2003; 62(1):20-26.

(13) McKenna SP, Doward LC, Whalley D, Tennant A, Emery P, Veale DJ. Development of the PsAQoL: a quality of life instrument specific to psoriatic arthritis. *Ann Rheum Dis* 2004; 63(2):162-169.

(14) Liu X, Machado GC, Eyles JP, Ravi V, Hunter DJ. Dietary supplements for treating osteoarthritis: a systematic review and meta-analysis. *Br J Sports Med* 2018; 52(3):167-175.

(15) Senftleber NK, Nielsen SM, Andersen JR, Bliddal H, Tarp S, Lauritzen L et al. Marine Oil Supplements for Arthritis Pain: A Systematic Review and Meta-Analysis of Randomized Trials. *Nutrients* 2017; 9(1).

(16) Azidah A, Arifah A, Roslida A, Jais A, Omar J, Sadagatullah A et al. A randomized, double-blind study comparing multiple doses of Channa striatus supplementation for knee osteoarthritis. *Orient Pharm Exp Med* 2017; 17:345-354.

(17) Hill CL, March LM, Aitken D, Lester SE, Battersby R, Hynes K et al. Fish oil in knee osteoarthritis: a randomised clinical trial of low dose versus high dose. *Ann Rheum Dis* 2016; 75(1):23-29.

(18) Chen JS, Hill CL, Lester S, Ruediger CD, Battersby R, Jones G et al. Supplementation with omega-3 fish oil has no effect on bone mineral density in adults with knee osteoarthritis: a 2-year randomized controlled trial. *Osteoporos Int* 2016; 27(5):1897-1905.

(19) Kumar S, Sugihara F, Suzuki K, Inoue N, Venkateswarathirukumara S. A double-blind, placebo-controlled, randomised, clinical study on the effectiveness of collagen peptide on osteoarthritis. *J Sci Food Agric* 2015; 95(4):702-707.

(20) Schauss AG, Stenehjem J, Park J, Endres JR, Clewell A. Effect of the novel low molecular weight hydrolyzed chicken sternal cartilage extract, BioCell Collagen, on improving osteoarthritis-related symptoms: a randomized, double-blind, placebo-controlled trial. *J Agric Food Chem* 2012; 60(16):4096-4101.

(21) Nagaoka I, Nabeshima K, Murakami S, Yamamoto T, Watanabe K, Tomonaga A et al. Evaluation of the effects of a supplementary diet containing chicken comb extract on symptoms and cartilage metabolism in patients with knee osteoarthritis. *Exp Ther Med* 2010; 1(5):817-827.

(22) Ruff KJ, Winkler A, Jackson RW, DeVore DP, Ritz BW. Eggshell membrane in the treatment of pain and stiffness from osteoarthritis of the knee: a randomized, multicenter, double-blind, placebo-controlled clinical study. *Clin Rheumatol* 2009; 28(8):907-914.

(23) Kalman DS, Heimer M, Valdeon A, Schwartz H, Sheldon E. Effect of a natural extract of chicken combs with a high content of hyaluronic acid (Hyal-Joint) on pain relief and quality of life in subjects with knee osteoarthritis: a pilot randomized double-blind placebo-controlled trial. *Nutr J* 2008; 7:3.

(24) Hesslink R, Jr., Armstrong D, III, Nagendran MV, Sreevatsan S, Barathur R. Cetylated fatty acids improve knee function in patients with osteoarthritis. *J Rheumatol* 2002; 29(8):1708-1712.

(25) Stammers T, Sibbald B, Freeling P. Efficacy of cod liver oil as an adjunct to non-steroidal anti-inflammatory drug treatment in the management of osteoarthritis in general practice. *Ann Rheum Dis* 1992; 51(1):128-129.

(26) Kilinc BE, Oc Y, Alibakan G, Bilgin E, Kanar M, Eren OT. An Observational 1-Month Trial on the Efficacy and Safety of Promerim for Improving Knee Joint. *Clin Med Insights Arthritis Musculoskelet Disord* 2018; 11:1179544118757496.

(27) Lu B, Driban JB, Duryea J, McAlindon T, Lapane KL, Eaton CB. Milk consumption and progression of medial tibiofemoral knee osteoarthritis: data from the Osteoarthritis Initiative. *Arthritis Care Res (Hoboken)* 2014; 66(6):802-809.

(28) Alrushud AS, Rushton AB, Kanavaki AM, Greig CA. Effect of physical activity and dietary restriction interventions on weight loss and the musculoskeletal function of overweight and obese older adults with knee osteoarthritis: a systematic review and mixed method data synthesis. *BMJ Open* 2017; 7(6):e014537.

(29) Dyer J, Davison G, Marcora SM, Mauger AR. Effect of a Mediterranean Type Diet on Inflammatory and Cartilage Degradation Biomarkers in Patients with Osteoarthritis. *J Nutr Health Aging* 2017; 21(5):562-566.

(30) Clinton CM, O'Brien S, Law J, Renier CM, Wendt MR. Whole-foods, plant-based diet alleviates the symptoms of osteoarthritis. *Arthritis* 2015; 2015:708152.

(31) Riecke BF, Christensen R, Christensen P, Leeds AR, Boesen M, Lohmander LS et al. Comparing two low-energy diets for the treatment of knee osteoarthritis symptoms in obese patients: a pragmatic randomized clinical trial. *Osteoarthritis Cartilage* 2010; 18(6):746-754.

(32) Lopez-Gomez JJ, Izaola-Jauregui O, Torres-Torres B, Gomez-Hoyos E, Castro Lozano MA, Ortola-Buigues A et al. Influence of a meal-replacement diet on quality of life in women with obesity and knee osteoarthritis before orthopedic surgery. *Nutr Hosp* 2018; 35(1):71-77.

(33) Messier SP, Mihalko SL, Legault C, Miller GD, Nicklas BJ, DeVita P et al. Effects of intensive diet and exercise on knee joint loads, inflammation, and clinical outcomes among overweight and obese adults with knee osteoarthritis: the IDEA randomized clinical trial. *JAMA* 2013; 310(12):1263-1273.

(34) Dai Z, Lu N, Niu J, Felson DT, Zhang Y. Dietary Fiber Intake in Relation to Knee Pain Trajectory. *Arthritis Care Res (Hoboken)* 2017; 69(9):1331-1339.

(35) Lu B, Driban JB, Xu C, Lapane KL, McAlindon TE, Eaton CB. Dietary Fat Intake and Radiographic Progression of Knee Osteoarthritis: Data From the Osteoarthritis Initiative. *Arthritis Care Res (Hoboken)* 2017; 69(3):368-375.

(36) Daily JW, Yang M, Park S. Efficacy of Turmeric Extracts and Curcumin for Alleviating the Symptoms of Joint Arthritis: A Systematic Review and Meta-Analysis of Randomized Clinical Trials. *J Med Food* 2016; 19(8):717-729.

(37) Cameron M, Chrubasik S. Oral herbal therapies for treating osteoarthritis. *Cochrane Database Syst Rev* 2014;(5):CD002947.

(38) Percope de Andrade MA, Campos TV, Abreu-E-Silva GM. Supplementary methods in the nonsurgical treatment of osteoarthritis. *Arthroscopy* 2015; 31(4):785-792.

(39) McAlindon TE, Bannuru RR, Sullivan MC, Arden NK, Berenbaum F, Bierma-Zeinstra SM et al. OARSI guidelines for the non-surgical management of knee osteoarthritis. *Osteoarthritis Cartilage* 2014; 22(3):363-388.

(40) Hashempur MH, Sadrneshin S, Mosavat SH, Ashraf A. Green tea (Camellia sinensis) for patients with knee osteoarthritis: A randomized open-label active-controlled clinical trial. *Clin Nutr* 2018; 37(1):85-90.

(41) Salimzadeh A, Alipoor E, Dehghani S, Yaseri M, Hosseini M, Feinle-Bisset C et al. The effect of 12-week garlic supplementation on symptom relief in overweight or obese women with knee osteoarthritis. *Int J Clin Pract* 2018; 72(6):e13208.

(42) Essouiri J, Harzy T, Benaicha N, Errasfa M, Abourazzak FE. Effectiveness of Argan Oil Consumption on Knee Osteoarthritis Symptoms: A Randomized Controlled Clinical Trial. *Curr Rheumatol Rev* 2017; 13(3):231-235.

(43) Karimifar M, Soltani R, Hajhashemi V, Sarrafchi S. Evaluation of the effect of Elaeagnus angustifolia alone and combined with Boswellia thurifera compared with ibuprofen in patients with knee osteoarthritis: a randomized double-blind controlled clinical trial. *Clin Rheumatol* 2017; 36(8):1849-1853.

(44) More M, Gruenwald J, Pohl U, Uebelhack R. A Rosa canina - Urtica dioica - Harpagophytum procumbens/zeyheri Combination Significantly Reduces Gonarthritis Symptoms in a Randomized, Placebo-Controlled Double-Blind Study. *Planta Med* 2017; 83(18):1384-1391.

(45) Rafraf M, Hemmati S, Jafarabadi M, Moghaddam A, Haghighian M. Pomegranate (Punica Granatum L.) peel hydroalcoholic extract supplementation reduces pain and improves clinical symptoms of knee osteoarthritis: a randomized double-blind placebo controlled study. *Iran Red Crescent Med J* 2017; 19(1).

(46) Ghoochani N, Karandish M, Mowla K, Haghighizadeh MH, Jalali MT. The effect of pomegranate juice on clinical signs, matrix metalloproteinases and antioxidant status in patients with knee osteoarthritis. *J Sci Food Agric* 2016; 96(13):4377-4381.

(47) Haghighian M., Alipoor B, Malek MA, Eftekhar SB, Asghari JM, Moghaddam A. Effects of sesame seed supplementation on inflammatory factors and oxidative stress biomarkers in patients with knee osteoarthritis. *Acta Med Iran* 2015; 53(4):207-213.

(48) Arjmandi BH, Ormsbee LT, Elam ML, Campbell SC, Rahnama N, Payton ME et al. A combination of Scutellaria baicalensis and Acacia catechu extracts for short-term symptomatic relief of joint discomfort associated with osteoarthritis of the knee. *J Med Food* 2014; 17(6):707-713.

(49) Ebrahimi A, Nikniaz Z, Ostadrahimi A, Mahdavi R, Nikniaz L. The effect of Elaeagnus angustifolia L. whole fruit and medulla powder on women with osteoarthritis of the knee: A randomized controlled clinical trial. *European Journal of Integrative Medicine* 2014; 6(6):672-679.

(50) Eftekhar SB, Khadem HM, Alipoor B, Malek MA, Asghari JM, Moghaddam A. Effects of sesame seed supplementation on clinical signs and symptoms in patients with knee osteoarthritis. *Int J Rheum Dis* 2013; 16(5):578-582.

(51) Paramdeep G. Efficacy and tolerability of ginger (Zingiber officinale) in patients of osteoarthritis of knee. *Indian J Physiol Pharmacol* 2013; 57(2):177-183.

(52) Schumacher HR, Pullman-Mooar S, Gupta SR, Dinnella JE, Kim R, McHugh MP. Randomized double-blind crossover study of the efficacy of a tart cherry juice blend in treatment of osteoarthritis (OA) of the knee. *Osteoarthritis Cartilage* 2013; 21(8):1035-1041.

(53) Kuehl K, Elliot D, Sleigh A, Smith J. Efficacy of Tart Cherry Juice to Reduce Inflammation Biomarkers among Women with Inflammatory Osteoarthritis (OA). *Journal of Food Studies* 2012; 1(1):14-25.

(54) Myers SP, O'Connor J, Fitton JH, Brooks L, Rolfe M, Connellan P et al. A combined phase I and II open label study on the effects of a seaweed extract nutrient complex on osteoarthritis. *Biologics* 2010; 4:33-44.

(55) Frestedt JL, Kuskowski MA, Zenk JL. A natural seaweed derived mineral supplement (Aquamin F) for knee osteoarthritis: a randomised, placebo controlled pilot study. *Nutr J* 2009; 8:7.

(56) Oben J, Enonchong E, Kothari S, Chambliss W, Garrison R, Dolnick D. Phellodendron and Citrus extracts benefit joint health in osteoarthritis patients: a pilot, double-blind, placebo-controlled study. *Nutr J* 2009; 8:38.

(57) Rein E, Kharazmi A, Winther K. A herbal remedy, Hyben Vital (stand. powder of a subspecies of Rosa canina fruits), reduces pain and improves general wellbeing in patients with osteoarthritis--a double-blind, placebo-controlled, randomised trial. *Phytomedicine* 2004; 11(5):383-391.

(58) Warholm O, Skaar S, Hedman E, Molmen HM, Eik L. The Effects of a Standardized Herbal Remedy Made from a Subtype of Rosa canina in Patients with Osteoarthritis: A Double-Blind, Randomized, Placebo-Controlled Clinical Trial. *Curr Ther Res Clin Exp* 2003; 64(1):21-31.

(59) Piscoya J, Rodriguez Z, Bustamante SA, Okuhama NN, Miller MJ, Sandoval M. Efficacy and safety of freeze-dried cat's claw in osteoarthritis of the knee: mechanisms of action of the species Uncaria guianensis. *Inflamm Res* 2001; 50(9):442-448.

(60) Hunt S, Stebbings S, McNamara D. An open-label six-month extension study to investigate the safety and efficacy of an extract of Artemisia annua for managing pain, stiffness and functional limitation associated with osteoarthritis of the hip and knee. *N Z Med J* 2016; 129(1444):97-102.

(61) Wan X, Wang W, Liu J, Tong T. Estimating the sample mean and standard deviation from the sample size, median, range and/or interquartile range. *BMC Med Res Methodol* 2014; 14:135.

(62) Singh JA, Noorbaloochi S, MacDonald R, Maxwell LJ. Chondroitin for osteoarthritis. *Cochrane Database Syst Rev* 2015; 1:CD005614.

(63) Gallagher B, Tjoumakaris FP, Harwood MI, Good RP, Ciccotti MG, Freedman KB. Chondroprotection and the prevention of osteoarthritis progression of the knee: a systematic review of treatment agents. *Am J Sports Med* 2015; 43(3):734-744.

(64) Lei M, Guo C, Wang D, Zhang C, Hua L. The effect of probiotic Lactobacillus casei Shirota on knee osteoarthritis: a randomised double-blind, placebo-controlled clinical trial. *Benef Microbes* 2017; 8(5):697-703.

(65) Neves M, Jr., Gualano B, Roschel H, Fuller R, Benatti FB, Pinto AL et al. Beneficial effect of creatine supplementation in knee osteoarthritis. *Med Sci Sports Exerc* 2011; 43(8):1538-1543.

(66) Scorei R, Mitrut P, Petrisor I, Scorei I. A double-blind, placebo-controlled pilot study to evaluate the effect of calcium fructoborate on systemic inflammation and dyslipidemia markers for middle-aged people with primary osteoarthritis. *Biol Trace Elem Res* 2011; 144(1-3):253-263.

(67) Roy BD, de BJ, Harvey D, Tarnopolsky MA. Creatine monohydrate supplementation does not improve functional recovery after total knee arthroplasty. *Arch Phys Med Rehabil* 2005; 86(7):1293-1298.

(68) Bansal H, Bansal A, Agrawal D, Singh D, Deb K. Chondroprotection using naturally occurring mineral supplementation formula in degenerative osteoarthritis of the knees. *J Stem Cells* 2014; 9(1):65-76.

(69) Diao N, Yang B, Yu F. Effect of vitamin D supplementation on knee osteoarthritis: A systematic review and meta-analysis of randomized clinical trials. *Clin Biochem* 2017; 50(18):1312-1316.

(70) Gao XR, Chen YS, Deng W. The effect of vitamin D supplementation on knee osteoarthritis: A meta-analysis of randomized controlled trials. *Int J Surg* 2017; 46:14-20.

(71) Hussain S, Singh A, Akhtar M, Najmi AK. Vitamin D supplementation for the management of knee osteoarthritis: a systematic review of randomized controlled trials. *Rheumatol Int* 2017; 37(9):1489-1498.

(72) Bastick AN, Belo JN, Runhaar J, Bierma-Zeinstra SM. What Are the Prognostic Factors for Radiographic Progression of Knee Osteoarthritis? A Meta-analysis. *Clin Orthop Relat Res* 2015; 473(9):2969-2989.

(73) Bischoff-Ferrari HA, Orav EJ, Egli A, Dawson-Hughes B, Fischer K, Staehelin HB et al. Recovery after unilateral knee replacement due to severe osteoarthritis and progression in the contralateral knee: a randomised clinical trial comparing daily 2000 IU versus 800 IU vitamin D. *RMD Open* 2018; 4(2):e000678.

(74) Arden NK, Cro S, Sheard S, Dore CJ, Bara A, Tebbs SA et al. The effect of vitamin D supplementation on knee osteoarthritis, the VIDEO study: a randomised controlled trial. *Osteoarthritis Cartilage* 2016; 24(11):1858-1866.

(75) Jin X, Jones G, Cicuttini F, Wluka A, Zhu Z, Han W et al. Effect of Vitamin D Supplementation on Tibial Cartilage Volume and Knee Pain Among Patients With Symptomatic Knee Osteoarthritis: A Randomized Clinical Trial. *JAMA* 2016; 315(10):1005-1013.

(76) McAlindon T, LaValley M, Schneider E, Nuite M, Lee JY, Price LL et al. Effect of vitamin D supplementation on progression of knee pain and cartilage volume loss in patients with symptomatic osteoarthritis: a randomized controlled trial. *JAMA* 2013; 309(2):155-162.

(77) Medhi B, Manpreet S, Deonis X, Aggarwal S, Pandhi P, Nagi O. Comparative clinical trial of paracetamol alone and vitamin C and E as an add on therapy in patients suffering from primary knee osteoarthritis. *JK Science* 2012; 14(1):38-42.

(78) Colker CM, Swain M, Lynch L, Gingerich DA. Effects of a milk-based bioactive micronutrient beverage on pain symptoms and activity of adults with osteoarthritis: a double-blind, placebo-controlled clinical evaluation. *Nutrition* 2002; 18(5):388-392.

(79) Jonas WB, Rapoza CP, Blair WF. The effect of niacinamide on osteoarthritis: a pilot study. *Inflamm Res* 1996; 45(7):330-334.

(80) Flynn MA, Irvin W, Krause G. The effect of folate and cobalamin on osteoarthritic hands. *J Am Coll Nutr* 1994; 13(4):351-356.

(81) Peregoy J, Wilder FV. The effects of vitamin C supplementation on incident and progressive knee osteoarthritis: a longitudinal study. *Public Health Nutr* 2011; 14(4):709-715.

(82) Wilder F, Leaverton P, Rogers M, Lemrow N. Vitamin supplements and radiographic knee osteoarthritis: The Clearwater Osteoarthritis Study. *Journal of Musculoskeletal Research* 2009; 12(02):85-93.

(83) McAlindon TE, Felson DT, Zhang Y, Hannan MT, Aliabadi P, Weissman B et al. Relation of dietary intake and serum levels of vitamin D to progression of osteoarthritis of the knee among participants in the Framingham Study. *Ann Intern Med* 1996; 125(5):353-359.

(84) Gioxari A, Kaliora AC, Marantidou F, Panagiotakos DP. Intake of omega-3 polyunsaturated fatty acids in patients with rheumatoid arthritis: A systematic review and meta-analysis. *Nutrition* 2018; 45:114-124.

(85) Cramp F, Hewlett S, Almeida C, Kirwan JR, Choy EH, Chalder T et al. Non-pharmacological interventions for fatigue in rheumatoid arthritis. *Cochrane Database Syst Rev* 2013;(8):CD008322.

(86) Abdulrazaq M, Innes JK, Calder PC. Effect of omega-3 polyunsaturated fatty acids on arthritic pain: A systematic review. *Nutrition* 2017; 39-40:57-66.

(87) Lindqvist HM, Gjertsson I, Eneljung T, Winkvist A. Influence of Blue Mussel (Mytilus edulis) Intake on Disease Activity in Female Patients with Rheumatoid Arthritis: The MIRA Randomized Cross-Over Dietary Intervention. *Nutrients* 2018; 10(4).

(88) Fu Y, Li G, Zhang X, Xing G, Hu X, Yang L et al. Lipid extract from hard-shelled mussel (Mytilus coruscus) improves clinical conditions of patients with rheumatoid arthritis: a randomized controlled trial. *Nutrients* 2015; 7(1):625-645.

(89) Rajaei E, Mowla K, Ghorbani A, Bahadoram S, Bahadoram M, Dargahi-Malamir M. The Effect of Omega-3 Fatty Acids in Patients With Active Rheumatoid Arthritis Receiving DMARDs Therapy: Double-Blind Randomized Controlled Trial. *Glob J Health Sci* 2015; 8(7):18-25.

(90) Reed GW, Leung K, Rossetti RG, Vanbuskirk S, Sharp JT, Zurier RB. Treatment of rheumatoid arthritis with marine and botanical oils: an 18-month, randomized, and double-blind trial. *Evid Based Complement Alternat Med* 2014; 2014:857456.

(91) Arborelius M, Jr., Konttinen YT, Nordstrom DC, Solovieva SA. Gly-X-Y repeat sequences in the treatment of active rheumatoid arthritis. *Rheumatol Int* 1999; 18(4):129-135.

(92) Skoldstam L, Borjesson O, Kjallman A, Seiving B, Akesson B. Effect of six months of fish oil supplementation in stable rheumatoid arthritis. A double-blind, controlled study. *Scand J Rheumatol* 1992; 21(4):178-185.

(93) Tulleken JE, Limburg PC, Muskiet FA, van Rijswijk MH. Vitamin E status during dietary fish oil supplementation in rheumatoid arthritis. *Arthritis Rheum* 1990; 33(9):1416-1419.

(94) van der Tempel H, Tulleken JE, Limburg PC, Muskiet FA, van Rijswijk MH. Effects of fish oil supplementation in rheumatoid arthritis. *Ann Rheum Dis* 1990; 49(2):76-80.

(95) Cleland LG, French JK, Betts WH, Murphy GA, Elliott MJ. Clinical and biochemical effects of dietary fish oil supplements in rheumatoid arthritis. *J Rheumatol* 1988; 15(10):1471-1475.

(96) Magaro M, Altomonte L, Zoli A, Mirone L, De SP, Di MG et al. Influence of diet with different lipid composition on neutrophil chemiluminescence and disease activity in patients with rheumatoid arthritis. *Ann Rheum Dis* 1988; 47(10):793-796.

(97) Kremer JM, Jubiz W, Michalek A, Rynes RI, Bartholomew LE, Bigaouette J et al. Fish-oil fatty acid supplementation in active rheumatoid arthritis. A double-blinded, controlled, crossover study. *Ann Intern Med* 1987; 106(4):497-503.

(98) Cleland LG, Caughey GE, James MJ, Proudman SM. Reduction of cardiovascular risk factors with longterm fish oil treatment in early rheumatoid arthritis. *J Rheumatol* 2006; 33(10):1973-1979.

(99) Podas T, Nightingale JM, Oldham R, Roy S, Sheehan NJ, Mayberry JF. Is rheumatoid arthritis a disease that starts in the intestine? A pilot study comparing an elemental diet with oral prednisolone. *Postgrad Med J* 2007; 83(976):128-131.

(100) Skoldstam L, Hagfors L, Johansson G. An experimental study of a Mediterranean diet intervention for patients with rheumatoid arthritis. *Ann Rheum Dis* 2003; 62(3):208-214.

(101) Hafstrom I, Ringertz B, Spangberg A, von ZL, Brannemark S, Nylander I et al. A vegan diet free of gluten improves the signs and symptoms of rheumatoid arthritis: the effects on arthritis correlate with a reduction in antibodies to food antigens. *Rheumatology (Oxford)* 2001; 40(10):1175-1179.

(102) Sarzi-Puttini P, Comi D, Boccassini L, Muzzupappa S, Turiel M, Panni B et al. Diet therapy for rheumatoid arthritis. A controlled double-blind study of two different dietary regimens. *Scand J Rheumatol* 2000; 29(5):302-307.

(103) Holst-Jensen SE, Pfeiffer-Jensen M, Monsrud M, Tarp U, Buus A, Hessov I et al. Treatment of rheumatoid arthritis with a peptide diet: a randomized, controlled trial. *Scand J Rheumatol* 1998; 27(5):329-336.

(104) Nenonen MT, Helve TA, Rauma AL, Hanninen OO. Uncooked, lactobacilli-rich, vegan food and rheumatoid arthritis. *Br J Rheumatol* 1998; 37(3):274-281.

(105) Kavanaghi R, Workman E, Nash P, Smith M, Hazleman BL, Hunter JO. The effects of elemental diet and subsequent food reintroduction on rheumatoid arthritis. *Br J Rheumatol* 1995; 34(3):270-273.

(106) Haugen MA, Kjeldsen-Kragh J, Forre O. A pilot study of the effect of an elemental diet in the management of rheumatoid arthritis. *Clin Exp Rheumatol* 1994; 12(3):275-279.

(107) van de Laar MA, van der Korst JK. Food intolerance in rheumatoid arthritis. I. A double blind, controlled trial of the clinical effects of elimination of milk allergens and azo dyes. *Ann Rheum Dis* 1992; 51(3):298-302.

(108) Panush RS, Carter RL, Katz P, Kowsari B, Longley S, Finnie S. Diet therapy for rheumatoid arthritis. *Arthritis Rheum* 1983; 26(4):462-471.

(109) Sundqvist T, Lindstrom F, Magnusson KE, Skoldstam L, Stjernstrom I, Tagesson C. Influence of fasting on intestinal permeability and disease activity in patients with rheumatoid arthritis. *Scand J Rheumatol* 1982; 11(1):33-38.

(110) Skoldstam L, Larsson L, Lindstrom FD. Effect of fasting and lactovegetarian diet on rheumatoid arthritis. *Scand J Rheumatol* 1979; 8(4):249-255.

(111) Abendroth A, Michalsen A, Ludtke R, Ruffer A, Musial F, Dobos GJ et al. Changes of Intestinal Microflora in Patients with Rheumatoid Arthritis during Fasting or a Mediterranean Diet. *Forsch Komplementmed* 2010; 17(6):307-313.

(112) McKellar G, Morrison E, McEntegart A, Hampson R, Tierney A, Mackle G et al. A pilot study of a Mediterranean-type diet intervention in female patients with rheumatoid arthritis living in areas of social deprivation in Glasgow. *Ann Rheum Dis* 2007; 66(9):1239-1243.

(113) Adam O, Beringer C, Kless T, Lemmen C, Adam A, Wiseman M et al. Anti-inflammatory effects of a low arachidonic acid diet and fish oil in patients with rheumatoid arthritis. *Rheumatol Int* 2003; 23(1):27-36.

(114) Fraser DA, Thoen J, Djoseland O, Forre O, Kjeldsen-Kragh J. Serum levels of interleukin-6 and dehydroepiandrosterone sulphate in response to either fasting or a ketogenic diet in rheumatoid arthritis patients. *Clin Exp Rheumatol* 2000; 18(3):357-362.

(115) Denissov LN, Sharafetdinov K, Samsonov MA. On the medicinal efficacy of dietetic therapy in patients with rheumatoid arthritis. *Int J Clin Pharmacol Res* 1992; 12(1):19-25.

(116) McDougall J, Bruce B, Spiller G, Westerdahl J, McDougall M. Effects of a very low-fat, vegan diet in subjects with rheumatoid arthritis. *J Altern Complement Med* 2002; 8(1):71-75.

(117) Kjeldsen-Kragh J, Haugen M, Borchgrevink CF, Forre O. Vegetarian diet for patients with rheumatoid arthritis--status: two years after introduction of the diet. *Clin Rheumatol* 1994; 13(3):475-482.

(118) Dawczynski C, Dittrich M, Neumann T, Goetze K, Welzel A, Oelzner P et al. Docosahexaenoic acid in the treatment of rheumatoid arthritis: A double-blind, placebo-controlled, randomized cross-over study with microalgae vs. sunflower oil. *Clin Nutr* 2018; 37(2):494-504.

(119) Ghavipour M, Sotoudeh G, Tavakoli E, Mowla K, Hasanzadeh J, Mazloom Z. Pomegranate extract alleviates disease activity and some blood biomarkers of inflammation and oxidative stress in Rheumatoid Arthritis patients. *Eur J Clin Nutr* 2017; 71(1):92-96.

(120) Javadi F, Ahmadzadeh A, Eghtesadi S, Aryaeian N, Zabihiyeganeh M, Rahimi FA et al. The Effect of Quercetin on Inflammatory Factors and Clinical Symptoms in Women with Rheumatoid Arthritis: A Double-Blind, Randomized Controlled Trial. *J Am Coll Nutr* 2017; 36(1):9-15.

(121) Hemmati A, Rajaee E, Houshmand G, Fakroddin M, Amir M, Hesam S et al. Study the effects of anti-inflammatory curcumex capsules containing three plants (ginger, curcumin and black pepper) in patients with active rheumatoid arthritis. *The IIOAB Journal* 2016; 7(Suppl 5):389-392.

(122) Javadi F, Eghtesadi S, Ahmadzadeh A, Aryaeian N, Zabihiyeganeh M, Foroushani AR et al. The effect of quercetin on plasma oxidative status, C-reactive protein and blood pressure in women with rheumatoid arthritis. *Int J Prev Med* 2014; 5(3):293-301.

(123) Willich SN, Rossnagel K, Roll S, Wagner A, Mune O, Erlendson J et al. Rose hip herbal remedy in patients with rheumatoid arthritis - a randomised controlled trial. *Phytomedicine* 2010; 17(2):87-93.

(124) Bae SC, Jung WJ, Lee EJ, Yu R, Sung MK. Effects of antioxidant supplements intervention on the level of plasma inflammatory molecules and disease severity of rheumatoid arthritis patients. *J Am Coll Nutr* 2009; 28(1):56-62.

(125) Li EK, Tam LS, Wong CK, Li WC, Lam CW, Wachtel-Galor S et al. Safety and efficacy of Ganoderma lucidum (lingzhi) and San Miao San supplementation in patients with rheumatoid arthritis: a double-blind, randomized, placebo-controlled pilot trial. *Arthritis Rheum* 2007; 57(7):1143-1150.

(126) Gheita TA, Kenawy SA. Effectiveness of Nigella sativa oil in the management of rheumatoid arthritis patients: a placebo controlled study. *Phytother Res* 2012; 26(8):1246-1248.

(127) Kamal E, Kaddam LA, Dahawi M, Osman M, Salih MA, Alagib A et al. Gum Arabic Fibers Decreased Inflammatory Markers and Disease Severity Score among Rheumatoid Arthritis Patients, Phase II Trial. *Int J Rheumatol* 2018; 2018:4197537.

(128) Kumar G, Srivastava A, Sharma SK, Rao TD, Gupta YK. Efficacy & safety evaluation of Ayurvedic treatment (Ashwagandha powder & Sidh Makardhwaj) in rheumatoid arthritis patients: a pilot prospective study. *Indian J Med Res* 2015; 141(1):100-106.

(129) Matsuno H, Nakamura H, Katayama K, Hayashi S, Kano S, Yudoh K et al. Effects of an oral administration of glucosamine-chondroitin-quercetin glucoside on the synovial fluid properties in patients with osteoarthritis and rheumatoid arthritis. *Biosci Biotechnol Biochem* 2009; 73(2):288-292.

(130) Aqaeinezhad Rudbane SM, Rahmdel S, Abdollahzadeh SM, Zare M, Bazrafshan A, Mazloomi SM. The efficacy of probiotic supplementation in rheumatoid arthritis: a meta-analysis of randomized, controlled trials. *Inflammopharmacology* 2018; 26(1):67-76.

(131) Mohammed AT, Khattab M, Ahmed AM, Turk T, Sakr N, Khalil M et al. The therapeutic effect of probiotics on rheumatoid arthritis: a systematic review and meta-analysis of randomized control trials. *Clin Rheumatol* 2017; 36(12):2697-2707.

(132) Zamani B, Farshbaf S, Golkar HR, Bahmani F, Asemi Z. Synbiotic supplementation and the effects on clinical and metabolic responses in patients with rheumatoid arthritis: a randomised, double-blind, placebo-controlled trial. *Br J Nutr* 2017; 117(8):1095-1102.

(133) Wilkinson TJ, Lemmey AB, Jones JG, Sheikh F, Ahmad YA, Chitale S et al. Can Creatine Supplementation Improve Body Composition and Objective Physical Function in Rheumatoid Arthritis Patients? A Randomized Controlled Trial. *Arthritis Care Res (Hoboken)* 2016; 68(6):729-737.

(134) Abdollahzad H, Alipour B, Aghdashi M, Jafarabadi M. Coenzyme Q10 supplementation in patients with rheumatoid arthritis: Are there any effects on cardiovascular risk factors? *European Journal of Integrative Medicine* 2015; 7(5):534-539.

(135) Mirtaheri E, Gargari BP, Kolahi S, Dehghan P, Asghari-Jafarabadi M, Hajalilou M et al. Effects of Alpha-Lipoic Acid Supplementation on Inflammatory Biomarkers and Matrix Metalloproteinase-3 in Rheumatoid Arthritis Patients. *J Am Coll Nutr* 2015; 34(4):310-317.

(136) Alavi A, Goodfellow L, Fraser O, Tarelli E, Bland M, Axford J. A double-blind, randomized, placebo-controlled study to explore the efficacy of a dietary plant-derived polysaccharide supplement in patients with rheumatoid arthritis. *Rheumatology (Oxford)* 2011; 50(6):1111-1119.

(137) Aryaeian N, Shahram F, Djalali M, Eshragian MR, Djazayeri A, Sarrafnejad A et al. Effect of conjugated linoleic acid, vitamin E and their combination on lipid profiles and blood pressure of Iranian adults with active rheumatoid arthritis. *Vasc Health Risk Manag* 2008; 4(6):1423-1432.

(138) Rastmanesh R, Abargouei AS, Shadman Z, Ebrahimi AA, Weber CE. A pilot study of potassium supplementation in the treatment of hypokalemic patients with rheumatoid arthritis: a randomized, double-blinded, placebo-controlled trial. *J Pain* 2008; 9(8):722-731.

(139) Nakamura H, Masuko K, Yudoh K, Kato T, Kamada T, Kawahara T. Effects of glucosamine administration on patients with rheumatoid arthritis. *Rheumatol Int* 2007; 27(3):213-218.

(140) Marcora S, Lemmey A, Maddison P. Dietary treatment of rheumatoid cachexia with beta-hydroxy-beta-methylbutyrate, glutamine and arginine: a randomised controlled trial. *Clin Nutr* 2005; 24(3):442-454.

(141) Mattingly PC, Mowat AG. Zinc sulphate in rheumatoid arthritis. *Ann Rheum Dis* 1982; 41(5):456-457.

(142) Simkin PA. Oral zinc sulphate in rheumatoid arthritis. *Lancet* 1976; 2(7985):539-542.

(143) Bepler C, Rogers F. A double blind study using manganese against placebo in rheumatoid arthritis. *Am J Med Sci* 1957; 234(4):459-461.

(144) Rasker JJ, Kardaun SH. Lack of beneficial effect of zinc sulphate in rheumatoid arthritis. *Scand J Rheumatol* 1982; 11(3):168-170.

(145) Franco AS, Freitas TQ, Bernardo WM, Pereira RMR. Vitamin D supplementation and disease activity in patients with immune-mediated rheumatic diseases: A systematic review and meta-analysis. *Medicine (Baltimore)* 2017; 96(23):e7024.

(146) Batooei M, Tahamoli-Roudsari A, Basiri Z, Yasrebifar F, Shahdoust M, Eshraghi A et al. Evaluating the Effect of Oral N-acetylcysteine as an Adjuvant Treatment on Clinical Outcomes of Patients with Rheumatoid Arthritis: A Randomized, Double Blind Clinical Trial. *Rev Recent Clin Trials* 2018; 13(2):132-138.

(147) Huang SC, Wei JC, Wu DJ, Huang YC. Vitamin B(6) supplementation improves pro-inflammatory responses in patients with rheumatoid arthritis. *Eur J Clin Nutr* 2010; 64(9):1007-1013.

(148) Nourmohammadi I, Athari-Nikazm S, Vada M, Bidari A, Jazayeri S, Hoshyarrad A et al. Effects of antioxidant supplementations on oxidative stress in rheumatoid arthritis patients. *Journal of Biological Sciences* 2010; 10(1):63-66.

(149) Chiang EP, Selhub J, Bagley PJ, Dallal G, Roubenoff R. Pyridoxine supplementation corrects vitamin B6 deficiency but does not improve inflammation in patients with rheumatoid arthritis. *Arthritis Res Ther* 2005; 7(6):R1404-R1411.

(150) Edmonds SE, Winyard PG, Guo R, Kidd B, Merry P, Langrish-Smith A et al. Putative analgesic activity of repeated oral doses of vitamin E in the treatment of rheumatoid arthritis. Results of a prospective placebo controlled double blind trial. *Ann Rheum Dis* 1997; 56(11):649-655.

(151) Helmy M, Shohayeb M, Helmy MH, el-Bassiouni EA. Antioxidants as adjuvant therapy in rheumatoid disease. A preliminary study. *Arzneimittelforschung* 2001; 51(4):293-298.

(152) Jalili M, Kolahi S, Aref-Hosseini SR, Mamegani ME, Hekmatdoost A. Beneficial role of antioxidants on clinical outcomes and erythrocyte antioxidant parameters in rheumatoid arthritis patients. *Int J Prev Med* 2014; 5(7):835-840.

(153) van Vugt RM, Rijken PJ, Rietveld AG, van Vugt AC, Dijkmans BA. Antioxidant intervention in rheumatoid arthritis: results of an open pilot study. *Clin Rheumatol* 2008; 27(6):771-775.

(154) Rodriguez Huerta MD, Trujillo-Martin MM, Rua-Figueroa I, Cuellar-Pompa L, Quiros-Lopez R, Serrano-Aguilar P. Healthy lifestyle habits for patients with systemic lupus erythematosus: A systemic review. *Semin Arthritis Rheum* 2016; 45(4):463-470.

(155) Curado Borges M., de Miranda Moura Dos Santos, Weiss TR, Melo de Andrade MV, Toulson Davisson Correia MI, Lanna CCD. Omega-3 fatty acids, inflammatory status and biochemical markers of patients with systemic lupus erythematosus: a pilot study. *Rev Bras Reumatol Engl Ed* 2017; 57(6):526-534.

(156) Arriens C, Hynan LS, Lerman RH, Karp DR, Mohan C. Placebo-controlled randomized clinical trial of fish oil's impact on fatigue, quality of life, and disease activity in Systemic Lupus Erythematosus. *Nutr J* 2015; 14:82.

(157) Bello KJ, Fang H, Fazeli P, Bolad W, Corretti M, Magder LS et al. Omega-3 in SLE: a double-blind, placebo-controlled randomized clinical trial of endothelial dysfunction and disease activity in systemic lupus erythematosus. *Rheumatol Int* 2013; 33(11):2789-2796.

(158) Wright SA, O'Prey FM, McHenry MT, Leahey WJ, Devine AB, Duffy EM et al. A randomised interventional trial of omega-3-polyunsaturated fatty acids on endothelial function and disease activity in systemic lupus erythematosus. *Ann Rheum Dis* 2008; 67(6):841-848.

(159) Duffy EM, Meenagh GK, McMillan SA, Strain JJ, Hannigan BM, Bell AL. The clinical effect of dietary supplementation with omega-3 fish oils and/or copper in systemic lupus erythematosus. *J Rheumatol* 2004; 31(8):1551-1556.

(160) Westberg G, Tarkowski A. Effect of MaxEPA in patients with SLE. A double-blind, crossover study. *Scand J Rheumatol* 1990; 19(2):137-143.

(161) Lozovoy MA, Simao AN, Morimoto HK, Scavuzzi BM, Iriyoda TV, Reiche EM et al. Fish oil N-3 fatty acids increase adiponectin and decrease leptin levels in patients with systemic lupus erythematosus. *Mar Drugs* 2015; 13(2):1071-1083.

(162) del Pino-Sedeno T, Trujillo-Martin MM, Ruiz-Irastorza G, Cuellar-Pompa L, de Pascual-Medina AM, Serrano-Aguilar P. Effectiveness of Nonpharmacologic Interventions for Decreasing Fatigue in Adults With Systemic Lupus Erythematosus: A Systematic Review. *Arthritis Care Res (Hoboken)* 2016; 68(1):141-148.

(163) Yuen HK, Cunningham MA. Optimal management of fatigue in patients with systemic lupus erythematosus: a systematic review. *Ther Clin Risk Manag* 2014; 10:775-786.

(164) Shah M, Kavanaugh A, Coyle Y, Adams-Huet B, Lipsky PE. Effect of a culturally sensitive cholesterol lowering diet program on lipid and lipoproteins, body weight, nutrient intakes, and quality of life in patients with systemic lupus erythematosus. *J Rheumatol* 2002; 29(10):2122-2128.

(165) Minami Y, Hirabayashi Y, Nagata C, Ishii T, Harigae H, Sasaki T. Intakes of vitamin B6 and dietary fiber and clinical course of systemic lupus erythematosus: a prospective study of Japanese female patients. *J Epidemiol* 2011; 21(4):246-254.

(166) Minami Y, Sasaki T, Arai Y, Kurisu Y, Hisamichi S. Diet and systemic lupus erythematosus: a 4 year prospective study of Japanese patients. *J Rheumatol* 2003; 30(4):747-754.

(167) Karlson EW, Daltroy LH, Lew RA, Wright EA, Partridge AJ, Fossel AH et al. The relationship of socioeconomic status, race, and modifiable risk factors to outcomes in patients with systemic lupus erythematosus. *Arthritis Rheum* 1997; 40(1):47-56.

(168) Shamekhi Z, Amani R, Habibagahi Z, Namjoyan F, Ghadiri A, Saki MA. A Randomized, Double-blind, Placebo-controlled Clinical Trial Examining the Effects of Green Tea Extract on Systemic Lupus Erythematosus Disease Activity and Quality of Life. *Phytother Res* 2017; 31(7):1063-1071.

(169) Singgih Wahono C., Diah Setyorini C., Kalim H, Nurdiana N, Handono K. Effect of Curcuma xanthorrhiza Supplementation on Systemic Lupus Erythematosus Patients with Hypovitamin D Which Were Given Vitamin D3 towards Disease Activity (SLEDAI), IL-6, and TGF-beta1 Serum. *Int J Rheumatol* 2017; 2017:7687053.

(170) Al-Kushi AG, Azzeh FS, Header EA, ElSawy NA, Hijazi HH, Jazar AS et al. Effect of Vitamin D and Calcium Supplementation in Patients with Systemic Lupus Erythematosus. *Saudi J Med Med Sci* 2018; 6(3):137-142.

(171) Karimzadeh H, Shirzadi M, Karimifar M. The effect of Vitamin D supplementation in disease activity of systemic lupus erythematosus patients with Vitamin D deficiency: A randomized clinical trial. *J Res Med Sci* 2017; 22:4.

(172) Andreoli L, Dall'Ara F, Piantoni S, Zanola A, Piva N, Cutolo M et al. A 24-month prospective study on the efficacy and safety of two different monthly regimens of vitamin D supplementation in pre-menopausal women with systemic lupus erythematosus. *Lupus* 2015; 24(4-5):499-506.

(173) Macfarlane TV, Abbood HM, Pathan E, Gordon K, Hinz J, Macfarlane GJ. Relationship between diet and ankylosing spondylitis: A systematic review. *Eur J Rheumatol* 2018; 5(1):45-52.

(174) Jenks K, Stebbings S, Burton J, Schultz M, Herbison P, Highton J. Probiotic therapy for the treatment of spondyloarthritis: a randomized controlled trial. *J Rheumatol* 2010; 37(10):2118-2125.

(175) Kristensen S, Schmidt EB, Schlemmer A, Rasmussen C, Johansen MB, Christensen JH. Beneficial effect of n-3 polyunsaturated fatty acids on inflammation and analgesic use in psoriatic arthritis: a randomized, double blind, placebo-controlled trial. *Scand J Rheumatol* 2018; 47(1):27-36.

(176) Madland TM, Bjorkkjaer T, Brunborg LA, Froyland L, Berstad A, Brun JG. Subjective improvement in patients with psoriatic arthritis after short-term oral treatment with seal oil. A pilot study with double blind comparison to soy oil. *J Rheumatol* 2006; 33(2):307-310.

(177) Veale DJ, Torley HI, Richards IM, O'Dowd A, Fitzsimons C, Belch JJ et al. A double-blind placebo controlled trial of Efamol Marine on skin and joint symptoms of psoriatic arthritis. *Br J Rheumatol* 1994; 33(10):954-958.

(178) Kharaeva Z, Gostova E, De LC, Raskovic D, Korkina L. Clinical and biochemical effects of coenzyme Q(10), vitamin E, and selenium supplementation to psoriasis patients. *Nutrition* 2009; 25(3):295-302.

(179) Doerfler B, Allen TS, Southwood C, Brenner D, Hirano I, Sheean P. Medical Nutrition Therapy for Patients With Advanced Systemic Sclerosis (MNT PASS): A Pilot Intervention Study. *JPEN J Parenter Enteral Nutr* 2017; 41(4):678-684.

(180) Ortiz-Santamaria V, Puig C, Soldevillla C, Barata A, Cuquet J, Recasens A. Nutritional support in patients with systemic sclerosis. *Reumatol Clin* 2014; 10(5):283-287.

(181) Ostojic P, Damjanov N. Effects of micronutrient antioxidants (alpha-tocopherol and ascorbic acid) on skin thickening and lung function in patients with early diffuse systemic sclerosis. *Rheumatol Int* 2011; 31(8):1051-1054.

(182) Herrick AL, Hollis S, Schofield D, Rieley F, Blann A, Griffin K et al. A double-blind placebo-controlled trial of antioxidant therapy in limited cutaneous systemic sclerosis. *Clin Exp Rheumatol* 2000; 18(3):349-356.

(183) Hulshof MM, Bouwes Bavinck JN, Bergman W, Masclee AA, Heickendorff L, Breedveld FC et al. Double-blind, placebo-controlled study of oral calcitriol for the treatment of localized and systemic sclerosis. *J Am Acad Dermatol* 2000; 43(6):1017-1023.

(184) Andres M, Sivera F, Falzon L, Buchbinder R, Carmona L. Dietary supplements for chronic gout. *Cochrane Database Syst Rev* 2014;(10):CD010156.

(185) Moi JH, Sriranganathan MK, Edwards CJ, Buchbinder R. Lifestyle interventions for chronic gout. *Cochrane Database Syst Rev* 2013;(5):CD010039.

(186) Yu XN, Wu HY, Deng YP, Zhuang GT, Tan BH, Huang YZ et al. "Yellow-dragon Wonderful-seed Formula" for hyperuricemia in gout patients with dampness-heat pouring downward pattern: a pilot randomized controlled trial. *Trials* 2018; 19(1):551.

(187) Stamp LK, O'Donnell JL, Frampton C, Drake JM, Zhang M, Chapman PT. Clinically insignificant effect of supplemental vitamin C on serum urate in patients with gout: a pilot randomized controlled trial. *Arthritis Rheum* 2013; 65(6):1636-1642.

(188) Azzeh F, Al-Hebshi A, Al-Essimii H, Alarjah M. Vitamin C supplementation and serum uric acid: A reaction to hyperuricemia and gout disease. *PharmaNutrition* 2017; 5(2):47-51.

(189) Jantti J, Isomaki H, Laitinen O, Nikkari T, Seppala E, Vapaatalo H. Linoleic acid treatment in inflammatory arthritis. *Int J Clin Pharmacol Ther Toxicol* 1985; 23(2):89-91.

(190) Bradley MH, Golden E. Powdered meal replacement - can they benefit overweight patients with concomitant conditions exacerbated by obesity? *Current Therapeutic Research* 1990; 47(3):429-436.
